# Characterization of di‐gital, tri‐gital, and tetra‐gital temporal movement of systolic blood pressure on the arterial pulse waveform of rats at different vascular stiffness

**DOI:** 10.1002/ame2.70108

**Published:** 2025-12-11

**Authors:** Anton Misak, Lenka Tomasova, Marian Grman, Karol Ondrias

**Affiliations:** ^1^ Institute of Clinical and Translational Research, Biomedical Research Center Slovak Academy of Sciences Bratislava Slovak Republic

**Keywords:** arterial pulse waveform, di‐gital, rat, stiffness, systolic pressure fluctuation

## Abstract

**Background:**

An arterial stiffness is an indicator of many cardiovascular diseases. The temporal position of systolic blood pressure (BP) on aorta pulse waveform is assumed to gradually shift on the waveform in response to increasing/decreasing vascular stiffness. The animal model of rats and invasive methods that cannot be used in humans was applied to test the assumption on arterial pulse waveform (APW) of anesthetized rat. The aim of this study was to characterize the temporal movement of diastolic and systolic pressures on the APW of anesthetized rats during increasing/decreasing vascular stiffness.

**Methods:**

The right jugular vein of anesthetized normotensive and spontaneously hypertensive rats was cannulated for intravascular administration of vascularly active compounds to alter systolic pressure and vascular stiffness. The left carotid artery was cannulated to detect APW, from which numerous APW parameters were evaluated.

**Results:**

During increases/decreases in systolic BP or stiffness, the temporal position of diastolic BP of individual heartbeats di‐gitally shifted on the APW between two temporal positions ~8–12 ms apart, and the temporal position of systolic BP on the APW did not gradually shift during increases/decreases in vascular stiffness, as expected, but oscillated between constant di‐gital, tri‐gital, or tetra‐gital temporal positions.

**Conclusions:**

Introducing new APW parameters, *n*‐gital systolic BP fluctuations on rat APW were found. Fluctuations in *n*‐gital were approximately constant during large changes in systolic pressure despite significant changes in augmentation index and cardiovascular stiffness, which may challenge the assumption of a gradual temporal location of systolic pressure on rat APW under these conditions.

## Graphical abstract

The example of the time dependent fluctuation of systolic pressure on rat arterial pulse waveform in ms. The four levels of the systolic pressure fluctuation show di‐gital, tri‐gital and tetra‐gital fluctuation. The red dots represent particular heartbeats.

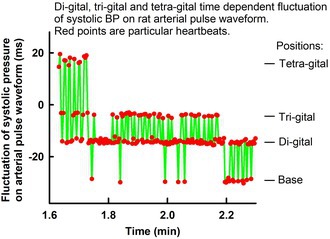

## 1 INTRODUCTION

Cardiovascular disease is a health problem and contributes significantly to global mortality.^1^, ^2^ The analysis of the detailed shape of a radial artery or arterial pulse waveform (APW) can provide information about many diseases. The more details of the APW are known, and more information can be obtained about the cardiovascular system (CVS).^3^, ^4^, ^5^, ^6^, ^7^, ^8^, ^9^, ^10^, ^11^, ^12^, ^13^, ^14^, ^15^, ^16^ Animal models and invasive methods that cannot be used in humans are widely used in experimental medicine research.^7^, ^17^, ^18^, ^19^ In our previous studies, we used invasive methods in a rat model to improve the methodology for obtaining details of APW parameters (APW‐Ps) at high resolution.^7^, ^20^

One important CVS parameter is aortic stiffness, which is associated with a higher risk of cardiovascular diseases and other chronic diseases of aging.^3^, ^10^, ^12^ Aortic stiffness is related to pulse wave velocity and can also be estimated from APW analysis, in which reflection waves play an important role. With high aortic stiffness, the reflected wave arrives early during systole and contributes to an increase in systolic pressure, opposing ventricular ejection. With no or low stiffness, the reflected wave comes after the systolic peak. The interaction between the forward and reflected pressure waves produces the overall pulse waveform.^3^, ^4^, ^5^, ^6^, ^9^, ^10^, ^11^, ^16^ Because arterial stiffening increases the propagation velocity of the pressure wave along the central arteries, leading to an earlier return of reflected waves, it is known that the position of the systolic pressure (maximum point on the pulse waveform) on the curve gradually shifts backward/forward as arterial stiffening increases/decreases. The aim of our study was to investigate the validity of this prediction on rat APW, how progressively increasing/decreasing stiffness affects the position of systolic pressure on the APW. Two models of increasing vascular stiffness were used. Stiffness in normotensive rats was induced by the nitric oxide synthase inhibitor L‐NAME (N_ω_‐nitro‐l‐arginine methyl ester hydrochloride), which reduces nitric oxide bioavailability and thus induces vascular stiffness.^21^, ^22^, ^23^ Spontaneously hypertensive (SHR) rats have been used as a natural animal model of vascular stiffness.^24^ From our previous studies^25^, ^26^ evaluating 35 rat APW‐Ps, we found that some APW‐Ps exhibited temporal jump (di‐gital) fluctuations. To study this phenomenon in relation to vascular stiffness, multiple APW‐Ps were introduced and from all 57 APW‐Ps, a few that showed temporal stepwise fluctuations in diastolic and systolic BP were selected.

## 2 MATERIALS AND METHODS

### 2.1 Chemicals and solutions

The following chemicals were purchased from Sigma‐Aldrich (Schnelldorf, Germany): sodium phosphate monobasic, sodium phosphate dibasic, sodium selenite, diethylenetriaminepentaacetic acid (DTPA), L‐NAME, nifedipine (NIF), acetylcholine chloride (ACE), and l‐(−)‐norepinephrine(+)‐bitartate salt monohydrate (NOR). Isoflurin (isoflurane [ISO]; 1000 mg/g) was purchased from Vetpharma (Barcelona, Spain). Stock solutions were prepared freshly in 0.9% NaCl: L‐NAME (50 mg/mL) and selenite (100 nmol/L). A stock solution of GSH (200 mmol/L) was prepared in 100 nmol/L sodium phoshate, 100 μmol/L DTPA, 7.4 pH buffer. NIF (100 nmol/L) was dissolved in DMSO and kept in dark for 0–2 days. The mixture was diluted with 0.9% NaCl just before administration. A mixture of glutathione/selenite (GS/Se) 75/12.5 in μmol/L was prepared by mixing 150 μL of 200 mmol/L glutathione with 50 μL of 100 mmol/L selenite and was incubated for 30 s (ISO‐anesthetized normotensive rats) and 45 s at 23 ± 1℃ (ISO‐anesthetized SHR rats).

### 2.2 Animals

Male normotensive Wistar (*n* = 16; 340 ± 40 g) and SHR rats (*n* = 6; 300 ± 30 g) were purchased from the Department of Toxicology and Laboratory Animal Breeding at Dobra Voda, Slovak Academy of Sciences, Slovakia. The rats were housed under a 12‐h light–12‐h dark cycle, at a constant humidity (45%–65%) and temperature (20–22℃), with free access to standard laboratory rat chow and drinking water. The veterinary nursing care was provided by the Central Animal Housing Facility of Pavilion of Medical Sciences (registration number: SK UCH 01017).

### 2.3 Experimental design

Two types of anesthesia were used. Normotensive rats were anesthetized with Zoletil 100 (tiletamine + zolazepam, 80 mg/kg, i.p.) and xylazine (5 mg/kg, i.p.).^20^ ISO was used for inhalational anesthesia of normotensive and SHR rats. Rats were placed in an induction chamber flushed with 5 vol% ISO in 100% oxygen until the loss of righting reflex. The rats were then placed on a heated pad (37℃), and 3 vol% ISO in 100% oxygen (0.9 L/min) was further administered using a nose cone during the experiments.^27^ The animals were under anesthesia throughout the duration of the experiment at 37℃ and euthanized at the end of surgery by an overdose of urethane injected into the heart. Continuous APW measurement, data analysis, and processing were performed as described.^20^, ^25^ The right jugular vein of the anesthetized rat was cannulated for intravascular administration of the compounds. The left common carotid artery was cannulated for insertion of pressure transducers (FISO LS 2F Harvard Apparatus, USA). The analog signal was low‐pass filtered at 1 kHz, digitized at 10 kHz, and analyzed to identify the 10 APW points (a–j) indicated in Figure S1. The definitions and abbreviations of the 57 and selected APW‐Ps parameters calculated from the APW points are given in Figure S1.

### 2.4 Definitions of di‐gital, tri‐gital, and tetra‐gital parameters

**FIGURE 1:**
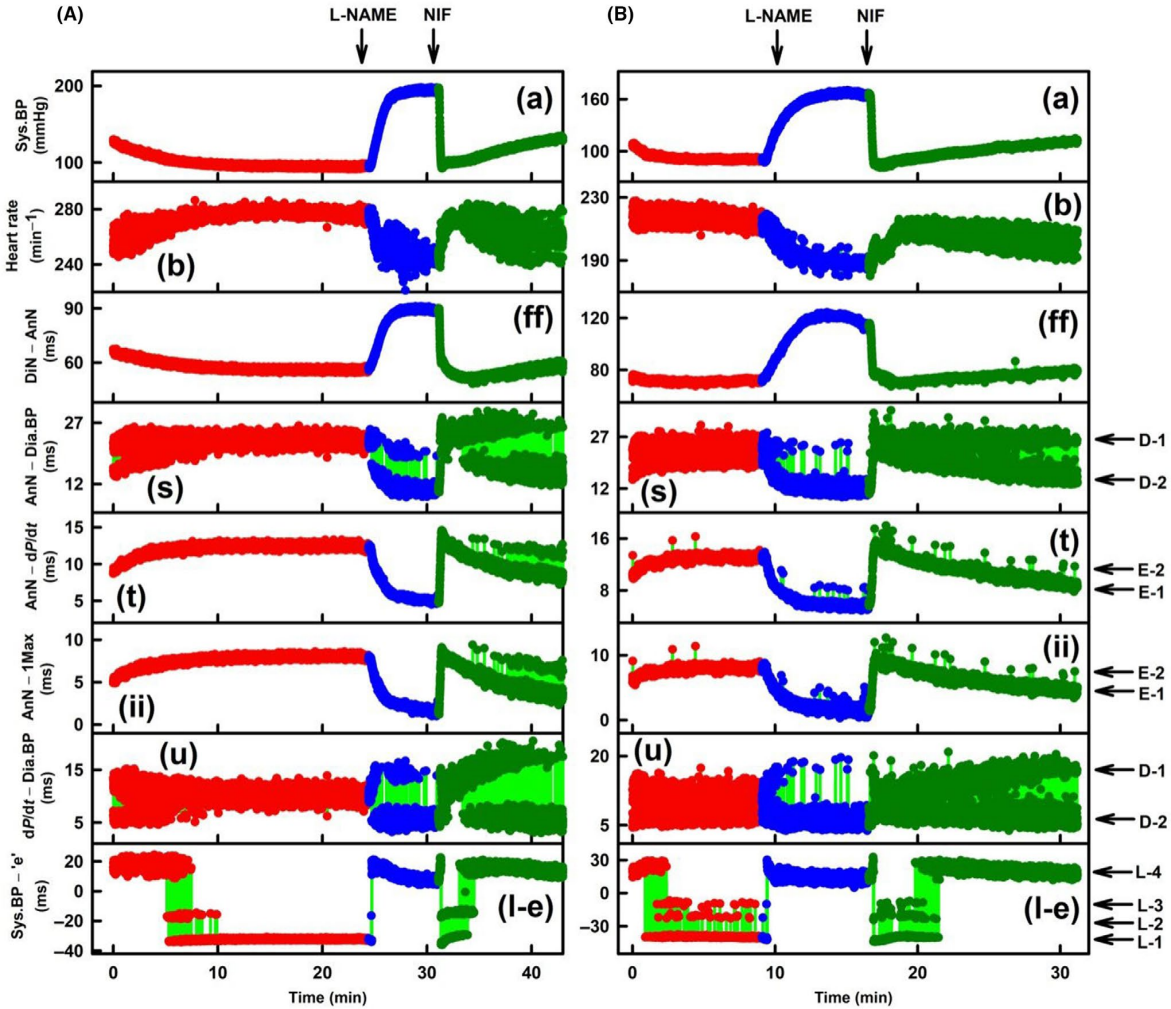
Time‐dependent changes in eight APW‐Ps at control and after administration of L‐NAME and NIF. (A) Exp‐1 and (B) Exp‐6. Time‐dependent changes in APW‐Ps, systolic BP (mmHg) (A), HR (min^−1^) (B), DiN–AnN (ms) (ff), AnN–Dia.BP (ms) (s), AnN–d*P*/d*t* (anacrotic notch–d*P*/d*t*_max_, ms) (t), AnN–1Max (ms) (II), d*P*/d*t*–Dia.BP (d*P*/d*t*_max_–diastolic BP, u), and Sys.BP–“e” (ms) (l‐e) of anesthetized rat: Control (red heartbeats), after intravenous administration of 15 mg/kg L‐NAME (blue heartbeats) and 400 nmol/kg NIF (dark green heartbeats). Red, blue, and dark green points are individual heartbeats. Horizontal arrows indicate predicted D‐1 and D‐2 levels, E‐1 and E‐2 levels, and L‐1 to L‐4 levels. The green lines show the connection between adjacent heartbeats. Definitions, units, and abbreviations of APW‐Ps evaluated from the APW are as explained in Figure S1. Zo/Xy anesthetized normotensive rats.

**FIGURE 2:**
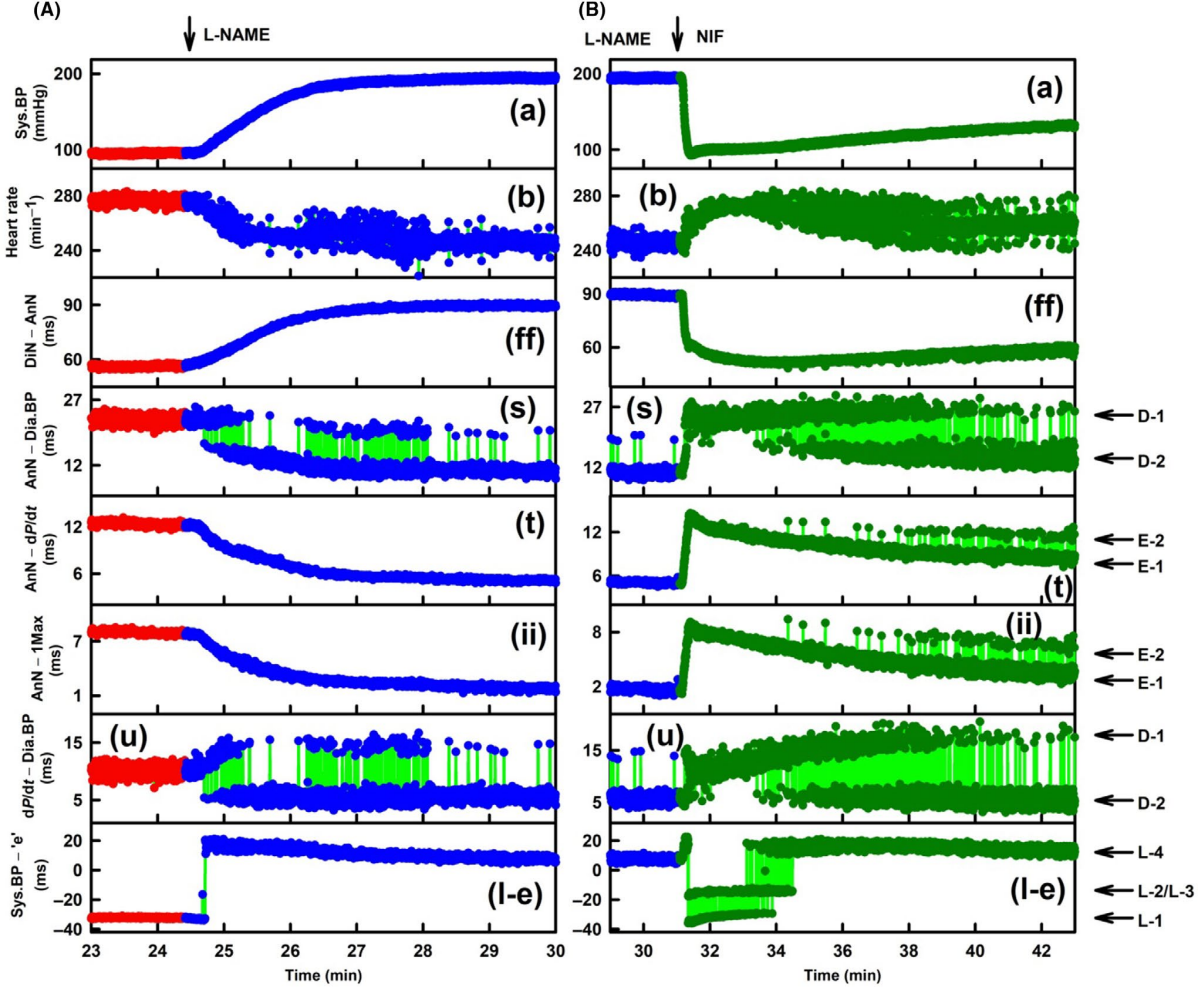
Exp‐1. Details of Figure 1, the time‐dependent changes in eight APW‐Ps at control and after the administration of L‐NAME and NIF. (A) Time‐dependent changes in APW‐Ps at control (red heartbeats) and after intravenous administration of 15 mg/kg L‐NAME (blue individual heartbeats). The green lines show the connection between adjacent heartbeats. (B) Time‐dependent changes in APW‐Ps of rat in the presence of 15 mg/kg L‐NAME (blue individual heartbeats) and after intravenous administration of 400 nmol/kg NIF (dark green heartbeats). The green lines show the connection between adjacent heartbeats. The legend details are the same as for Figure 1. Zo/Xy anesthetized normotensive rats.

**FIGURE 3:**
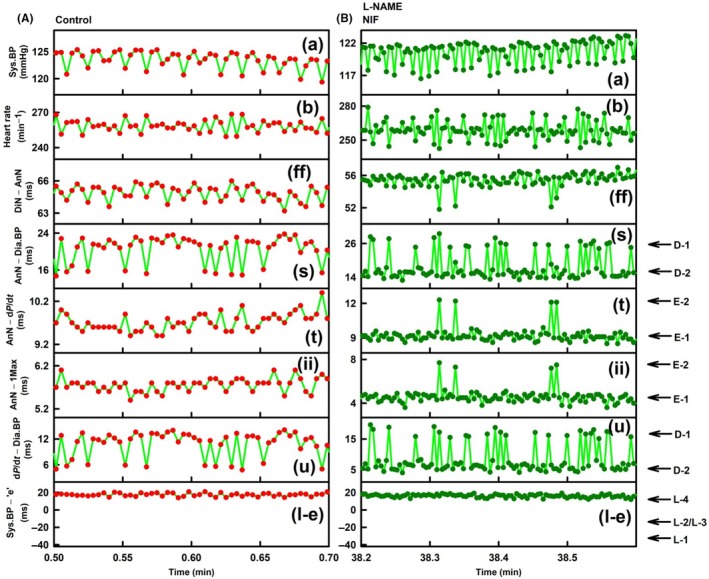
Exp‐1. Details of Figure 1, the time‐dependent changes in eight APW‐Ps at control and after the administration of L‐NAME and NIF. (A) Time‐dependent changes in APW‐Ps at control (red individual heartbeats). The green lines show the connection between adjacent heartbeats. (B) Time‐dependent changes in APW‐Ps of rat in the presence of 15 mg/kg L‐NAME and after intravenous administration of 400 nmol/kg NIF (dark green heartbeats). The green lines show the connection between adjacent heartbeats. The legend details are the same as for Figure 1. Zo/Xy anesthetized normotensive rats.

To describe results presented in Figures (1, 2, 3, 5, 6, 10, 12, and S6–S105), di‐gital (D1/D2 or E1/E2) and di‐gital, tri‐gital, and tetra‐gital (L‐1, L‐2, L‐3, and L‐4) parameters were defined. When the position of any parameter on APW, describing successive heartbeats, fluctuated regularly between two distinguished levels for more than 10 s, we defined it as di‐gital parameter. For example, Figures 1, 3, and S15, D1/D2, and E1/E2 show di‐gital behavior at parameters (s), (u), and (t), (ii). We defined the basic level as D‐1 (or E‐1), which is the level of control normotensive systolic BP (~100 mmHg). When the position of any parameter on APW, describing successive heartbeats, fluctuated regularly between more than two distinguished levels for at least 1 min, we defined the first level as L‐1, which is the level of control normotensive systolic BP (~100 mmHg). Levels L‐2 (di‐gital), L‐3 (tri‐gital), and L‐4 (tetra‐gital) represent regular fluctuations at higher systolic BP. For example, Figures 1A and 2B, parameter (l‐e) or Figure 12, parameter (w) show tri‐gital behavior, showing L‐1, L‐2, and L‐4 levels. Figures 1B, S11, parameter (l‐e), Figure 10, parameter (w) show tetra‐gital behavior, showing L‐1, L‐2, L‐3, and L‐4 levels.

### 2.5 Summary of experimental procedure after obtaining experimental data

The original experiments were designed to determine the effects of increased/decreased stiffness on APW parameters induced by L/NAME and NIF. Ten Xy/Zo anesthesized normotensive rats were used. Because we found that some parameters showed unusual behavior (they fluctuated regularly between several distinguished levels), we decided to study this phenomenon in detail. The parameters with the unusual behavior were selected and compared with systolic BP, HR, and/or augmentation index. Because it was not clear from the time difference of two points, fluctuated regularly between several distinguished levels on APW, which point is moving during consecutive heartbeats and which is not, or whether both points are moving, it was necessary to compare a combination of different APW parameters and use at least two reference points. From this comparison of several APW parameters, *n*‐gital parameters were suggested. To prove the suggested parameters, two reference points on APW were selected, point “e” and point “b” (d*P*/d*t*), which were used in different figures. Using both the reference points, qualitatively similar results were observed.

## 3 RESULTS

It should be noted that the aim of this work was not to investigate the effects of drugs on APW‐Ps, but to look for and characterize the time step of systolic pressure fluctuation on APW at different arterial stiffness. Two reference points on APW were selected to evaluate the *n*‐gital fluctuation of APW‐Ps. The first point is the APW parameter “e” (Figure S1). The second chosen reference point, d*P*/d*t*_max_ in mmHg ms^−1^, is the maximum derivative at point b; (P is BP in mmHg; Figure S1). To the best of our knowledge, we report data on di‐gital, tri‐gital, and tetra‐gital fluctuations of some APW‐Ps for the first time. Therefore, we present several original figures in the Figures S2–S105. Three animal rat models were used: intraperitoneal Zoletil/xylazine‐anesthetized normotensive rats, ISO‐anesthetized normotensive rats, and ISO‐anesthetized SHR rats.

### 3.1 The n‐gital fluctuation of APW‐Ps in 10 Zoletil/xylazine (Zo/Xy) anesthetized normotensive rats

It has been noticed that some APW‐Ps showed time step fluctuations on APW (Figures S1, S3, S5). Therefore, these APW‐Ps were selected and evaluated from 10 normotensive Zo/Xy anesthetized rats at control and after intravenous administration of L‐NAME, which decreased NO bioavailability and increased stiffness, followed by the administration of NIF to inhibit membrane calcium channels and decrease stiffness. These rat experiments were marked from Exp‐1 to Exp‐10. The results of selected APW‐Ps presented in Figures 1, 2, 3, 4, 5, 6 (except Figure 1B) were evaluated from Exp‐1. Results from nine similar experiments (nine rats) are presented in Figures S6–S80. In the time‐dependent Figures 1, 2, 3, 5, 6 and S6–S55, S59–S77, red, blue, and green circles show individual heartbeats at control, after the administration of L‐NAME and followed by administration of NIF, respectively.

Figure 1 shows a full record of control, intravenous administration of L‐NAME, and the subsequent NIF on eight APW‐Ps in Exp‐1 (Figure 1A) and Exp‐6 (Figure 1B). Some APW‐Ps showed time‐dependent gradual changes (plots [a], [ff]) or noise response [plot (b)], but other APW‐Ps oscillated between different levels and various cardiovascular conditions (marked by horizontal arrows). Parameters (s), (t), (ii), and (u) showed di‐gital responses, and parameter (l‐e) showed tri‐gital responses in Exp‐1 (Figure 1A) and tetra‐gital responses in Exp‐6 (Figure 1B).

Figure 2 shows more details of Figure 1A. After L‐NAME administration (Figure 2A), APW‐Ps (a), (ff), (t), and (ii) gradually changed, and APW‐P (b) was noisier. However, after systolic BP (a) was gradually increased, APW‐Ps (s) and (u) suddenly started to fluctuate di‐gitally from D‐1 to the new D‐2 level and back at individual heartbeats. As systolic BP increased (a), the probability of heartbeats (parameters [s] and [u]) occurring in the D‐1 position shifted to D‐2 position, and later, at high systolic BP, they were mostly in the D‐2 position. Values of time D‐1/D‐2 di‐gital fluctuation varied from ~5 to ~13 ms. Values of time E‐1/E‐2 di‐gital fluctuation were ~2.5 ms. During the increase in systolic BP, parameter (l‐e) jumped from L‐1 level to L‐4 level. Because states D‐1 and L‐1 (and state E‐1, Figure 2B) were observed at ~100 mmHg of systolic BP, it is suggested that these states are basic states of the CVS. The states D‐2 (E‐2) and L‐ >1, for the purpose of this work, are named activated states of CVS.

Figure 2B shows that in the presence of L‐NAME, when systolic BP was high, di‐gital values D‐2 and E‐2 and tri‐gital values L‐4 were mostly in the activated states. After subsequent administration of NIF, systolic BP (a) and parameter (ff) decreased rapidly (systolic BP to control ~100 mmHg [Figure S2]), and parameter (b) increased moderately and later fluctuated. However, after a rapid decrease in systolic BP (a) for the first 2 min, APW‐Ps (s), (t), (ii), and (u) showed di‐gital heartbeat fluctuation from activated D‐2 state (in the presence of L‐NAME) to basic D‐1 state and from activated E‐2 state to basic E‐1 state. During this time, parameter (l‐e) showed tri‐gital response from L‐4 through L‐2/L‐3 to basic L‐1 stage. Later after ~34 min during a gradual increase in systolic BP (a), the probability of di‐gital response to levels D‐2 and E‐2 again increased. Similarly, parameter (l‐e) increased from L‐1 through L‐2/3 to L‐4 level. According to changes in systolic BP (a), time‐dependent changes in parameter (l‐e), shown in Figure 1B, similarly fluctuated between tetra‐gital positions. Qualitatively similar results, as described in Figures 1 and 2, were observed in several other experiments (Figures S6–S54). The observation of the particular *n*‐gital fluctuation is summarized in Table 1.

**TABLE 1:** D‐1/D‐2 di‐gital and (l‐e) di‐gital, tri‐gital, and tetra‐gital observations in 10 rat experiments at control and after the administration of L‐NAME and subsequent administration of NIF.

Experiments			1	2	3	4	5	6	7	8	9	10
Control	D1/D2	Di‐gital	*						*	*	*	
Control	l‐e	Di‐gital		*				*	*		*	
Control	l‐e	Tri‐gital	*							*		*
Control	l‐e	Tetra‐gital						*		*		
L‐NAME	D1/D2	Di‐gital	*	*			*		*	*	*	*
L‐NAME	l‐e	Di‐gital		*								
L‐NAME	l‐e	Tri‐gital	*	*	*	*	*	*		*	*	*
L‐NAME	l‐e	Tetra‐gital						*	*			
NIF	D1/D2	Di‐gital	*	*	*		*	*	*	*	*	*
NIF	l‐e	Di‐gital		*		*						
NIF	l‐e	Tri‐gital	*	*		*	*	*	*	*	*	
NIF	l‐e	Tetra‐gital			*		*	*				

*Note*: Data are from Figures 1, 2, 3 and Figures S6–S54. Normotensive rats were anesthetized with Zoletil 100 (tiletamine + zolazepam, 80 mg/kg, i.p.) and xylazine (5 mg/kg, i.p.).

Figure 3A shows time‐dependent APW‐P details of Figure 1A at control. Systolic BP (a) periodically oscillated with low amplitude ~4 mmHg. Two APW‐Ps (s) and (u) showed di‐gital responses, whereas parameter (l‐e) was constant at L‐4 level. Di‐gital (D‐1 and D‐2) parameters (s) and (u) are at the same time at the same position indicating that these APW‐Ps reflect the same physiological event(s). Qualitatively similar results are shown in other four experiments (e.g., Figures S33, S34, S40, and S47) in which D‐1/D‐2 di‐gitally fluctuated.

Figure 3B shows details of the time‐dependent responses in the presence of L‐NAME and after the administration of NIF. Systolic BP (a) did not regularly oscillate with amplitude ~4 mmHg, similarly heart rate (HR) (b) ± 12 min^−1^ and parameter (ff) had a few perturbations. Regular decrease in systolic BP was during exhalation. Notably again, di‐gital fluctuation of parameters (s) and (u) (D‐1 and D‐2) had the same time position. Similarly, parameters (t) and (ii) (E‐1 and E‐2 positions) had the same time‐dependent responses. Similar results of D‐1/D‐2 fluctuation in the presence of L‐NAME or NIF were observed in other eight experiments (Figures S9, S10, S12, S16, S31, S36, S37, S42, S43, S44, S49, and S54), and the results of E‐1/E‐2 fluctuation in the presence of L‐NAME or NIF were observed in other eight experiments (Figures S10, S13, S23, S36, S37, S43, S44, S49, and S54). These results indicate that parameters (s) and (u) have the same physiological bases. Similarly, parameters (t) and (ii) have the same physiological bases. However, none of them are connected with the (l‐e) parameter, which was mostly at constant L level during the time record presented.

The similar time‐dependent response of parameters AnN–Dia.BP (s) and d*P*/d*t*_max_–Dia.BP (u) suggests that diastolic BP (Dia.BP) is time dependently di‐gitally fluctuating on APW. The similar time‐dependent response of parameters (t) and (ii) may suggest that anacrotic notch (AnN) is time dependently di‐gitally fluctuating on APW.

The relationships of APW‐Ps to systolic BP revealed the range of systolic pressure over which di‐gital, tri‐gital, and tetra‐gital changes occurred (Figure S55). Tri‐gital parameter (l‐e) was observed within 97 to 105 mmHg at control, within 98 to 106 mmHg after L‐NAME, and <94 to 106 mmHg after subsequent NIF. Parameter (l‐e) was in stage L‐1 at systolic BP ~ <95 mmHg and in stage L‐4 at ~>110 mmHg. In the presence of L‐NAME, di‐gital parameters (s) and (u) fluctuated within the range ~100 to ~200 mmHg of systolic BP. After the subsequent addition of NIF, di‐gital parameters (s), (t), (ii), and (u) fluctuated within the range ~100 to ~140 mmHg. Similar results, as presented in Figures 1, 2, 3, but quantitatively on a wider scale, were obtained within nine other experiments (Table S1).

**FIGURE 4:**
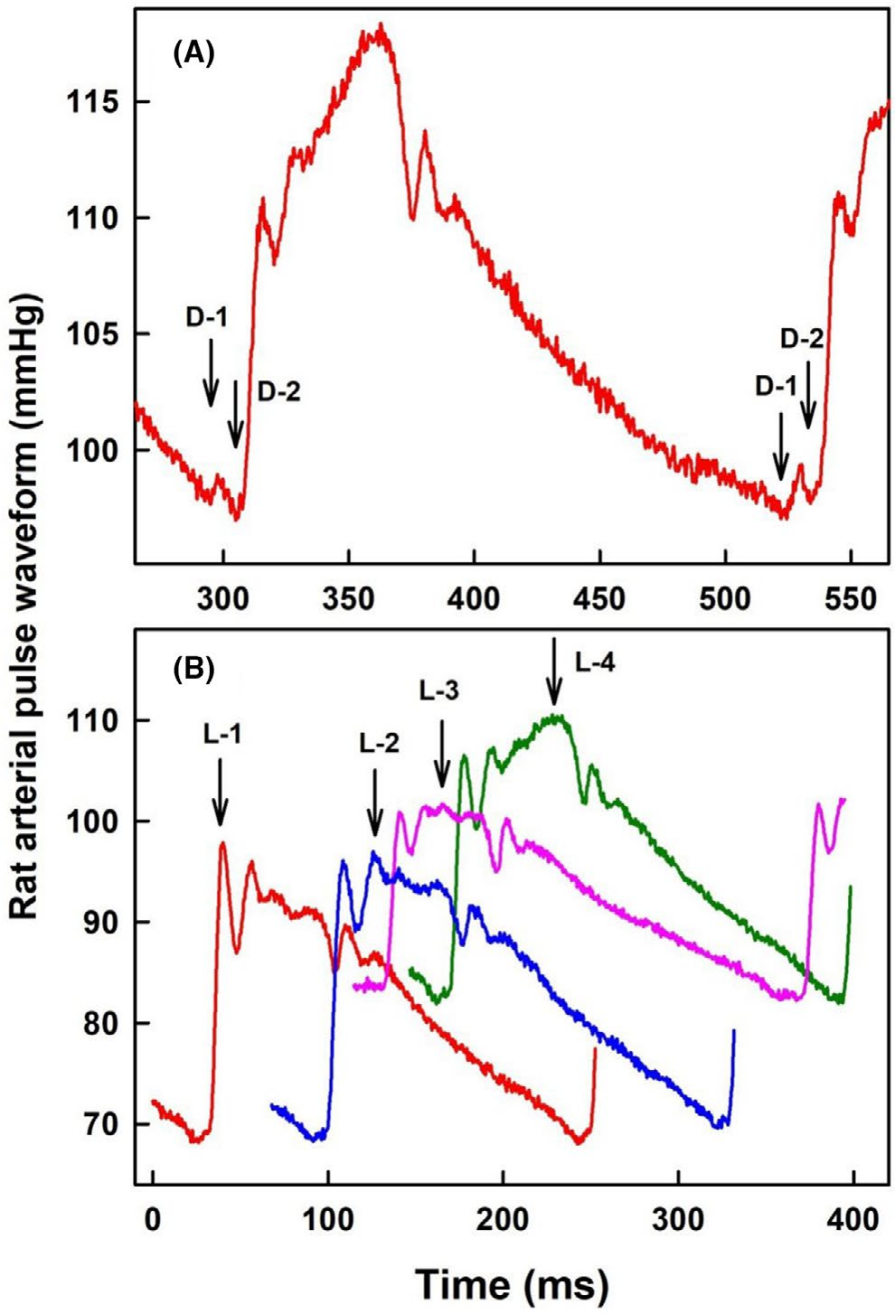
The examples of the D‐1/D‐2 time positions and L levels on the left common carotid APWs in the anesthetized rat. (A) Two time positions of diastolic BP of the neighboring APWs are marked as D‐1 and D‐2. (B) Time positions: L‐1 (red), L‐2 (blue), L‐3 (pink), and L‐4 (green) showed systolic BP (maximum on APW). Zo/Xy anesthetized normotensive rats.

The basis of the digital D‐1/D‐2 fluctuation as the time shift of the diastolic pressure position was confirmed by original records (Figure 4A). Original records of L‐1, L‐2, L‐3, and L‐4 positions confirmed that the basis of di‐gital, tri‐gital, and tetra‐gital fluctuations of parameter (l‐e) is the time position of systolic BP (Figure 4B). But the time positions of the E‐1/E‐2 di‐gital fluctuations were not visible in the original records. The time shift of anacrotic notch within ~2.5 ms was not detectable due to noise of no regular time (~2 ms) and BP (1 mmHg) fluctuation of systolic BP (Figure S56). Therefore, existence of E‐1/E‐2 di‐gital time fluctuation was not clear and was omitted in the next analysis.

To obtain more details of the time step fluctuation of parameter (l‐e), histogram of the parameter was evaluated in different CVS conditions. HR of 10 rat experiments without and with L‐NAME and NIF was in the range of 190–310 min^−1^. Therefore, to compare parameter (l‐e) in these different experiments, the (l‐e) parameter of 10 rats was normalized to 300 min^−1^ HR. It was originally hypothesized that reducing HR would proportionally increase the length between points on the APW in ms (Figure S1). However, it is not valid in our experiments. As it is seen from the cross‐relationships of 34 APW‐Ps to HR (Figure S4), plots DiN–AnN (ff, in ms) and dicrotic N. delay (mm, in ms) were very roughly linear with HR. Therefore, it should be considered that this normalization of (l‐e) values to 300 min^−1^ HR is only approximate.

Comparison of histograms (l‐e) of control with L‐NAME or NIF at three resolutions (Figure S57) showed three main peaks. Comparison of histograms in 10 experiments of control (Figure S57) and in the presence of L‐NAME (Figure S58A) showed three or four main peaks. Histograms after subsequent administration of NIF (Figure S58B) showed three or four main peaks and several small peaks supporting results of several distinct levels of the parameter (l‐e). No symmetrical distribution of the data on some peaks indicates further details of the parameter (Figures S57, S58, Table S2).

**FIGURE 5:**
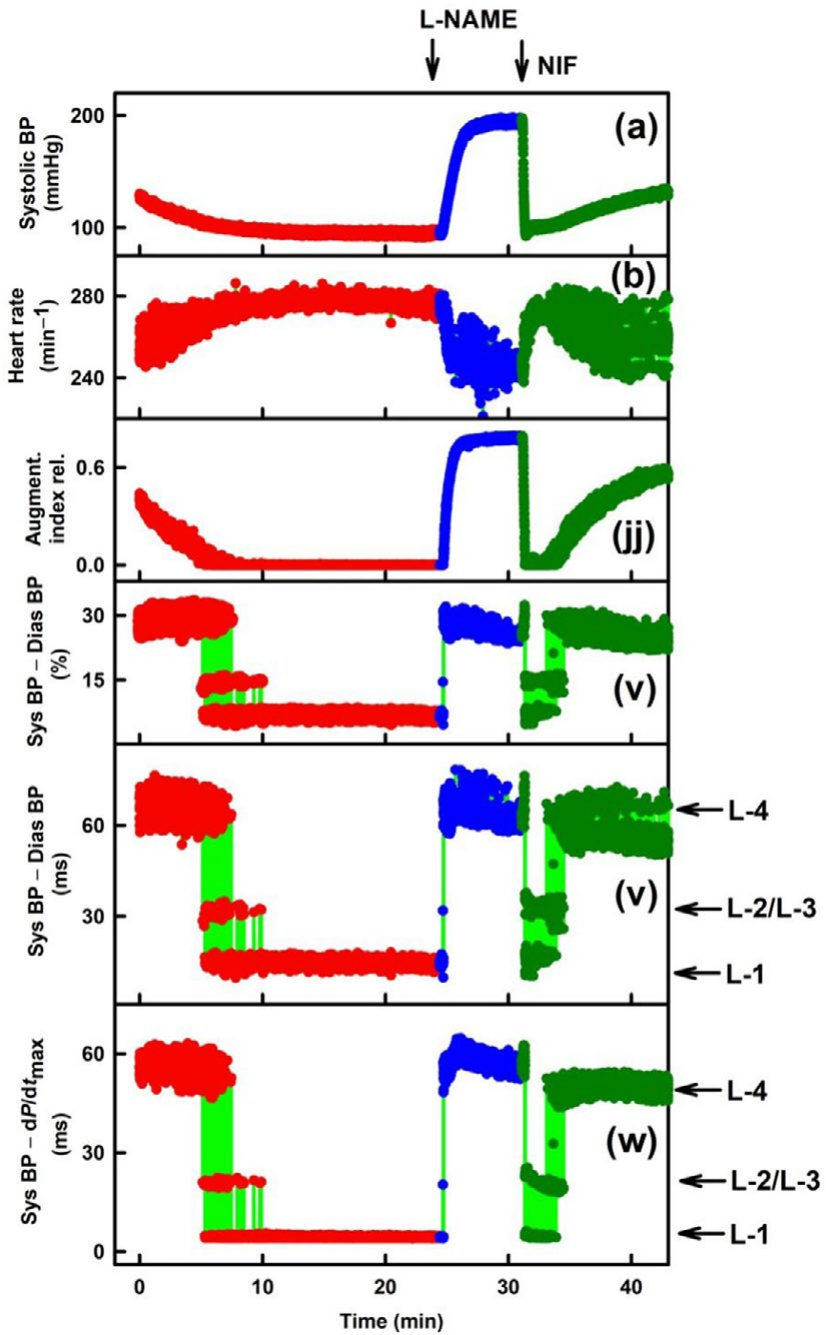
Exp‐1. Time‐dependent changes in six APW‐Ps at control and after the administration of L‐NAME and NIF. Systolic BP (mmHg) (A), HR (min^−1^) (B), augmentation index relative (jj), systolic BP–diastolic BP (v, %), systolic BP–diastolic BP (ms) (v) and systolic BP–d*P*/d*t*_max_ (ms) (w) at control (red), after the intravenous administration of 15 mg/kg L‐NAME (blue), and after the subsequent administration of 400 nmol/kg of NIF (dark green). Horizontal arrows indicate predicted L‐1 to L‐4 levels. The green lines show the connection between adjacent heartbeats. Definitions, units, and abbreviations of APW‐Ps evaluated from the APW are as explained in Figure S1. Zo/Xy anesthetized normotensive rats.

**FIGURE 6:**
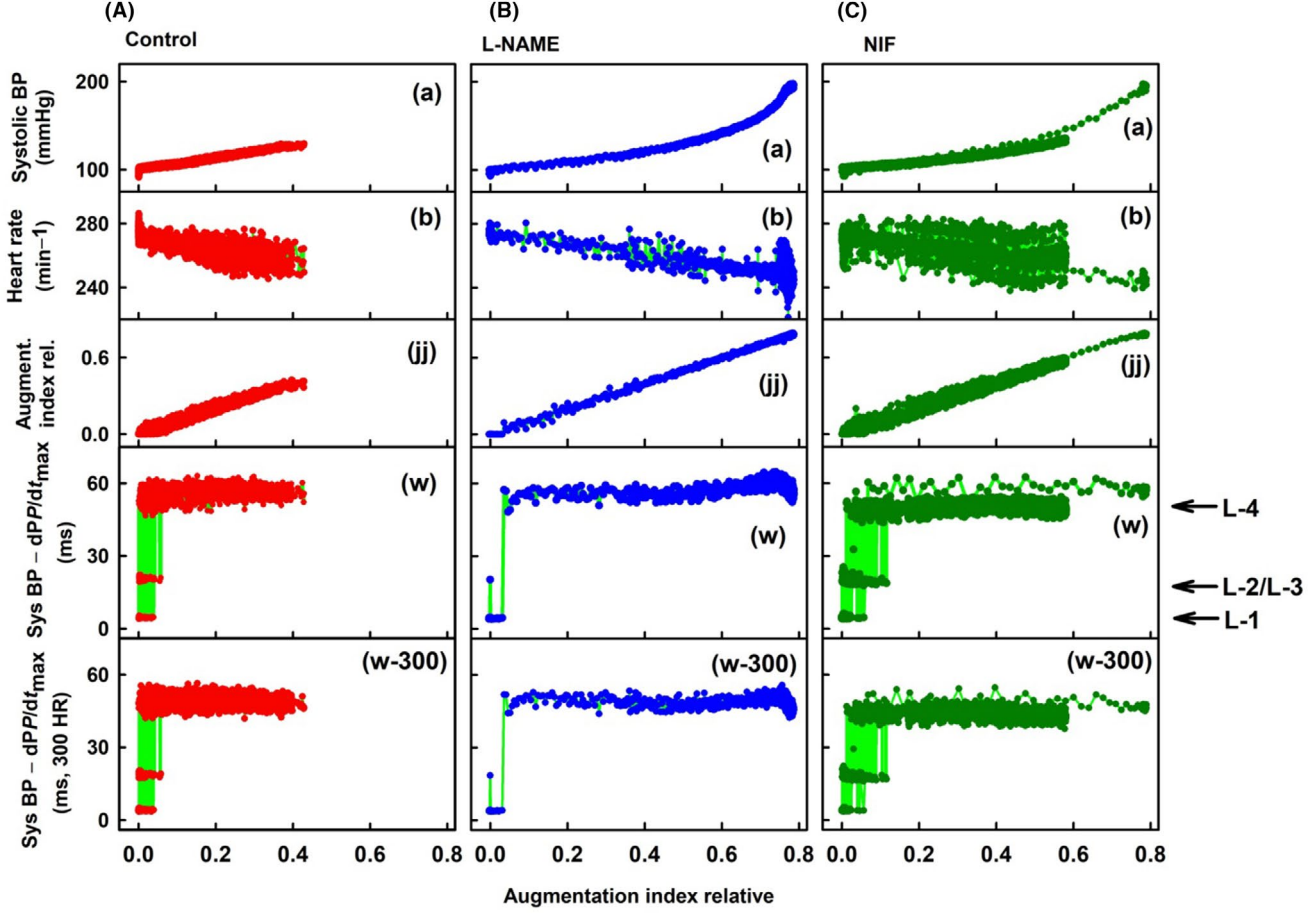
Exp‐1. Cross‐relationships of five APW‐Ps to augmentation index. (A) Control (red heartbeats) and (B) after the intravenous administration of 15 mg/kg L‐NAME (blue heartbeats) and (C) after the subsequent administration of 400 nmol/kg of NIF (dark green heartbeats). The green lines show the connection between adjacent heartbeats. Arrows indicate predicted L‐1 to L‐4 levels. Definitions, units, and abbreviations of APW‐Ps evaluated from the APW are as explained in Figure S1. Zo/Xy anesthetized normotensive rats.

**FIGURE 7:**
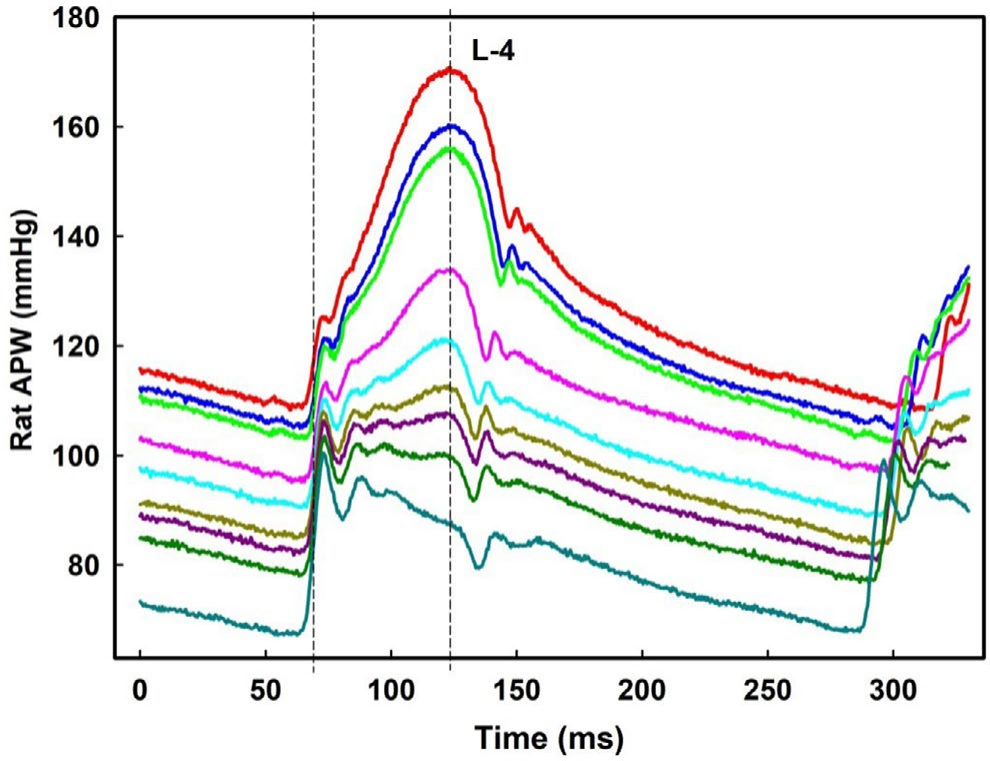
Exp‐3. Constant time position of systolic BP at L‐4 level of APW. Nine selected records of anesthetized rat APW after the intravenous administration of 15 mg/kg L‐NAME. L‐4 level is marked by the right dash line. Reference point is d*P*/d*t*_max_ values (marked by the left dash line). Zo/Xy anesthetized normotensive rats.

**FIGURE 8:**
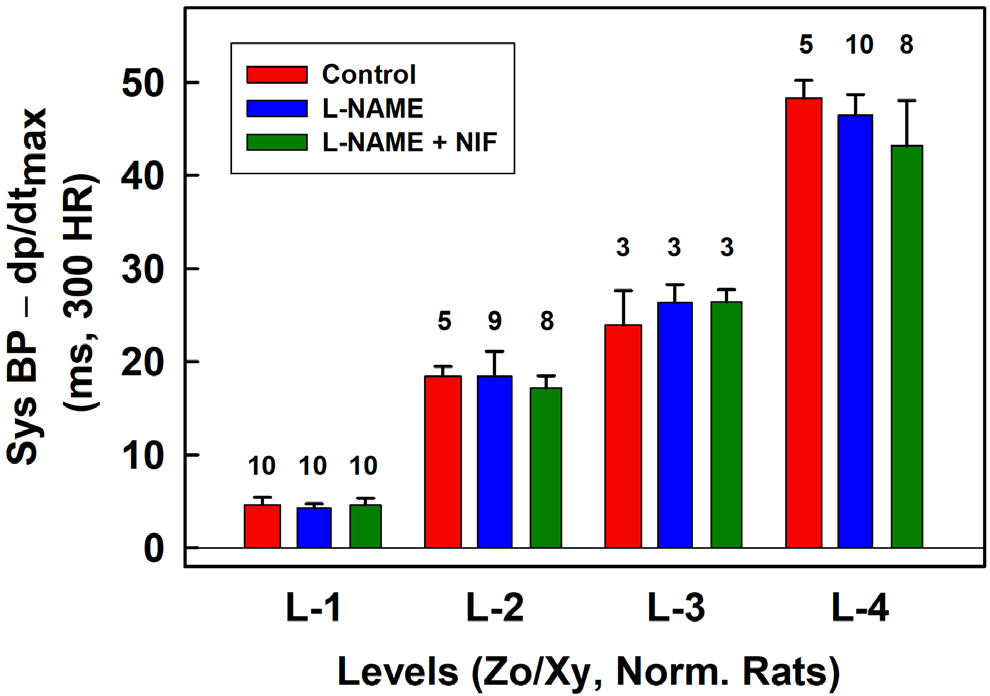
Time distances of systolic BP–d*P*/d*t*_max_ for L levels normalized to 300 min^−1^ heart rate (HR) in controls (red), in the presence of 15 mg/kg L‐NAME (blue), and after the subsequent administration of 200 or 400 nmol/kg of NIF (dark green). Data are from Exp‐1 (Figure 5) and from Figures S59–S68 after normalized to 300 min^−1^ HR. The numbers above the bars are the number of experiments. Means ± standard deviation (SD). Zo/Xy anesthetized normotensive rats.

To confirm the results obtained using “e” as the reference point, the reference point d*P*/d*t*_max_ (point “b,” Figure S1) was applied. Time distances between systolic BP and d*P*/d*t*_max_ (point “b”) without and with normalized distance to 300 min^−1^ HR are shown in Figure 5 (w) and 6 (w‐300). As shown in Figures 5, 6, S59–S80, qualitative results were similar as obtained when the reference point “e” was used. A comparison of the quality parameters of “w” and “w‐300” show that the *n*‐gital properties are similar. Constant time position of systolic BP at L‐4 level is confirmed also from original records of APW after the administration of L‐NAME in which systolic BP and augmentation index >0 significantly increased (Figure 7). Notably, the BP difference between the point of first maximum (point “c” in Figure S1) and diastolic pressure decreased with increasing systolic pressure. Statistical comparison of L levels, normalized to 300 min^−1^ HR, in controls, and after the subsequent administration of L‐NAME and NIF revealed that L‐1 values were similar despite large differences in stiffness induced by L‐NAME and NIF (Figure 8). The same applies to the values of L‐2, L‐3, and L‐4. The differences between the values of L‐1, L‐2, L‐3, and L4 are statistically significant.

**FIGURE 9:**
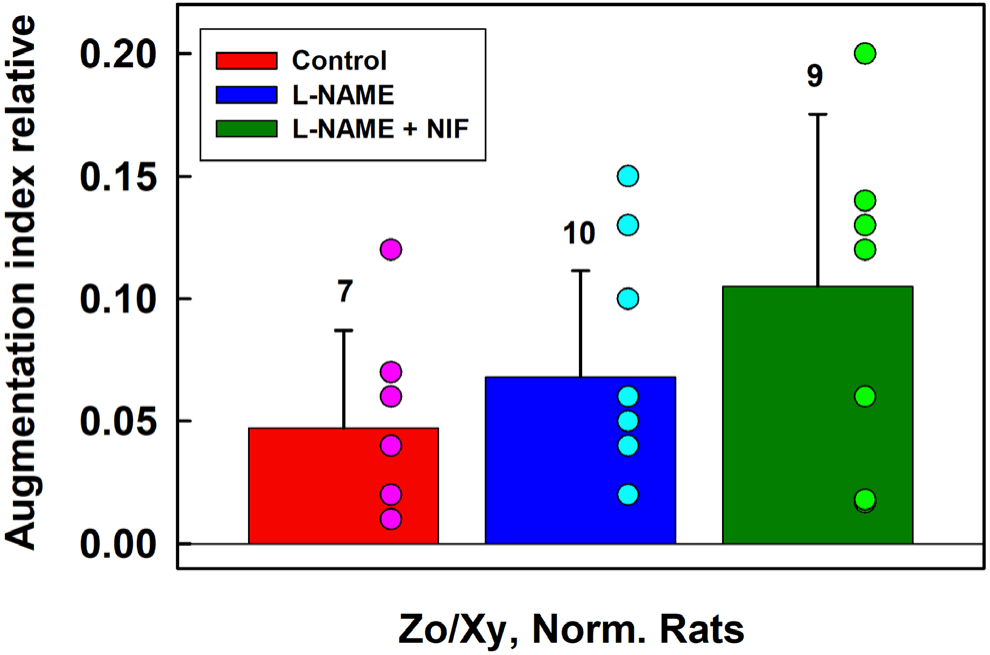
The minimal values of augmentation index at L‐4 level in controls (red), in the presence of 15 mg/kg L‐NAME (blue), and after subsequent administration of 200 or 400 nmol/kg of NIF (dark green). Data are from Exp‐1 (Figure 6) and from Figures S69–S77. The numbers above the bars are the number of experiments. Means ± standard deviation (SD). Pink, cyan, and green points show values in particular experiments. Zo/Xy anesthetized normotensive rats.

It was noticed that at high augmentation index, only L‐4 level of systolic BP was observed (Figures 6, S69–S77). Therefore, we evaluated minimal values of augmentation index at L‐4 level (red, blue, and dark green bars in Figure 9). Statistical comparison of the minimal values of augmentation index at L‐4 level in controls and after the subsequent administration of L‐NAME and NIF revealed large dispersion. This was in contrast to the L‐values (Figure 8). In all experiments, the position of systolic BP at augmentation index ~>0.2 was at L‐4 level.

### 3.2 The n‐gital fluctuation of APW‐Ps in ISO‐anesthetized normotensive and SHR rats

**FIGURE 10:**
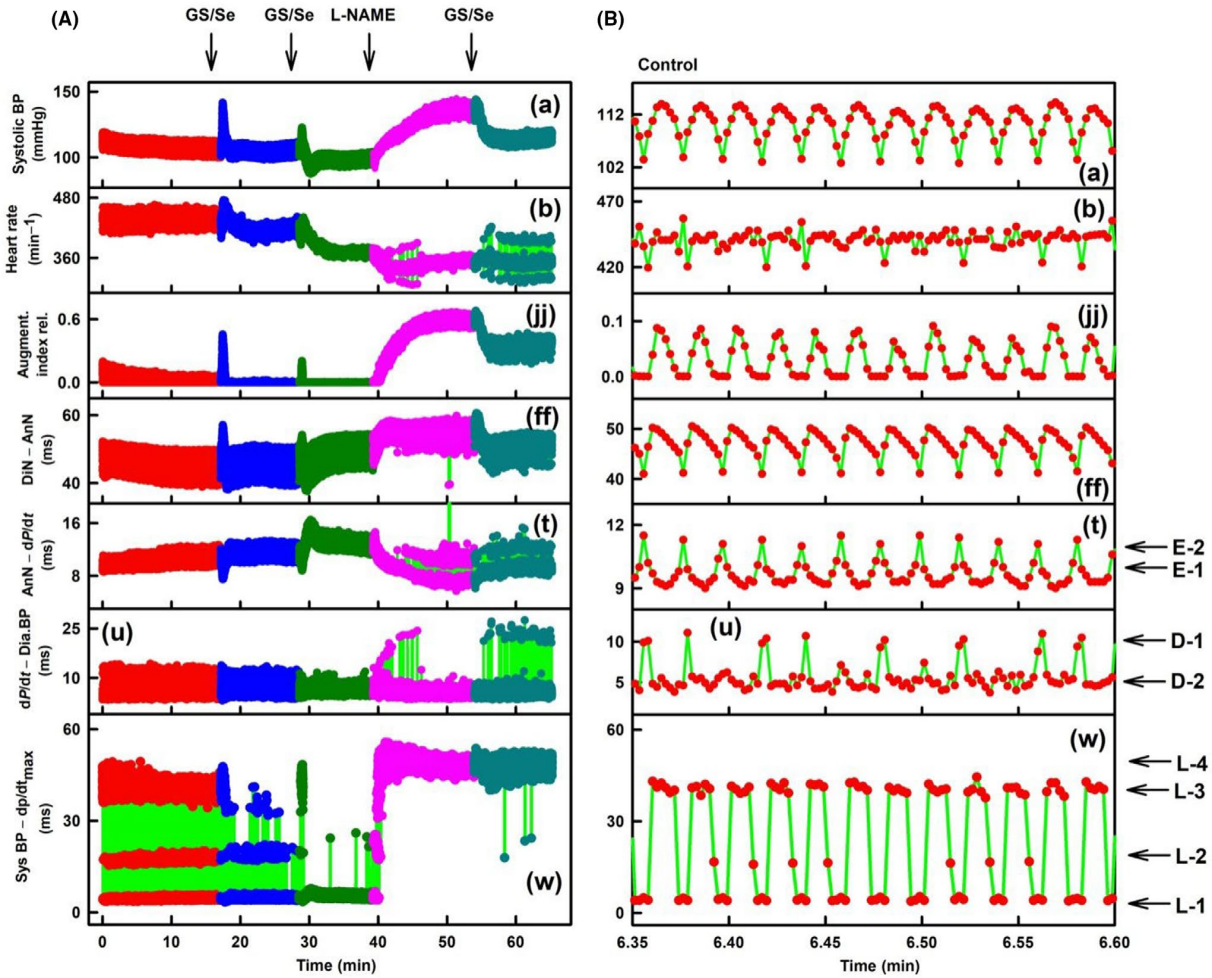
ExN‐1. ISO‐anesthetized normotensive rat. (A) Time‐dependent changes in seven APW‐Ps at control (red) and after the administration of twice glutathione/selenite (GS/Se, 75/12.5 in μmol L^−1^, blue, dark green), 30 mg/kg L‐NAME (pink), and GS/Se (dark cyan). APW‐Ps: Systolic blood pressure (BP) (mmHg) (a), HR (heart rate, min^−1^) (b), augment. index rel. (augmentation index relative [jj]), DiN–AnN (dicrotic notch–anacrotic notch, ms) (ff), AnN–d*P*/d*t*_max_ (anacrotic notch–d*P*/d*t*_max_, ms) (t), d*P*/d*t*–Dia BP (d*P*/d*t*_max_–diastolic BP [u]), and syst BP–d*P*/d*t*_max_ (systolic BP–d*P*/d*t*_max_, [w]). (B) Detailed time‐dependent APW‐Ps in control (red) of (A). Red, blue, dark green, pink, and dark cyan points are individual heartbeats. Horizontal arrows indicate predicted D‐1 and D‐2 levels, E‐1 and E‐2 levels, and L‐1 to L‐4 levels. The green lines show the connection between adjacent heartbeats. Definitions, units, and abbreviations of APW‐Ps evaluated from the APW are provided in Figure S1.

**FIGURE 11:**
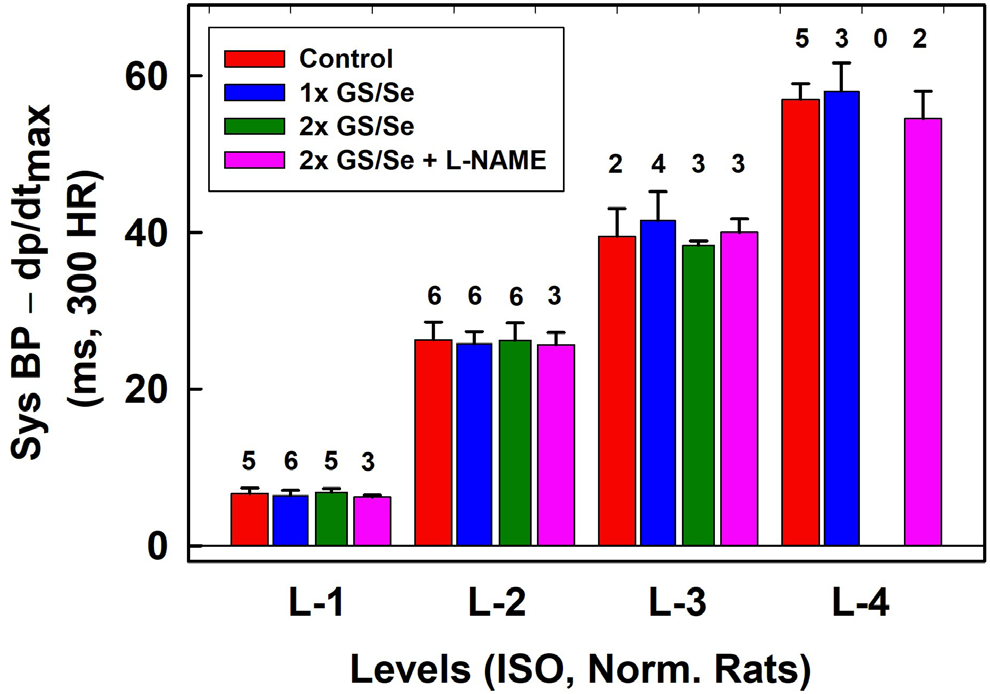
ISO‐anesthetized normotensive rats. Time distances of systolic BP–d*P*/d*t*_max_ for L levels, normalized to 300 min^−1^ HR, in controls (red) and after the administration of twice glutathione/selenite (GS/Se, 75/12.5 in μmol/L, blue, dark green) and 30 mg/kg L‐NAME (pink). Data are from ExN‐1 (Figure 10) and Figures S81–S92 after normalized to 300 min^−1^ HR. The numbers above the bars are the number of experiments. Means ± standard deviation (SD).

Because 35 APW‐Ps depended on the used anesthesia,^20^ we studied whether the *n*‐gital time fluctuation in rat APW‐Ps is observed in differently anesthetized normotensive and SHR rats and after modulated by different active compounds.^27^ In ISO‐anesthetized normotensive rats, in six experiments, marked as ExN‐1–ExN‐6, diastolic BP di‐gital (D1/D2) and systolic BP *n*‐gital fluctuations of APW‐Ps were observed (Figures 10, S81–S92). Some parameters oscillated based on the rat's breathing (Figure 10B). Similar to findings in normotensive rats anesthetized with Zo/Xy (Figure 8), statistical comparisons of L levels, normalized to 300 min^−1^ HR, in controls, and after subsequent administration of twice GS/Se and L‐NAME revealed that the values in a given L level were similar despite large differences in stiffness induced by GS/Se and L‐NAME (Figure 11). Similar to findings in normotensive rats anesthetized with Zo/Xy (Figure 9), a statistical comparison of the minimal values of augmentation index at L‐4 level in controls and after the subsequent administration of twice GS/Se and L‐NAME revealed a large dispersion (Figure S106). This was in contrast to the L values (Figure 11). In all experiments, the position of systolic BP at augmentation index ~>0.28 was at L‐4 level.

**FIGURE 12:**
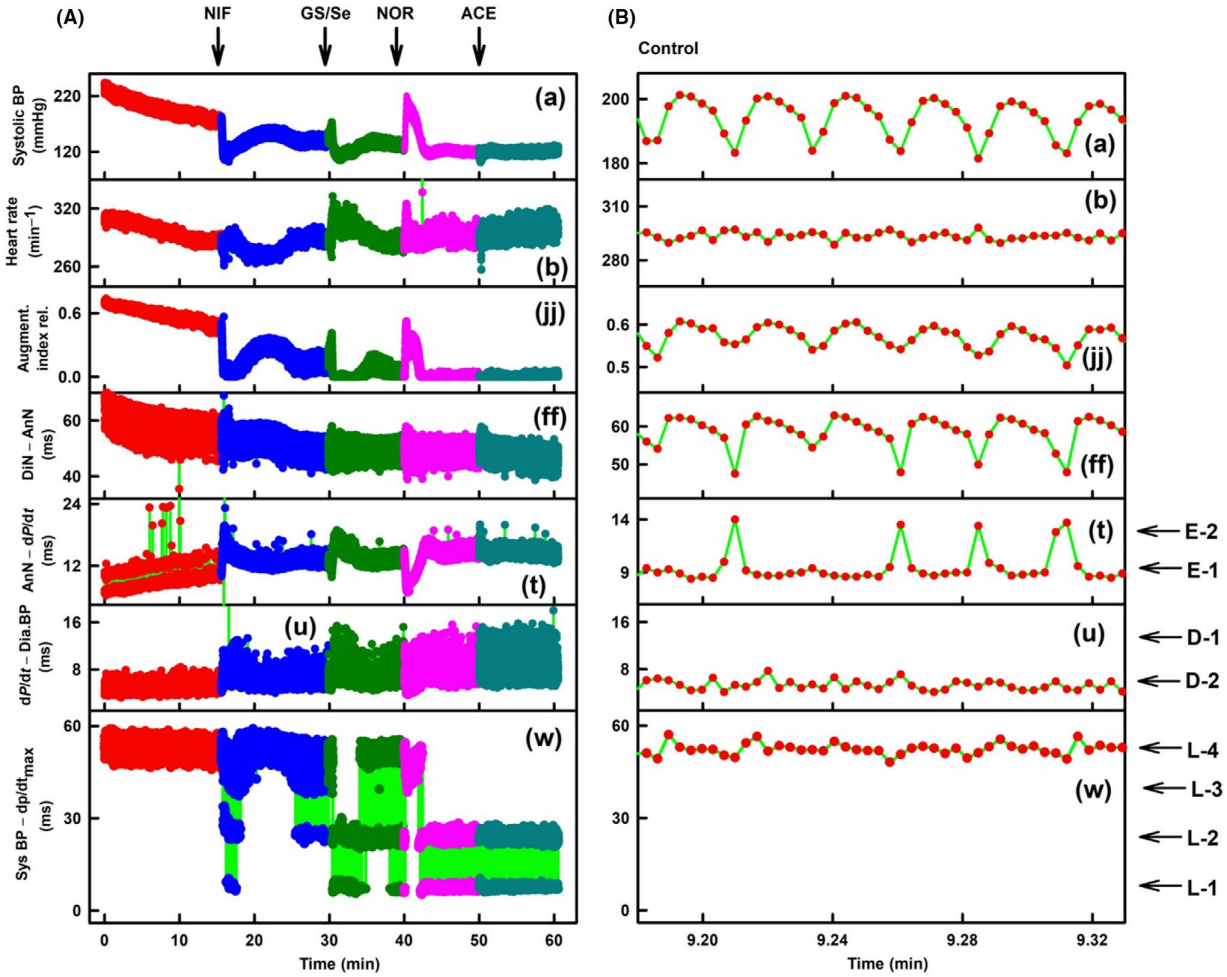
SHR‐1. ISO‐anesthetized spontaneously hypertensive (SHR) rats. (A) Time‐dependent changes in seven APW‐Ps at control (red) and after the administration of 400 nmol/kg NIF (blue), glutathione/selenite (GS/Se, 75/12.5 in μmol/L, dark green), 0.5 μg/kg NOR (pink), and 1 μg/kg ACE (dark cyan). APW‐Ps: Systolic BP (mmHg) (a), HR (heart rate, min^−1^) (b), augment. index rel. (augmentation index relative [jj]), DiN‐AnN (dicrotic notch–anacrotic notch, ms) (ff), AnN–d*P*/d*t*_max_ (anacrotic notch–d*P*/d*t*_max_, ms) (t), d*P*/d*t*–Dia BP (d*P*/d*t*_max_–diastolic BP [u]), and syst BP–d*P*/d*t*_max_ (systolic BP–d*P*/d*t*_max_, [w]). (B) Detailed time‐dependent APW‐Ps in control (red) of (A). Red, blue, dark green, pink, and dark cyan points are individual heartbeats. Horizontal arrows indicate predicted D‐1 and D‐2 levels, E‐1 and E‐2 levels, and L‐1 to L‐4 levels. The green lines show the connection between adjacent heartbeats. Definitions, units, and abbreviations of APW‐Ps evaluated from the APW are provided in Figure S1.

**FIGURE 13:**
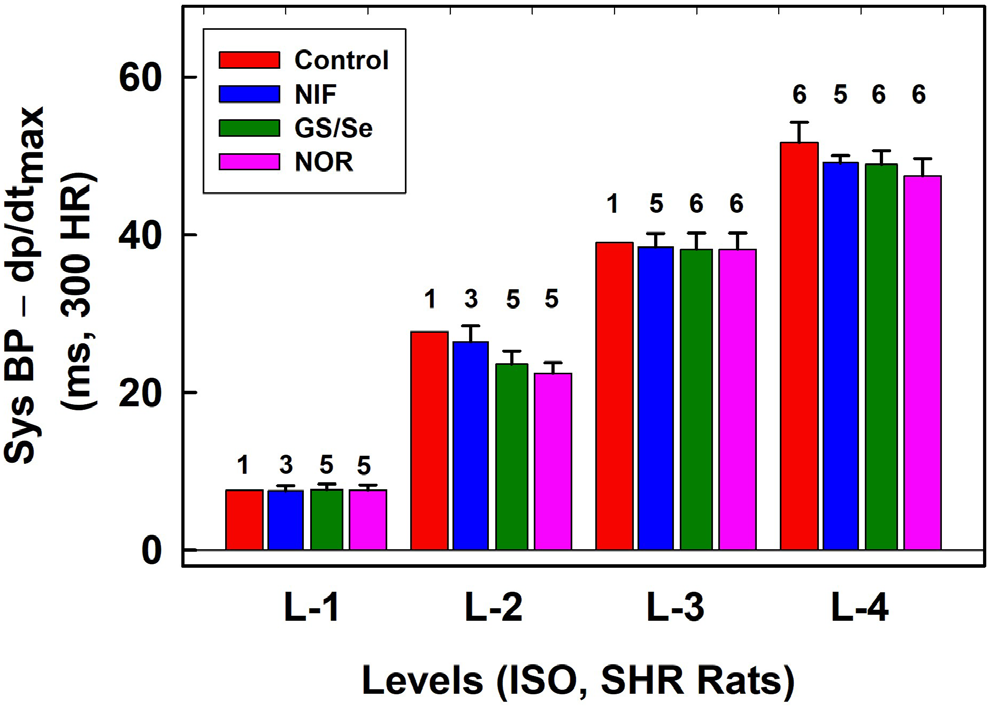
ISO‐anesthetized SHR rats. Time distances of systolic BP–d*P*/d*t*_max_ for L levels normalized to 300 min^−1^ HR in controls (red) and after the administration of 200 and 400 nmol/kg NIF (blue), glutathione/selenite (GS/Se, 75/12.5 in μmol/L, dark green), and 0.5 μg/kg NOR (pink). Data are from SHR‐1 (Figure 12) and from Figures S94–105 after normalized to 300 min^−1^ HR. The numbers above the bars are the number of experiments. Means ± standard deviation (SD).

**FIGURE 14:**
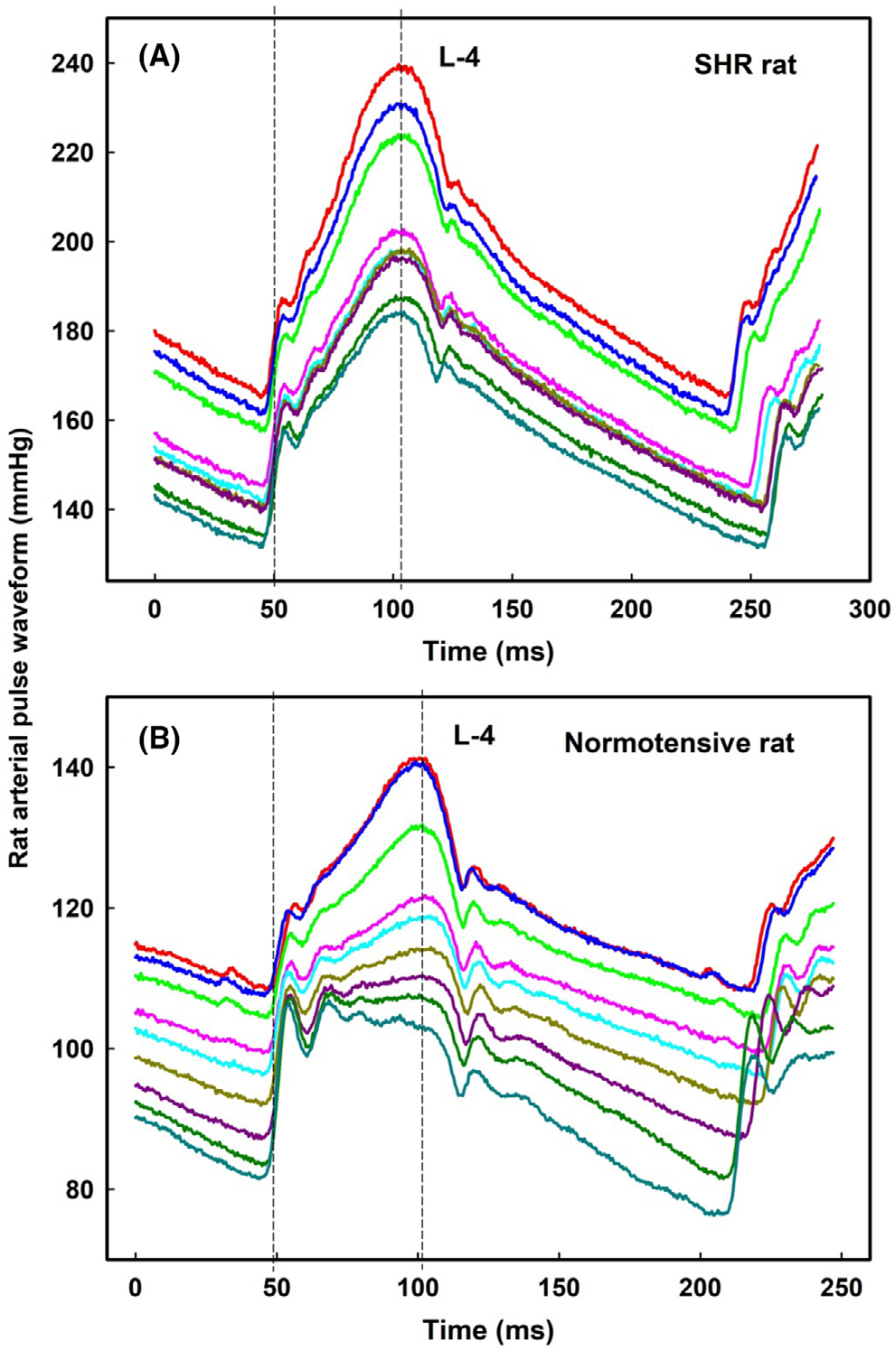
Constant time position of systolic BP at L‐4 level of APW. (A) Nine selected records of ISO‐anesthetized SHR rat APW in control (Figure 9; SHR‐1). (B) Nine selected records of ISO‐anesthetized normotensive rat APW after intravenous administration of 30 mg/kg L‐NAME (Figure 8; ExN‐1). L‐4 level is marked by the right dash line. Reference point is d*P*/d*t*_max_ values (marked by the left dash line).

In ISO‐anesthetized six SHR rats, marked as SHR‐1 to SHR‐6, *n*‐gital fluctuation of “w” parameter was also observed (Figures 12, S94–S105). Some APW‐Ps oscillated based on the rat's breathing and decreased when the rat exhaled (Figure 12B). The statistical comparisons of L levels and augmentation index presented in Figures 12, 13, S107 are qualitatively similar to those found for normotensive rats anesthetized with Zo/Xy or urane. All conclusions for SHR rats (Figures 12, 13, S107) are the same as presented for normotensive rats anesthetized with Zo/Xy. Constant time position of systolic BP at L‐4 level is confirmed also from original records of SHR rat APW (Figure 14A) and normotensive rat APW after the administration of L‐NAME (Figure 14B).

HR of normotensive and SHR rats was different and depended on the administered compound and anesthesia. Therefore, for the statistical comparison (Figures 8, 11, 13), the L‐data were normalized to 300 min^−1^ HR. However, as it was mentioned in Section 3.1, the increase/decrease in HR did not decrease/increase the length between points on the APW in ms proportionaly (Figure S1). Therefore, it should be considered that this normalization of L‐values to 300 min^−1^ HR is only approximate. Therefore, we did not statistically compared L level for normotensive and SHR rats under different anesthesia. As for D1 and D2, their values varied in experiments with normotensive and SHR rats and with administration of different substances, statistical comparisons of these parameters were not performed.

## 4 DISCUSSION

To the previously described 35 APW‐Ps,^7^ new 22 APW‐Ps were introduced in the present work. From these APW‐Ps, it was observed that some parameters, which measure time position of diastolic BP on APW, showed di‐gital time fluctuation, and the parameters that measured systolic BP time position on APW showed di‐gital, tri‐gital, and tetra‐gital time fluctuation. Values of the diastolic BP di‐gital time fluctuation were observed mostly in Zo/Xy normotensive rats, less in ISO‐anesthetized normotensive rats, and very rarely in ISO‐anesthetized SHR rats. It is assumed that the occurrence of the di‐gital time fluctuation of diastolic BP depends on specific conditions of CVS. In our previous studies, using normotensive Zo/Xy anesthetized rats, distinct (di‐gital) fluctuation of diastolic BP has been observed from the fluctuation of anacrotic notch delay (dd, Figure S3) after S‐nitrosoglutathione and H_2_S administration that decreased/increased systolic BP, respectively.^25^, ^26^ These studies, along with the present work, suggest that the time di‐gital fluctuation of diastolic BP in anesthetized rats exists during specific cardiovascular conditions. The physiological basis of the di‐gital fluctuation is unknown; it may reflect two distinct physiological events at the beginning of cardiac contraction. Clarifying this is a challenge for further studies.

Inhibition of NO synthesis by L‐NAME decreases NO bioavailability and increases systolic BP and arterial stiffness.^21^, ^22^, ^23^ Therefore, we used L‐NAME to measure time position of systolic BP on APW at different stiffness. The temporal position of systolic BP on artery waveform is assumed to gradually shift on the APW as a function of the increase/decrease stiffness.^3^, ^4^, ^5^, ^6^, ^11^, ^15^ However, in our APW study in anesthetized rats, the time position of systolic BP had one, two, three, or four constant time values. Notably, even di‐gital, tri‐gital, and tetra‐gital time fluctuating positions of systolic BP were approximately constant within the significant changes of systolic BP and augmentation index induced by L‐NAME or using SHR rats. The observation that the BP difference between the points of the first maximum (point “c” in Figure S1) and diastolic pressure decreased with increasing systolic pressure (Figure 7) may not be in support of the prediction that at high aortic stiffness, the reflected wave arrives early during systole and contributes to the increase in systolic pressure in case of rat APW. Thus, our results may challenge the assumption of the gradual changes in the time position of systolic BP at APW of anesthetized rats. We can assume that the reflection waves contribute less significantly to the shape of the rat APW after increasing the stiffness.

Because the relationships between rat breathing and HR are known,^28^ it is supposed that the oscillations of systolic BP and other APW‐Ps were a consequence of the anesthetized rat breathing, which was significantly pronounced in rats under ISO anesthesia. Due to nonregular time and BP fluctuation of systolic BP (~2 ms, 1 mmHg), it was not possible to confirm the time di‐gital fluctuation of anacrotic notch (E di‐gital values) from original APW records. Therefore, existence of the di‐gital time fluctuation of anacrotic notch is not clear at present.

To our knowledge, there have been no reports on the constant time positions of systolic pressure on the APW in spite of high difference in BP and vascular stiffness. Also, fluctuations in individual heartbeats between several L‐levels have not been published. At this stage of research, the physiological origin of the constant L levels is unknown. We may only speculate that the constant systolic pressure time position measured at the left carotid artery at different values of systolic BP and vascular stiffness may result from regulated volume of arterial tree that might be advantageous for blood circulation at different BP. Because the left common carotid artery was cannulated for the insertion of pressure transducers, blood flow through it was stopped. Therefore, we cannot exclude that this experimental approach may also contribute to the observation of *n*‐gital fluctuations.

Because high BP and related augmentation index or arterial stiffness are indicators of many negative phenomena in CVS, understanding the constant time positions of systolic BP may contribute to understanding of CVS in pathological conditions. To find out the physiological base of the step time fluctuation in diastolic and systolic BP on APW, and the question whether it is present also in other animals and in humans, remains a challenge for future studies.

At the current stage of the first knowledge about *n*‐gital fluctuation of diastolic and systolic time positions on APW, it is first necessary to understand the basics of *n*‐gital fluctuation. Without it, it is difficult to predict the physiological or pathological significance of “constant time positions” of systolic pressure. Therefore, understanding the nature of *n*‐gital fluctuation is a challenge for further studies.

## 5 CONCLUSIONS

The temporal position of systolic BP on the aortic pulse curve is thought to shift gradually on the curve in response to increasing/decreasing vascular stiffness.

Rat models were used to characterize the temporal movement of diastolic and systolic BP on the APW of anesthetized rats during increasing/decreasing vascular stiffness. During increases/decreases in stiffness, the temporal position of diastolic pressure of individual heartbeats on the APW shifted di‐gitally between two temporal positions, and the temporal position of systolic pressure on the APW did not shift gradually during increases/decreases in vascular stiffness, as expected, but oscillated between constant di‐gital, tri‐gital, or tetra‐gital temporal positions. The results may challenge the assumption of a gradual temporal positioning of systolic pressure at the rat APW under these conditions.

## AUTHOR CONTRIBUTIONS

**Anton Misak:** Data curation; formal analysis; investigation; methodology; validation. **Lenka Tomasova:** Data curation; methodology. **Marian Grman:** Formal analysis. **Karol Ondrias:** Conceptualization; formal analysis; investigation; methodology; supervision; writing – original draft.

## FUNDING INFORMATION

This work was supported by funding from ERA4Health: InterHeart 2025 to L. Tomasova and the VEGA Grant Agency of the Slovak Republic (grant number 2/0138/25 to A. Misak and 2/0066/23 to L. Tomasova).

## CONFLICT OF INTEREST STATEMENT

The authors declare no conflicts of interest.

## ETHICS STATEMENT

The procedures were approved by the State Veterinary and Food Administration of the Slovak Republic (C.k. Ro 3123/17‐221) according to the guidelines from Directive 2010/63/EU of the European Parliament. The procurement of animals, the husbandry, and the experiments conformed to the “European Convention for the Protection of Vertebrate Animals used for Experimental and other Scientific Purposes” (Council of Europe No 123, Strasbourg 1985). Experiments were carried out according to the guidelines established by the Animal Welfare Committee of the Biomedical Research Center, Slovak Academy of Sciences, Bratislava. All animal experiments were complied with the ARRIVE guidelines.

## Supporting information


Figure S1.

## Data Availability

Most of the data are presented in the main text and in the Supporting Information. All original records are available from K. Ondrias.
