# Supplementary material for: Characterization of di‐gital, tri‐gital, and tetra‐gital temporal movement of systolic blood pressure on the arterial pulse waveform of rats at different vascular stiffness
Source: Animal Model Exp Med. 2025 Dec 11;8(12):2266–79. doi: 10.1002/ame2.70108 (PMC12884430; doi:10.1002/ame2.70108)
Supplement: Supplementary file 1 — Figure S1. [file AME2-8-2266-s001.doc]

**Supplementary Information**

**to**

**Characterization of di-gital, tri-gital and tetra-gital temporal movement of systolic blood pressure on the arterial pulse waveform of rats under different vascular stiffnes**

**Anton Misak, Lenka Tomasova, Marian Grman, Karol Ondrias**

Institute of Clinical and Translational Research, Biomedical Research Center, Slovak Academy of Sciences, Dubravska Cesta 9, 845 05 Bratislava, Slovak Republic.

Correspodence to: Karol Ondrias, [karol.ondrias@savba.sk](mailto:karol.ondrias@savba.sk); Tel: +421-908577943

**Definition of arterial pulse waveform parameters (APW-Ps) from rat arterial pulse waveform (Kurakova et al. 2020; Tomasova et al. 2021)**


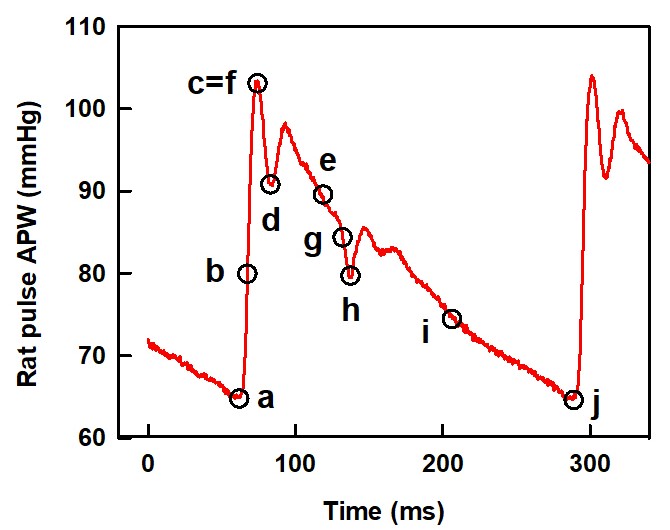


FIGURE S1 The left common carotid artery pulse waveform (APW) in the anesthetized rat with marked ten points **a** - **j** (black circles).

The APW parameter ‘**e**’ (BP and time position) is on the APW line in the middle between the APW parameter ‘**c**’ (BP and the time position of the first maximum) and the APW parameter ‘**h**’ (BP and time position of dicrotic notch).

Ten points **a - j** (in bold letters) are from Fig. S1 and they mark the values of BP and time that are used to define (calculate) specific APW-Ps^1,2^.

(a) Systolic blood pressure in mmHg; point **c** or **f**.

(b) Heart rate in min^–1^; 60 / (**j**– **a**); (**j**– **a**) represents time interval between **a** and **j**, **a** and **j** are two reference points to diastolic BP value.

(c) Systolic area in mmHg s; integral BP of **a** to **h**; **h** refers to BP at the dicrotic notch (dicrotic BP).

(d) dP/dt_max_ in mmHg ms^–1^; maximum derivative at the point **b**; P is BP in mmHg.

(e) dP/dt_max_ relative level; relative level (RL) of point **b**; (**b**– **a**) / (**c** (or **f**) – **a**) in mmHg/mmHg (dimensionless).

(f) dP/dt_d_ in mmHg ms^–1^; negative derivative at the point **i**; the point **i** is the BP in the middle of the time interval between **h** and **j**.

(g) dP/dt_d_ relative level, relative level of point **i**; (**i**– **a**) / (**c** (or **f**) – **a**) in mmHg/mmHg (dimensionless).

(h) dP/dt_d_ – dP/dt_max_ in s; time interval between **b** and **i**, dP/dt_d_ – dP/dt_max_ = (**i**– **b**).

(i) dP/dt_d_ – dP/dt_min_ in s; time interval between **g** and **i**, dP/dt_d_ – dP/dt_min_ = (**i**– **g**); dP/dt_min_ is maximum negative derivative at the point **g**.

(j) Diastolic blood pressure in mmHg; the point **a** or **j.**

(k) Pulse BP in mmHg; (**c**– **a**) or (**f**– **a**).

(l) Diastolic area in mmHg s; integral BP of **h** to **j**.

(m) dP/dt_min_ in mmHg ms^–1^; dP/dt_min_ is maximum negative derivative at the point **g**.

(n) dP/dt_min_ relative level, relative level of point **g**; (**g**– **a**) / (**c** (or **f**) – **a**) in mmHg/mmHg (dimensionless).

(o) dP/dt_min_ delay in s; delay in s of point **g**; (**g**– **a**) time interval between **a** and **g**.

(p) dP/dt_d_ delay in s; delay in s of point **i**; (**i**– **a**) time interval between **a** and **i**.

(q) dP/dt_d_ – dP/dt_max_ in mmHg; (**i**– **b**) BP difference between **b** and **i**.

(r) dP/dt_d_ – dP/dt_min_ in mmHg; (**i**– **g**) BP difference between **g** and **i**.

(s) [AnN in ms (point **d**)] – [Diast. BP in ms (point **a**)] in ms; (**d**– **a**) time interval between **d** and **a**.

(t) [AnN in ms (point **d**)] – [dP/dt in ms (point **b**)] in ms; (**d**– **b**) time interval between **d** and **b**.

(u) [dP/dt_max_, position in ms (point **b**)] – [Diast. BP in ms (point **a**)] in ms; (**b**– **a**) time interval between **b** and **a**.

(v) [Systolic blood pressure in ms (point **c** or **f)] – [**Diastolic blood pressure in ms (point **a)].**

(w) [Systolic blood pressure in ms (point **c** or **f)] –** [dP/dt_max_, position in ms (point **b**)].

(bb) Anacrotic notch in mmHg; BP at the point **d**.

(cc) Anacrotic notch relative level; relative level of point **d**; (**d**– **a**) / (**c** (or **f**) – **a**) in mmHg/mmHg (dimensionless).

(dd) Anacrotic notch delay in ms; delay in ms of point **d**; (**d**– **a**) time interval between **a** and **d**.

(ee) Anacrotic notch relative delay; relative delay (RD) of point **d**; (**d**– **a**) / (**j**– **a**) in ms/ms (dimensionless).

(ff) [Dicrotic notch (DiN) in s] – [Anacrotic notch (AnN) in s] in s; (**h**– **d**) time interval between **d** and **h**.

(gg) [(DiN – AnN) in s] / [dP/dt_min_ in mmHg µs^-1^] in s/mmHg µs^–1^; (**h**– **d**) / **g**.

(hh) [(DiN – AnN) in s] / [dP/dt_max_ in mmHg µs^-1^] in s/mmHg µs^–1^; (**h**– **d**) / **b**.

(ii) [AnN in ms] – [1Max (point **c** or the 1st. maximum) in ms] in ms; (**d**– **c**) time interval between **c** and **d**.

(jj) Augmentation index relative; (**f**– **c**) / (**f**– **a**) in mmHg/mmHg (dimensionless)**.

(kk) Dicrotic notch in mmHg; BP at the point **h**.

(ll) Dicrotic notch relative level; relative level of point **h**; (**h**– **a**) / (**c** (or **f**) – **a**) in mmHg/mmHg (dimensionless).

(mm) Dicrotic notch delay in ms, delay in ms of point **h**; (**h**– **a**).time interval between **a** and **h**.

(nn) Dicrotic notch relative delay; relative delay of point **h**; (**h**– **a**) / (**j**– **a**); in ms/ms (dimensionless).

(oo) [DiN in mmHg] – [AnN in mmHg] in mmHg; (**h**– **d**) BP difference between **d** and **h.**

(pp) [(DiN – AnN) in mmHg] / [dP/dt_min_ in mmHg ms^–1^] in mmHg/mmHg ms^–1^; (**h**– **d**) / **g.**

(qq) [(DiN – AnN) in mmHg] / [dP/dt_max_ in mmHg ms^–1^] in mmHg/mmHg ms^–1^; (**h**– **d**) / **b**.

(rr) [AnN in mmHg] – [1Max (point **c** or the 1st. maximum) in mmHg] in mmHg; (**d**– **c**) BP difference between **c** and **d**.

*Units in plots (gg), (hh), (pp) and (qq) are informative only.

**The plot of augmentation index relative (**jj**) was not possible to determine in cases when the highest point at APW was “c” and not “f” and it was set to zero (Kurakova et al. 2020; Tomasova et al. 2021).

**Definition of 17 parameters from APW**

Ten points **a - j** (in bold letters) are from Figure S1 and they mark the values of BP and time that are used to define (calculate) specific e-APW-Ps. Point '**e**' on APW is in the middle of points ‘**c**’ (the first maximum on APW) and ‘**h**’ (dicrotic notch).

**Parameters in mmHg:**

(b-e) Point **a** – point **e** = Diastolic BP – 'e' (in mmHg).

(c-e) Point **c** or **f** (Systolic BP) – point **e** = Systolic BP – 'e' (in mmHg).

(d-e) Point **c** – point **e** = 1Max – e (in mmHg).

(e-e) Point **b** – point **e** = dP/dt_max_ – e (in mmHg)

(f-e) Point **d** – point **e** = AnN – e (in mmHg).

(g-e) Point **g** – point **e** = dP/dt_min_ – e (in mmHg).

(h-e) Point **h** – point **e** = DiN – e (in mmHg).

(i-e) Point **i** – point **e** = dP/dt_d_ – e (mmHg).

(j-e) Point **e** = e (in mmHg)

**Parameters in ms:**

(k-e) Point **a** – point **e** = Diastolic BP – 'e' (in ms).

(l-e) Point **c** or **f** (Systolic BP) – point **e** = Systolic BP – 'e' (in ms).

(m-e) Point **c** – point **e** = 1Max – e (in ms).

(n-e) Point **b** – point **e** = dP/dt_max_ – e (in ms).

(o-e) Point **d** – point **e** = AnN – e (in ms).

(p-e) Point **g** – point **e** = dP/dt_min_ – e (in ms).

(q-e) Point **h** – point **e** = DiN – e (in ms).

(r-e) Point **i** – point **e** = dP/dt_d_ – e (ms).

**Definition of selected eleven APW-Ps**

Points on APW and details are described in (Supplementary FIGURE 1).

(a) Systolic blood pressure in mmHg; point **c** or **f**

(b) Heart rate in min^–1^; 60 / (**j**– **a**); (**j**– **a**) represents time interval between **a** and **j**, **a** and **j** are two reference points to diastolic BP value.

(ff) [Dicrotic notch (DiN) in s] – [Anacrotic notch (AnN) in s] in s; (**h**– **d**) time interval between **d** and **h**.

(s) [AnN in ms (point **d**)] – [Diast. BP in ms (point **a**)] in ms; (**d**– **a**) time interval between **d** and **a**.

(t) [AnN in ms (point **d**)] – [dP/dt in ms (point **b**)] in ms; (**d**– **b**) time interval between **d** and **b**.

(ii) [AnN in ms] – [1Max (point **c** or the 1st. maximum) in ms] in ms; (**d**– **c**) time interval between **c** and **d**.

(u) [dP/dt_max_, position in ms (point **b**)] – [Diast. BP in ms (point **a**)] in ms; (**b**– **a**) time interval between **b** and **a**.

(l-e) Point **c** or **f** (Systolic BP) – point **e** = Systolic BP – 'e' (in ms).

(jj) Augmentation index relative; (**f**– **c**) / (**f**– **a**) in mmHg/mmHg (dimensionless).

(v) [Systolic blood pressure in ms (point **c** or **f)] – [**Diastolic blood pressure in ms (point **a)].**

(w) [Systolic blood pressure in ms (point **c** or **f)] –** [dP/dt_max_, position in ms (point **b**)].

**Supplementary figures**


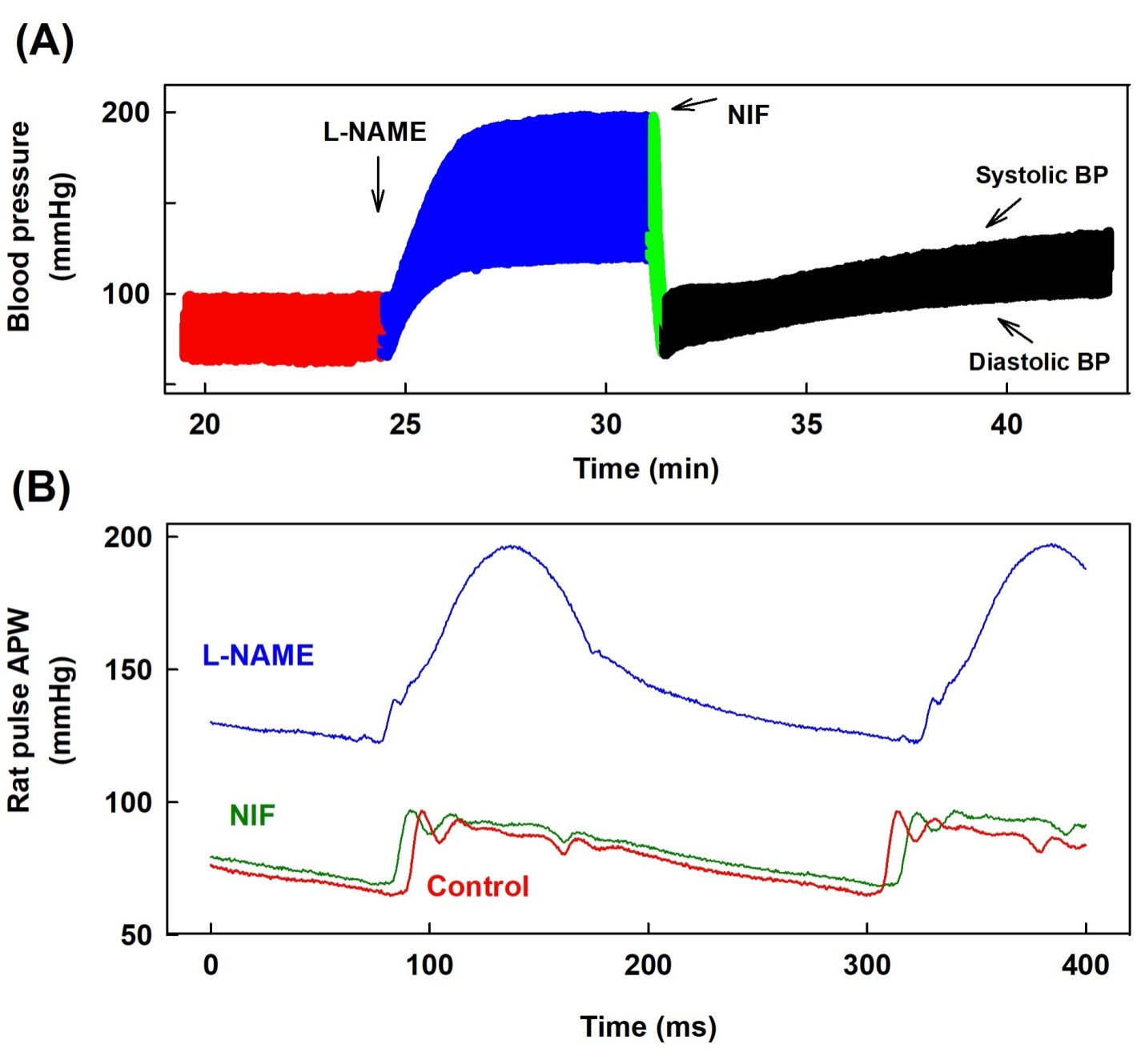


FIGURE S2 (A) Example of the time-dependent record of the left common carotid artery pulse waveform (APW) before (red) and after i.v. administration of 15 mg kg^–1^ of L-NAME (blue) and after subsequent i.v. administration of 400 nmol kg^–1^ of NIF (green and black). (B) Control APW (Control, red), L-NAME administration (blue) and after the subsequent administration of 400 nmol kg^–1^ of NIF (green; at the time of the minimum diastolic BP). Normotensive rats were anesthetized with Zoletil 100 (tiletamine+zolazepam, 80 mg kg^–1^, i.p.) and xylazine (5 mg kg^–1^, i.p.) (Misak et al. 2023).


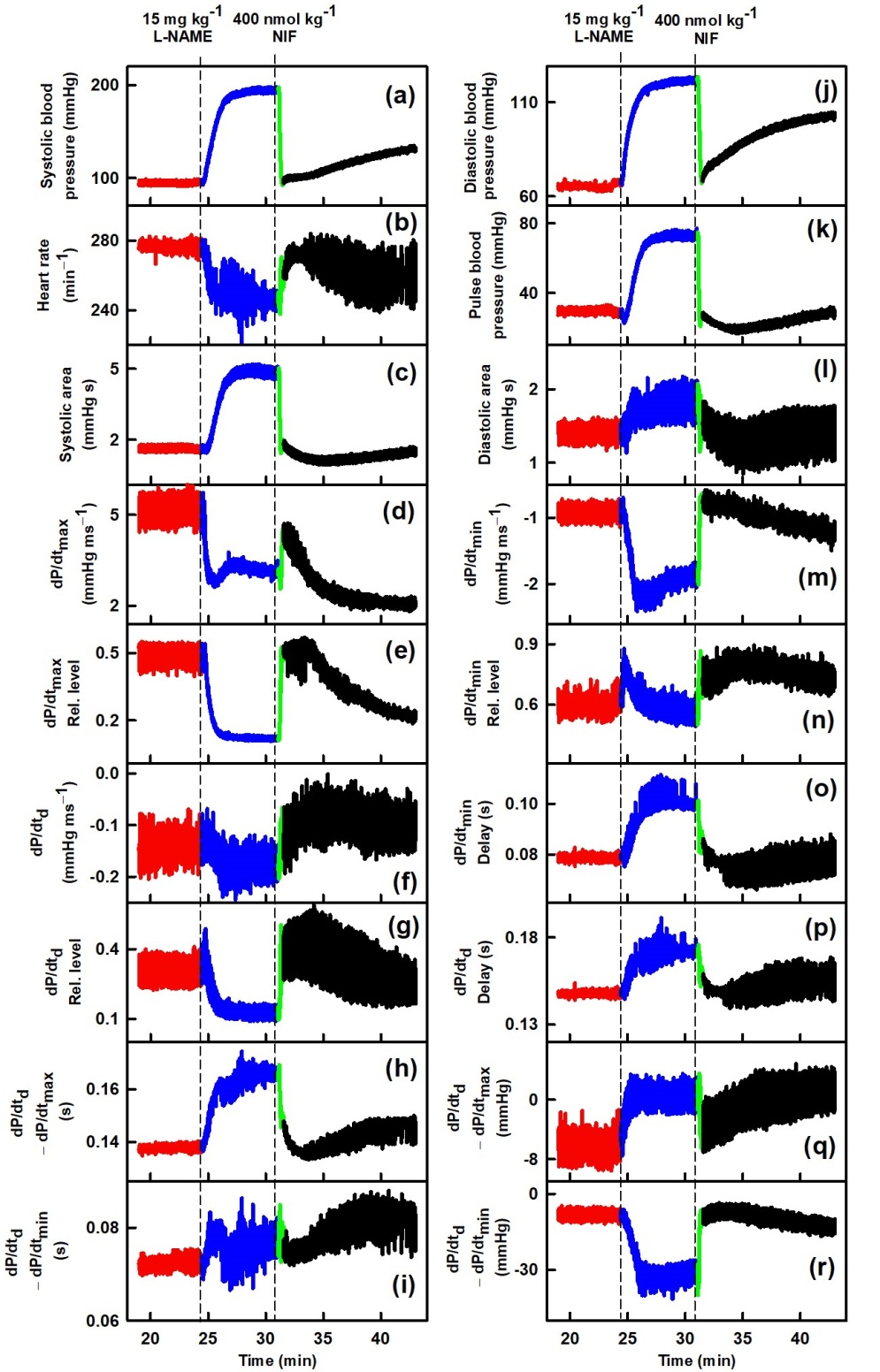


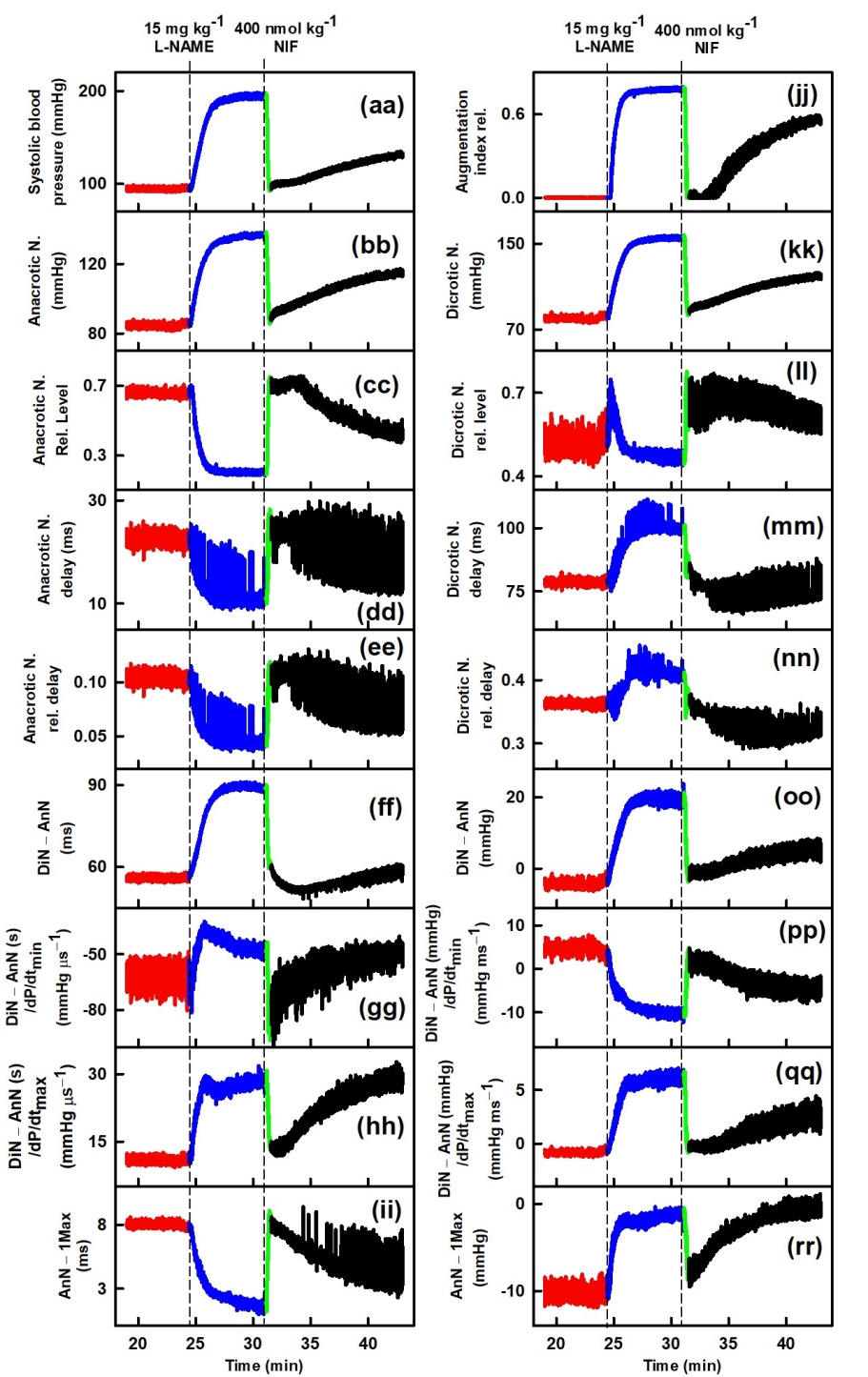


FIGURE S3 Exp-1. Time-dependent changes in 35 APW-Ps of anesthetized rats after i.v. administration of L-NAME and NIF. Control (red), administration of 15 mg kg^–1^ of L-NAME (blue) and 400 nmol kg^–1^ of NIF (decrease in systolic BP – green and subsequent increase in systolic BP – black). Data were obtained from the record shown in Fig. 1A. Definitions, units and abbreviations of APW-Ps evaluated from the APW are as explained previously (Misak et al. 2020; Kurakova et al. 2020; Tomasova et al. 2021) and briefly in Supplementary Information FIGURE S1. Normotensive rats were anesthetized with Zoletil/xylazine.


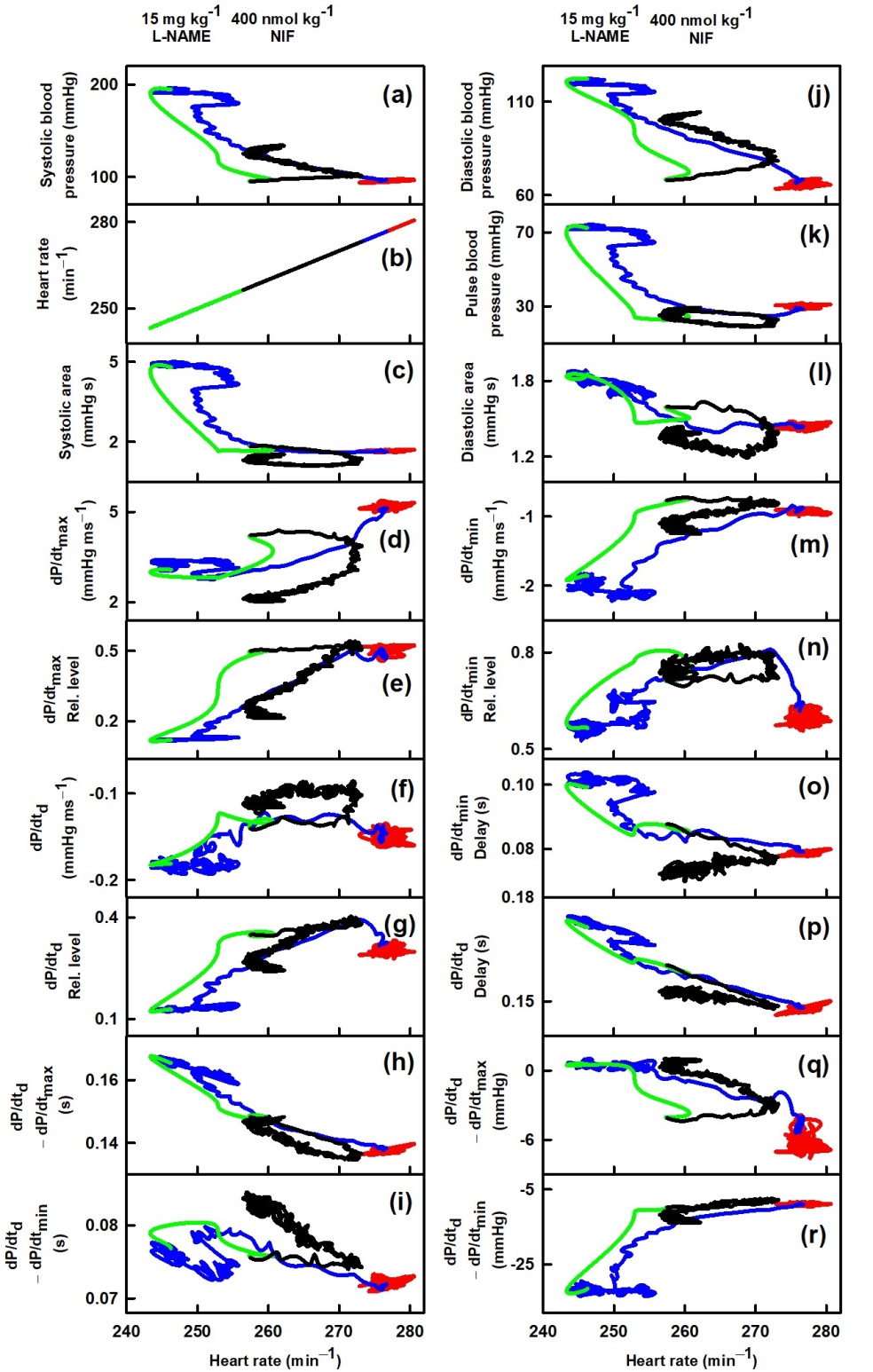


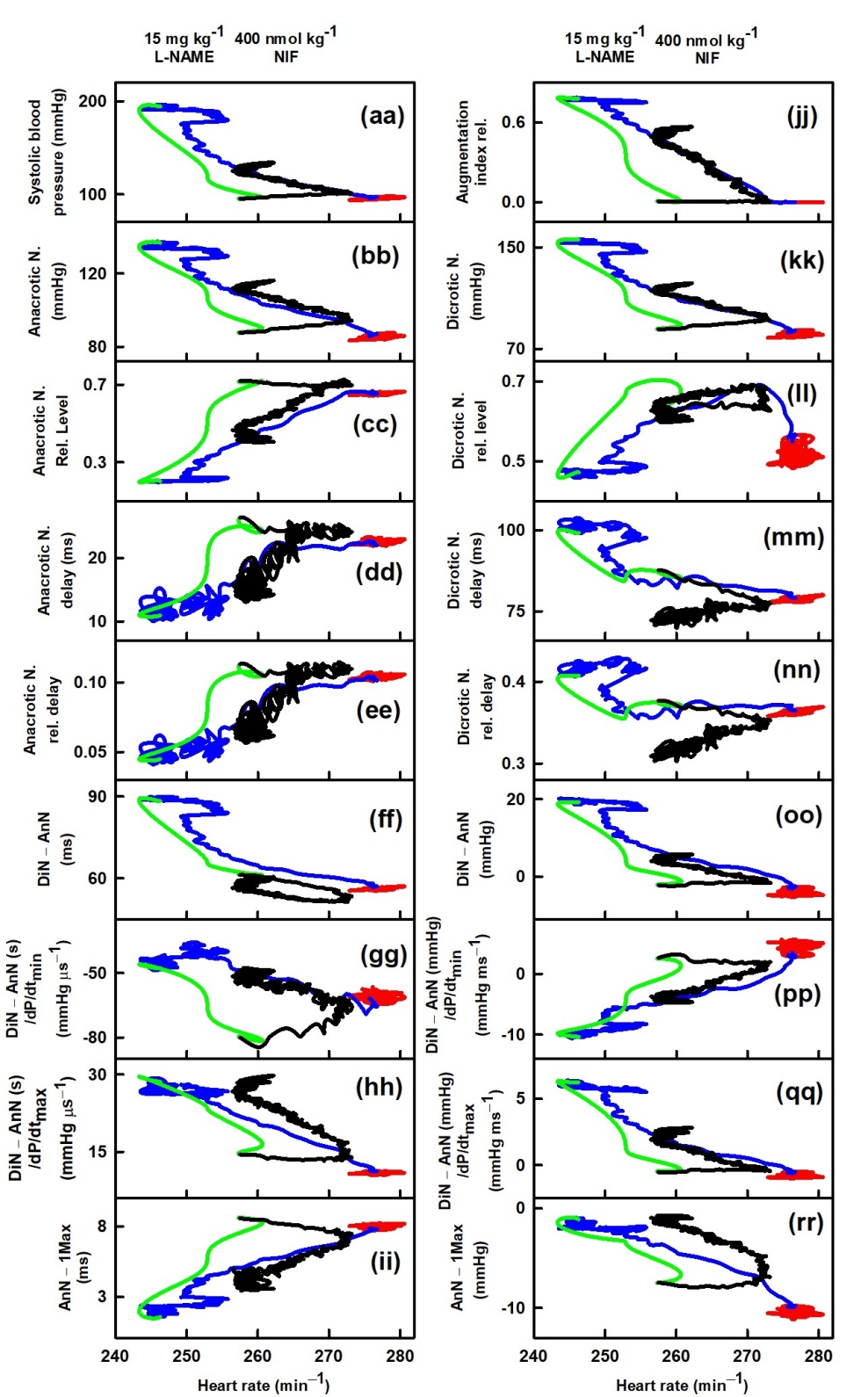


FIGURE S4 Exp-1. Cross-relationships of 34 APW-Ps to heart rate in control (red) and after the i.v. administration of 15 mg kg^–1^ of L-NAME (blue) and 400 nmol kg^–1^ of NIF (decrease in systolic BP – green and subsequent increase in systolic BP – black). Filtered data from Fig. S3 were used. Definitions, units and abbreviations of APW-Ps evaluated from the APW are as explained previously (Misak et al. 2020; Kurakova et al. 2020; Tomasova et al. 2021) and briefly in Supplementary Information FIGURE S1. Normotensive rats were anesthetized with Zoletil/xylazine.


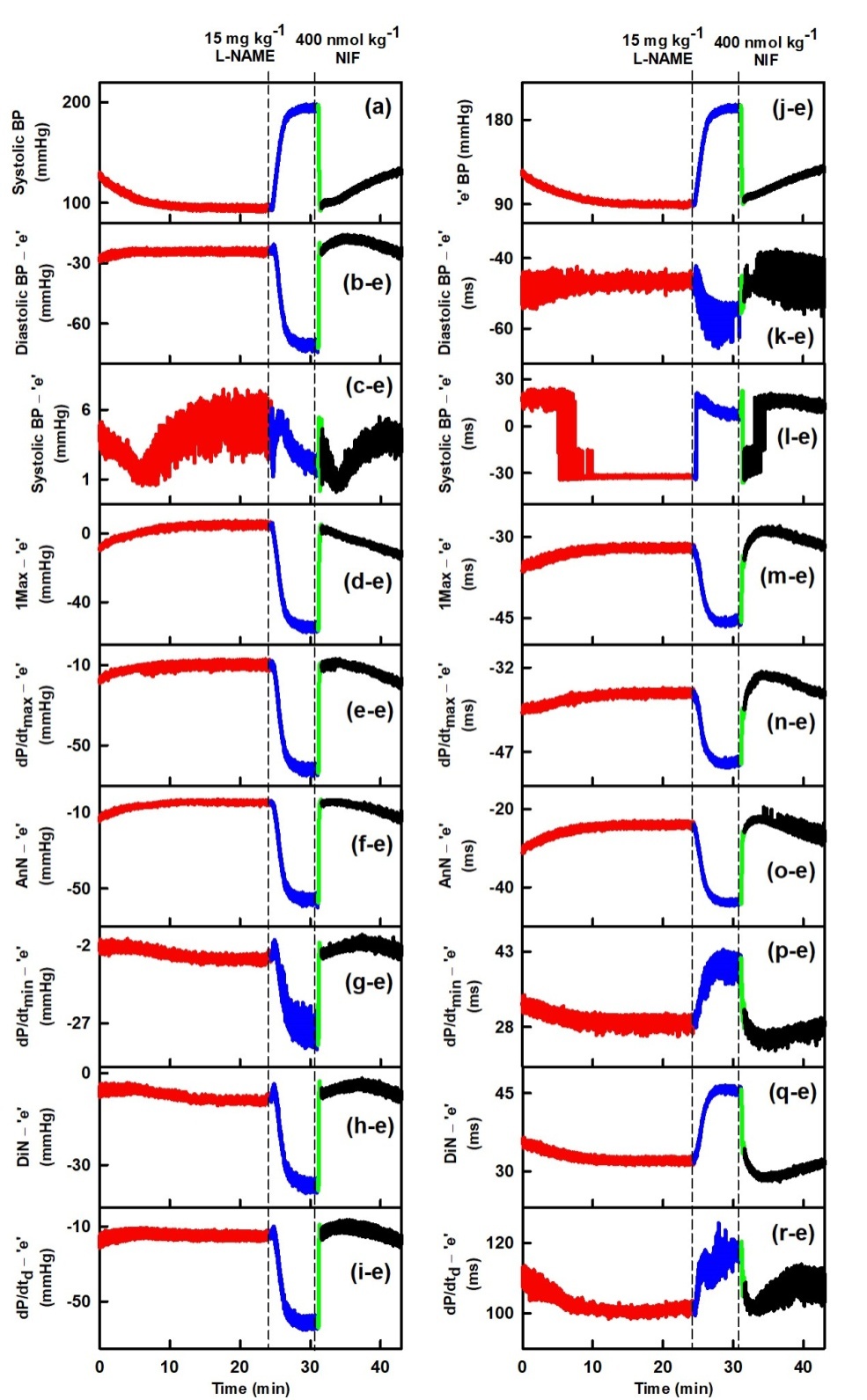


FIGURE S5 Exp-1. Time-dependent changes in 18 APW-Ps of anesthetized rat in control (red) and after i.v. administration of 15 mg kg^–1^ of L-NAME (blue) and 400 nmol kg^–1^ of NIF (decrease in systolic BP – green and subsequent increase in systolic BP – black). Definitions, units and abbreviations of APW-Ps evaluated from the APW are as explained in Supplementary Information FIGURE S1. Normotensive rats were anesthetized with Zoletil/xylazine.


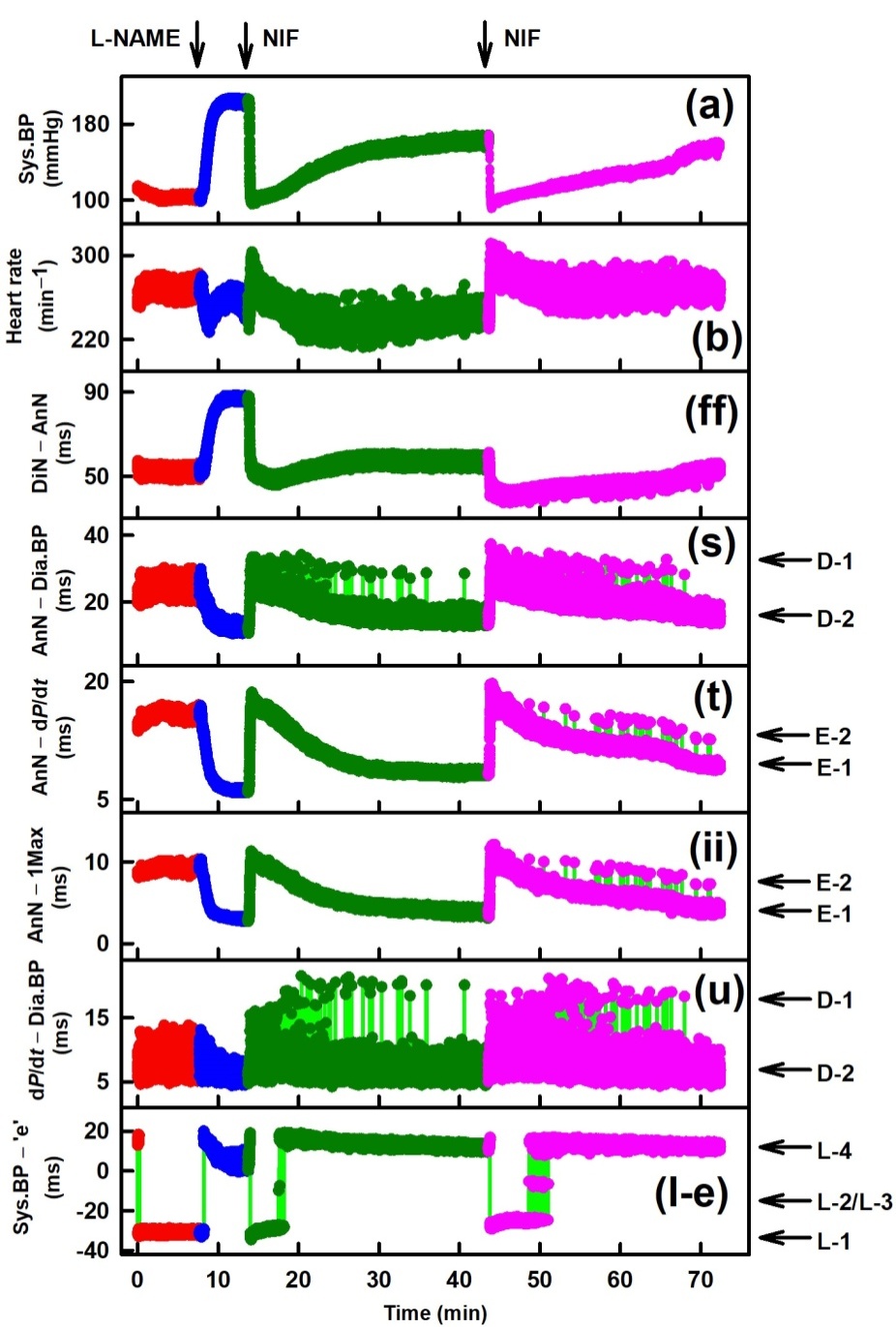


FIGURE S6 Exp-2. Time-dependent changes in APW-Ps, systolic BP (mmHg) (a), heart rate (min^–1^) (b), DiN-AnN (ms) (ff), AnN – Dia.BP (ms) (s), AnN – dP/dt (anacrotic notch – d*P*/d*t*_max_, ms) (t), AnN – 1Max (ms) (ii), dP/dt – Dia.BP (d*P*/d*t*_max_ – diastolic BP, u) and Sys.BP – ‘e’ (ms) (l-e) of anesthetized rat: control (red heartbeats), after i.v. administration of 15 mg kg^–1^ L-NAME (blue heartbeats) and 400 nmol kg^–1^ NIF (dark green heartbeats) and the second administration of 400 nmol kg^–1^ NIF (pink heartbeats). Red, blue, dark green and pink points are individual heartbeats. Individual heartbeats are clearly visible in next Figures. Horizontal arrows indicate predicted D-1 and D-2 levels, E-1 and E-2 levels and L-1 to L-4 levels. The green lines show the connection between adjacent heartbeats. Definitions, units and abbreviations of APW-Ps evaluated from the APW are as explained in Supplementary Information FIGURE S1. Normotensive rats were anesthetized with Zoletil/xylazine.


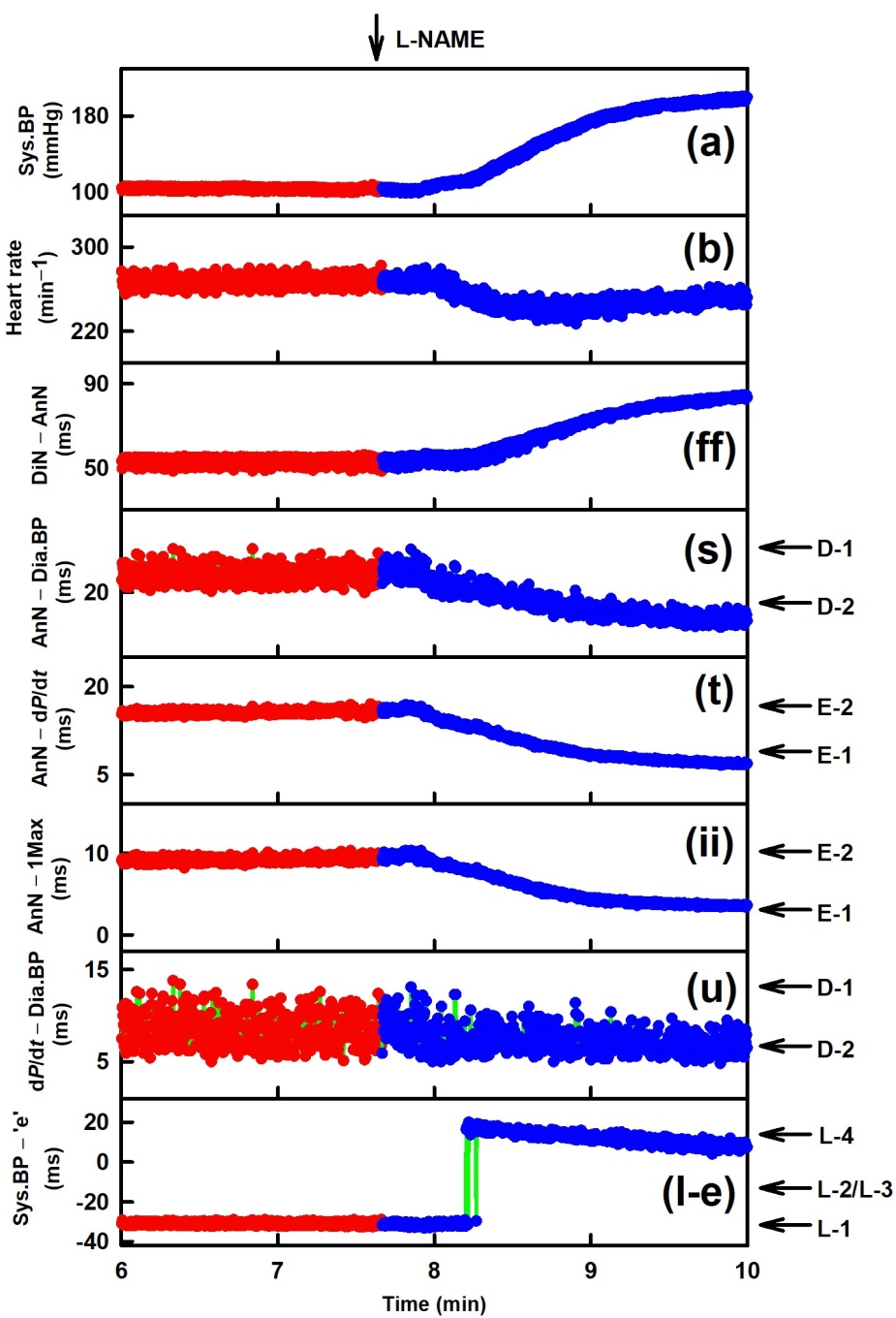


FIGURE S7 Exp-2. Time-dependent changes in APW-Ps, control (red heartbeats) and after i.v. administration of 15 mg kg^–1^ L-NAME (blue heartbeats) Individual heartbeats are clearly visible in next Figures. Horizontal arrows indicate predicted D-1 and D-2 levels, E-1 and E-2 levels and L-1 to L-4 levels. The green lines show the connection between adjacent heartbeats. Definitions, units and abbreviations of APW-Ps evaluated from the APW are as explained in legend to FIGURE S6 and Supplementary Information FIGURE S1. Normotensive rats were anesthetized with Zoletil/xylazine.


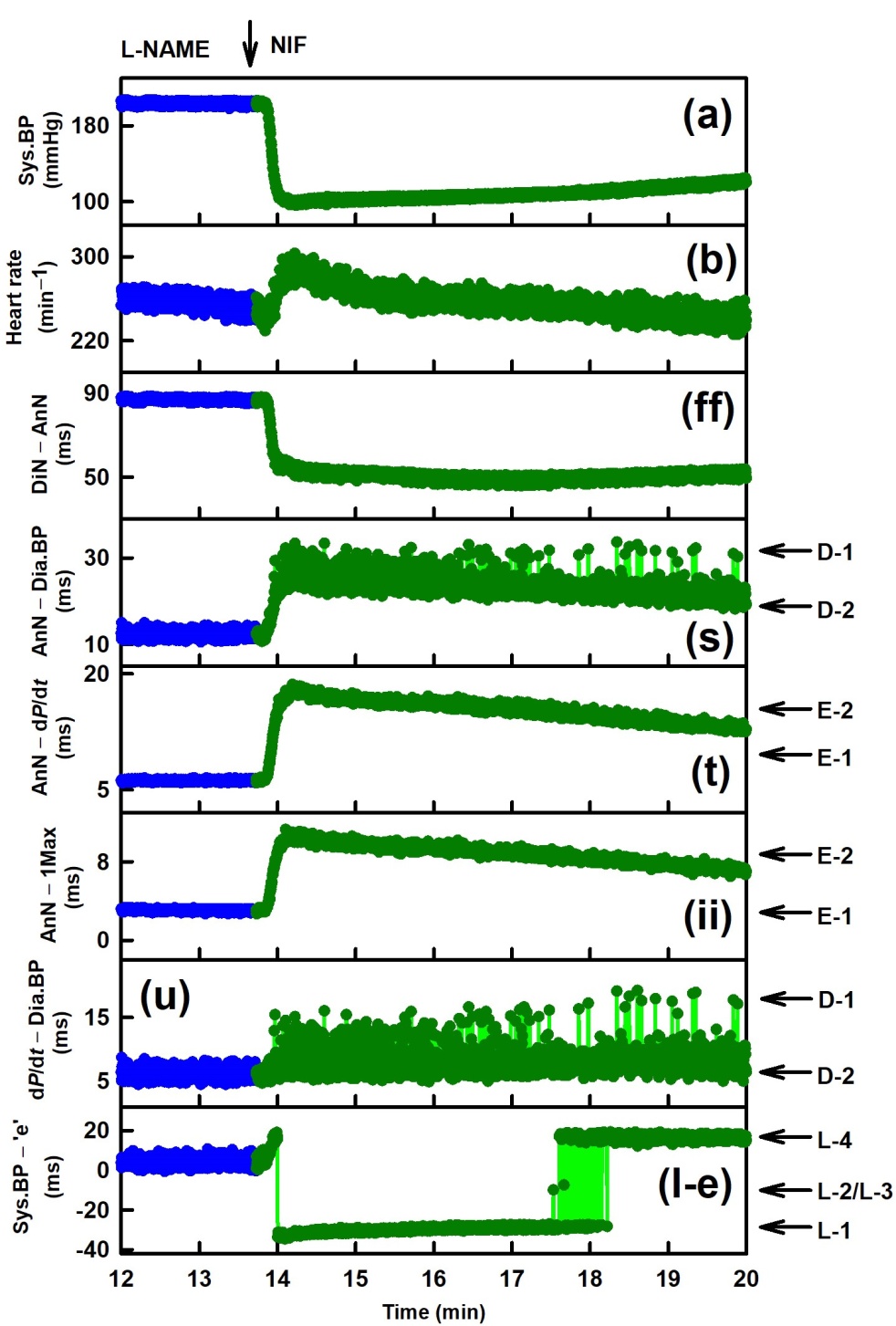


FIGURE S8 Exp-2. Time-dependent changes in APW-Ps of anesthetized rat after i.v. administration of 15 mg kg^–1^ L-NAME (blue heartbeats) and subsequent administration of 400 nmol kg^–1^ NIF (dark green heartbeats). Horizontal arrows indicate predicted D-1 and D-2 levels, E-1 and E-2 levels and L-1 to L-4 levels. The green lines show the connection between adjacent heartbeats. Definitions, units and abbreviations of APW-Ps evaluated from the APW are as explained in legend to FIGURE S6 and Supplementary Information FIGURE S1. Normotensive rats were anesthetized with Zoletil/xylazine.


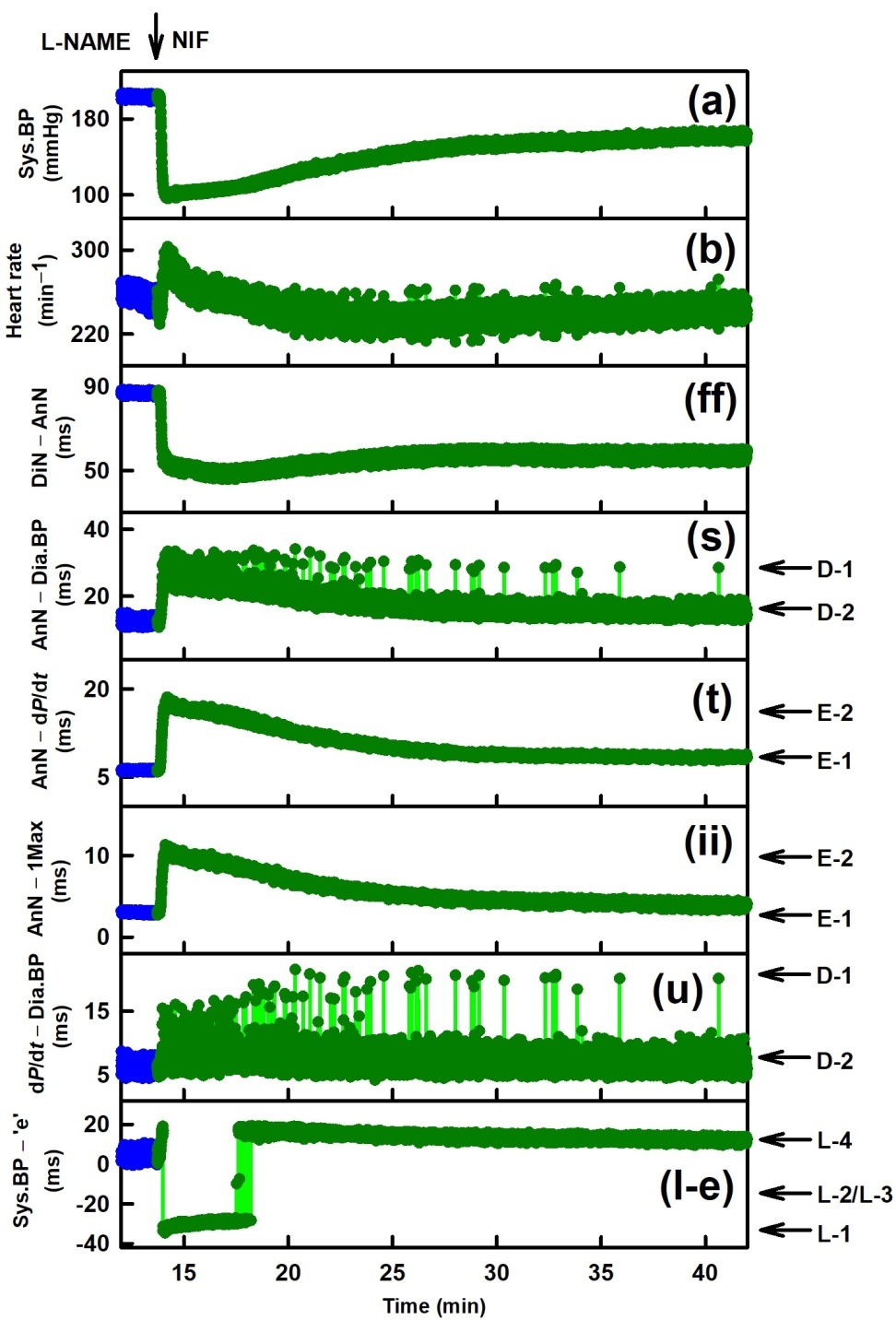


FIGURE S9 Exp-2. Time-dependent changes in APW-Ps of anesthetized rat after i.v. administration of 15 mg kg^–1^ L-NAME (blue heartbeats) and subsequent administration of 400 nmol kg^–1^ NIF (dark green heartbeats). Horizontal arrows indicate predicted D-1 and D-2 levels, E-1 and E-2 levels and L-1 to L-4 levels. The green lines show the connection between adjacent heartbeats. Definitions, units and abbreviations of APW-Ps evaluated from the APW are as explained in legend to FIGURE S6 and Supplementary Information FIGURE S1. Normotensive rats were anesthetized with Zoletil/xylazine.


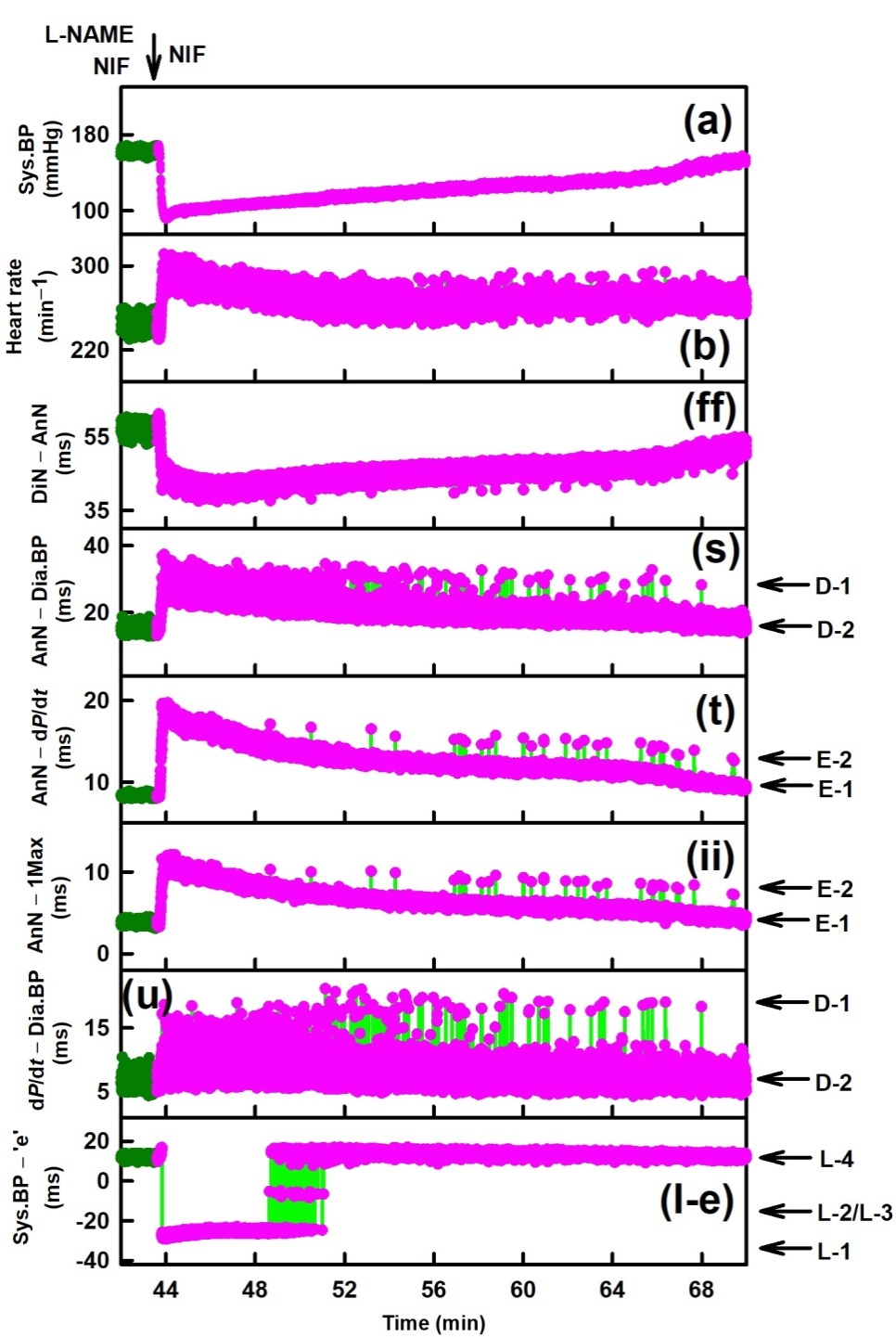


FIGURE S10 Exp-2. Time-dependent changes in APW-Ps of anesthetized rat after i.v. administration of 400 nmol kg^–1^ NIF (dark green heartbeats) and second subsequent administration of 400 nmol kg^–1^ NIF (pink heartbeats). Horizontal arrows indicate predicted D-1 and D-2 levels, E-1 and E-2 levels and L-1 to L-4 levels. The green lines show the connection between adjacent heartbeats. Definitions, units and abbreviations of APW-Ps evaluated from the APW are as explained in legend to FIGURE S6 and Supplementary Information FIGURE S1. Normotensive rats were anesthetized with Zoletil/xylazine.


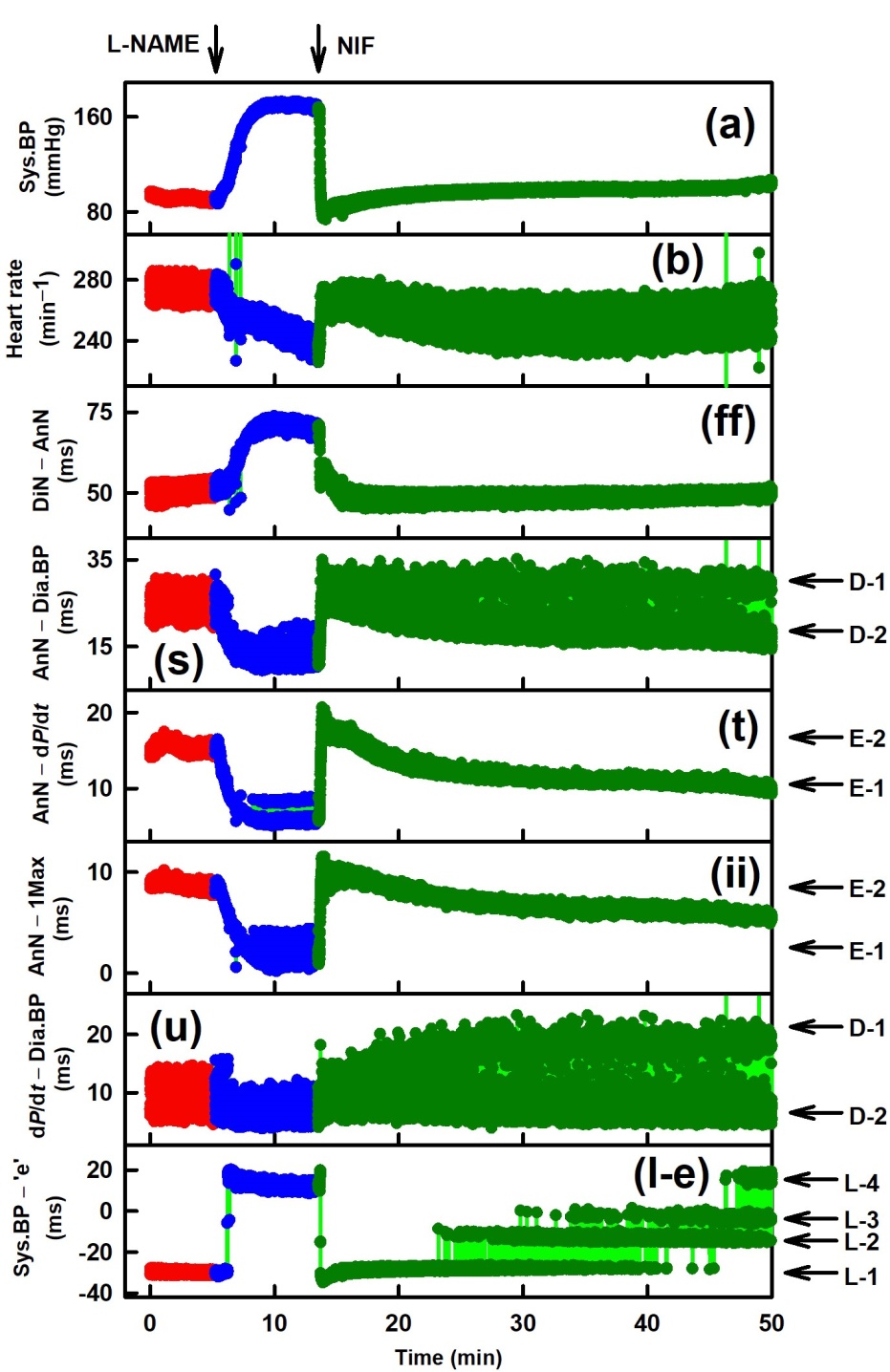


FIGURE S11 Exp-3. Time-dependent changes in APW-Ps of anesthetized rat: control (red heartbeats), after i.v. administration of 15 mg kg^–1^ L-NAME (blue heartbeats) and 400 nmol kg^–1^ NIF (dark green heartbeats). Red, blue and dark green are individual heartbeats. Horizontal arrows indicate predicted D-1 and D-2 levels, E-1 and E-2 levels and L-1 to L-4 levels. The green lines show the connection between adjacent heartbeats. Definitions, units and abbreviations of APW-Ps evaluated from the APW are as explained in legend to FIGURE S6 and Supplementary Information FIGURE S1. Normotensive rats were anesthetized with Zoletil/xylazine.

**
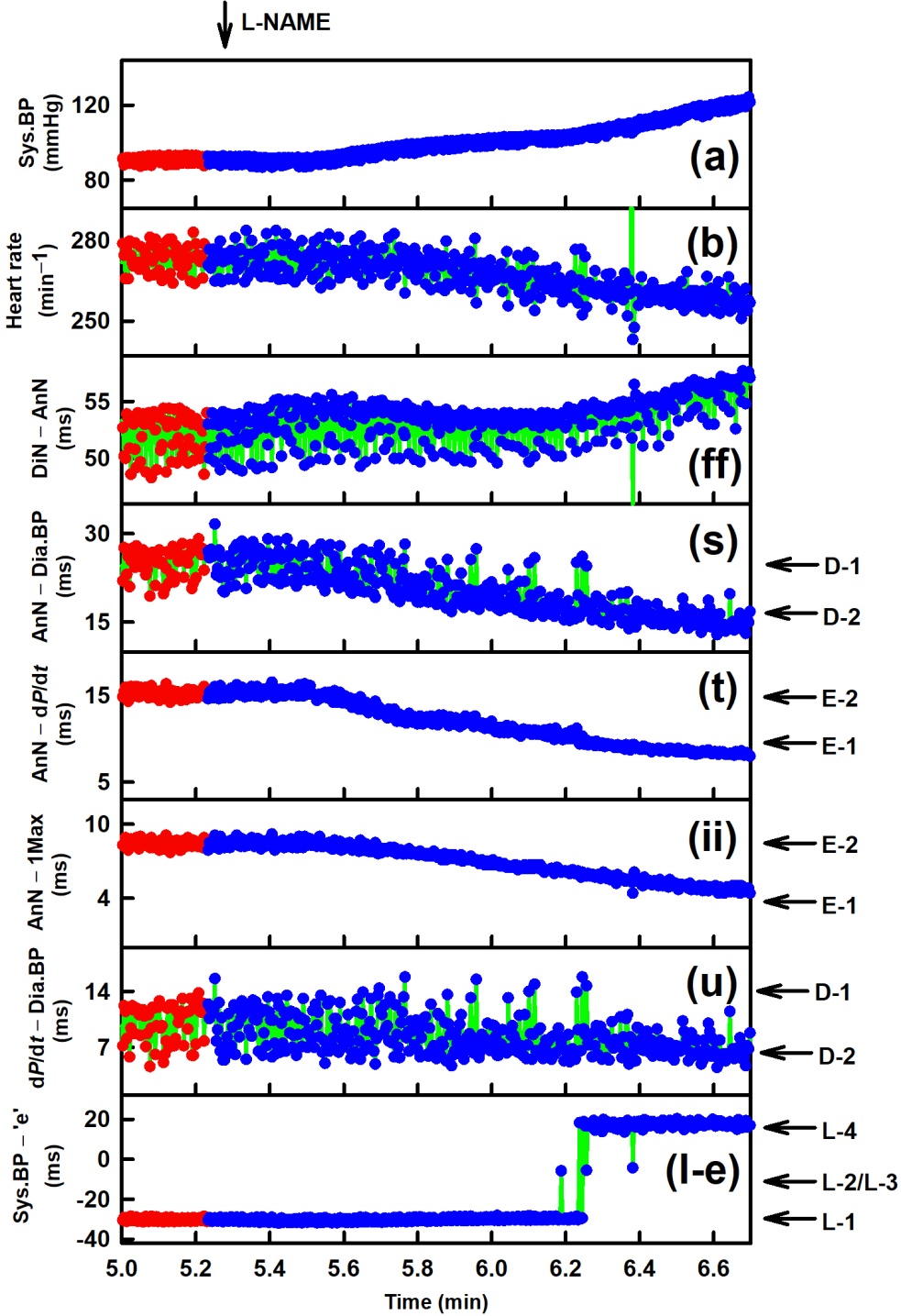
**

FIGURE S12 Exp-3. Time-dependent changes in APW-Ps of anesthetized rat: control (red heartbeats) and after i.v. administration of 15 mg kg^–1^ L-NAME (blue heartbeats) Horizontal arrows indicate predicted D-1 and D-2 levels, E-1 and E-2 levels and L-1 to L-4 levels. The green lines show the connection between adjacent heartbeats. Definitions, units and abbreviations of APW-Ps evaluated from the APW are as explained in legend to FIGURE S6 and Supplementary Information FIGURE S1. Normotensive rats were anesthetized with Zoletil/xylazine.

**
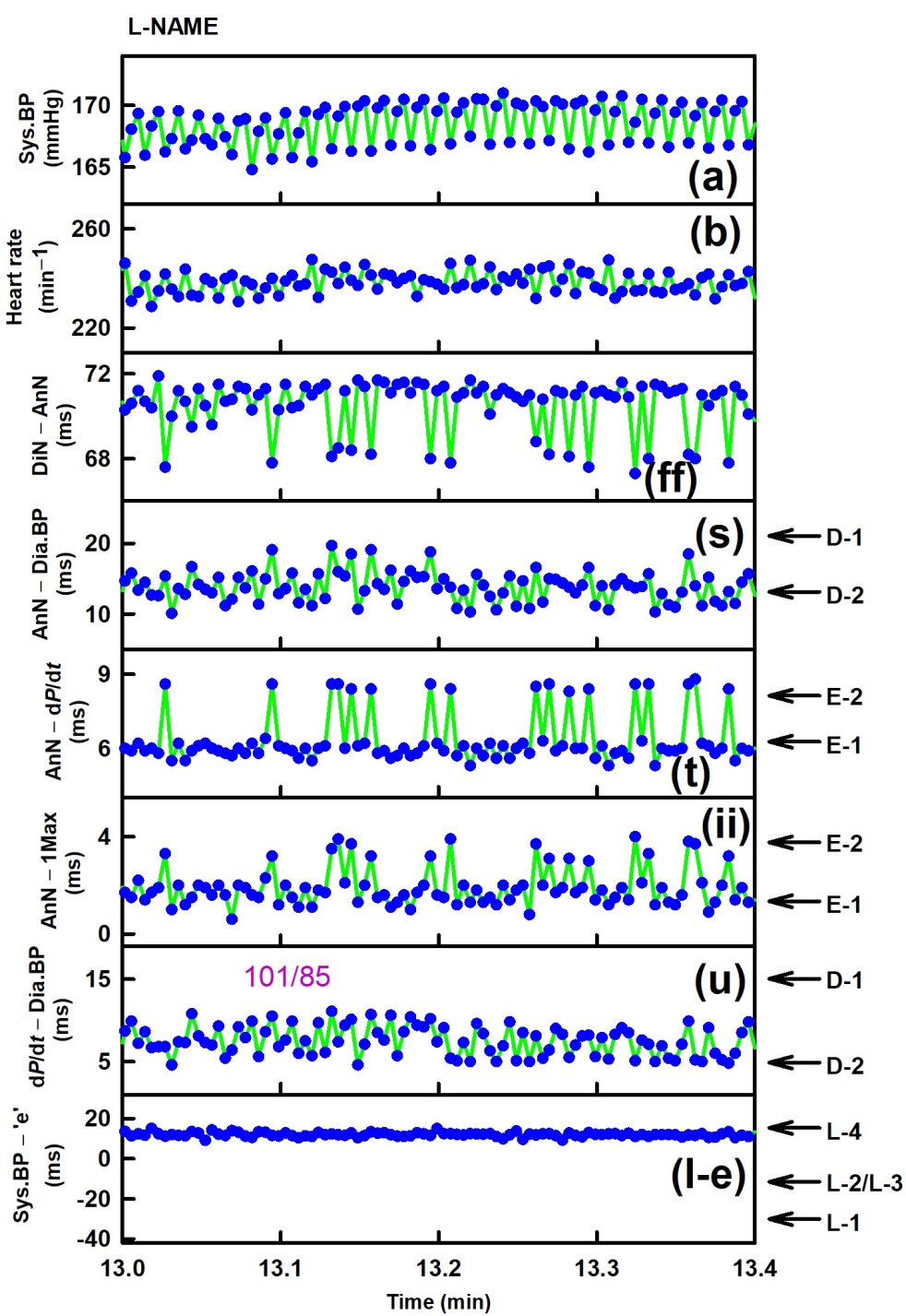
**

FIGURE S13 Exp-3. Time-dependent changes in APW-Ps of anesthetized rat after i.v. administration of 15 mg kg^–1^ L-NAME (blue heartbeats). Horizontal arrows indicate predicted D-1 and D-2 levels, E-1 and E-2 levels and L-1 to L-4 levels. The green lines show the connection between adjacent heartbeats. Definitions, units and abbreviations of APW-Ps evaluated from the APW are as explained in legend to FIGURE S6 and Supplementary Information FIGURE S1. Normotensive rats were anesthetized with Zoletil/xylazine.


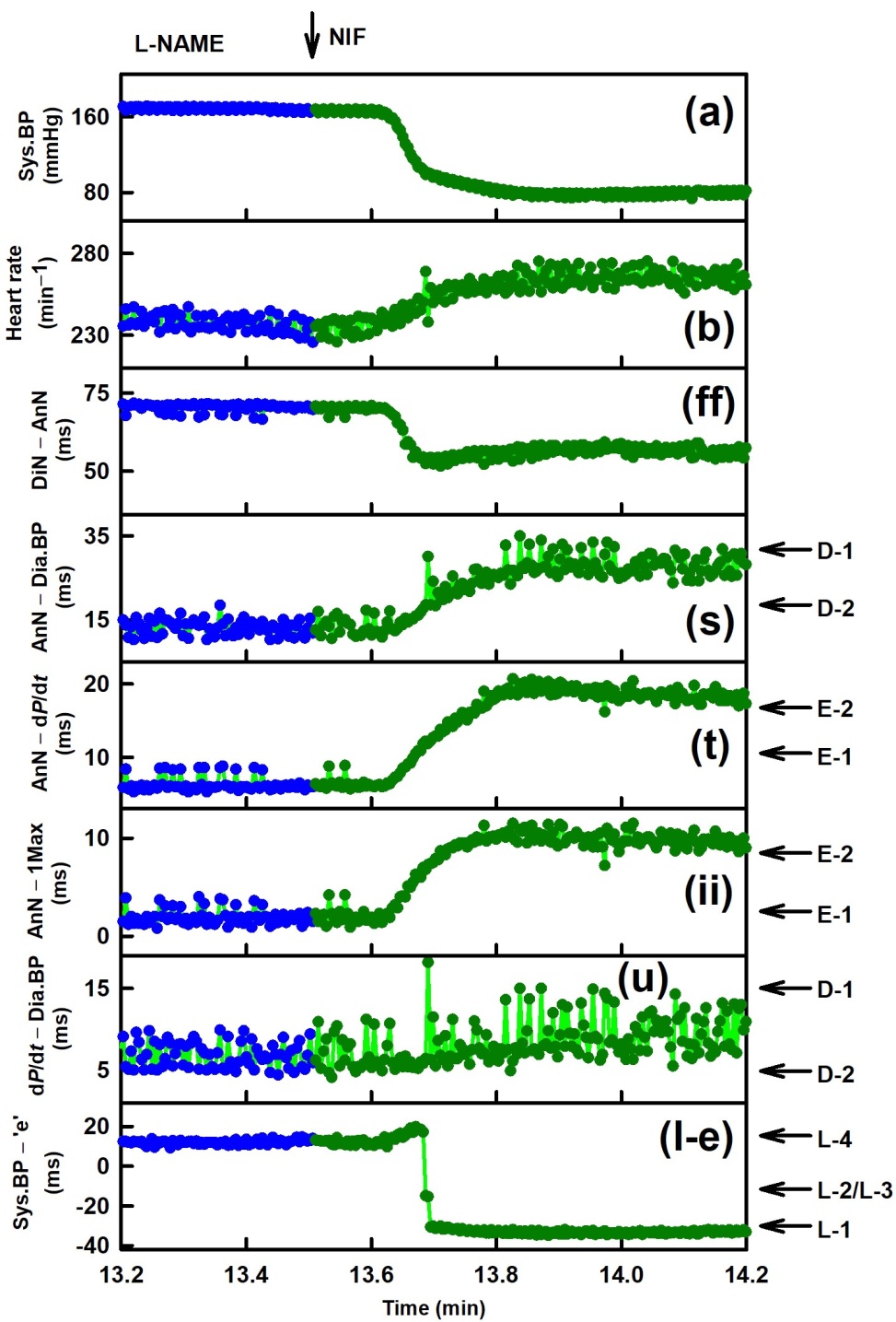


FIGURE S14 Exp-3. Time-dependent changes in APW-Ps of anesthetized rat after i.v. administration of 15 mg kg^–1^ L-NAME (blue heartbeats) and subsequent administration of 400 nmol kg^–1^ NIF (dark green heartbeats). Horizontal arrows indicate predicted D-1 and D-2 levels, E-1 and E-2 levels and L-1 to L-4 levels. The green lines show the connection between adjacent heartbeats. Definitions, units and abbreviations of APW-Ps evaluated from the APW are as explained in legend to FIGURE S6 and Supplementary Information FIGURE S1. Normotensive rats were anesthetized with Zoletil/xylazine.


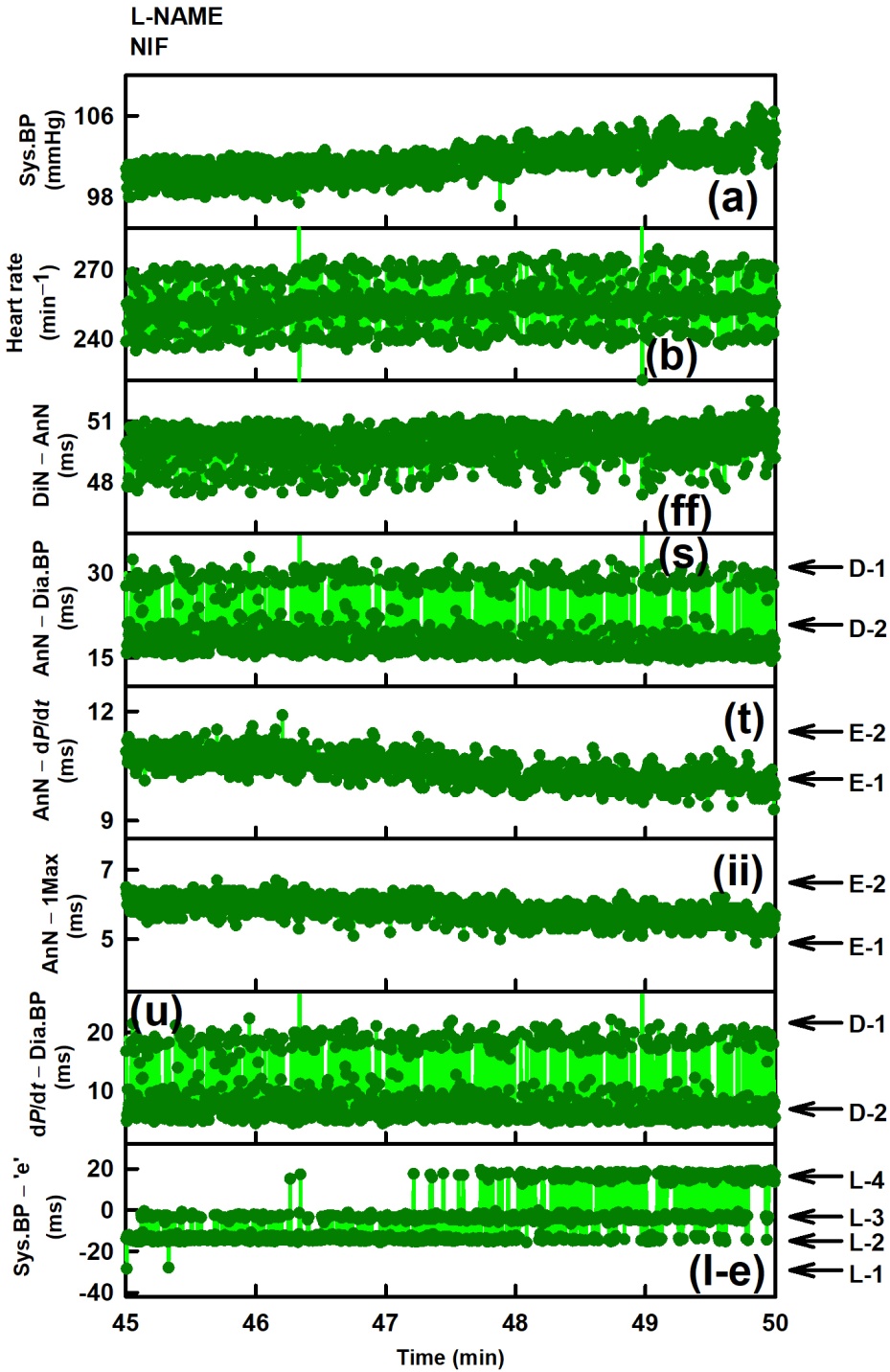


FIGURE S15 Exp-3. Time-dependent changes in APW-Ps of anesthetized rat in the presence of 15 mg kg^–1^ L-NAME and subsequent administration of 400 nmol kg^–1^ NIF (dark green heartbeats). Horizontal arrows indicate predicted D-1 and D-2 levels, E-1 and E-2 levels and L-1 to L-4 levels. The green lines show the connection between adjacent heartbeats. Definitions, units and abbreviations of APW-Ps evaluated from the APW are as explained in legend to FIGURE S6 and Supplementary Information FIGURE S1. Normotensive rats were anesthetized with Zoletil/xylazine.


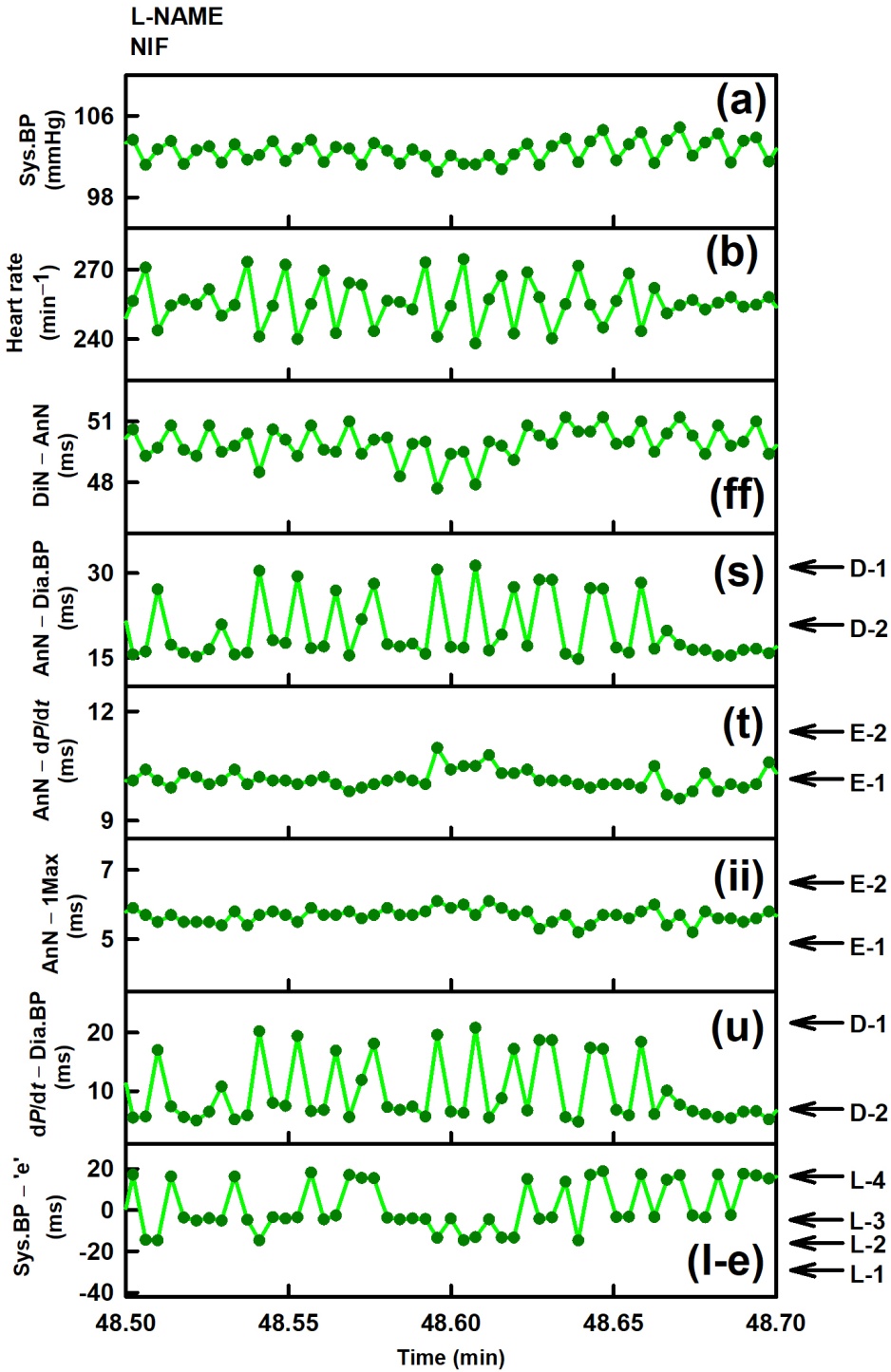


FIGURE S16 Exp-3. Details of the time-dependent changes in APW-Ps of anesthetized rat in the presence of 15 mg kg^–1^ L-NAME and subsequent administration of 400 nmol kg^–1^ NIF (dark green heartbeats). Horizontal arrows indicate predicted D-1 and D-2 levels, E-1 and E-2 levels and L-1 to L-4 levels. The green lines show the connection between adjacent heartbeats. Definitions, units and abbreviations of APW-Ps evaluated from the APW are as explained in legend to FIGURE S6 and Supplementary Information FIGURE S1. Normotensive rats were anesthetized with Zoletil/xylazine.


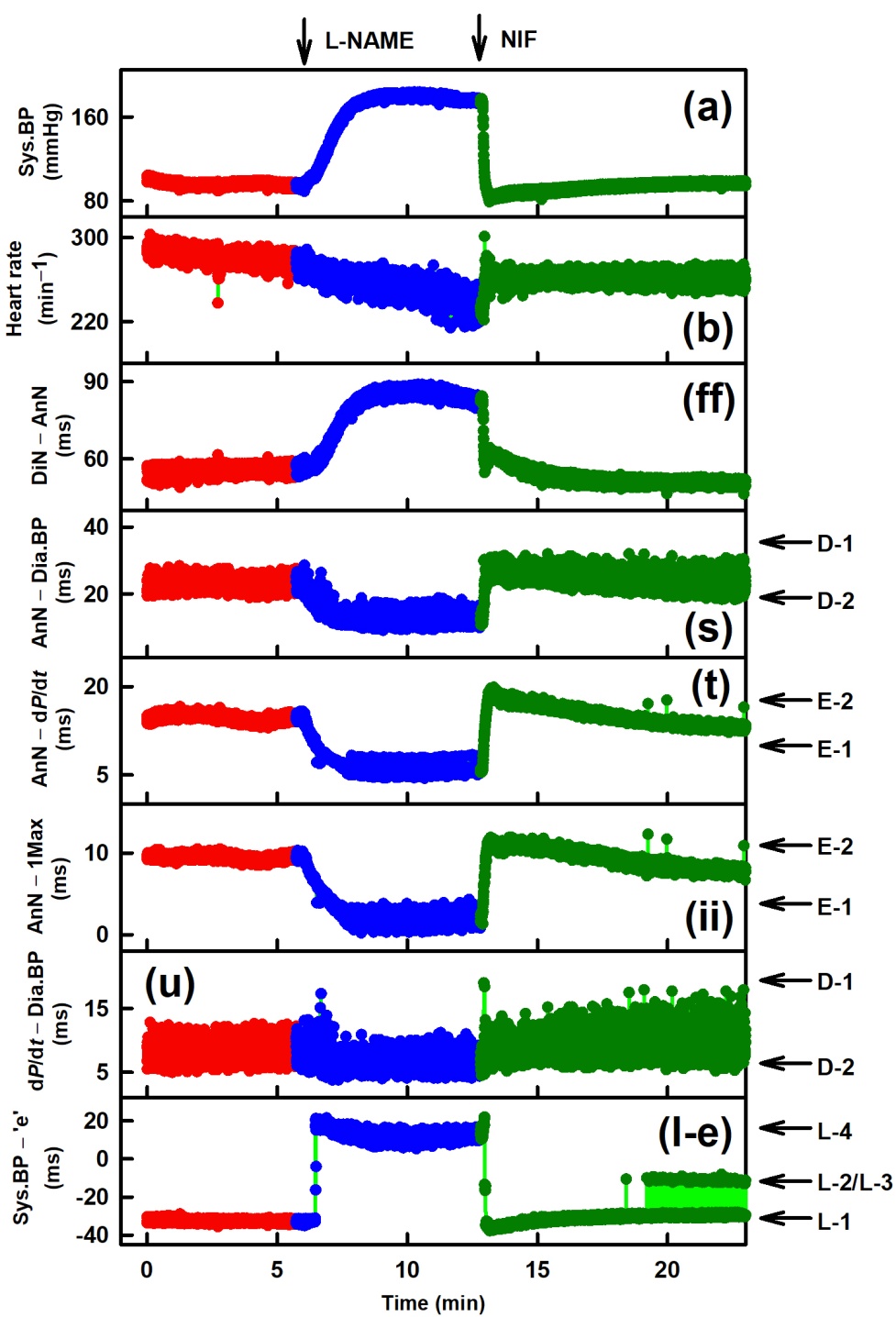


FIGURE S17 Exp-4. Time-dependent changes in APW-Ps of anesthetized rat: control (red heartbeats), after i.v. administration of 15 mg kg^–1^ L-NAME (blue heartbeats) and 400 nmol kg^–1^ NIF (dark green heartbeats). Red, blue and dark green are individual heartbeats. Horizontal arrows indicate predicted D-1 and D-2 levels, E-1 and E-2 levels and L-1 to L-4 levels. The green lines show the connection between adjacent heartbeats. Definitions, units and abbreviations of APW-Ps evaluated from the APW are as explained in legend to FIGURE S6 and Supplementary Information FIGURE S1. Normotensive rats were anesthetized with Zoletil/xylazine.


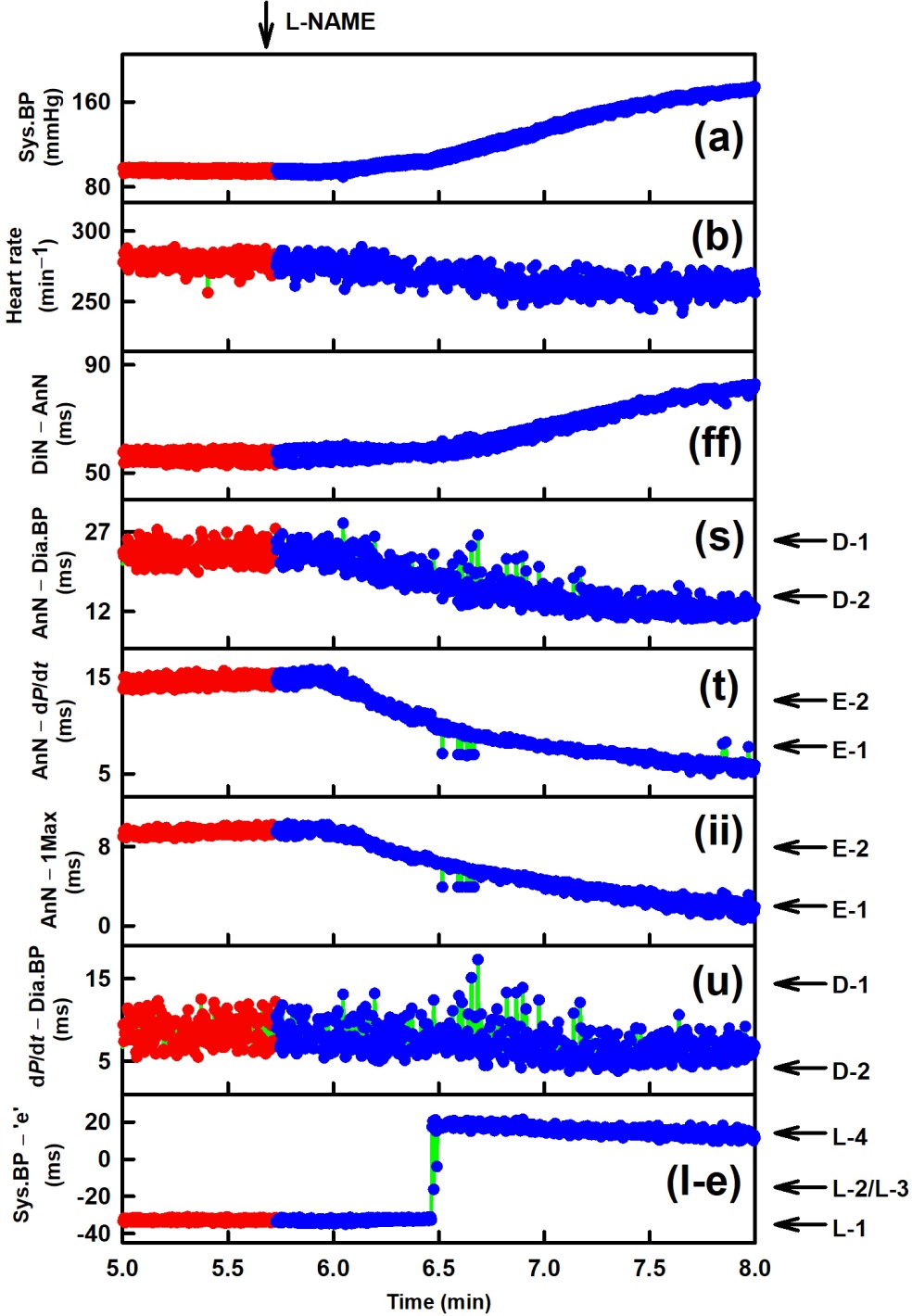


FIGURE S18 Exp-4. Time-dependent changes in APW-Ps of anesthetized rat: control (red heartbeats) and after i.v. administration of 15 mg kg^–1^ L-NAME (blue heartbeats) Horizontal arrows indicate predicted D-1 and D-2 levels, E-1 and E-2 levels and L-1 to L-4 levels. The green lines show the connection between adjacent heartbeats. Definitions, units and abbreviations of APW-Ps evaluated from the APW are as explained in legend to FIGURE S6 and Supplementary Information FIGURE S1. Normotensive rats were anesthetized with Zoletil/xylazine.


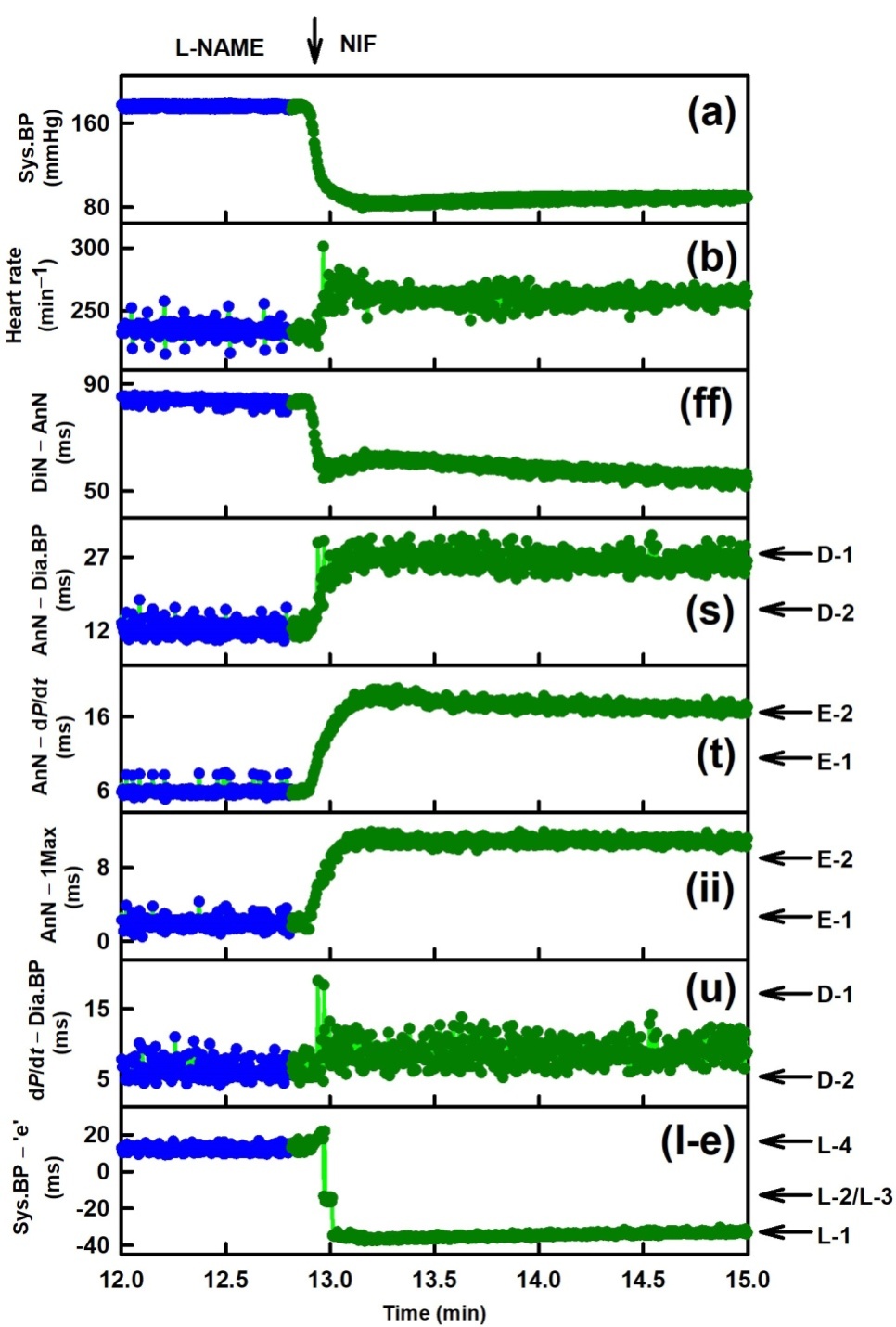


FIGURE S19 Exp-4. Time-dependent changes in APW-Ps of anesthetized rat after i.v. administration of 15 mg kg^–1^ L-NAME (blue heartbeats) and subsequent administration of 400 nmol kg^–1^ NIF (dark green heartbeats). Horizontal arrows indicate predicted D-1 and D-2 levels, E-1 and E-2 levels and L-1 to L-4 levels. The green lines show the connection between adjacent heartbeats. Definitions, units and abbreviations of APW-Ps evaluated from the APW are as explained in legend to FIGURE S6 and Supplementary Information FIGURE S1. Normotensive rats were anesthetized with Zoletil/xylazine.


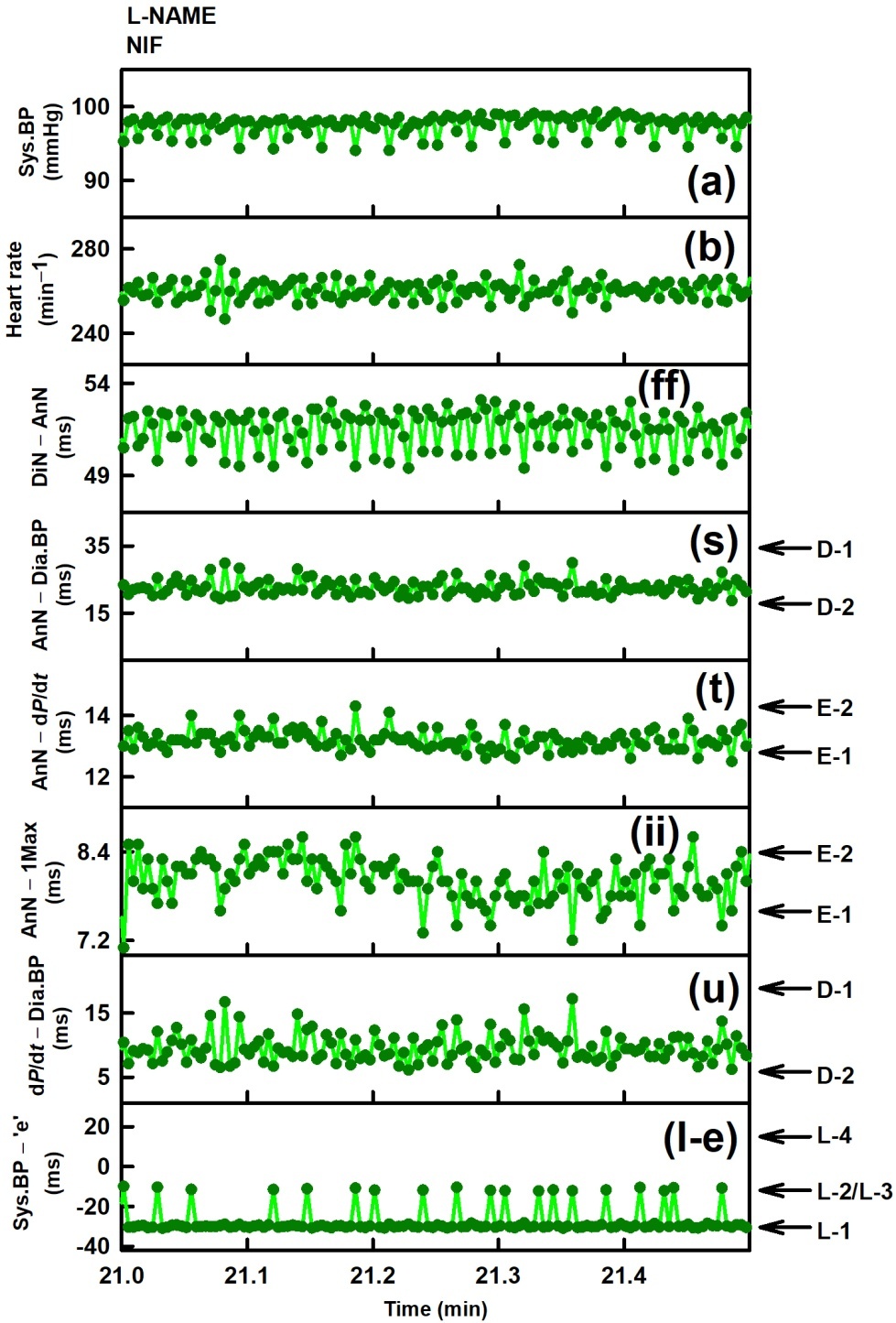


FIGURE S20 Exp-4. Details of the time-dependent changes in APW-Ps of anesthetized rat in the presence of 15 mg kg^–1^ L-NAME and subsequent administration of 400 nmol kg^–1^ NIF (dark green heartbeats). Horizontal arrows indicate predicted D-1 and D-2 levels, E-1 and E-2 levels and L-1 to L-4 levels. The green lines show the connection between adjacent heartbeats. Definitions, units and abbreviations of APW-Ps evaluated from the APW are as explained in legend to FIGURE S6 and Supplementary Information FIGURE S1. Normotensive rats were anesthetized with Zoletil/xylazine.

**
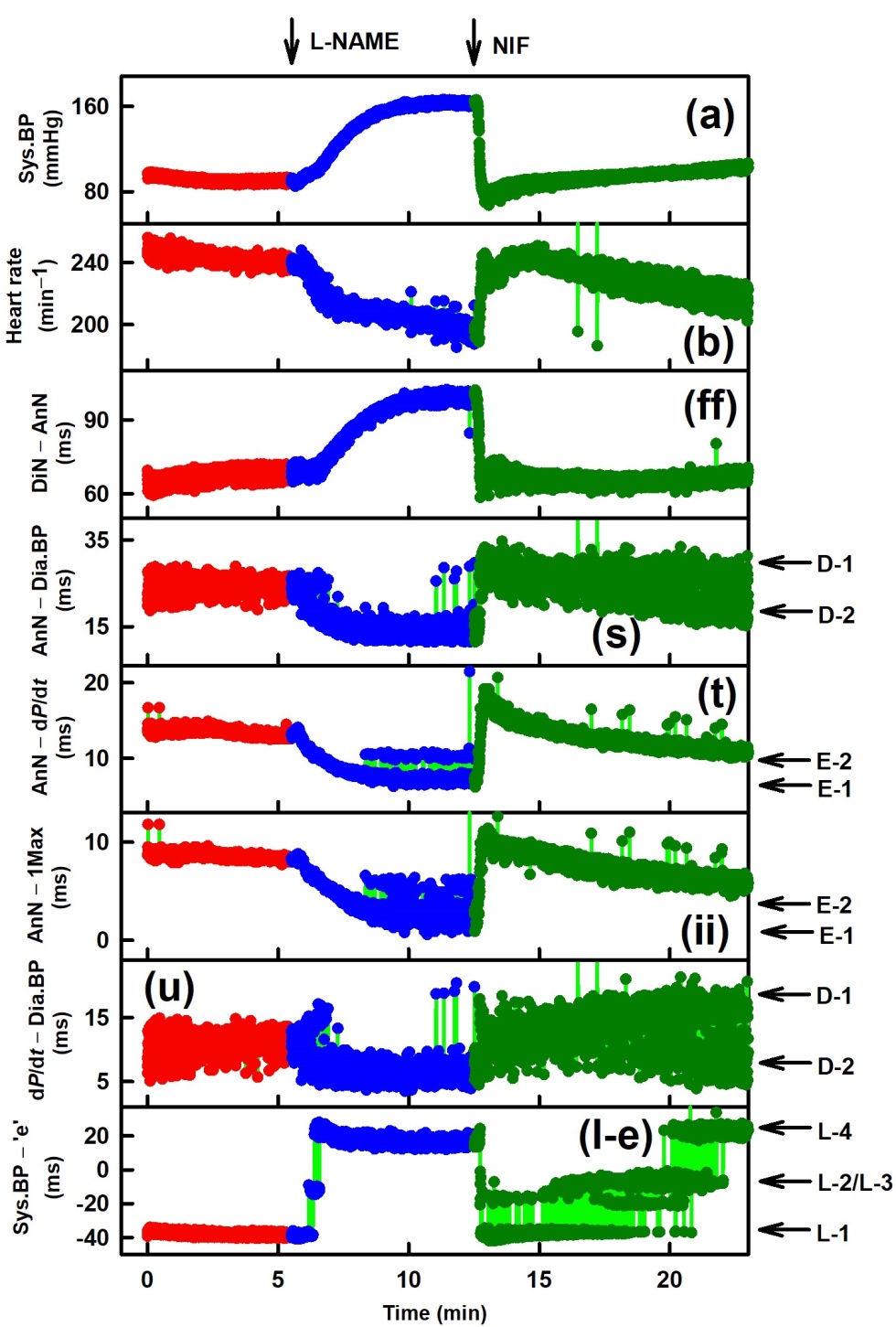
**

FIGURE S21 Exp-5. Time-dependent changes in APW-Ps of anesthetized rat: control (red heartbeats), after i.v. administration of 15 mg kg^–1^ L-NAME (blue heartbeats) and 400 nmol kg^–1^ NIF (dark green heartbeats). Horizontal arrows indicate predicted D-1 and D-2 levels, E-1 and E-2 levels and L-1 to L-4 levels. The green lines show the connection between adjacent heartbeats. Definitions, units and abbreviations of APW-Ps evaluated from the APW are as explained in legend to FIGURE S6 and Supplementary Information FIGURE S1. Normotensive rats were anesthetized with Zoletil/xylazine.

**
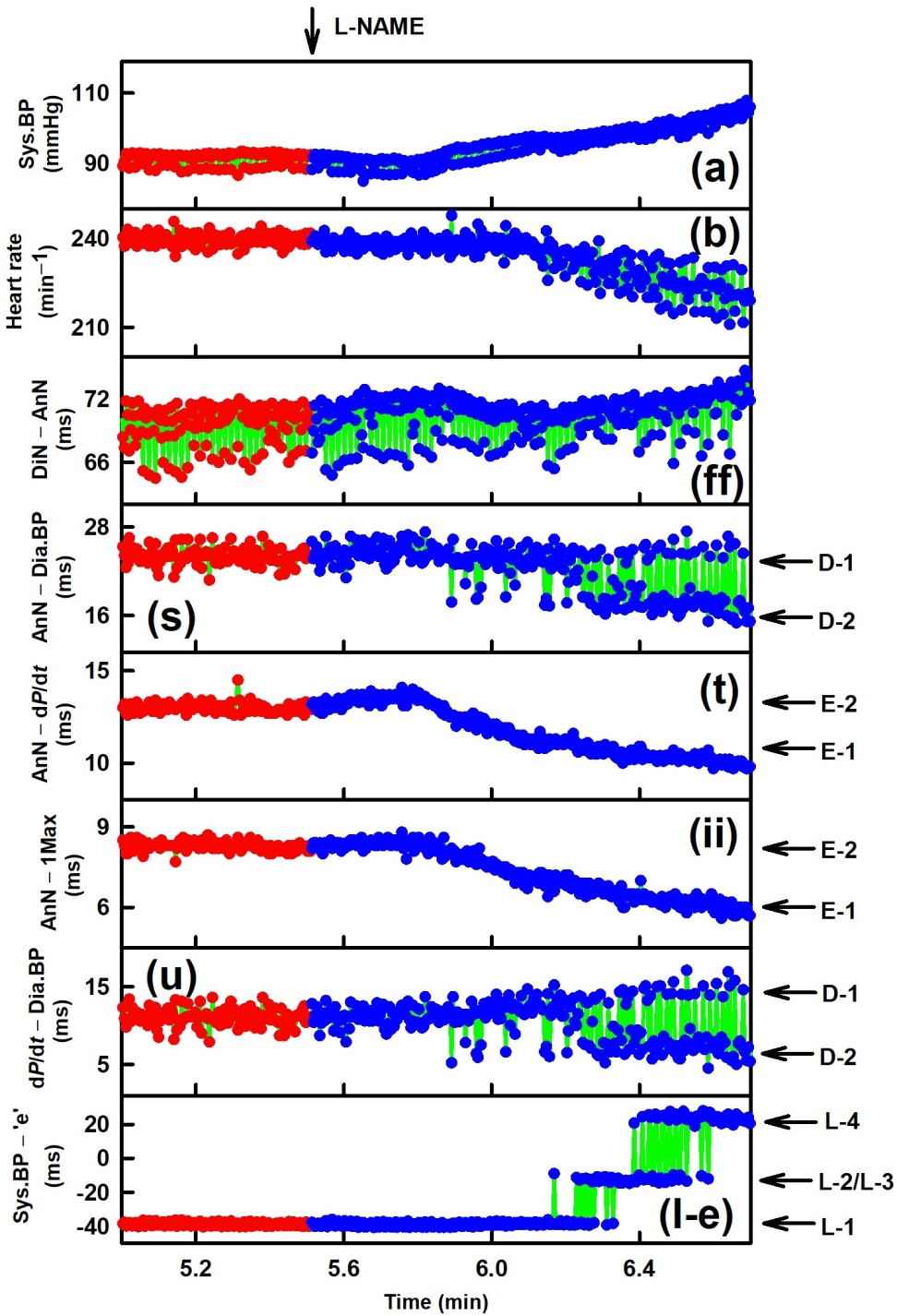
**

FIGURE S22 Exp-5. Time-dependent changes in APW-Ps of anesthetized rat: control (red heartbeats) and after i.v. administration of 15 mg kg^–1^ L-NAME (blue heartbeats) Horizontal arrows indicate predicted D-1 and D-2 levels, E-1 and E-2 levels and L-1 to L-4 levels. The green lines show the connection between adjacent heartbeats. Definitions, units and abbreviations of APW-Ps evaluated from the APW are as explained in legend to FIGURE S6 and Supplementary Information FIGURE S1. Normotensive rats were anesthetized with Zoletil/xylazine.

**
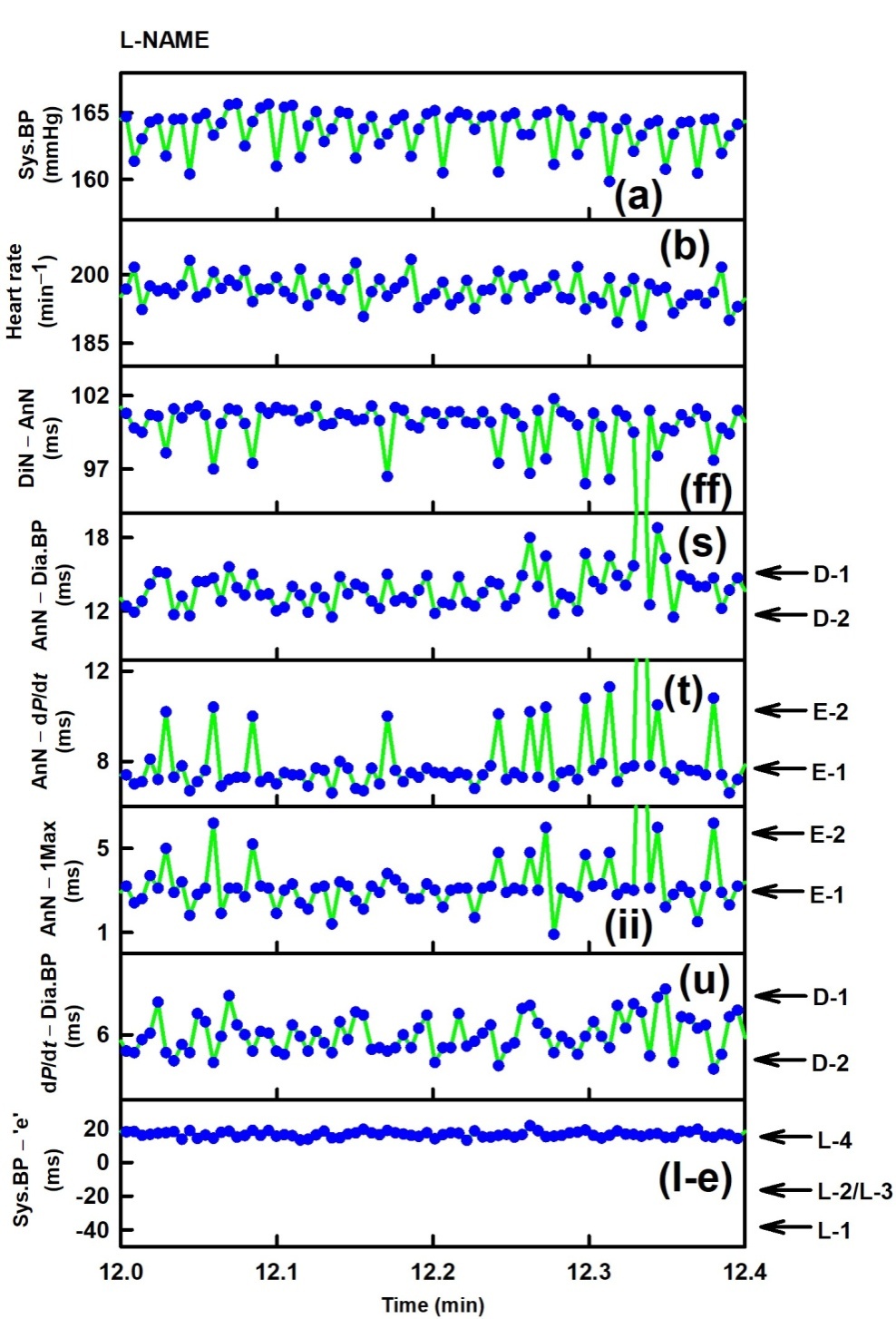
**

FIGURE S23 Exp-5. Time-dependent changes in APW-Ps of anesthetized rat after i.v. administration of 15 mg kg^–1^ L-NAME (blue heartbeats). Horizontal arrows indicate predicted D-1 and D-2 levels, E-1 and E-2 levels and L-1 to L-4 levels. The green lines show the connection between adjacent heartbeats. Definitions, units and abbreviations of APW-Ps evaluated from the APW are as explained in legend to FIGURE S6 and Supplementary Information FIGURE S1. Normotensive rats were anesthetized with Zoletil/xylazine.

**
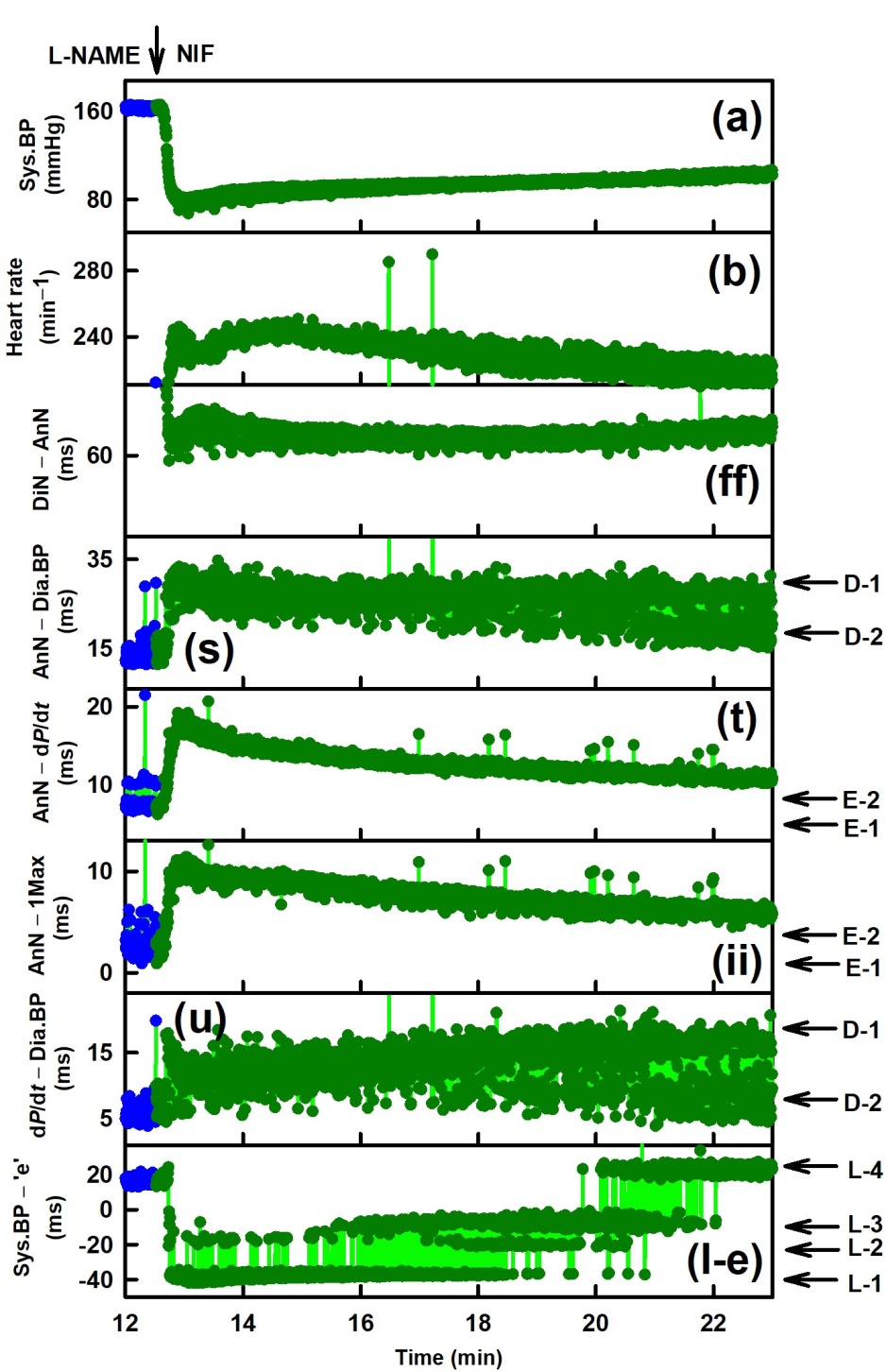
**

FIGURE S24 Exp-5. Time-dependent changes in APW-Ps of anesthetized rat after i.v. administration of 15 mg kg^–1^ L-NAME (blue heartbeats) and subsequent administration of 400 nmol kg^–1^ NIF (dark green heartbeats). Horizontal arrows indicate predicted D-1 and D-2 levels, E-1 and E-2 levels and L-1 to L-4 levels. The green lines show the connection between adjacent heartbeats. Definitions, units and abbreviations of APW-Ps evaluated from the APW are as explained in legend to FIGURE S6 and Supplementary Information FIGURE S1. Normotensive rats were anesthetized with Zoletil/xylazine.

**
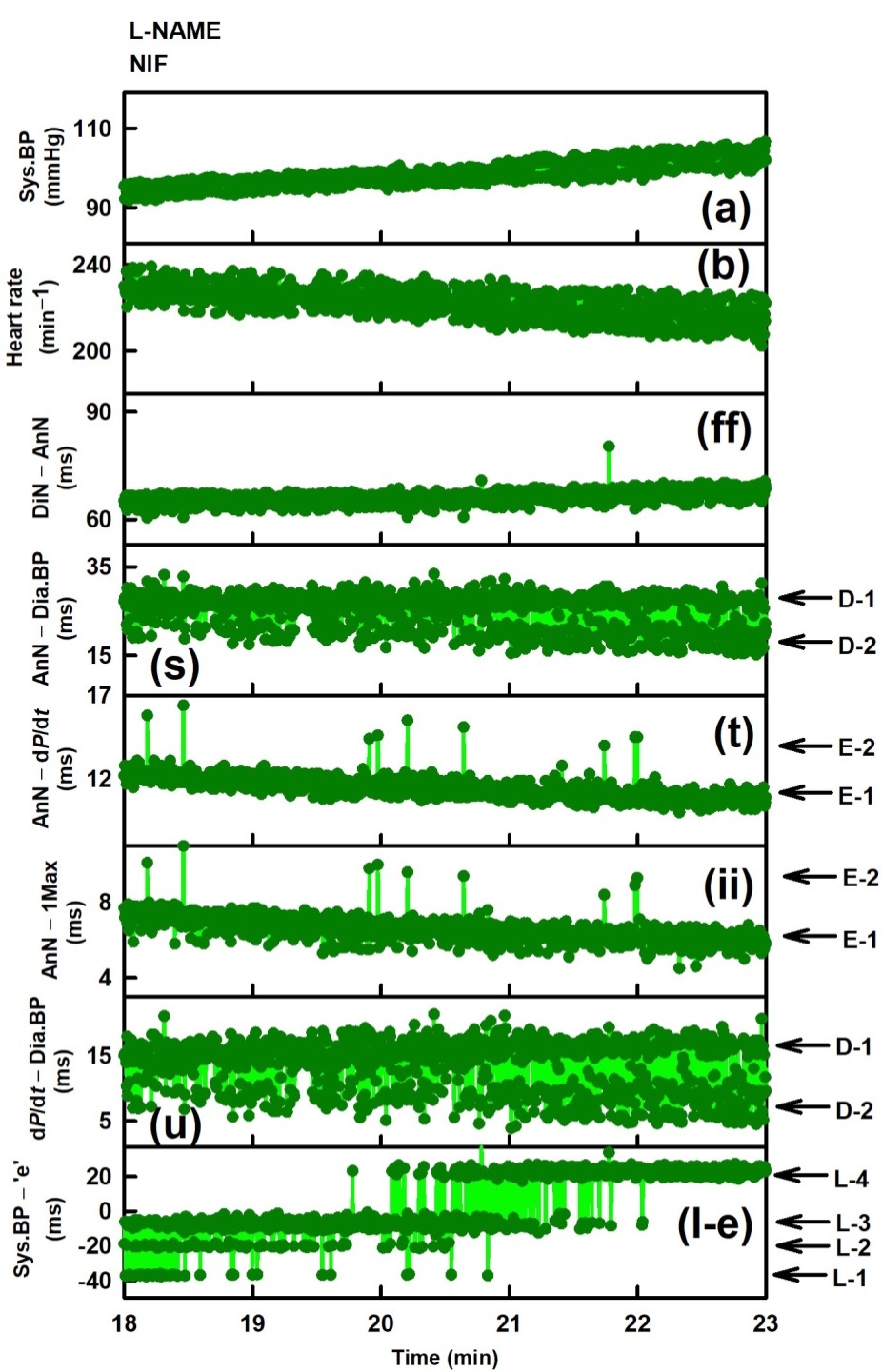
**

FIGURE S25 Exp-5. Time-dependent changes in APW-Ps of anesthetized rat in the presence of 15 mg kg^–1^ L-NAME (blue heartbeats) and subsequent administration of 400 nmol kg^–1^ NIF (dark green heartbeats). Horizontal arrows indicate predicted D-1 and D-2 levels, E-1 and E-2 levels and L-1 to L-4 levels. The green lines show the connection between adjacent heartbeats. Definitions, units and abbreviations of APW-Ps evaluated from the APW are as explained in legend to FIGURE S6 and Supplementary Information FIGURE S1. Normotensive rats were anesthetized with Zoletil/xylazine.


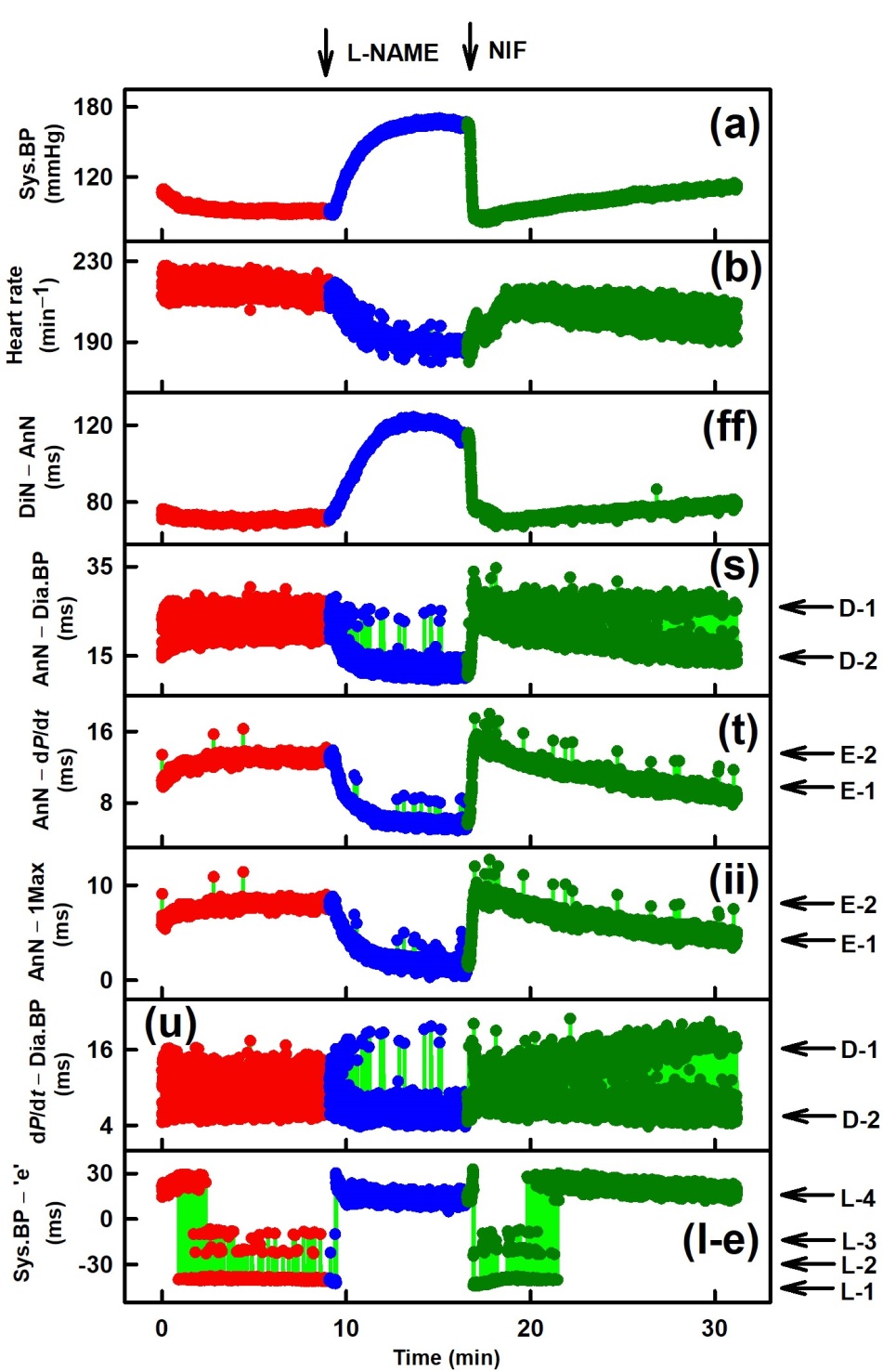


FIGURE S26 Exp-6. Time-dependent changes in APW-Ps of anesthetized rat: control (red heartbeats), after i.v. administration of 15 mg kg^–1^ L-NAME (blue heartbeats) and 400 nmol kg^–1^ NIF (dark green heartbeats). Horizontal arrows indicate predicted D-1 and D-2 levels, E-1 and E-2 levels and L-1 to L-4 levels. The green lines show the connection between adjacent heartbeats. Definitions, units and abbreviations of APW-Ps evaluated from the APW are as explained in legend to FIGURE S6 and Supplementary Information FIGURE S1. Normotensive rats were anesthetized with Zoletil/xylazine.

**
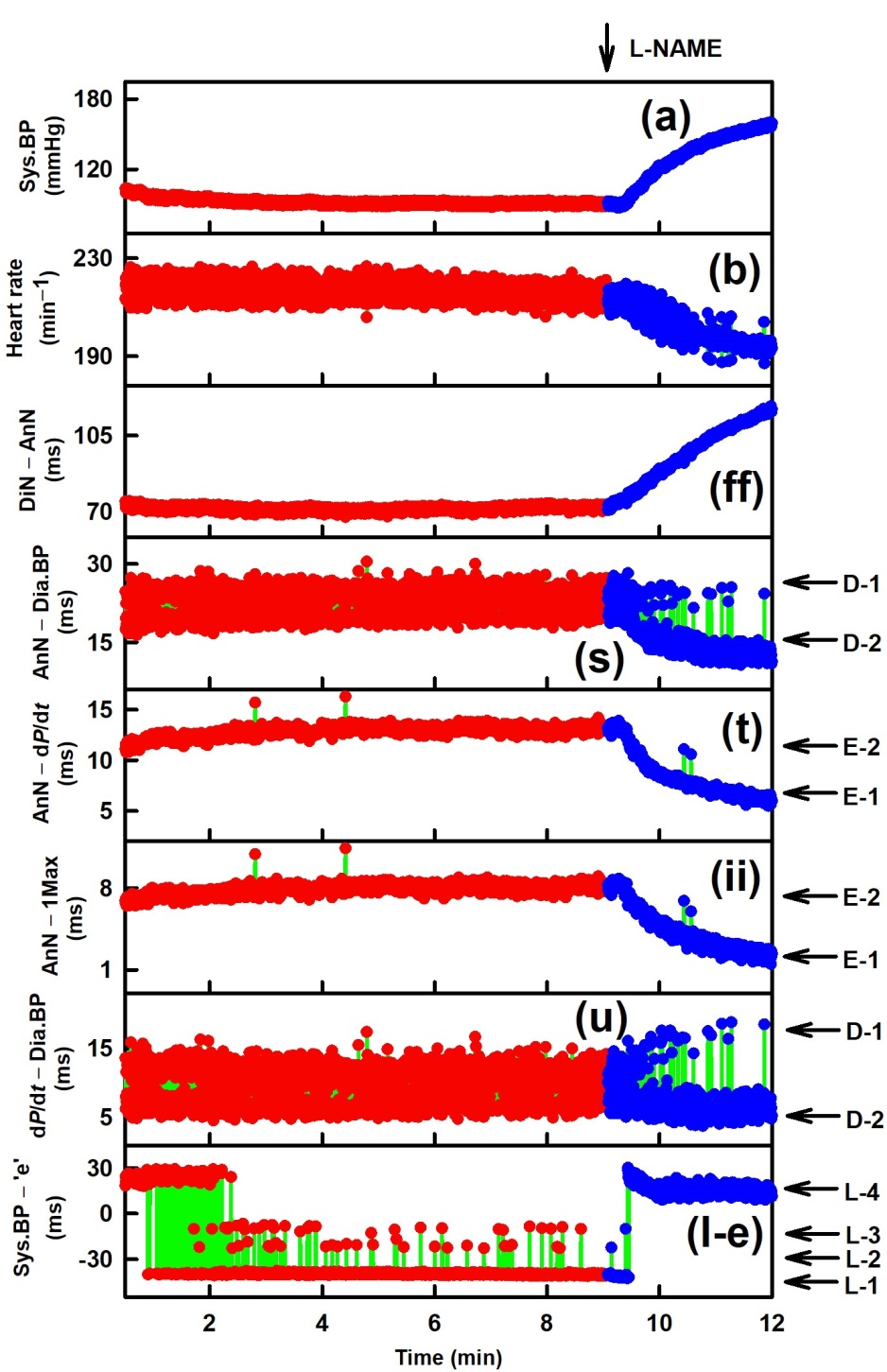
**

FIGURE S27 Exp-6. Time-dependent changes in APW-Ps of anesthetized rat: control (red heartbeats) and after i.v. administration of 15 mg kg^–1^ L-NAME (blue heartbeats) Horizontal arrows indicate predicted D-1 and D-2 levels, E-1 and E-2 levels and L-1 to L-4 levels. The green lines show the connection between adjacent heartbeats. Definitions, units and abbreviations of APW-Ps evaluated from the APW are as explained in legend to FIGURE S6 and Supplementary Information FIGURE S1. Normotensive rats were anesthetized with Zoletil/xylazine.

**
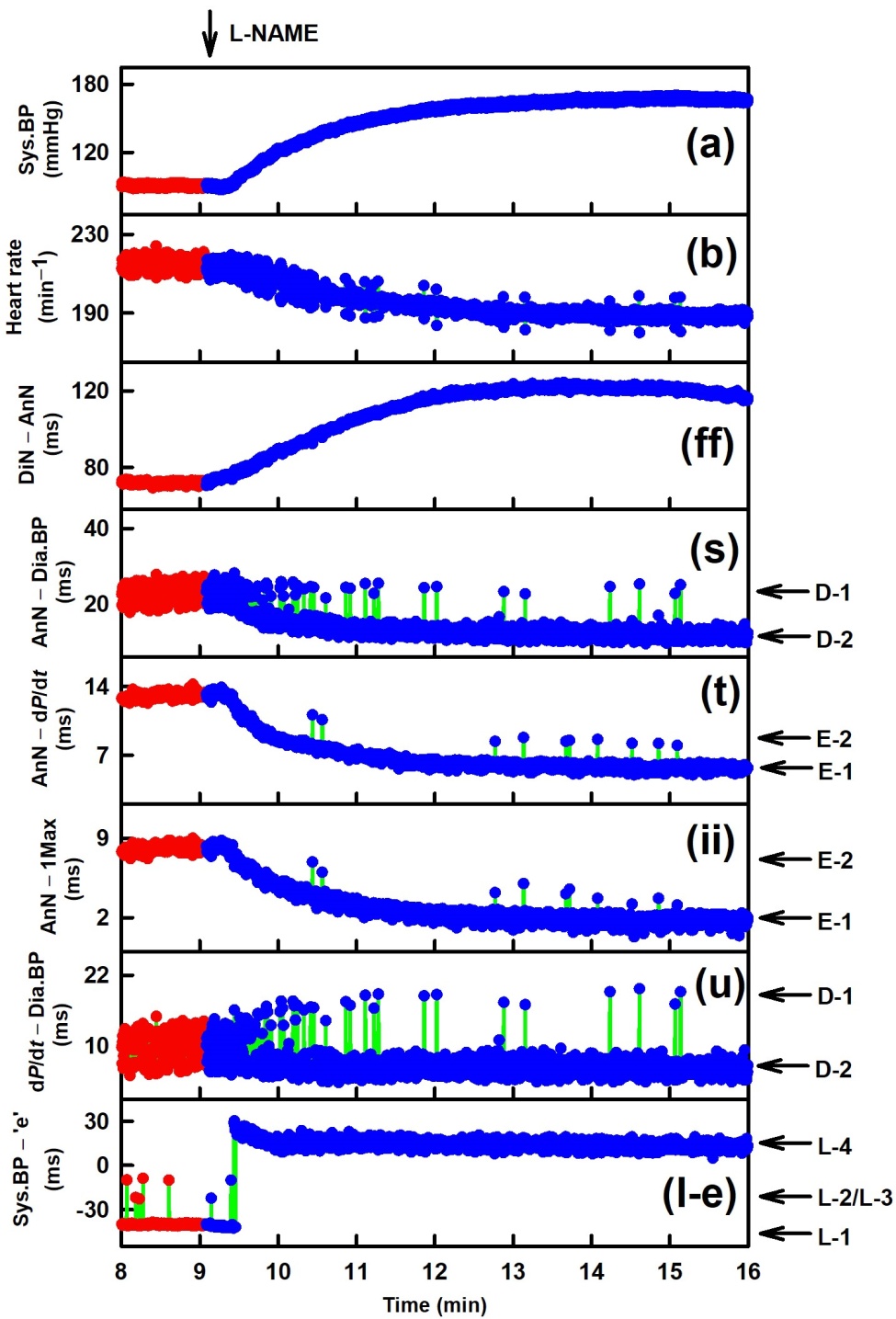
**

FIGURE S28 Exp-6. Time-dependent changes in APW-Ps of anesthetized rat: control (red heartbeats) and after i.v. administration of 15 mg kg^–1^ L-NAME (blue heartbeats) Horizontal arrows indicate predicted D-1 and D-2 levels, E-1 and E-2 levels and L-1 to L-4 levels. The green lines show the connection between adjacent heartbeats. Definitions, units and abbreviations of APW-Ps evaluated from the APW are as explained in legend to FIGURE S6 and Supplementary Information FIGURE S1. Normotensive rats were anesthetized with Zoletil/xylazine.

**
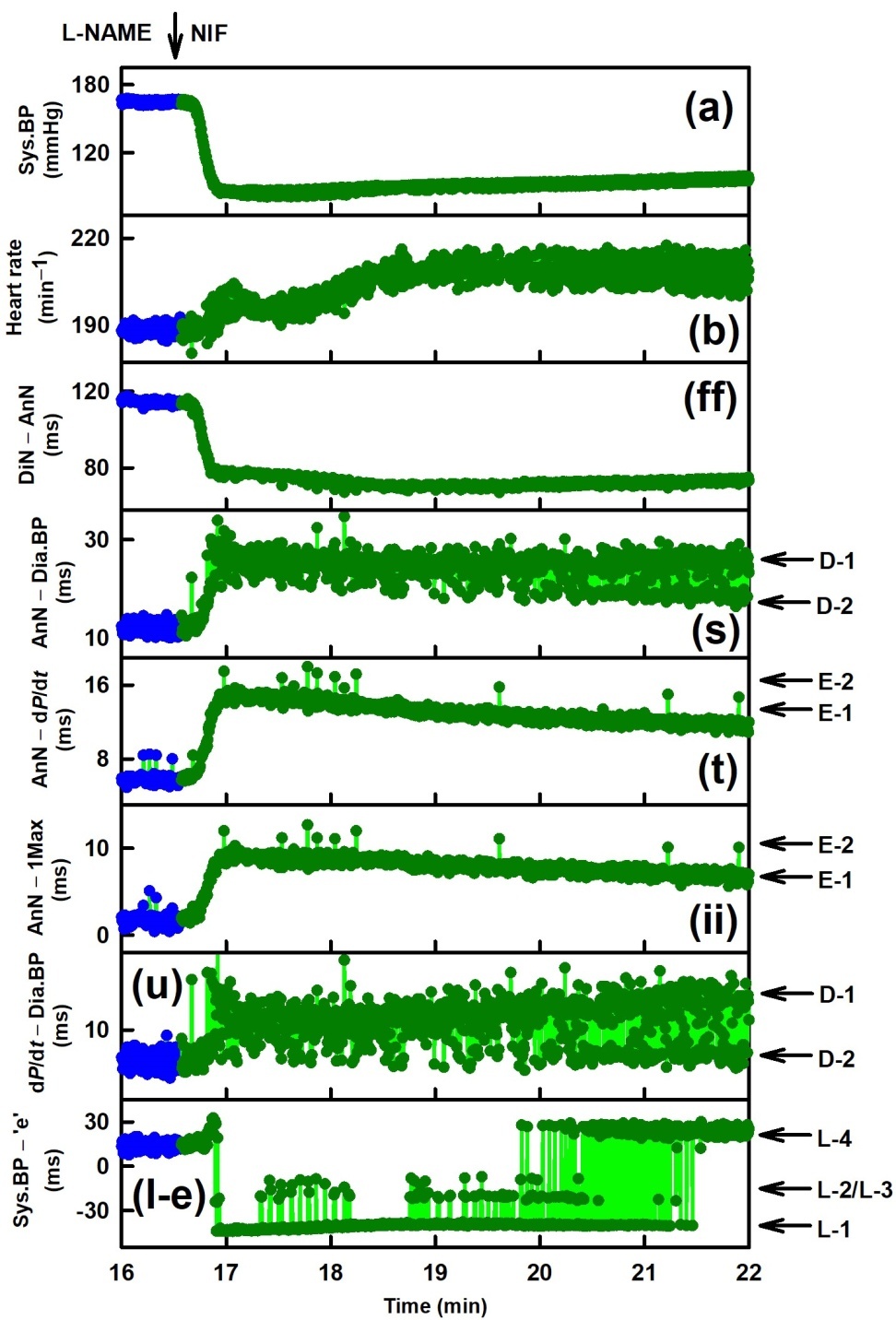
**

FIGURE S29 Exp-6. Time-dependent changes in APW-Ps of anesthetized rat after i.v. administration of 15 mg kg^–1^ L-NAME (blue heartbeats) and subsequent administration of 400 nmol kg^–1^ NIF (dark green heartbeats). Horizontal arrows indicate predicted D-1 and D-2 levels, E-1 and E-2 levels and L-1 to L-4 levels. The green lines show the connection between adjacent heartbeats. Definitions, units and abbreviations of APW-Ps evaluated from the APW are as explained in legend to FIGURE S6 and Supplementary Information FIGURE S1. Normotensive rats were anesthetized with Zoletil/xylazine.

**
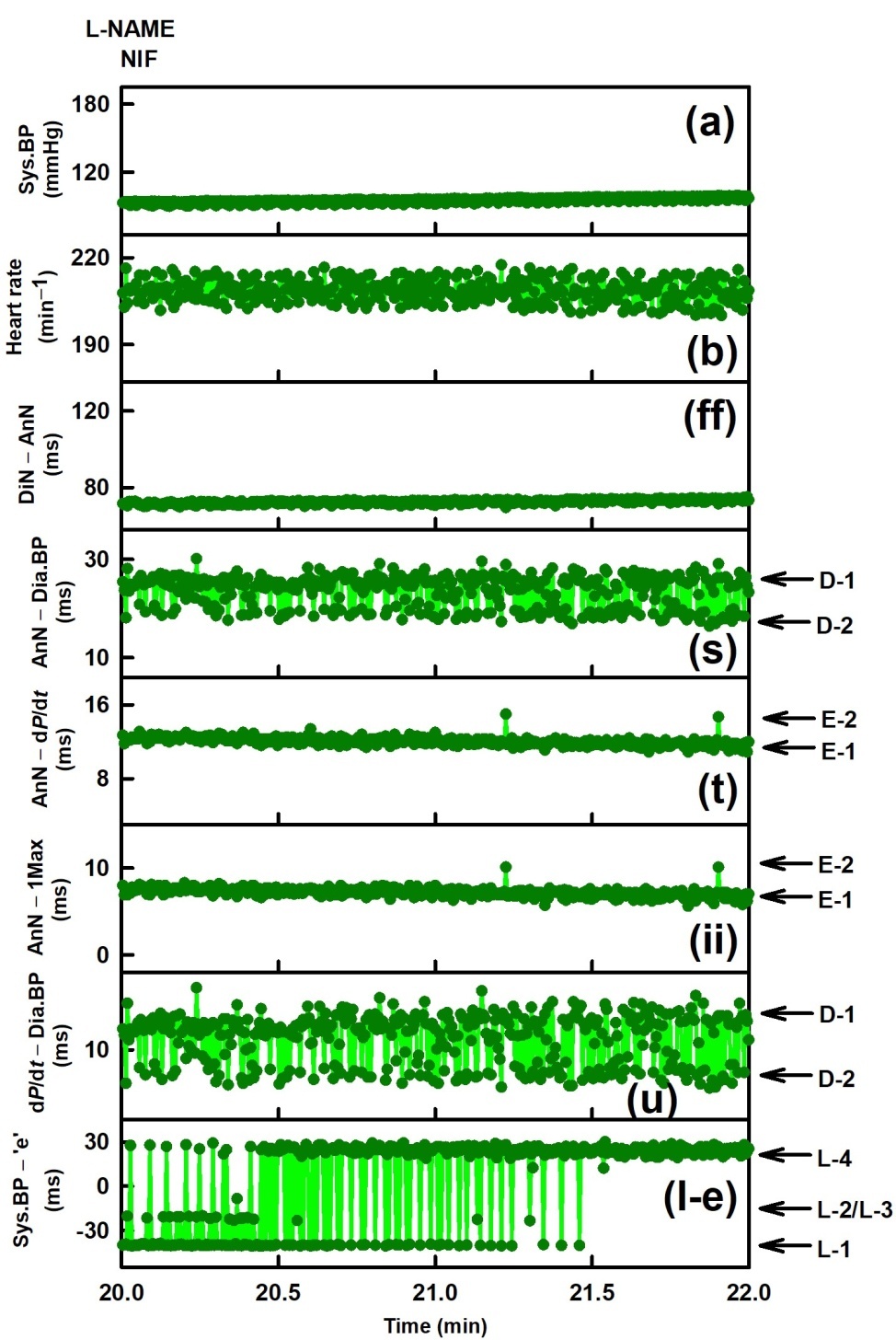
**

FIGURE S30 Exp-6. Time-dependent changes in APW-Ps of anesthetized rat in the presence of 15 mg kg^–1^ L-NAME (blue heartbeats) and subsequent administration of 400 nmol kg^–1^ NIF (dark green heartbeats). Horizontal arrows indicate predicted D-1 and D-2 levels, E-1 and E-2 levels and L-1 to L-4 levels. The green lines show the connection between adjacent heartbeats. Definitions, units and abbreviations of APW-Ps evaluated from the APW are as explained in legend to FIGURE S6 and Supplementary Information FIGURE S1. Normotensive rats were anesthetized with Zoletil/xylazine.

**
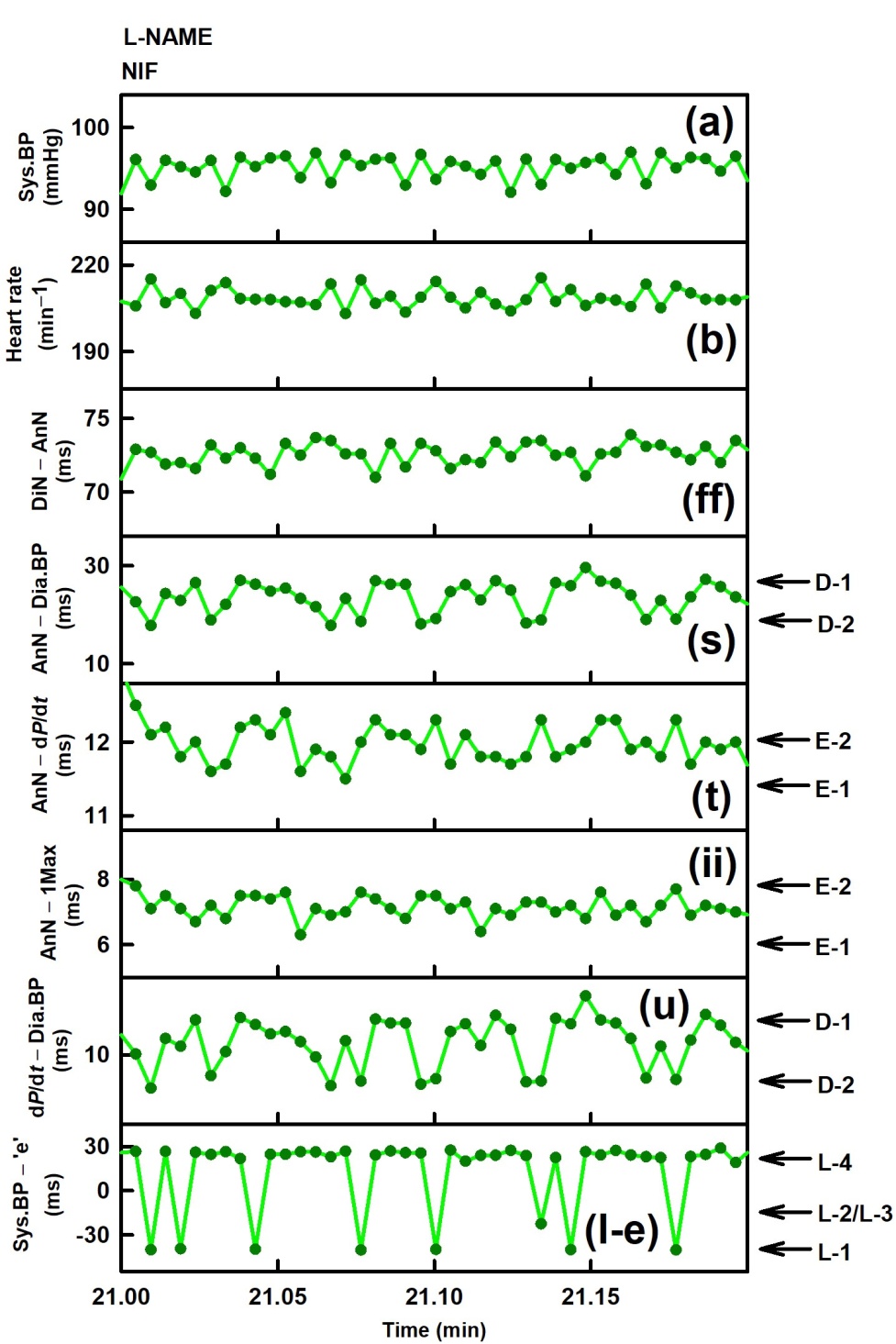
**

FIGURE S31 Exp-6. Details of the time-dependent changes in APW-Ps of anesthetized rat in the presence of 15 mg kg^–1^ L-NAME and subsequent administration of 400 nmol kg^–1^ NIF (dark green heartbeats). Horizontal arrows indicate predicted D-1 and D-2 levels, E-1 and E-2 levels and L-1 to L-4 levels. The green lines show the connection between adjacent heartbeats. Definitions, units and abbreviations of APW-Ps evaluated from the APW are as explained in legend to FIGURE S6 and Supplementary Information FIGURE S1. Normotensive rats were anesthetized with Zoletil/xylazine.


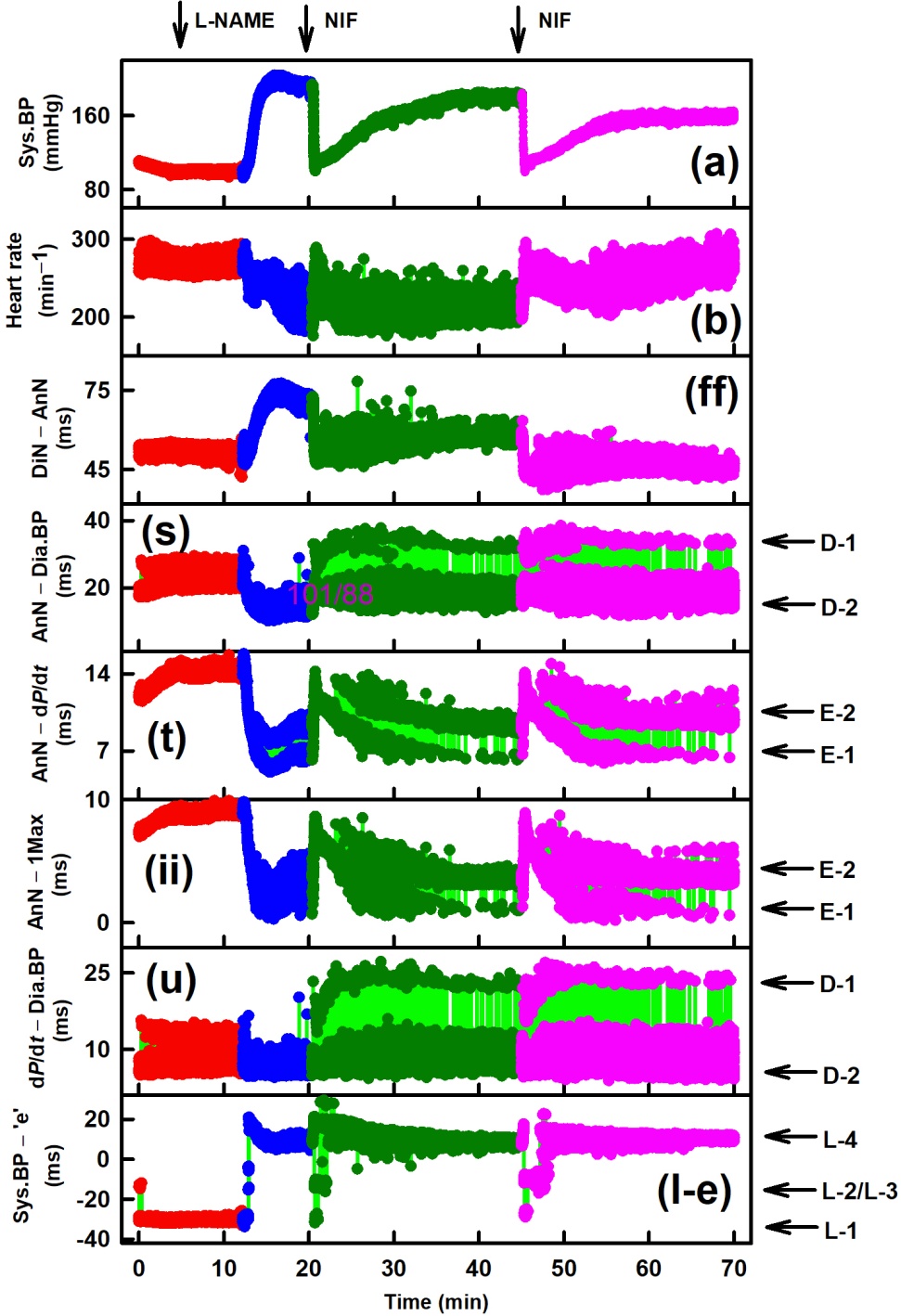


FIGURE S32 Exp-7. Time-dependent changes in APW-Ps of anesthetized rat: control (red heartbeats), after i.v. administration of 15 mg kg^–1^ L-NAME (blue heartbeats) and 400 nmol kg^–1^ NIF (dark green heartbeats) and the second administration of 400 nmol kg^–1^ NIF (pink heartbeats). Horizontal arrows indicate predicted D-1 and D-2 levels, E-1 and E-2 levels and L-1 to L-4 levels. The green lines show the connection between adjacent heartbeats. Definitions, units and abbreviations of APW-Ps evaluated from the APW are as explained in legend to FIGURE S6 and Supplementary Information FIGURE S1. Normotensive rats were anesthetized with Zoletil/xylazine.


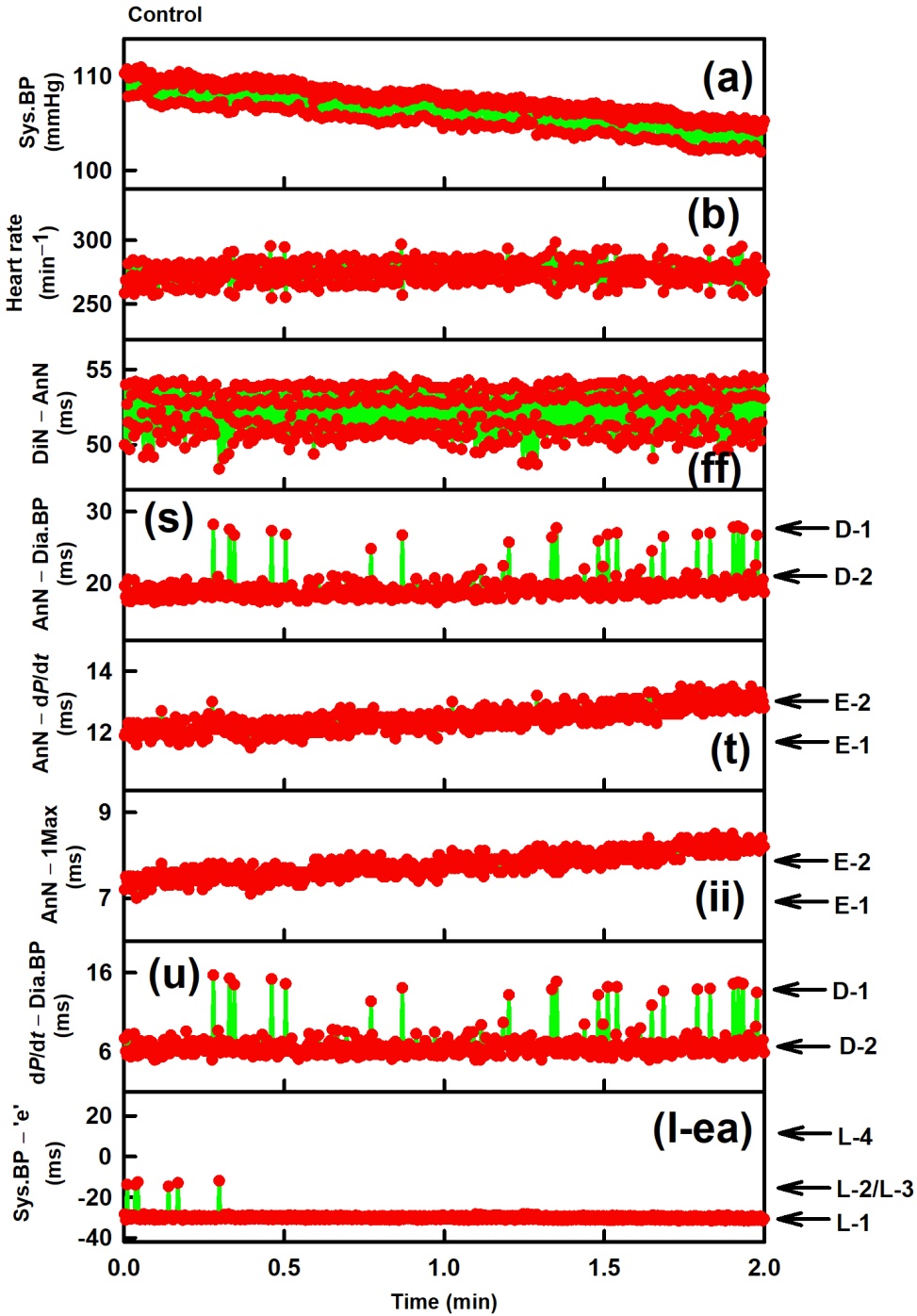


FIGURE S33 Exp-7. Time-dependent changes in APW-Ps of anesthetized rat: control (red heartbeats). Horizontal arrows indicate predicted D-1 and D-2 levels, E-1 and E-2 levels and L-1 to L-4 levels. The green lines show the connection between adjacent heartbeats. Definitions, units and abbreviations of APW-Ps evaluated from the APW are as explained in legend to FIGURE S6 and Supplementary Information FIGURE S1. Normotensive rats were anesthetized with Zoletil/xylazine.


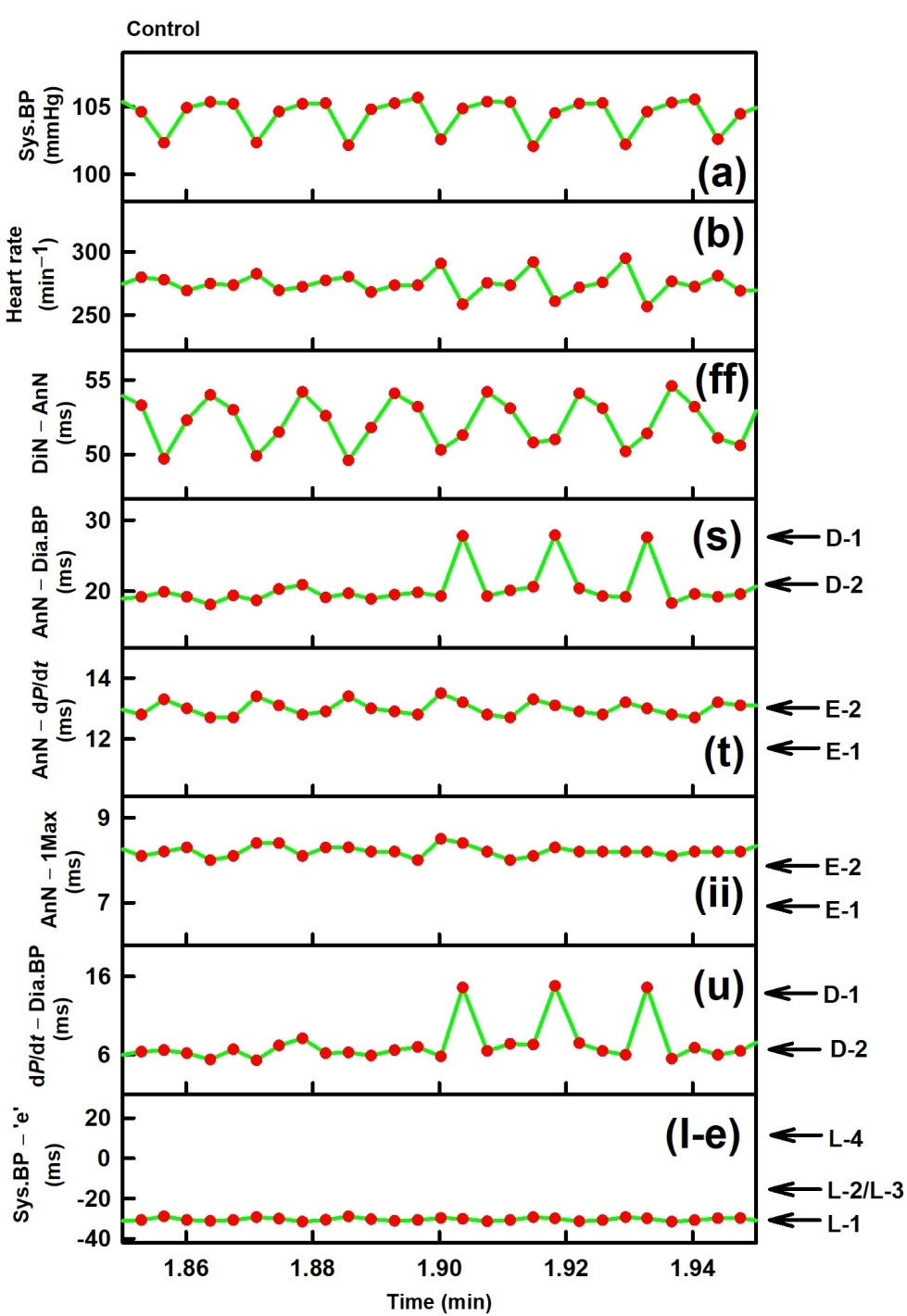


FIGURE S34 Exp-7. Details of the time-dependent changes in APW-Ps of anesthetized rat: control (red heartbeats). Horizontal arrows indicate predicted D-1 and D-2 levels, E-1 and E-2 levels and L-1 to L-4 levels. The green lines show the connection between adjacent heartbeats. Definitions, units and abbreviations of APW-Ps evaluated from the APW are as explained in legend to FIGURE S6 and Supplementary Information FIGURE S1. Normotensive rats were anesthetized with Zoletil/xylazine.


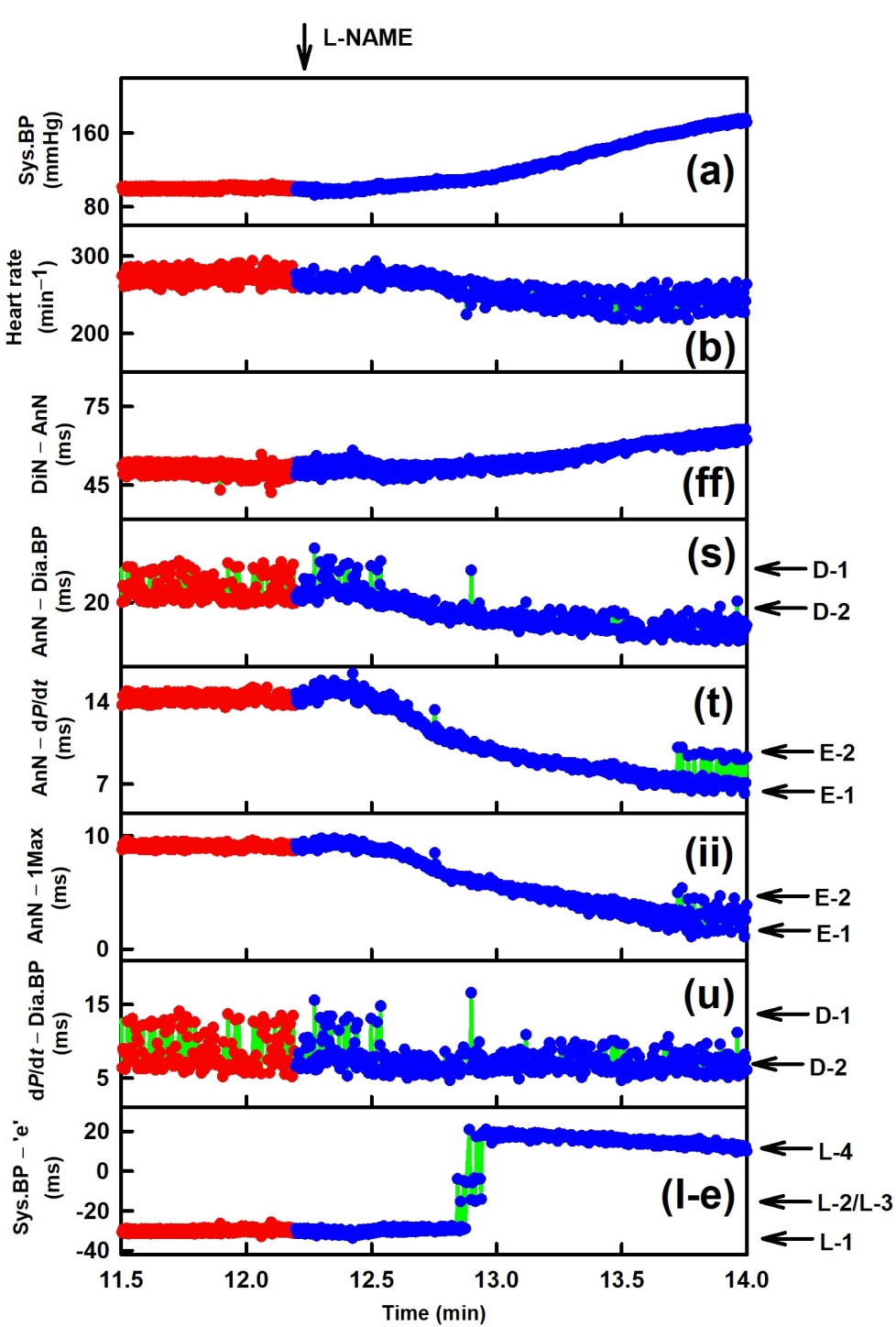


FIGURE S35 Exp-7. Time-dependent changes in APW-Ps of anesthetized rat: control (red heartbeats) and after i.v. administration of 15 mg kg^–1^ L-NAME (blue heartbeats) Horizontal arrows indicate predicted D-1 and D-2 levels, E-1 and E-2 levels and L-1 to L-4 levels. The green lines show the connection between adjacent heartbeats. Definitions, units and abbreviations of APW-Ps evaluated from the APW are as explained in legend to FIGURE S6 and Supplementary Information FIGURE S1. Normotensive rats were anesthetized with Zoletil/xylazine.


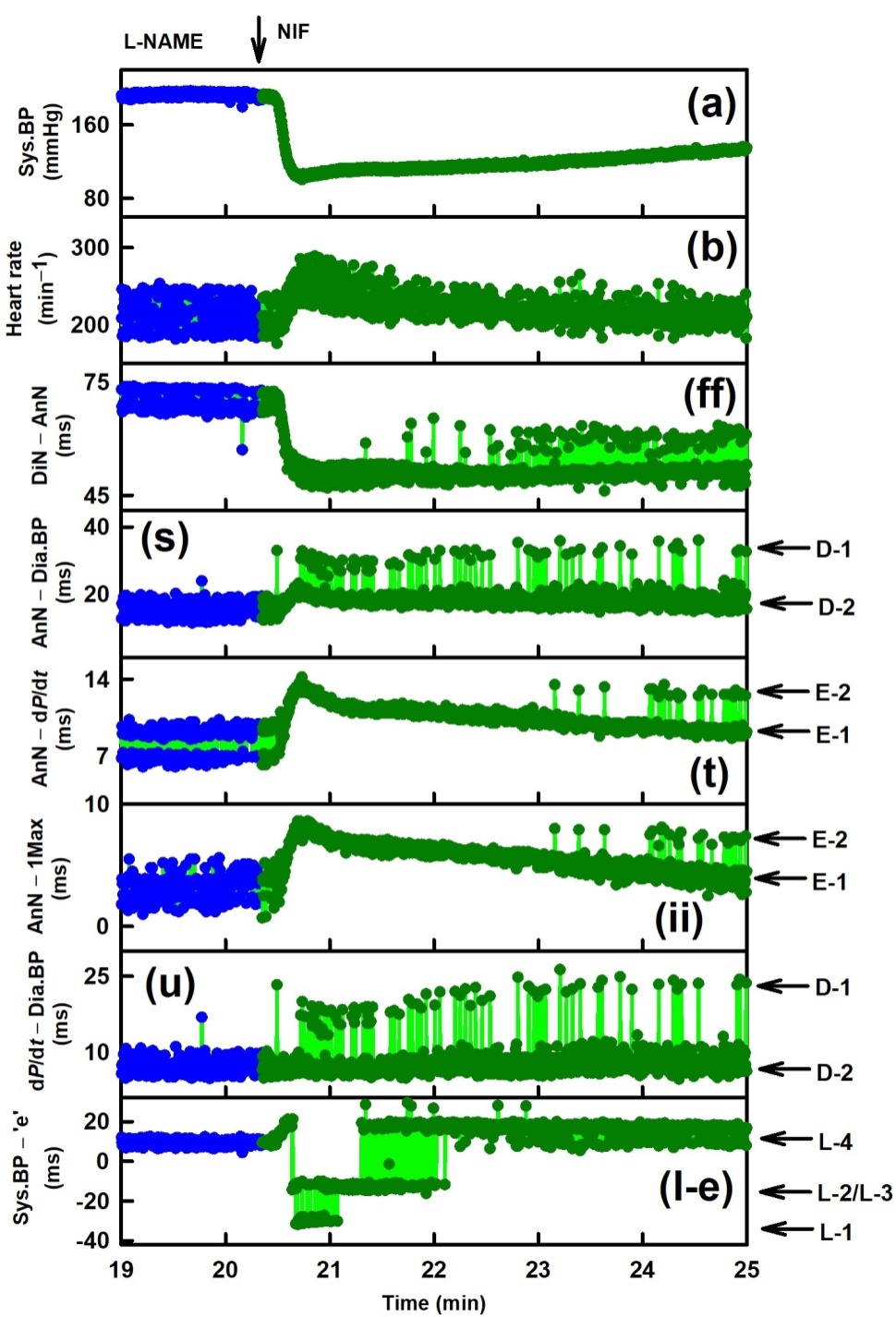


FIGURE S36 Exp-7. Time-dependent changes in APW-Ps of anesthetized rat after i.v. administration of 15 mg kg^–1^ L-NAME (blue heartbeats) and subsequent administration of 400 nmol kg^–1^ NIF (dark green heartbeats). Horizontal arrows indicate predicted D-1 and D-2 levels, E-1 and E-2 levels and L-1 to L-4 levels. The green lines show the connection between adjacent heartbeats. Definitions, units and abbreviations of APW-Ps evaluated from the APW are as explained in legend to FIGURE S6 and Supplementary Information FIGURE S1. Normotensive rats were anesthetized with Zoletil/xylazine.


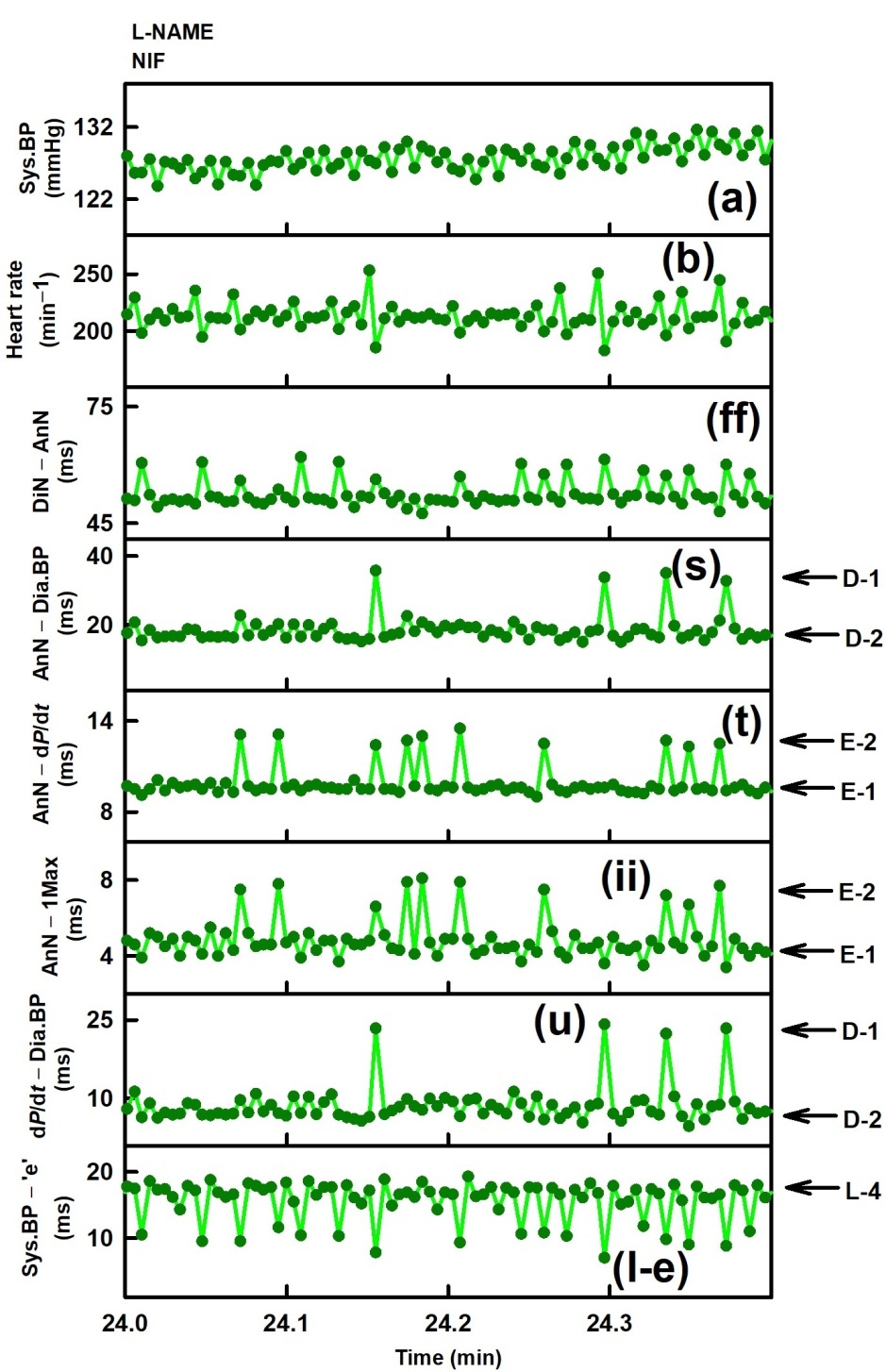


FIGURE S37 Exp-7. Details of the time-dependent changes in APW-Ps of anesthetized rat in the presence of 15 mg kg^–1^ L-NAME and subsequent administration of 400 nmol kg^–1^ NIF (dark green heartbeats). Horizontal arrows indicate predicted D-1 and D-2 levels, E-1 and E-2 levels and L-1 to L-4 levels. The green lines show the connection between adjacent heartbeats. Definitions, units and abbreviations of APW-Ps evaluated from the APW are as explained in legend to FIGURE S6 and Supplementary Information FIGURE S1. Normotensive rats were anesthetized with Zoletil/xylazine.


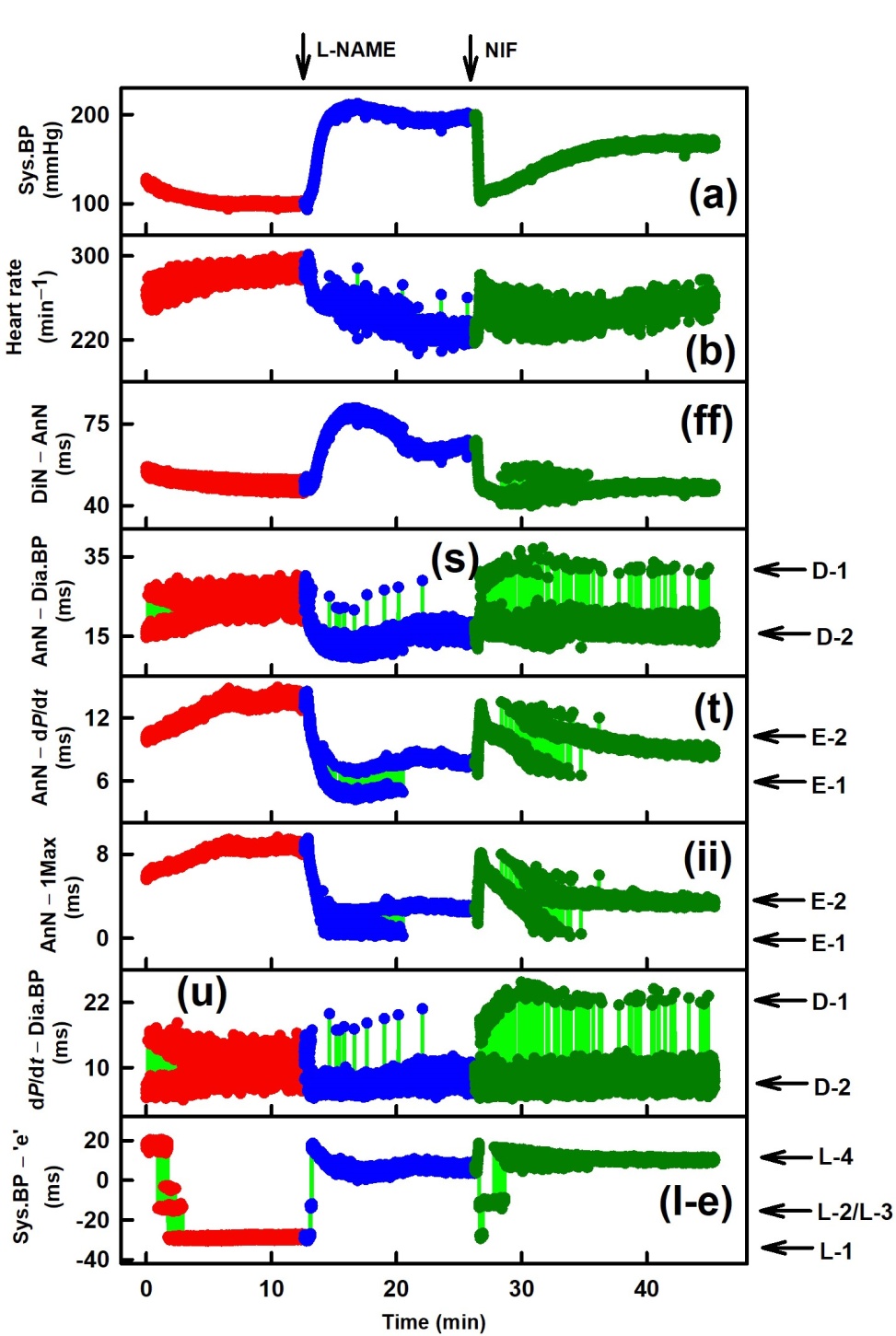


FIGURE S38 Exp-8. Time-dependent changes in APW-Ps of anesthetized rat: control (red heartbeats), after i.v. administration of 15 mg kg^–1^ L-NAME (blue heartbeats) and 400 nmol kg^–1^ NIF (dark green heartbeats). Horizontal arrows indicate predicted D-1 and D-2 levels, E-1 and E-2 levels and L-1 to L-4 levels. The green lines show the connection between adjacent heartbeats. Definitions, units and abbreviations of APW-Ps evaluated from the APW are as explained in legend to FIGURE S6 and Supplementary Information FIGURE S1. Normotensive rats were anesthetized with Zoletil/xylazine.


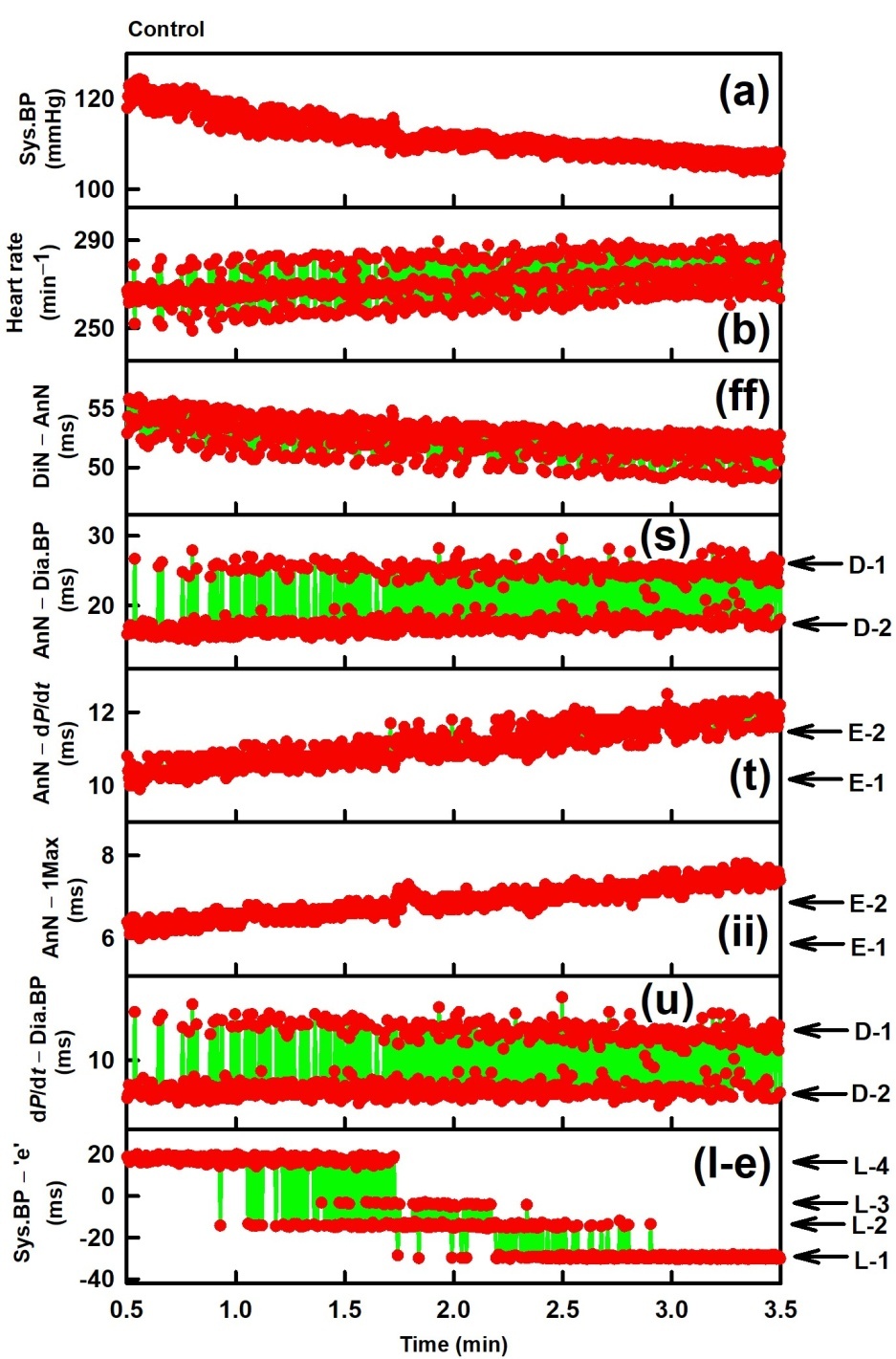


FIGURE S39 Exp-8. Time-dependent changes in APW-Ps of anesthetized rat: control (red heartbeats). Horizontal arrows indicate predicted D-1 and D-2 levels, E-1 and E-2 levels and L-1 to L-4 levels. The green lines show the connection between adjacent heartbeats. Definitions, units and abbreviations of APW-Ps evaluated from the APW are as explained in legend to FIGURE S6 and Supplementary Information FIGURE S1. Normotensive rats were anesthetized with Zoletil/xylazine.


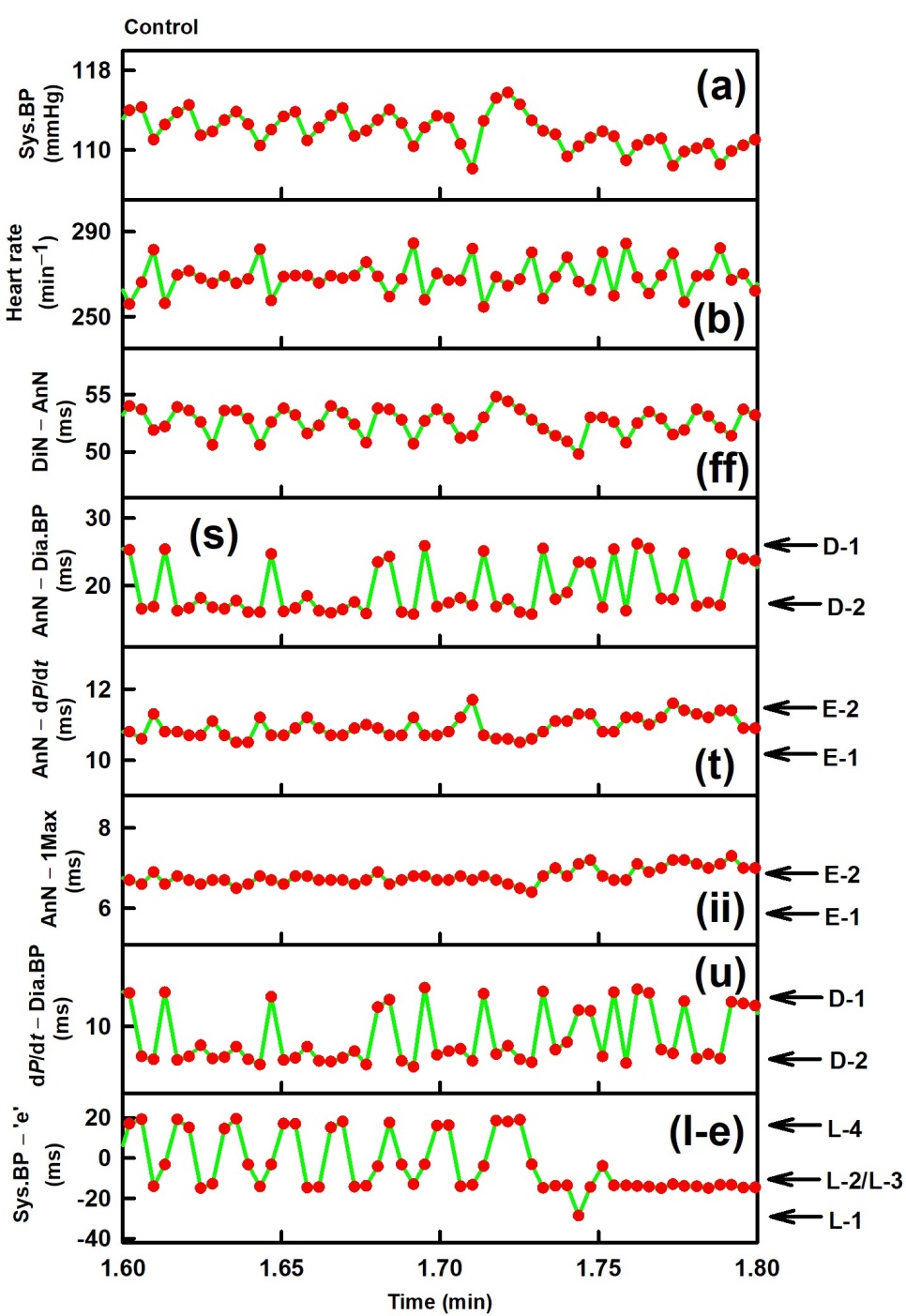


FIGURE S40 Exp-8. Details of the time-dependent changes in APW-Ps of anesthetized rat: control (red heartbeats). Horizontal arrows indicate predicted D-1 and D-2 levels, E-1 and E-2 levels and L-1 to L-4 levels. The green lines show the connection between adjacent heartbeats. Definitions, units and abbreviations of APW-Ps evaluated from the APW are as explained in legend to FIGURE S6 and Supplementary Information FIGURE S1. Normotensive rats were anesthetized with Zoletil/xylazine.


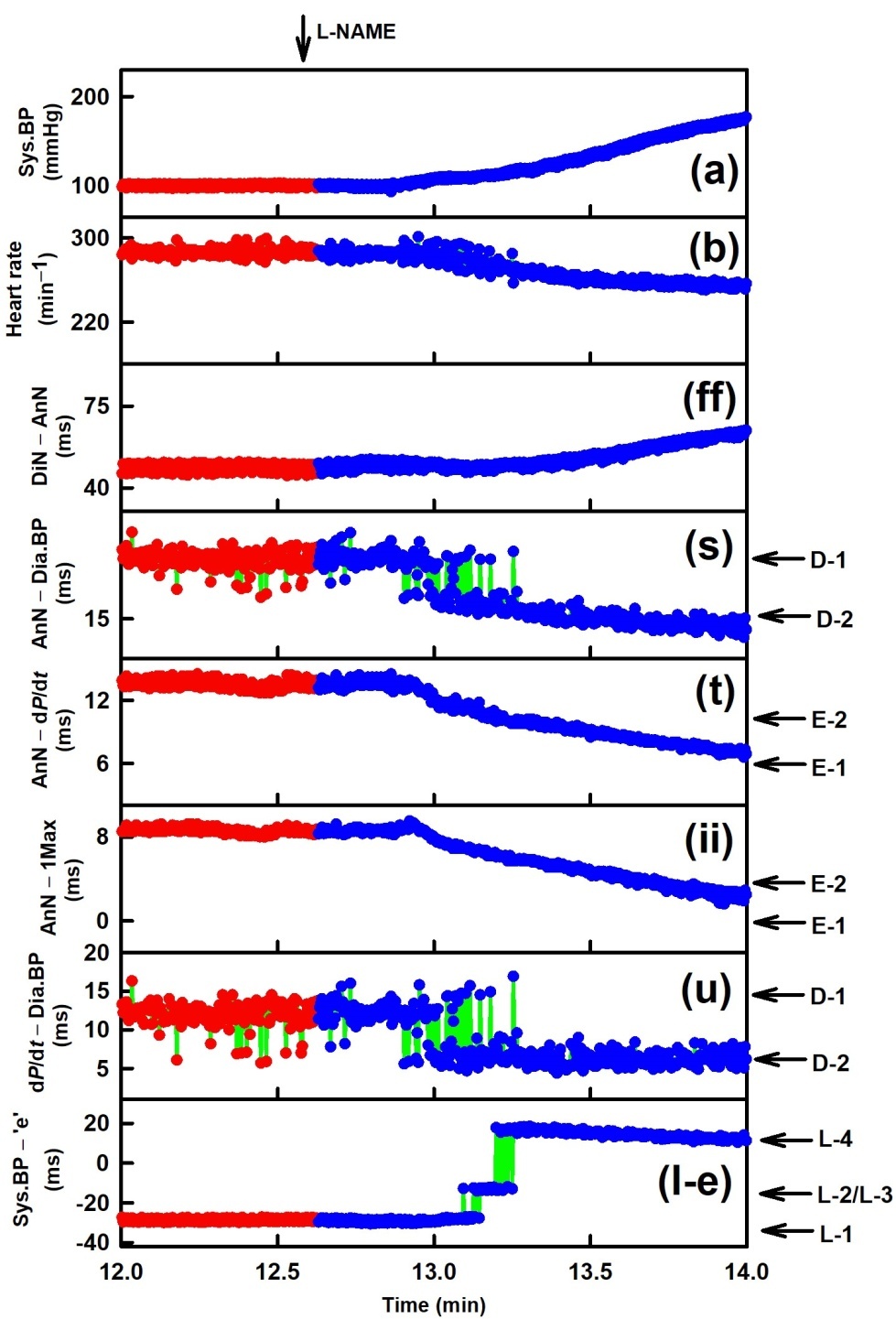


FIGURE S41 Exp-8. Time-dependent changes in APW-Ps of anesthetized rat: control (red heartbeats) and after i.v. administration of 15 mg kg^–1^ L-NAME (blue heartbeats) Horizontal arrows indicate predicted D-1 and D-2 levels, E-1 and E-2 levels and L-1 to L-4 levels. The green lines show the connection between adjacent heartbeats. Definitions, units and abbreviations of APW-Ps evaluated from the APW are as explained in legend to FIGURE S6 and Supplementary Information FIGURE S1. Normotensive rats were anesthetized with Zoletil/xylazine.


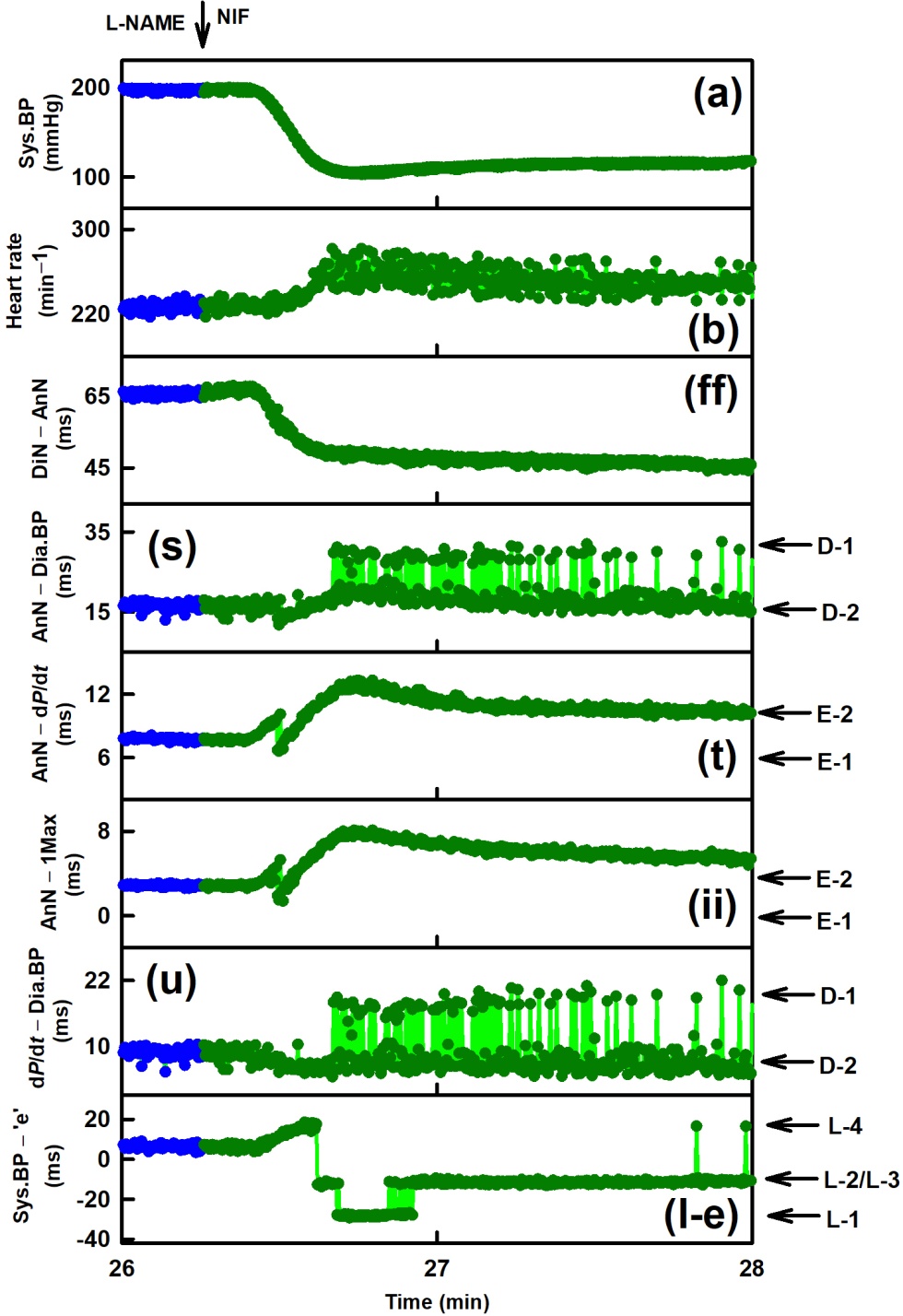


FIGURE S42 Exp-8. Time-dependent changes in APW-Ps of anesthetized rat after i.v. administration of 15 mg kg^–1^ L-NAME (blue heartbeats) and subsequent administration of 400 nmol kg^–1^ NIF (dark green heartbeats). Horizontal arrows indicate predicted D-1 and D-2 levels, E-1 and E-2 levels and L-1 to L-4 levels. The green lines show the connection between adjacent heartbeats. Definitions, units and abbreviations of APW-Ps evaluated from the APW are as explained in legend to FIGURE S6 and Supplementary Information FIGURE S1. Normotensive rats were anesthetized with Zoletil/xylazine.


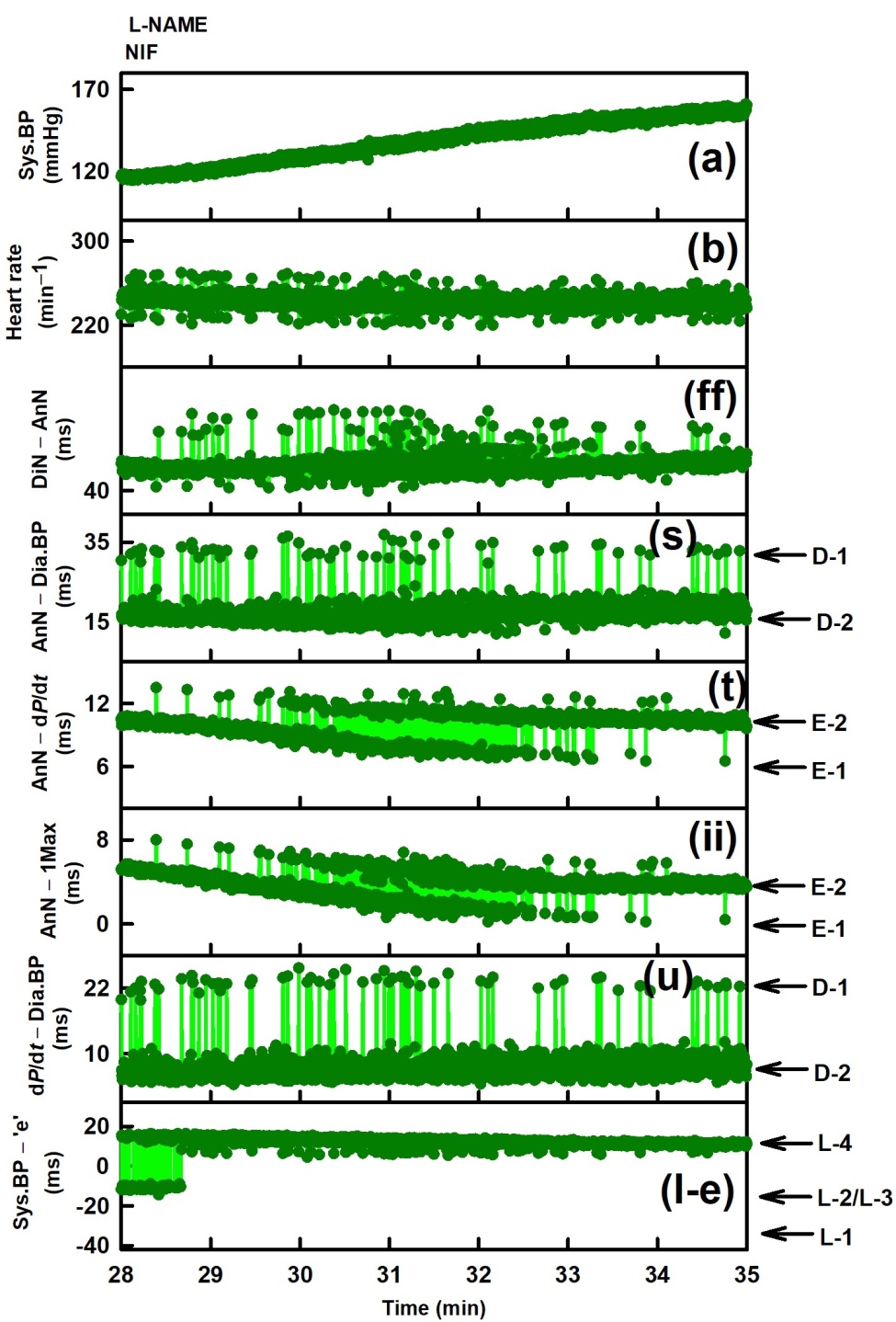


FIGURE S43 Exp-8. Time-dependent changes in APW-Ps of anesthetized rat in the presence of 15 mg kg^–1^ L-NAME (blue heartbeats) and subsequent administration of 400 nmol kg^–1^ NIF (dark green heartbeats). Horizontal arrows indicate predicted D-1 and D-2 levels, E-1 and E-2 levels and L-1 to L-4 levels. The green lines show the connection between adjacent heartbeats. Definitions, units and abbreviations of APW-Ps evaluated from the APW are as explained in legend to FIGURE S6 and Supplementary Information FIGURE S1. Normotensive rats were anesthetized with Zoletil/xylazine.


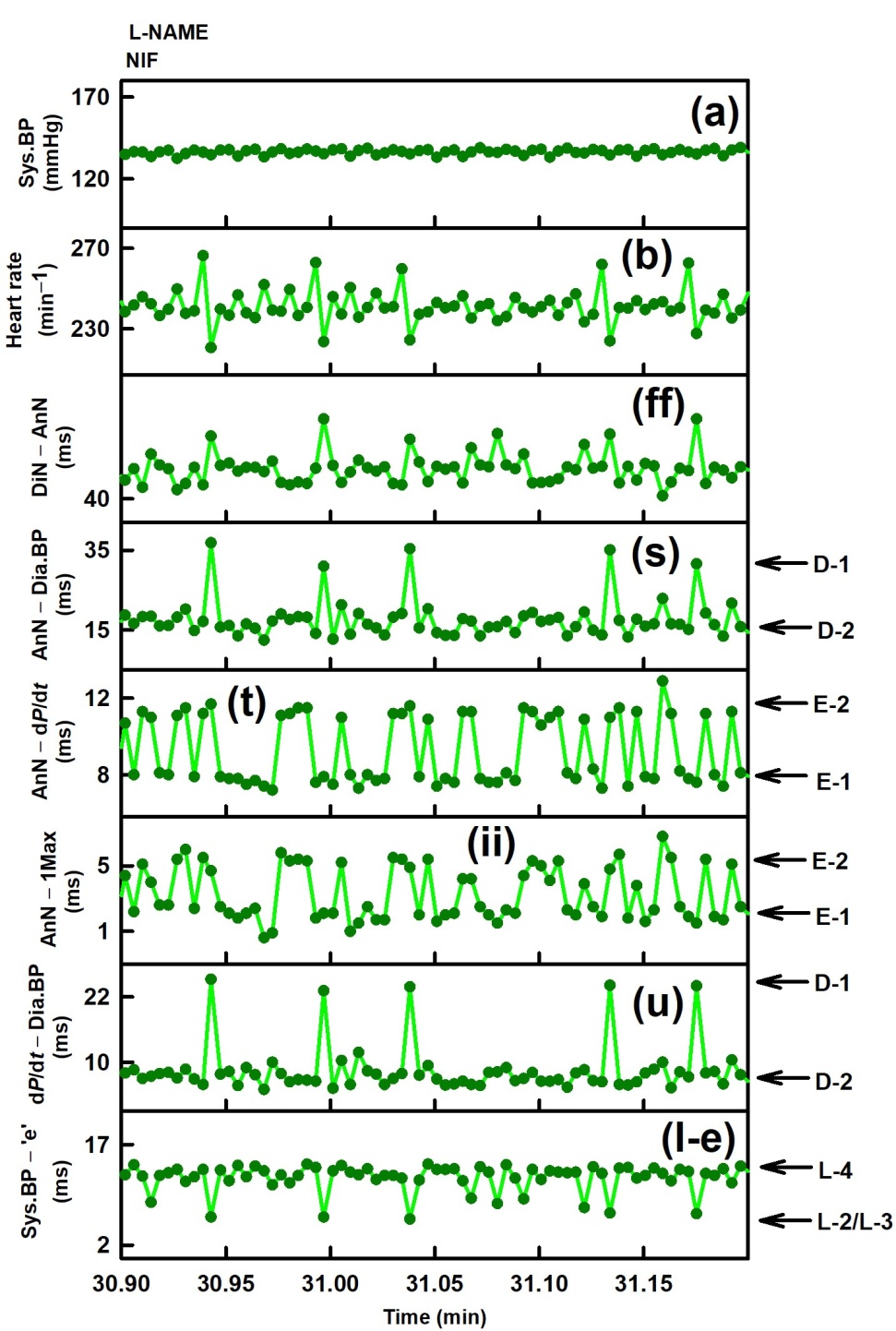


FIGURE S44 Exp-8. Details of the time-dependent changes in APW-Ps of anesthetized rat in the presence of 15 mg kg^–1^ L-NAME and subsequent administration of 400 nmol kg^–1^ NIF (dark green heartbeats). Horizontal arrows indicate predicted D-1 and D-2 levels, E-1 and E-2 levels and L-1 to L-4 levels. The green lines show the connection between adjacent heartbeats. Definitions, units and abbreviations of APW-Ps evaluated from the APW are as explained in legend to FIGURE S6 and Supplementary Information FIGURE S1. Normotensive rats were anesthetized with Zoletil/xylazine.


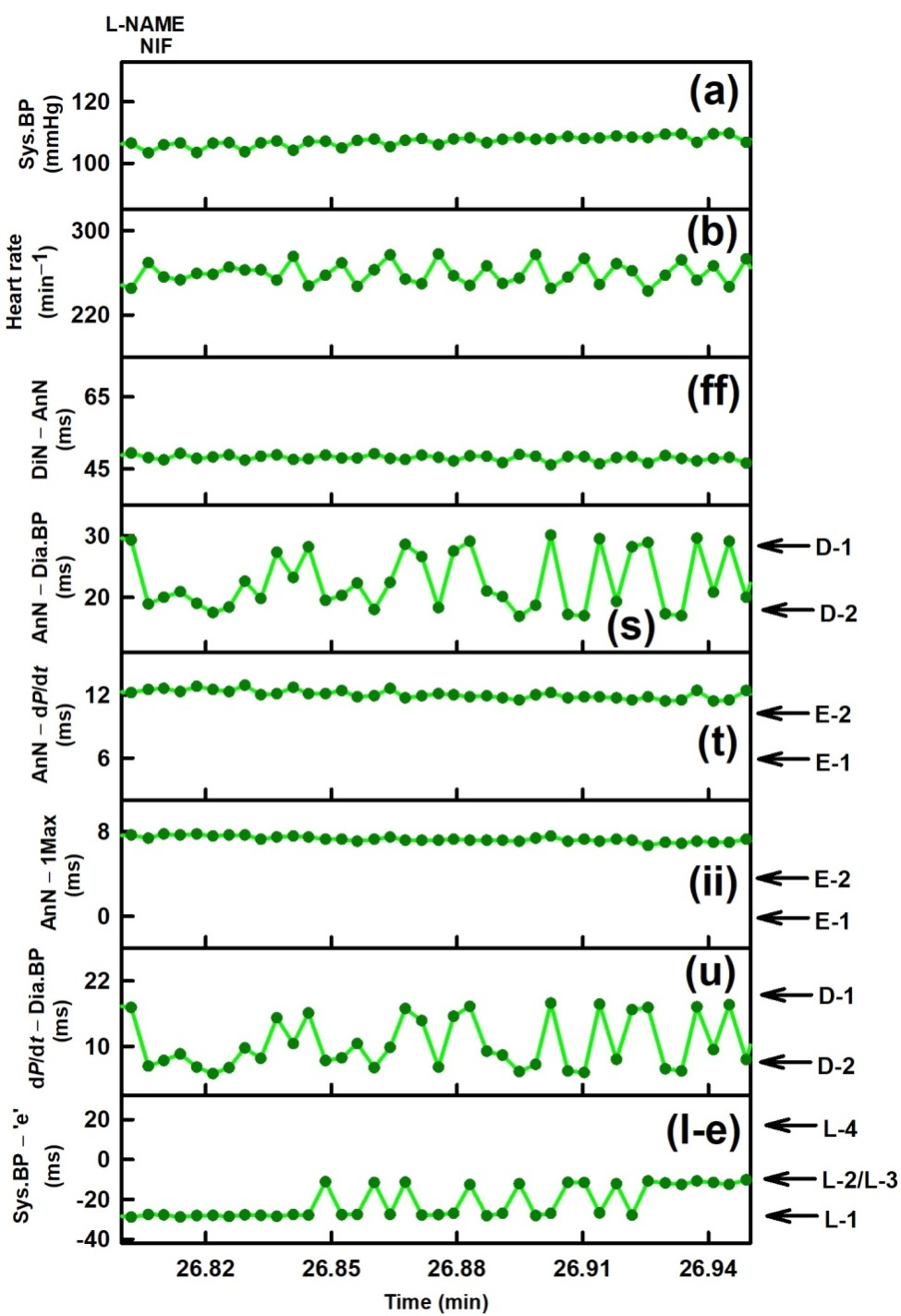


FIGURE S45 Exp-8. Details of the time-dependent changes in APW-Ps of anesthetized rat in the presence of 15 mg kg^–1^ L-NAME and subsequent administration of 400 nmol kg^–1^ NIF (dark green heartbeats). Horizontal arrows indicate predicted D-1 and D-2 levels, E-1 and E-2 levels and L-1 to L-4 levels. The green lines show the connection between adjacent heartbeats. Definitions, units and abbreviations of APW-Ps evaluated from the APW are as explained in legend to FIGURE S6 and Supplementary Information FIGURE S1. Normotensive rats were anesthetized with Zoletil/xylazine.


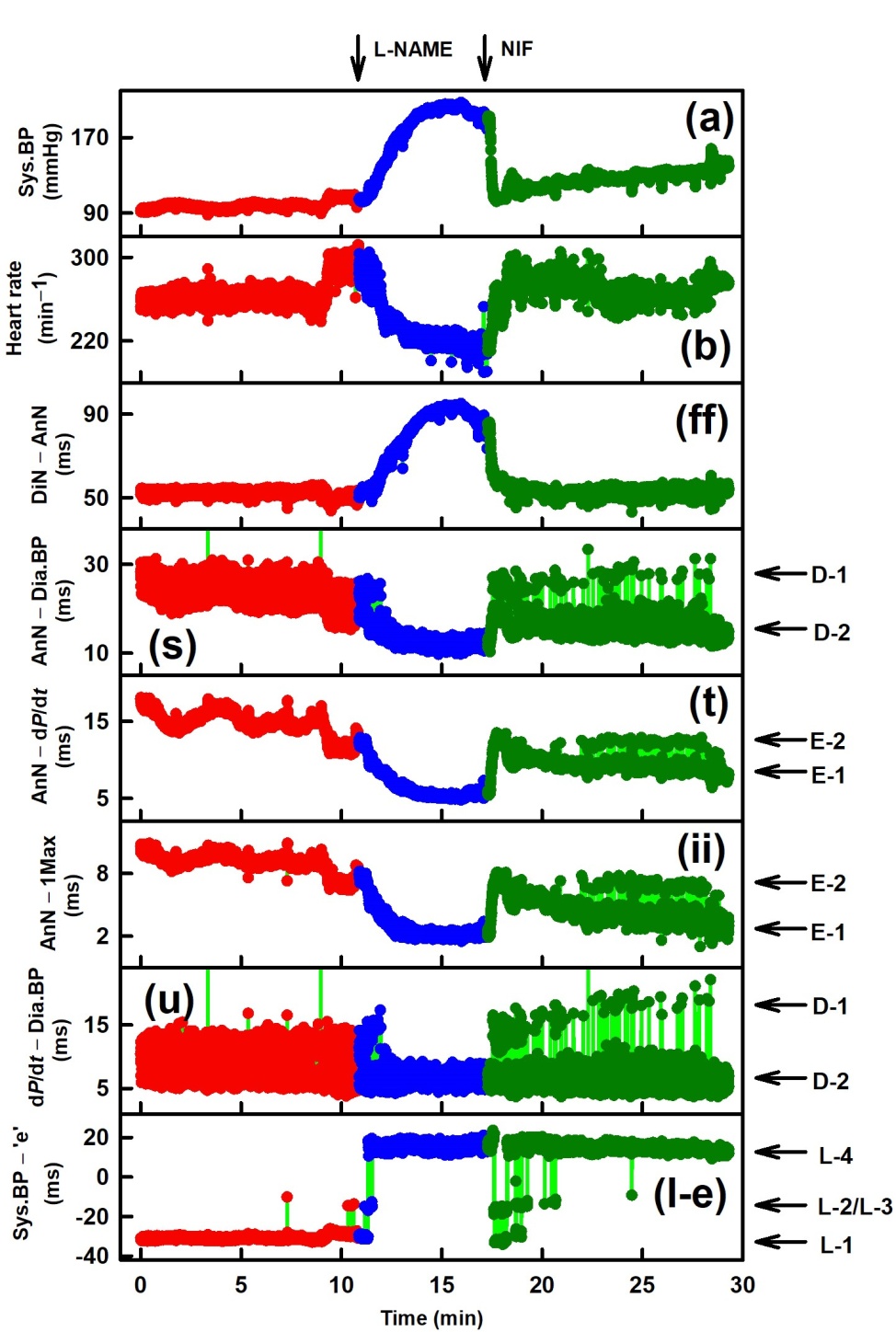


FIGURE S46 Exp-9. Time-dependent changes in APW-Ps of anesthetized rat: control (red heartbeats), after i.v. administration of 15 mg kg^–1^ L-NAME (blue heartbeats) and 400 nmol kg^–1^ NIF (dark green heartbeats). Horizontal arrows indicate predicted D-1 and D-2 levels, E-1 and E-2 levels and L-1 to L-4 levels. The green lines show the connection between adjacent heartbeats. Definitions, units and abbreviations of APW-Ps evaluated from the APW are as explained in legend to FIGURE S6 and Supplementary Information FIGURE S1. Normotensive rats were anesthetized with Zoletil/xylazine.


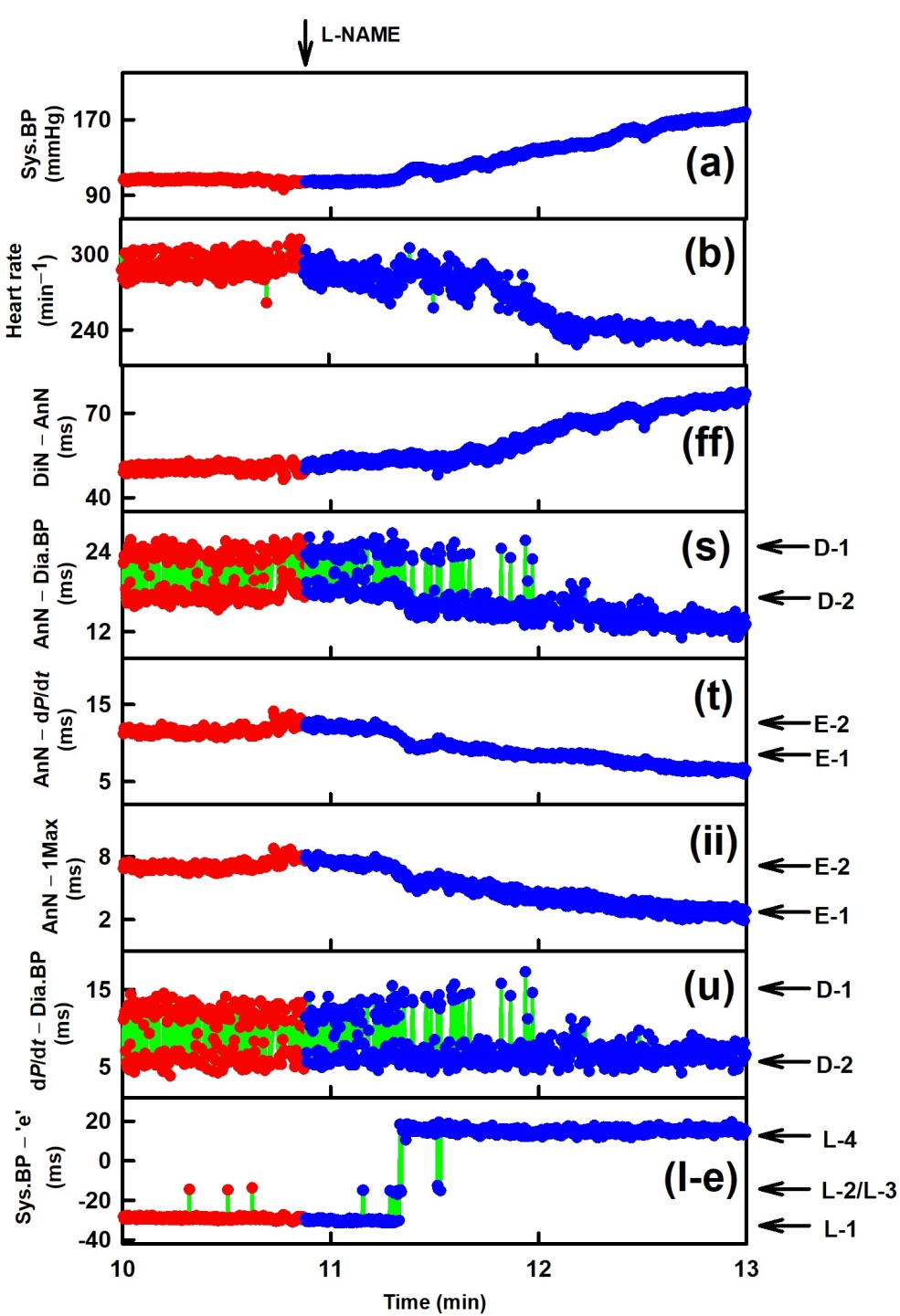


FIGURE S47 Exp-9. Time-dependent changes in APW-Ps of anesthetized rat: control (red heartbeats) and after i.v. administration of 15 mg kg^–1^ L-NAME (blue heartbeats) Horizontal arrows indicate predicted D-1 and D-2 levels, E-1 and E-2 levels and L-1 to L-4 levels. The green lines show the connection between adjacent heartbeats. Definitions, units and abbreviations of APW-Ps evaluated from the APW are as explained in legend to FIGURE S6 and Supplementary Information FIGURE S1. Normotensive rats were anesthetized with Zoletil/xylazine.


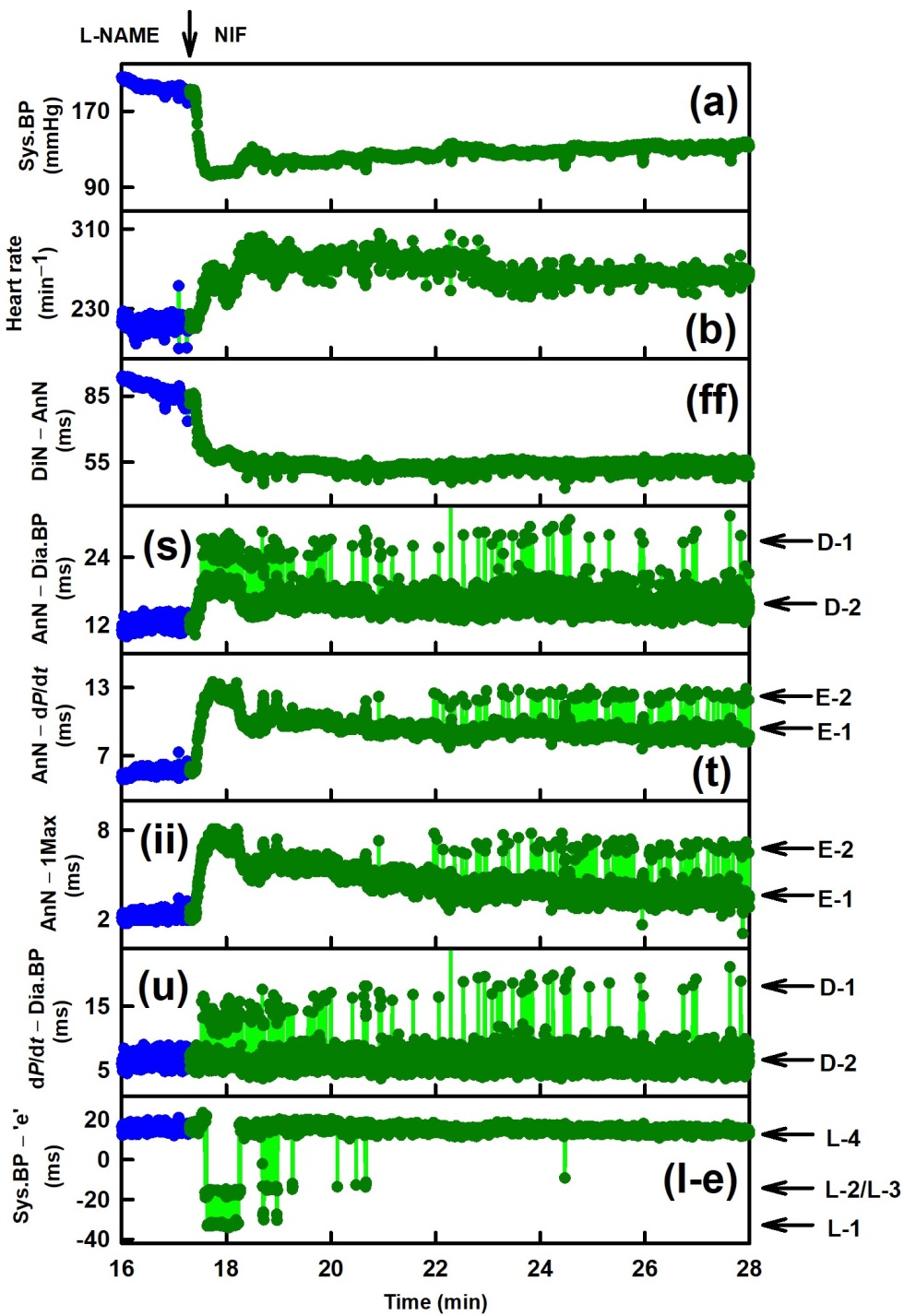


FIGURE S48 Exp-9. Time-dependent changes in APW-Ps of anesthetized rat after i.v. administration of 15 mg kg^–1^ L-NAME (blue heartbeats) and subsequent administration of 400 nmol kg^–1^ NIF (dark green heartbeats). Horizontal arrows indicate predicted D-1 and D-2 levels, E-1 and E-2 levels and L-1 to L-4 levels. The green lines show the connection between adjacent heartbeats. Definitions, units and abbreviations of APW-Ps evaluated from the APW are as explained in legend to FIGURE S6 and Supplementary Information FIGURE S1. Normotensive rats were anesthetized with Zoletil/xylazine.

.


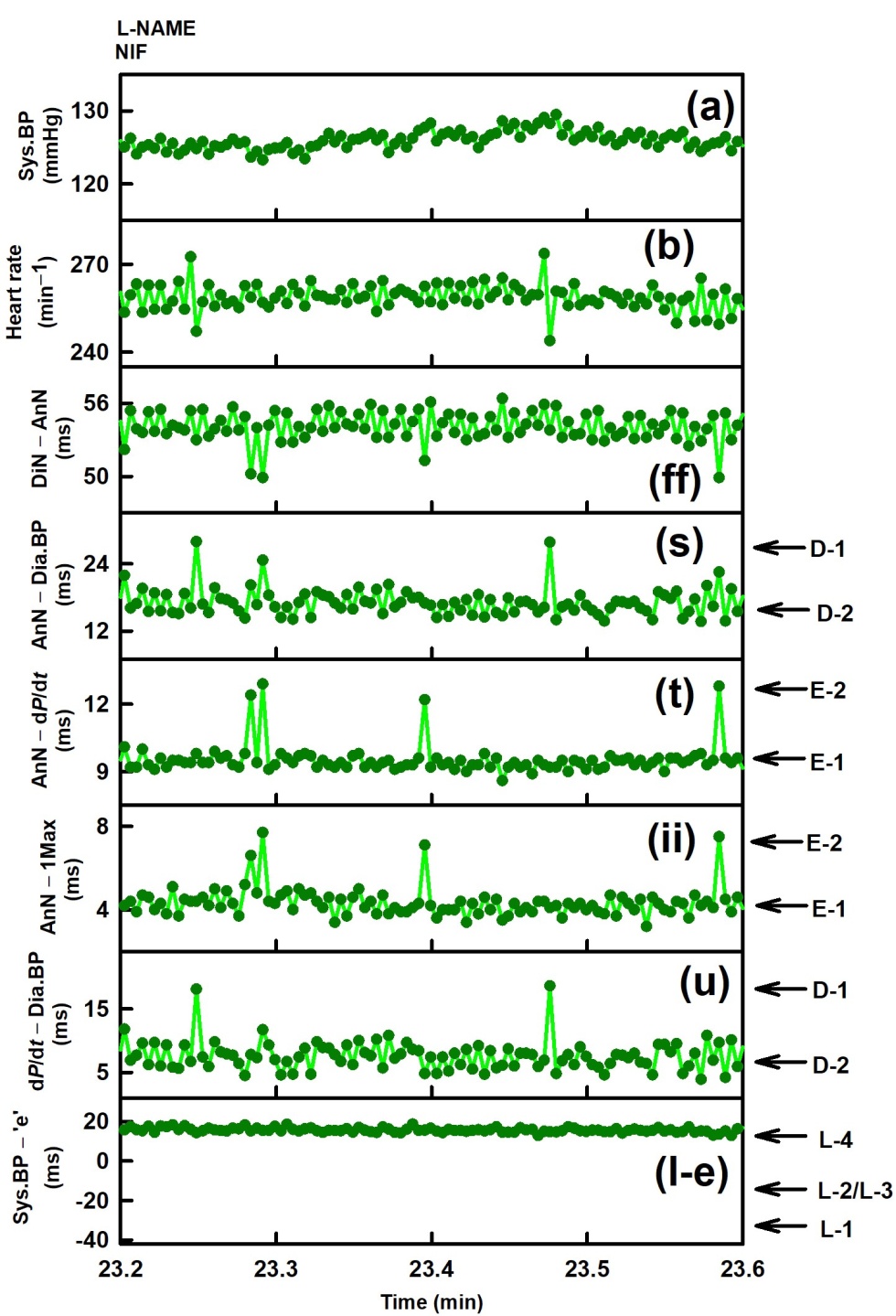


FIGURE S49 Exp-9. Details of the time-dependent changes in APW-Ps of anesthetized rat in the presence of 15 mg kg^–1^ L-NAME and subsequent administration of 400 nmol kg^–1^ NIF (dark green heartbeats). Horizontal arrows indicate predicted D-1 and D-2 levels, E-1 and E-2 levels and L-1 to L-4 levels. The green lines show the connection between adjacent heartbeats. Definitions, units and abbreviations of APW-Ps evaluated from the APW are as explained in legend to FIGURE S6 and Supplementary Information FIGURE S1. Normotensive rats were anesthetized with Zoletil/xylazine.

**
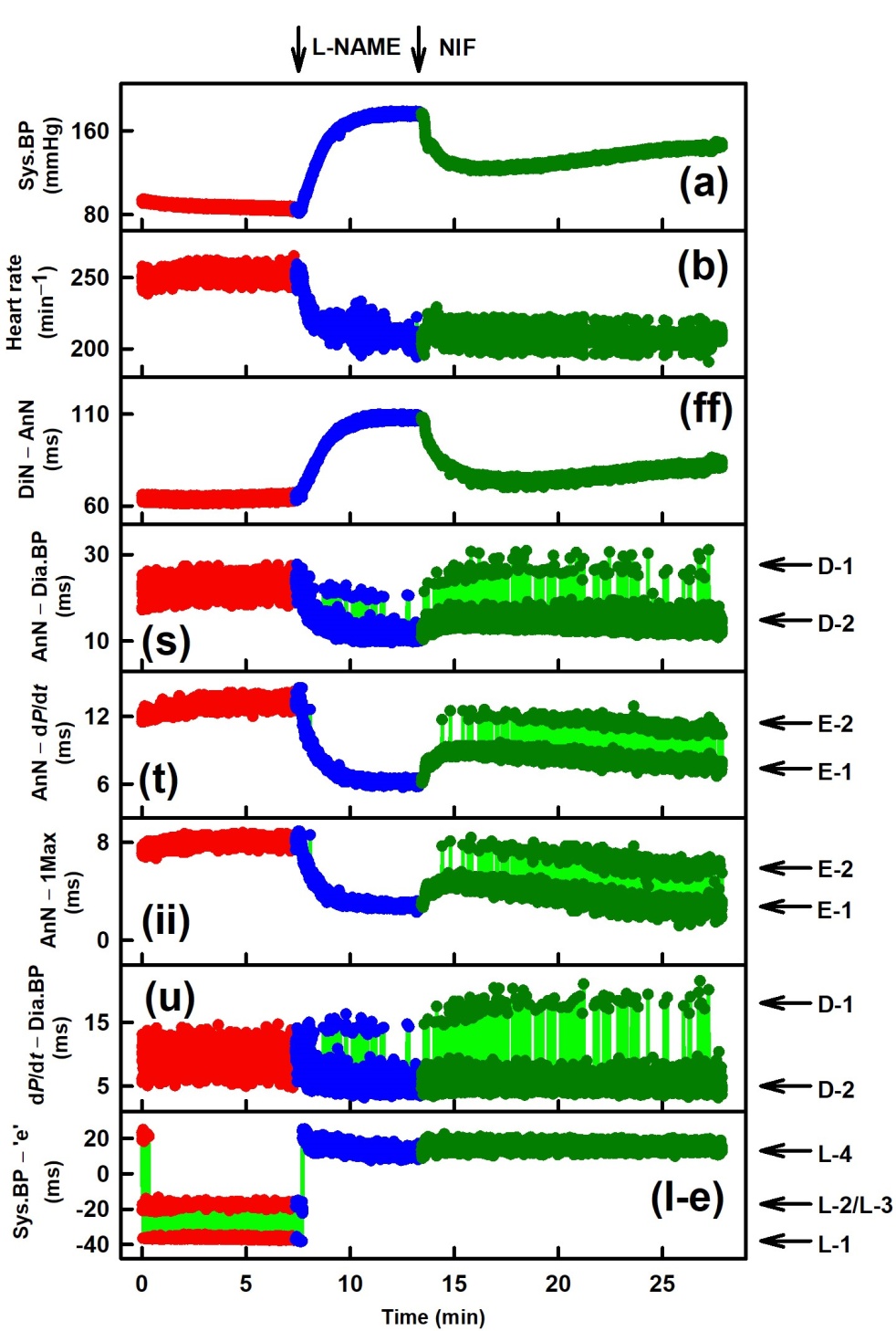
**

FIGURE S50 Exp-10. Time-dependent changes in APW-Ps of anesthetized rat: control (red heartbeats), after i.v. administration of 15 mg kg^–1^ L-NAME (blue heartbeats) and 400 nmol kg^–1^ NIF (dark green heartbeats). Horizontal arrows indicate predicted D-1 and D-2 levels, E-1 and E-2 levels and L-1 to L-4 levels. The green lines show the connection between adjacent heartbeats. Definitions, units and abbreviations of APW-Ps evaluated from the APW are as explained in legend to FIGURE S6 and Supplementary Information FIGURE S1. Normotensive rats were anesthetized with Zoletil/xylazine.

**
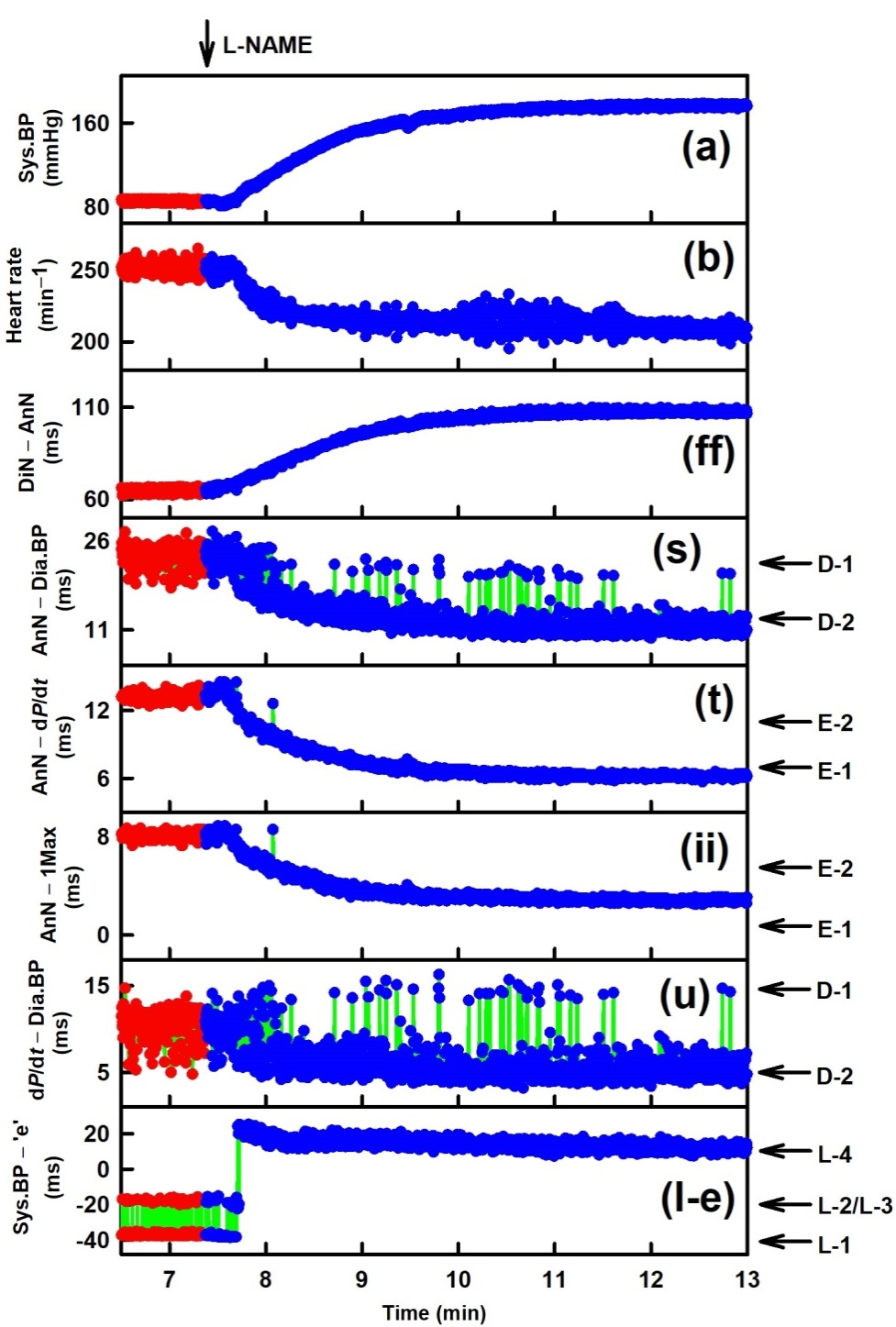
**

FIGURE S51 Exp-10. Time-dependent changes in APW-Ps of anesthetized rat: control (red heartbeats) and after i.v. administration of 15 mg kg^–1^ L-NAME (blue heartbeats) Horizontal arrows indicate predicted D-1 and D-2 levels, E-1 and E-2 levels and L-1 to L-4 levels. The green lines show the connection between adjacent heartbeats. Definitions, units and abbreviations of APW-Ps evaluated from the APW are as explained in legend to FIGURE S6 and Supplementary Information FIGURE S1. Normotensive rats were anesthetized with Zoletil/xylazine.

**
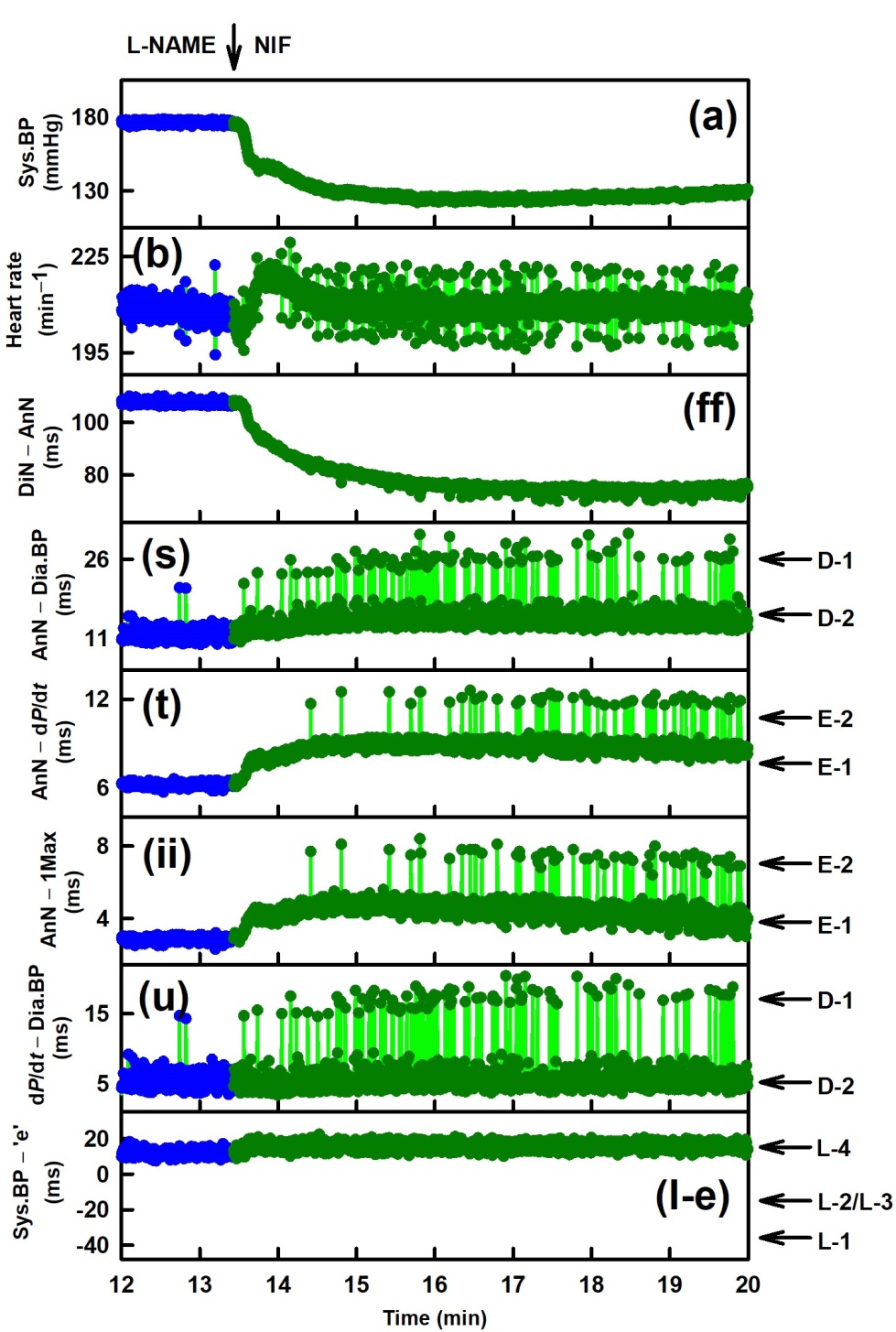
**

FIGURE S52 Exp-10. Time-dependent changes in APW-Ps of anesthetized rat after i.v. administration of 15 mg kg^–1^ L-NAME (blue heartbeats) and subsequent administration of 400 nmol kg^–1^ NIF (dark green heartbeats). Horizontal arrows indicate predicted D-1 and D-2 levels, E-1 and E-2 levels and L-1 to L-4 levels. The green lines show the connection between adjacent heartbeats. Definitions, units and abbreviations of APW-Ps evaluated from the APW are as explained in legend to FIGURE S6 and Supplementary Information FIGURE S1. Normotensive rats were anesthetized with Zoletil/xylazine.

**
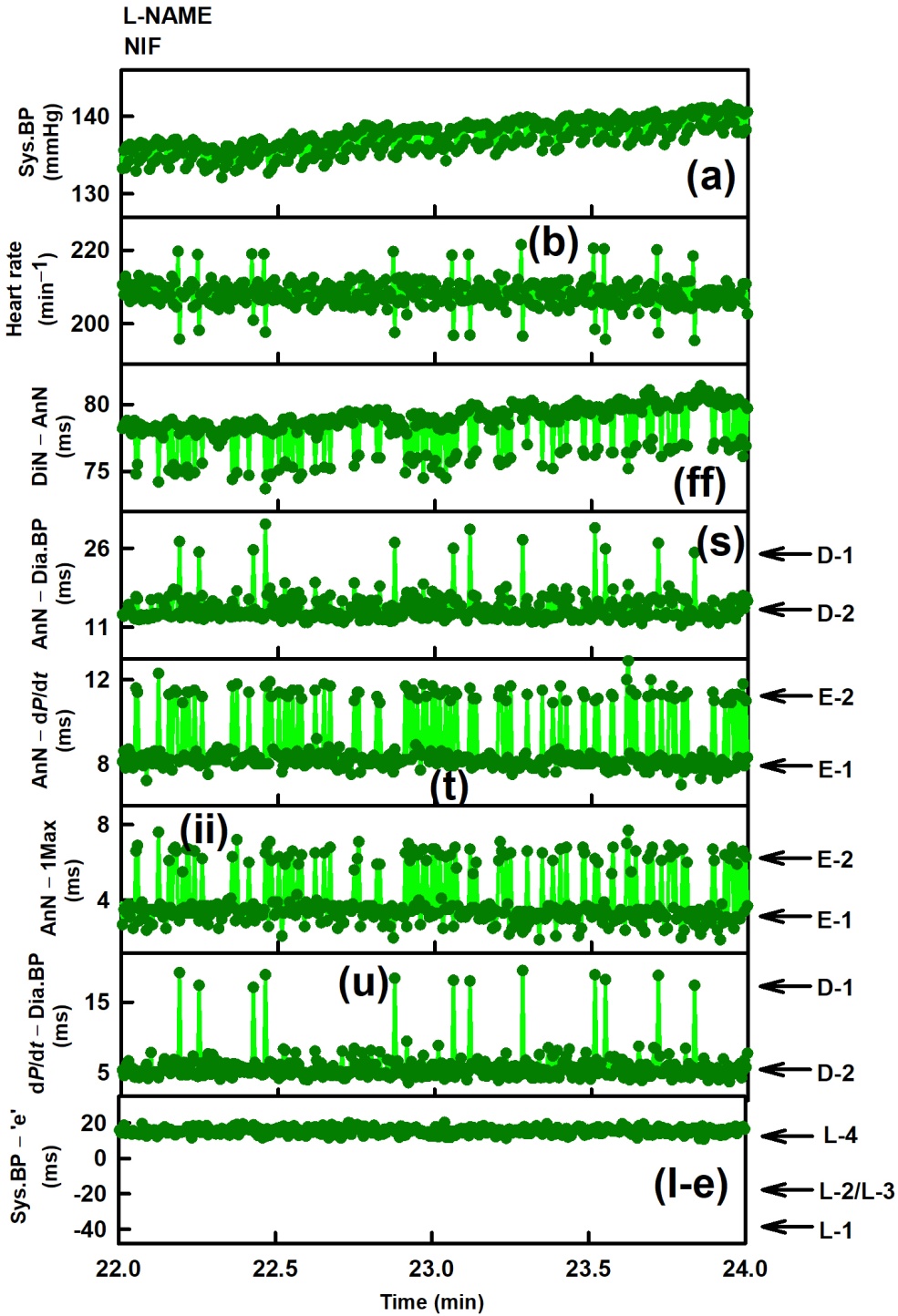
**

FIGURE S53 Exp-10. Time-dependent changes in APW-Ps of anesthetized rat in the presence of 15 mg kg^–1^ L-NAME (blue heartbeats) and subsequent administration of 400 nmol kg^–1^ NIF (dark green heartbeats). Horizontal arrows indicate predicted D-1 and D-2 levels, E-1 and E-2 levels and L-1 to L-4 levels. The green lines show the connection between adjacent heartbeats. Definitions, units and abbreviations of APW-Ps evaluated from the APW are as explained in legend to FIGURE S6 and Supplementary Information FIGURE S1. Normotensive rats were anesthetized with Zoletil/xylazine.

**
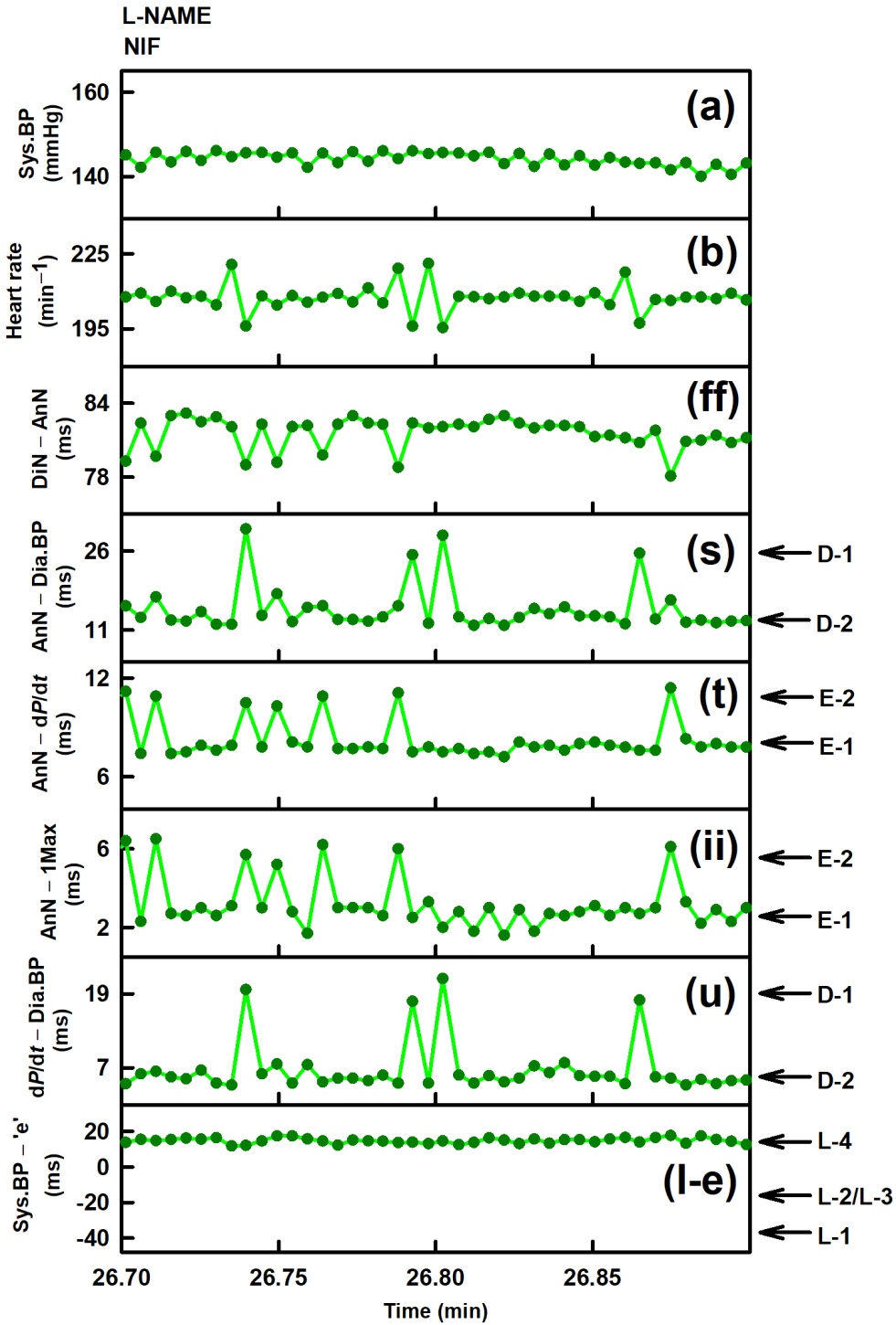
**

FIGURE S54 Exp-10. Details of the time-dependent changes in APW-Ps of anesthetized rat in the presence of 15 mg kg^–1^ L-NAME and subsequent administration of 400 nmol kg^–1^ NIF (dark green heartbeats). Horizontal arrows indicate predicted D-1 and D-2 levels, E-1 and E-2 levels and L-1 to L-4 levels. The green lines show the connection between adjacent heartbeats. Definitions, units and abbreviations of APW-Ps evaluated from the APW are as explained in legend to FIGURE S6 and Supplementary Information FIGURE S1. Normotensive rats were anesthetized with Zoletil/xylazine.


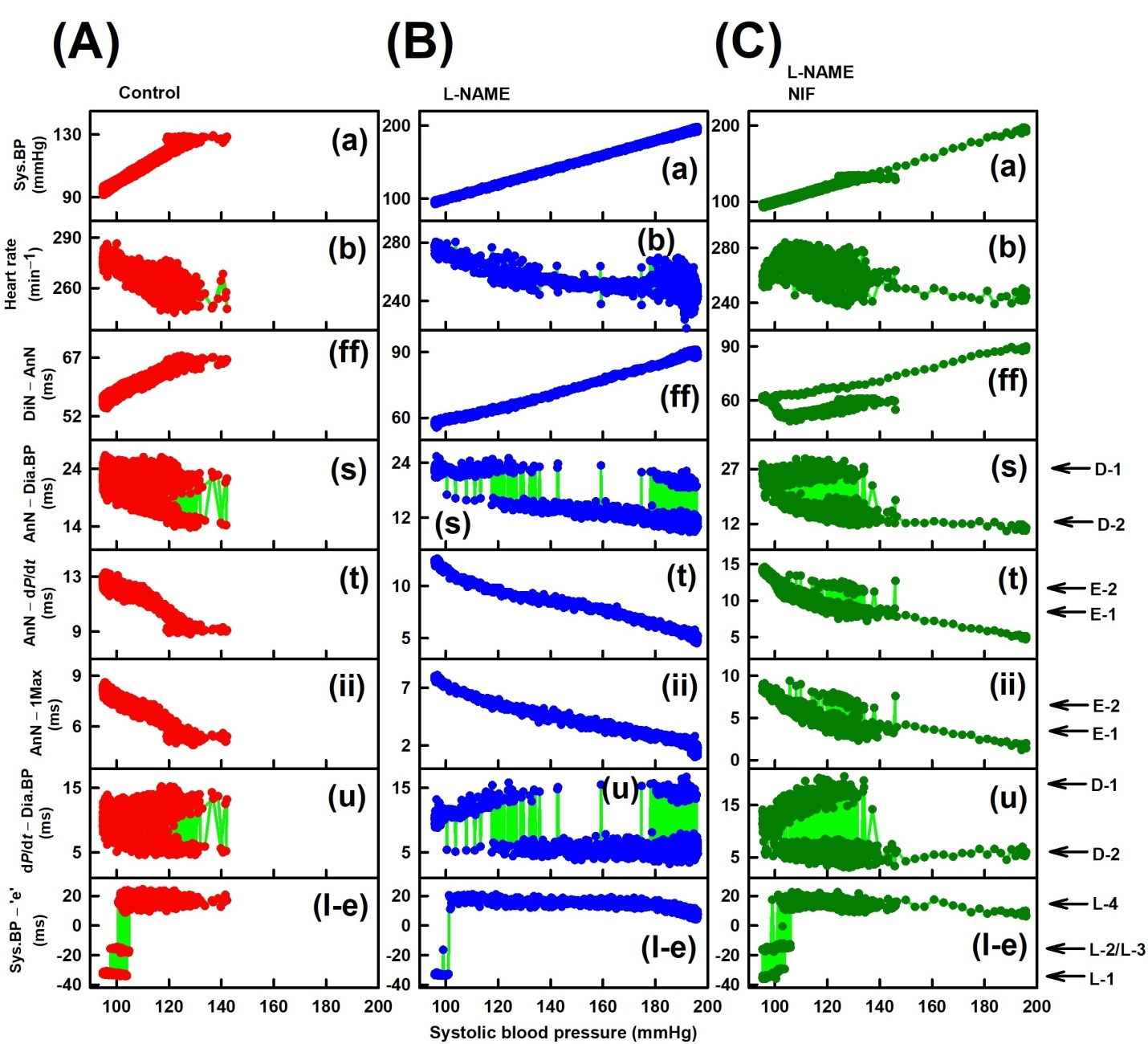


FIGURE S55 Exp-1. Cross-relationships of eight APW-Ps to systolic BP. **a)** At control (red heartbeats) and **b)** after the i.v. administration of 15 mg kg^–1^ L-NAME **(**blue heartbeats**)** and **c)** after subsequent administration of 400 nmol kg^–1^ of NIF (dark green heartbeats**).** The green lines show the connection between adjacent heartbeats. Arrows indicate predicted levels E-1, E-2, D-1, D-2 and L-1 to L-4. Definitions, units and abbreviations of APW-Ps evaluated from the APW are as explained in legend to FIGURE S6 and Supplementary Information FIGURE S1. Normotensive rats were anesthetized with Zoletil/xylazine.


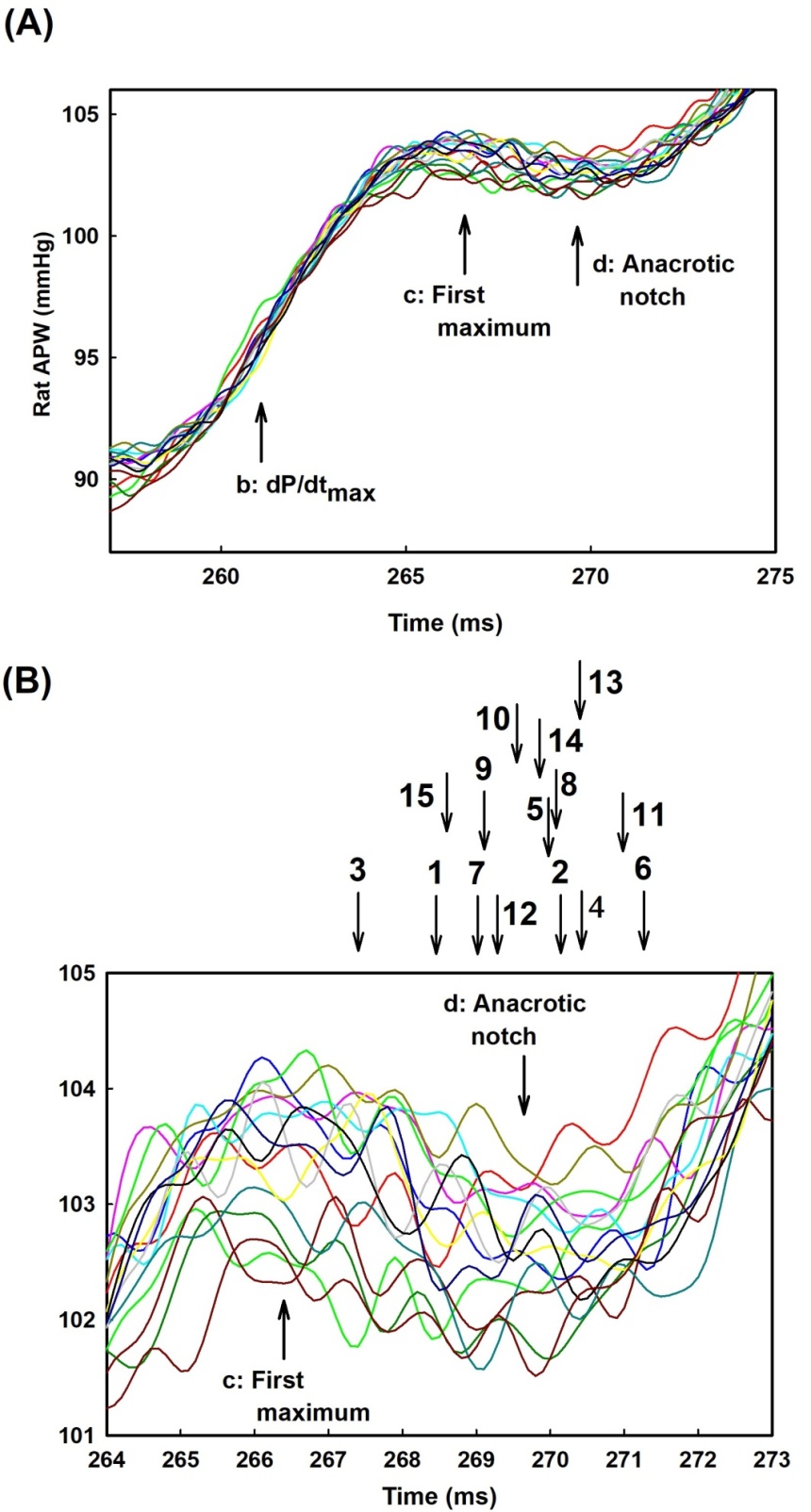


FIGURE S56 Exp-10. (A) Part of APW of 14 consecutive pulses from 22.96 min to 23.03 min. Arrows show position of APW parameters (b), (c) and (d). (B) Details of the part of APW of 14 consecutive pulses. Arrows inside box show position of APW parameters (c) and (d). Arrows autside of box show position of anacrotic notch (d) for individual heartbeats. Normotensive rats were anesthetized with Zoletil/xylazine.


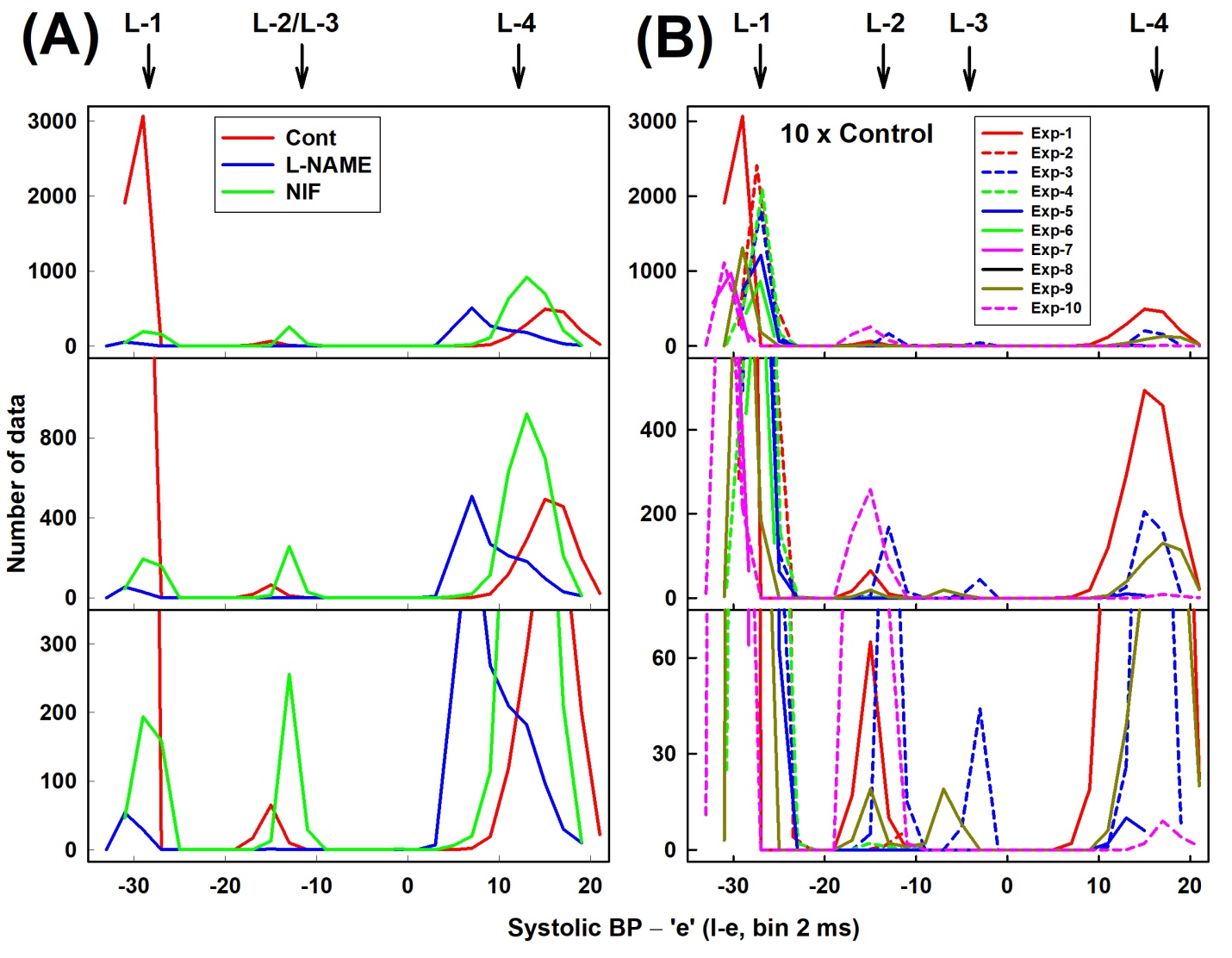


FIGURE S57 Histograms of (l-e) normalized to 300 min^–1^ HR. (A) Exp-1, at control (red) and after the i.v. administration of 15 mg kg^–1^ L-NAME (blue**)** and after subsequent administration of 400 nmol kg^–1^ of NIF **(**green**).** (B) Histogram of (l-e) from ten rats experiments at control. Histograms are at three resolutions. Arrows indicate predicted levels L-1 to L-4. Normotensive rats were anesthetized with Zoletil/xylazine.


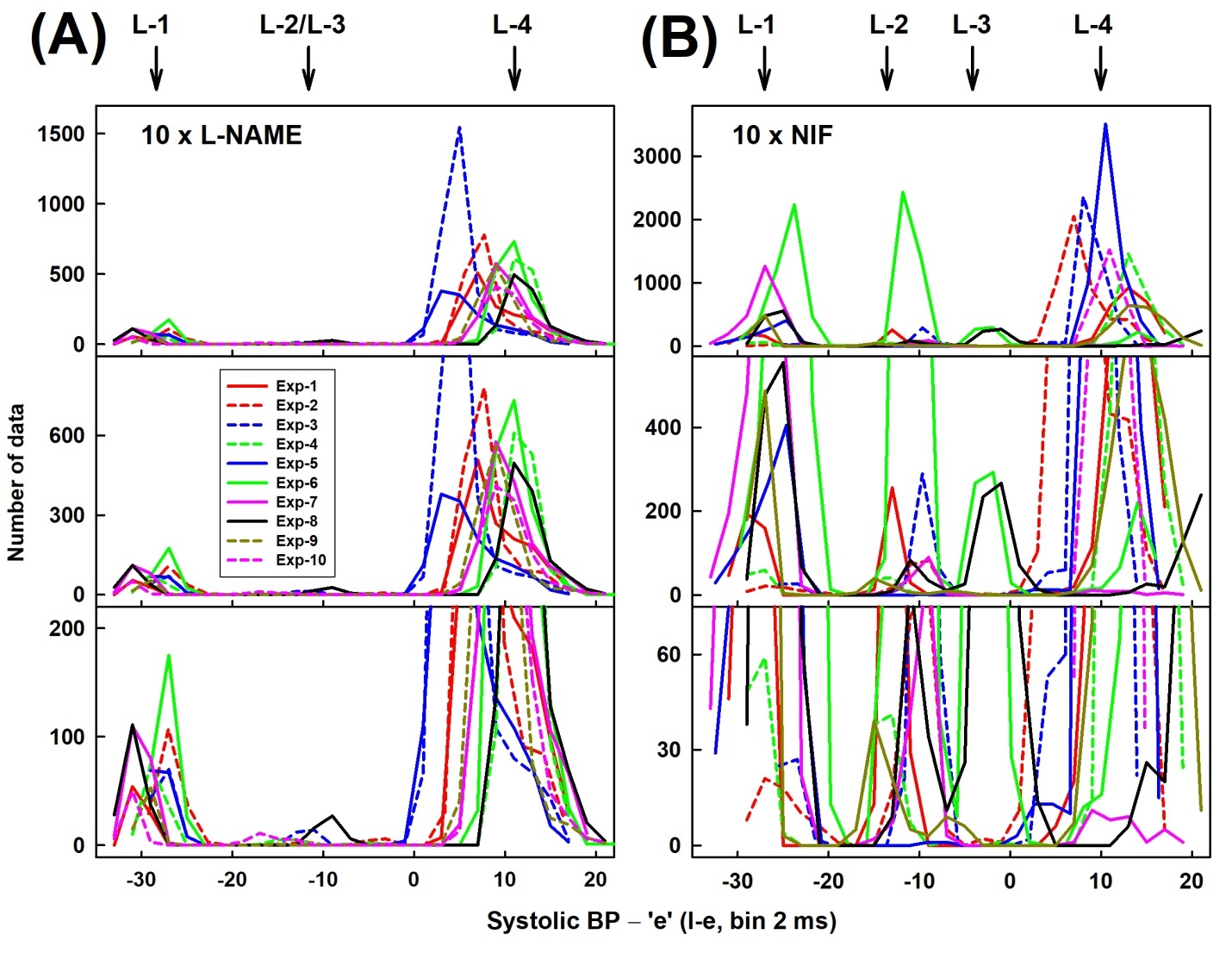


FIGURE S58 Histograms (l-e) of ten rats normalized to 300 min^–1^ HR. (A) In the presence of 15 mg kg^–1^ L-NAME and (B) after subsequent administration of NIF. Data are at three resolutions. Arrows indicate predicted levels L-1 to L-4. Colors and lines for ten experiments are the same as in FIGURE S57. Normotensive rats were anesthetized with Zoletil/xylazine.


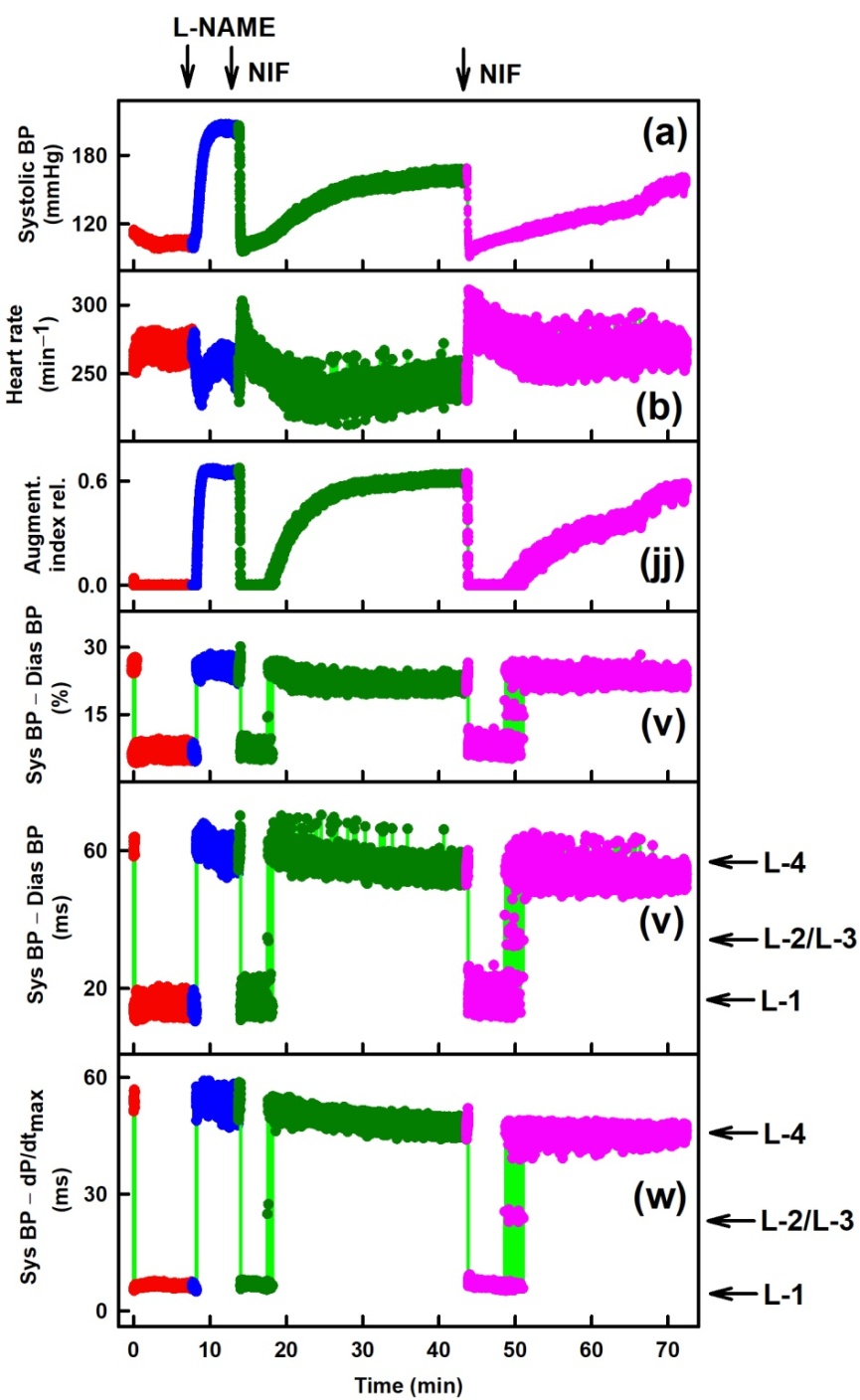


FIGURE S59 Exp-2. Time-dependent changes in APW-Ps, systolic BP (mmHg) (a), heart rate (min^–1^) (b), augmentation index (jj), systolic BP – diastolic BP (v,%), systolic BP – diastolic BP (ms) (v) and systolic BP – dP/dt_max_ (ms) (w) in control **(**red**),** after the i.v. administration of of 15 mg kg^–1^ L-NAME **(**blue**)** and after subsequent administration of 400 nmol kg^–1^ of NIF (dark green). Horizontal arrows indicate predicted L-1 to L-4 levels. The green lines show the connection between adjacent heartbeats. Definitions, units and abbreviations of APW-Ps evaluated from the APW are as explained in Supplementary Information FIGURE S1. Normotensive rats were anesthetized with Zoletil/xylazine.

**
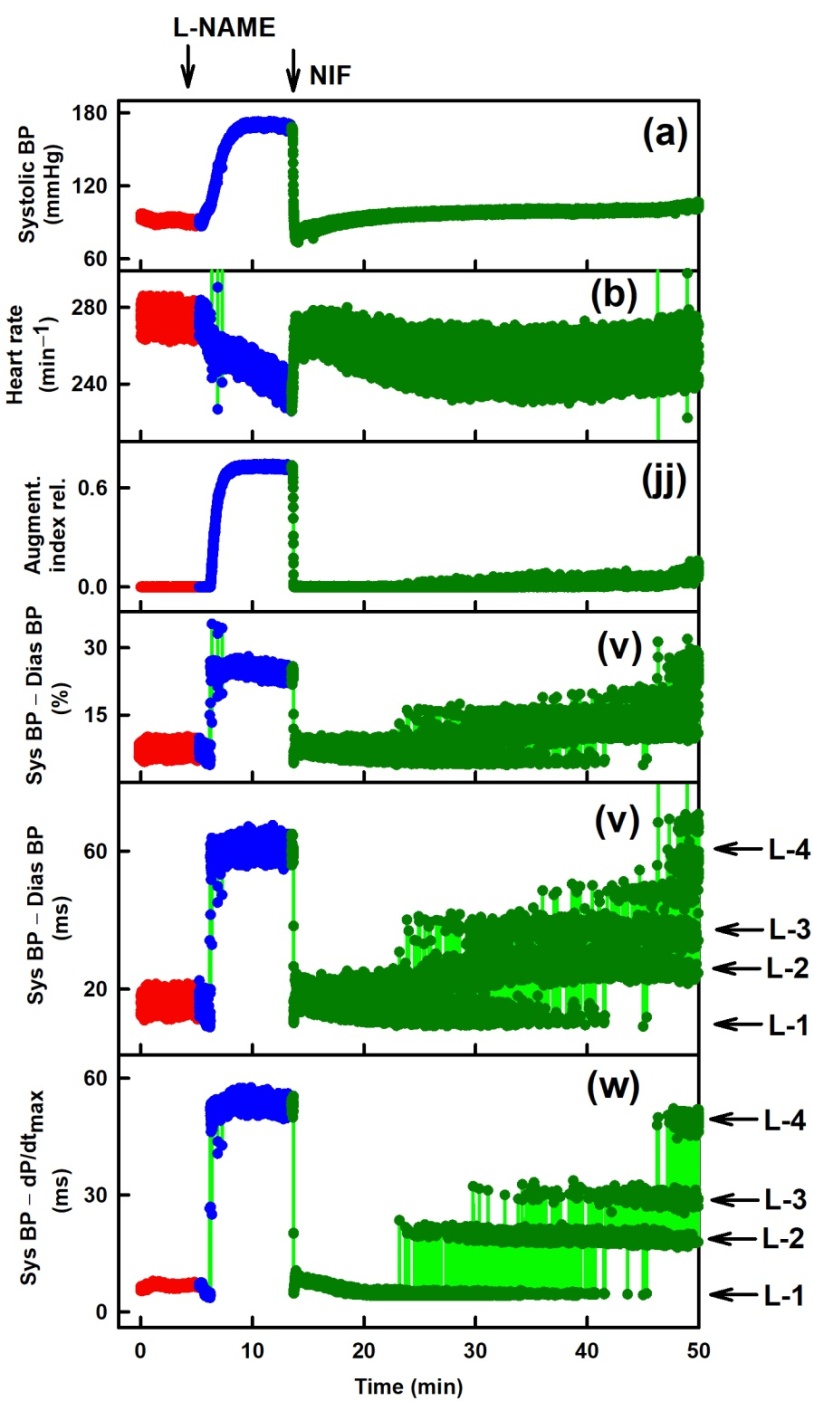
**

FIGURE S60 Exp-3. Time-dependent changes in APW-Ps, systolic BP (mmHg) (a), heart rate (min^–1^) (b), augmentation index (jj), systolic BP – diastolic BP (v,%), systolic BP – diastolic BP (ms) (v) and systolic BP – dP/dt_max_ (ms) (w) in control (red), after the i.v. administration of of 15 mg kg^–1^ L-NAME **(**blue**)** and after subsequent administration of 400 nmol kg^–1^ of NIF (dark green). Horizontal arrows indicate predicted L-1 to L-4 levels. The green lines show the connection between adjacent heartbeats. Definitions, units and abbreviations of APW-Ps evaluated from the APW are as explained in Supplementary Information FIGURE S1. Normotensive rats were anesthetized with Zoletil/xylazine.


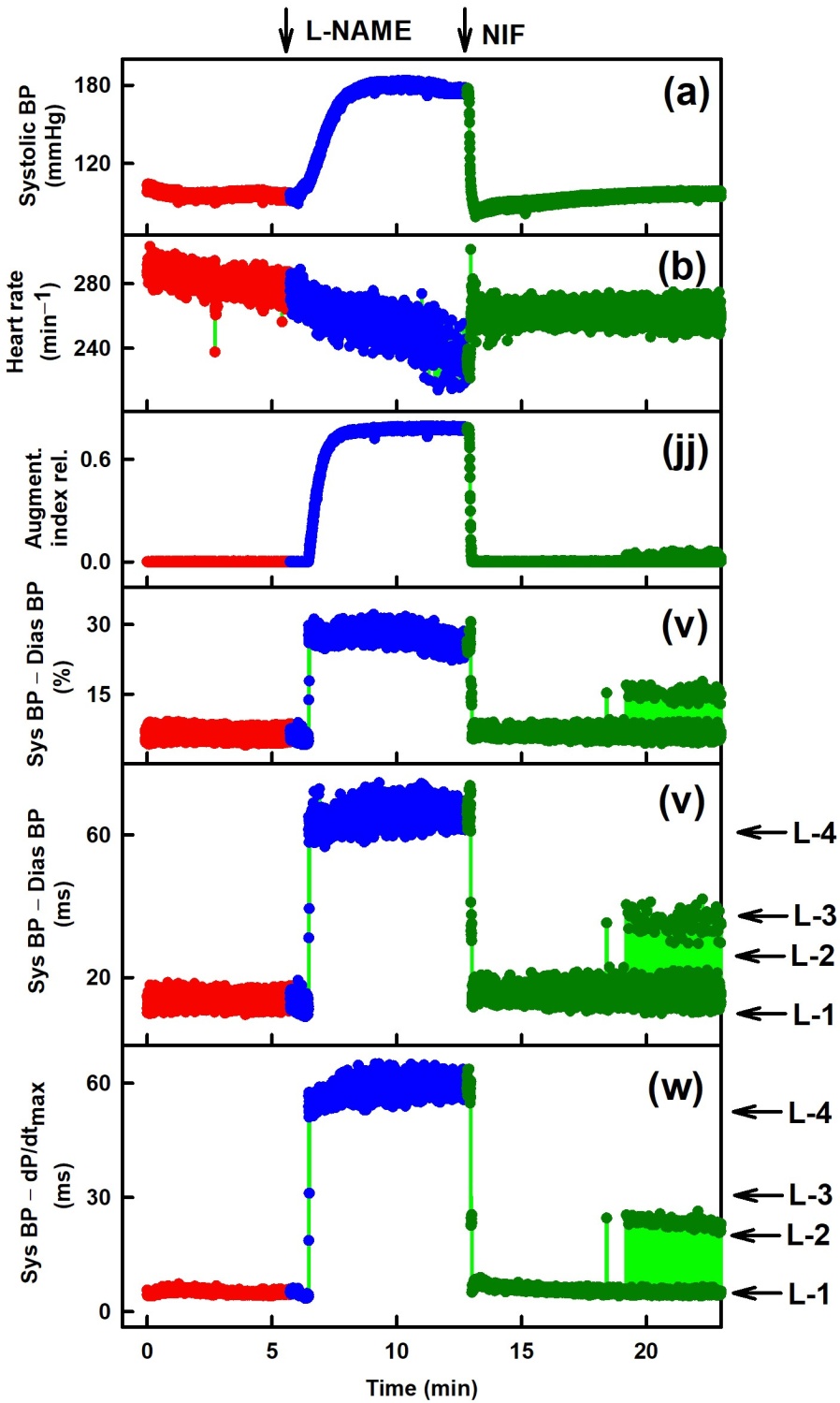


FIGURE S61 Exp-4. Time-dependent changes in APW-Ps, systolic BP (mmHg) (a), heart rate (min^–1^) (b), augmentation index (jj), systolic BP – diastolic BP (v,%), systolic BP – diastolic BP (ms) (v) and systolic BP – dP/dt_max_ (ms) (w) in control **(**red**),** after the i.v. administration of of 15 mg kg^–1^ L-NAME **(**blue**)** and after subsequent administration of 400 nmol kg^–1^ of NIF (dark green). Horizontal arrows indicate predicted L-1 to L-4 levels. The green lines show the connection between adjacent heartbeats. Definitions, units and abbreviations of APW-Ps evaluated from the APW are as explained in Supplementary Information FIGURE S1. Normotensive rats were anesthetized with Zoletil/xylazine.


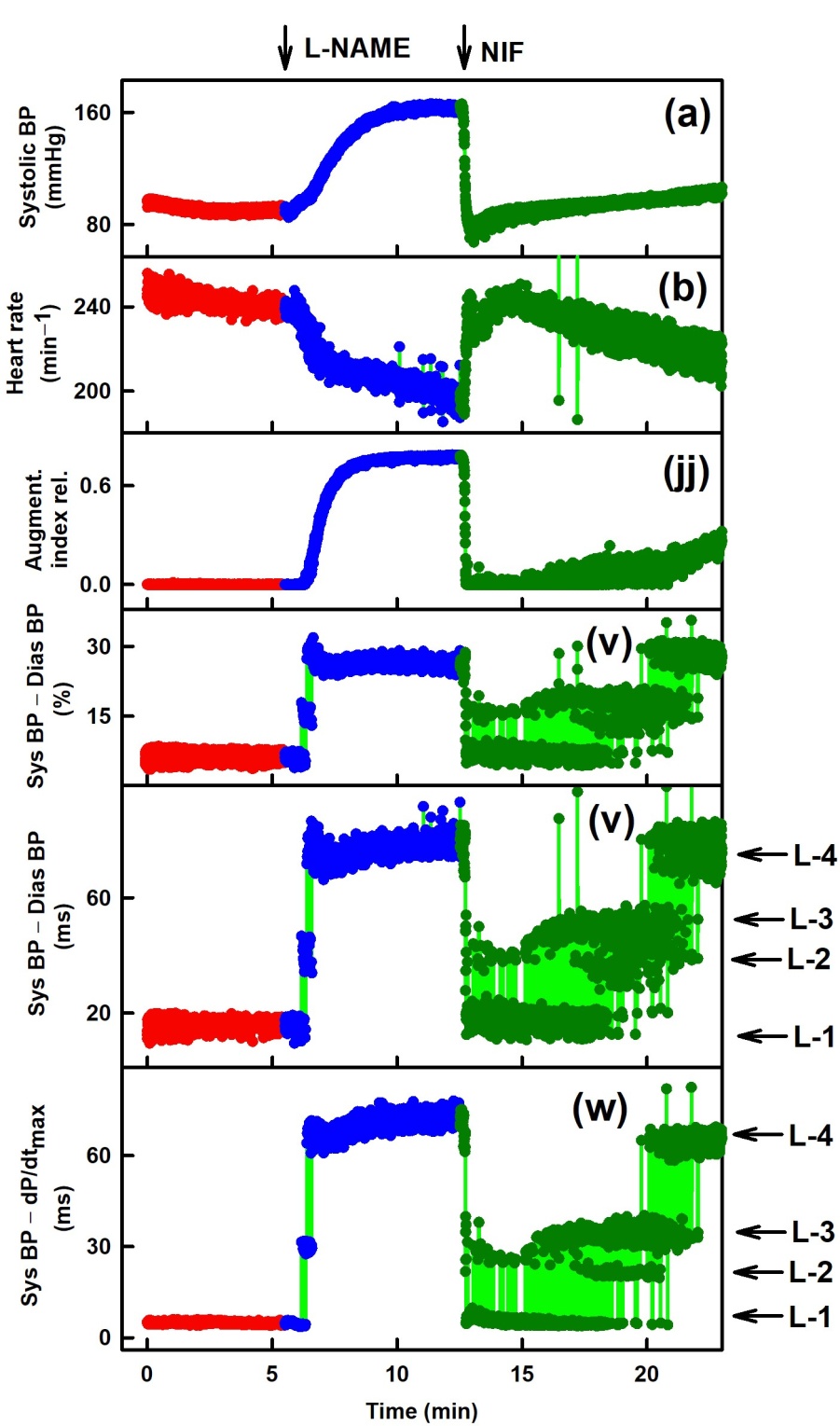


FIGURE S62 Exp-5. Time-dependent changes in APW-Ps, systolic BP (mmHg) (a), heart rate (min^–1^) (b), augmentation index (jj), systolic BP – diastolic BP (v,%), systolic BP – diastolic BP (ms) (v) and systolic BP – dP/dt_max_ (ms) (w) in control **(**red**),** after the i.v. administration of of 15 mg kg^–1^ L-NAME **(**blue**)** and after subsequent administration of 400 nmol kg^–1^ of NIF (dark green). Horizontal arrows indicate predicted L-1 to L-4 levels. The green lines show the connection between adjacent heartbeats. Definitions, units and abbreviations of APW-Ps evaluated from the APW are as explained in Supplementary Information FIGURE S1. Normotensive rats were anesthetized with Zoletil/xylazine.


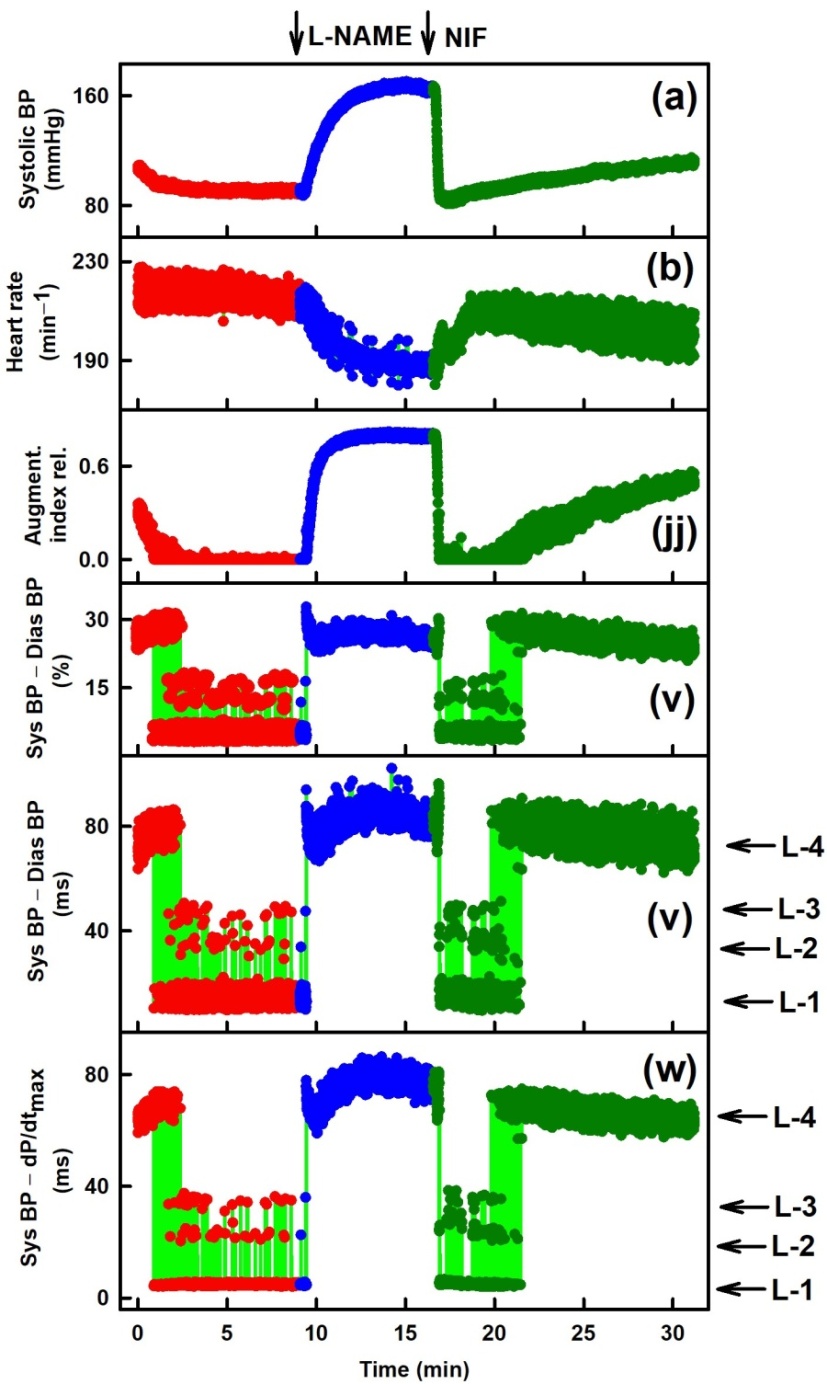


FIGURE S63 Exp-6. Time-dependent changes in APW-Ps, systolic BP (mmHg) (a), heart rate (min^–1^) (b), augmentation index (jj), systolic BP – diastolic BP (v,%), systolic BP – diastolic BP (ms) (v) and systolic BP – dP/dt_max_ (ms) (w) in control **(**red**),** after the i.v. administration of of 15 mg kg^–1^ L-NAME **(**blue**)** and after subsequent administration of 400 nmol kg^–1^ of NIF (dark green). Horizontal arrows indicate predicted L-1 to L-4 levels. The green lines show the connection between adjacent heartbeats. Definitions, units and abbreviations of APW-Ps evaluated from the APW are as explained in Supplementary Information FIGURE S1. Normotensive rats were anesthetized with Zoletil/xylazine.


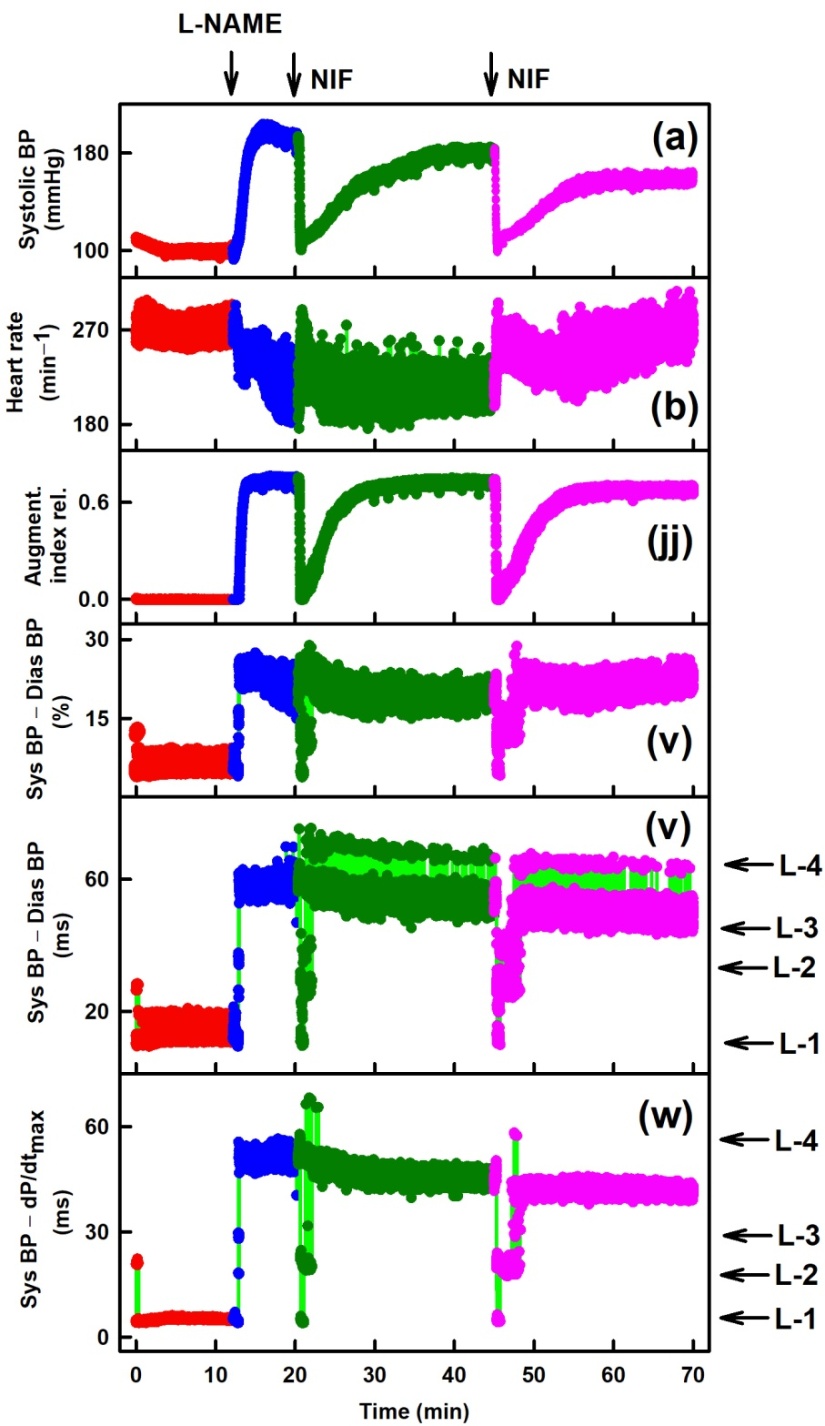


FIGURE S64 Exp-7. Time-dependent changes in APW-Ps, systolic BP (mmHg) (a), heart rate (min^–1^) (b), augmentation index (jj), systolic BP – diastolic BP (v,%), systolic BP – diastolic BP (ms) (v) and systolic BP – dP/dt_max_ (ms) (w) in control **(**red**),** after the i.v. administration of of 15 mg kg^–1^ L-NAME **(**blue**)** and after subsequent administration of 400 nmol kg^–1^ of NIF (dark green). Horizontal arrows indicate predicted L-1 to L-4 levels. The green lines show the connection between adjacent heartbeats. Definitions, units and abbreviations of APW-Ps evaluated from the APW are as explained in Supplementary Information FIGURE S1. Normotensive rats were anesthetized with Zoletil/xylazine.


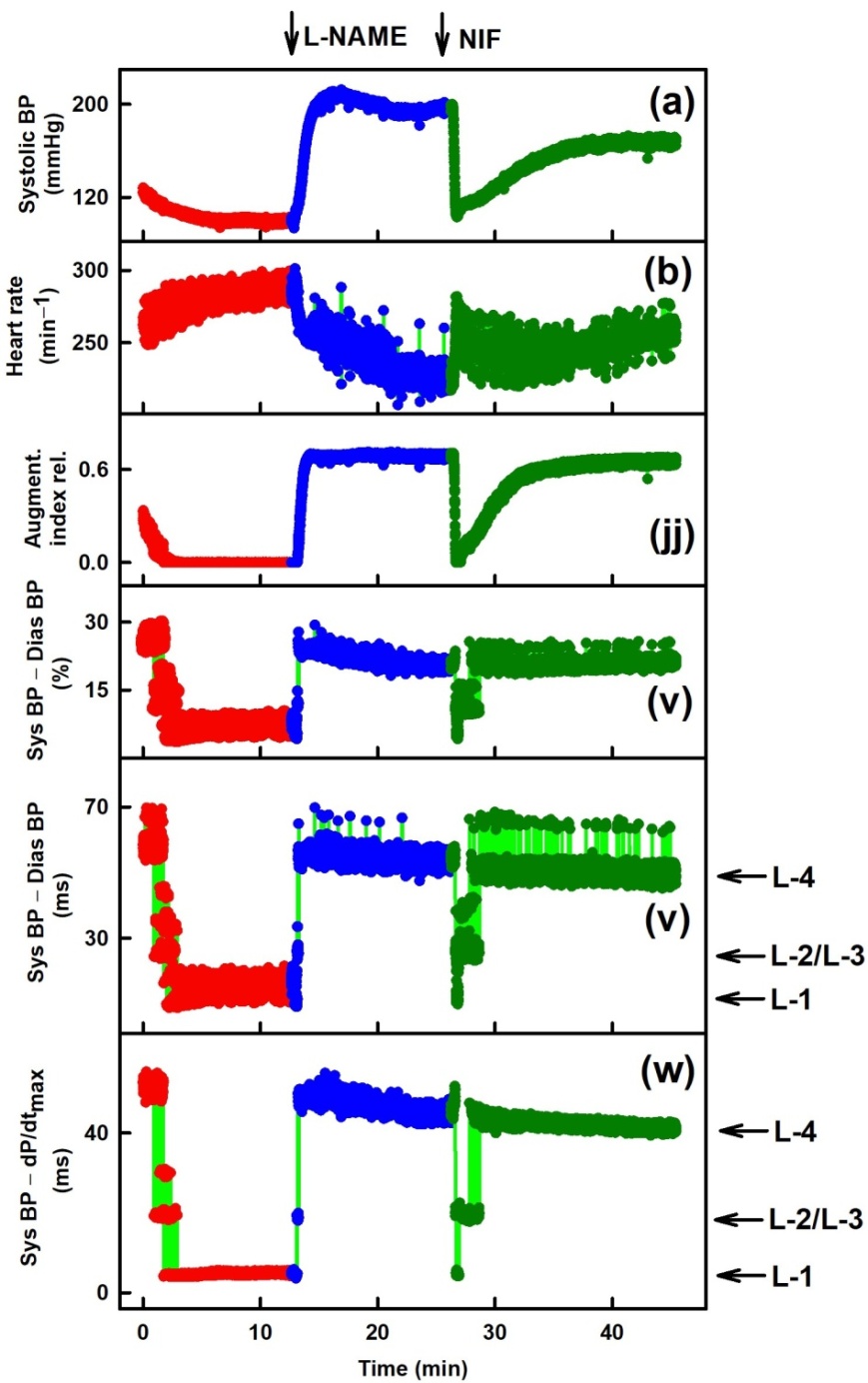


FIGURE S65 Exp-8. Time-dependent changes in APW-Ps, systolic BP (mmHg) (a), heart rate (min^–1^) (b), augmentation index (jj), systolic BP – diastolic BP (v,%), systolic BP – diastolic BP (ms) (v) and systolic BP – dP/dt_max_ (ms) (w) in control **(**red**),** after the i.v. administration of of 15 mg kg^–1^ L-NAME **(**blue**)** and after subsequent administration of 400 nmol kg^–1^ of NIF (dark green). Horizontal arrows indicate predicted L-1 to L-4 levels. The green lines show the connection between adjacent heartbeats. Definitions, units and abbreviations of APW-Ps evaluated from the APW are as explained in Supplementary Information FIGURE S1. Normotensive rats were anesthetized with Zoletil/xylazine.


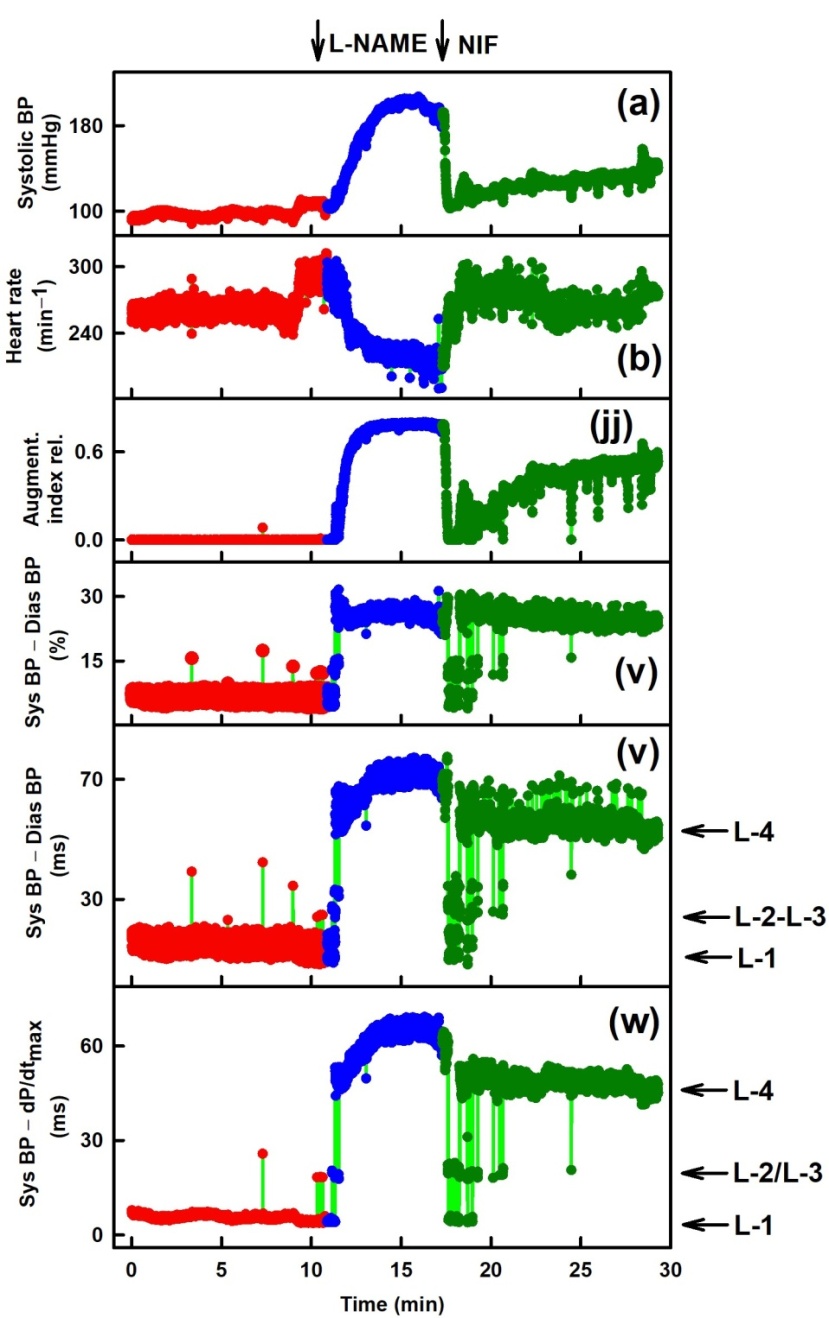


FIGURE S66 Exp-9. Time-dependent changes in APW-Ps, systolic BP (mmHg) (a), heart rate (min^–1^) (b), augmentation index (jj), systolic BP – diastolic BP (v,%), systolic BP – diastolic BP (ms) (v) and systolic BP – dP/dt_max_ (ms) (w) in control **(**red**),** after the i.v. administration of of 15 mg kg^–1^ L-NAME **(**blue**)** and after subsequent administration of 400 nmol kg^–1^ of NIF (dark green). Horizontal arrows indicate predicted L-1 to L-4 levels. The green lines show the connection between adjacent heartbeats. Definitions, units and abbreviations of APW-Ps evaluated from the APW are as explained in Supplementary Information FIGURE S1. Normotensive rats were anesthetized with Zoletil/xylazine.


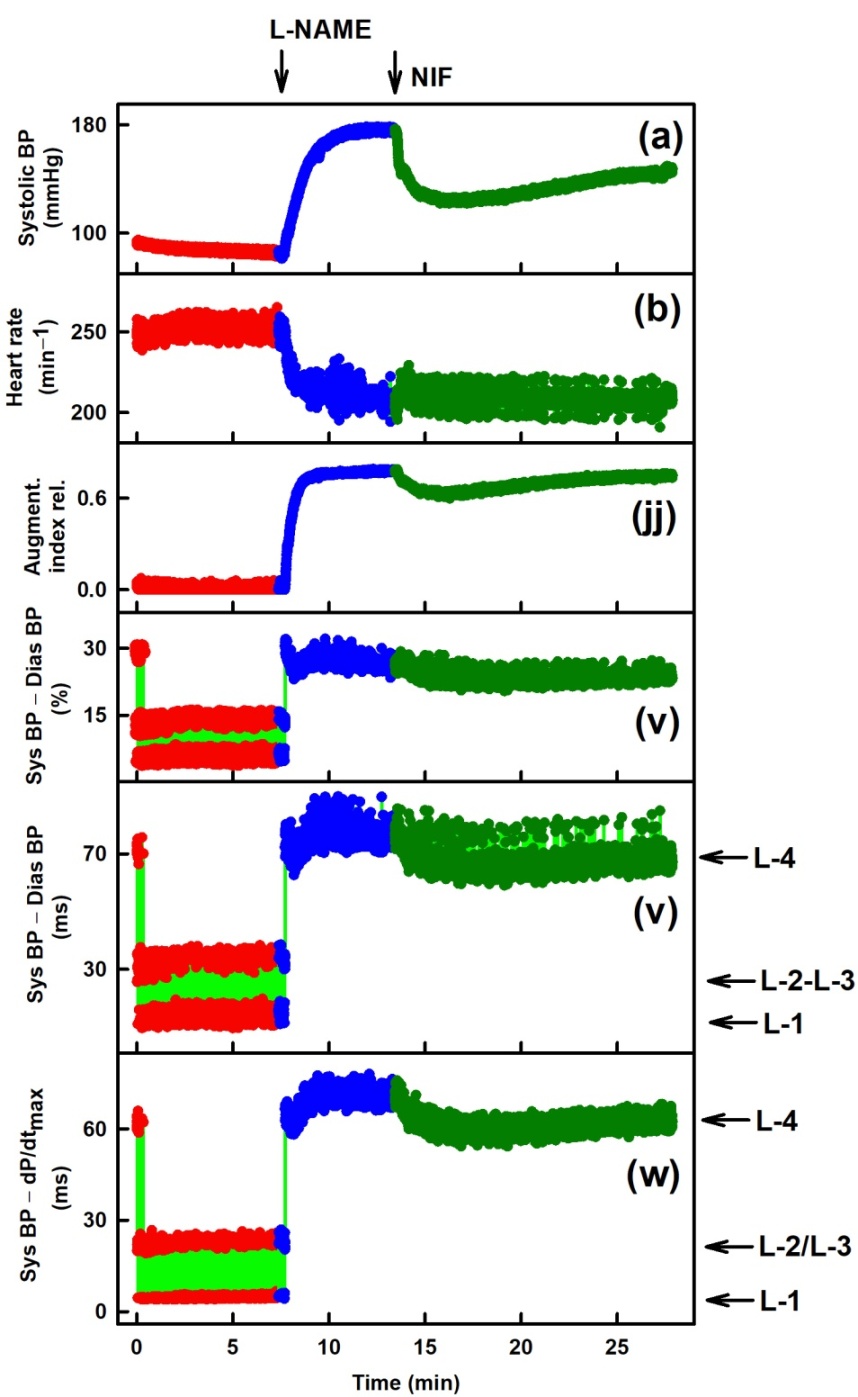


FIGURE S67 Exp-10. Time-dependent changes in APW-Ps, systolic BP (mmHg) (a), heart rate (min^–1^) (b), augmentation index (jj), systolic BP – diastolic BP (v,%), systolic BP – diastolic BP (ms) (v) and systolic BP – dP/dt_max_ (ms) (w) in control **(**red**),** after the i.v. administration of of 15 mg kg^–1^ L-NAME **(**blue**)** and after subsequent administration of 400 nmol kg^–1^ of NIF (dark green). Horizontal arrows indicate predicted L-1 to L-4 levels. The green lines show the connection between adjacent heartbeats. Definitions, units and abbreviations of APW-Ps evaluated from the APW are as explained in Supplementary Information FIGURE S1. Normotensive rats were anesthetized with Zoletil/xylazine.


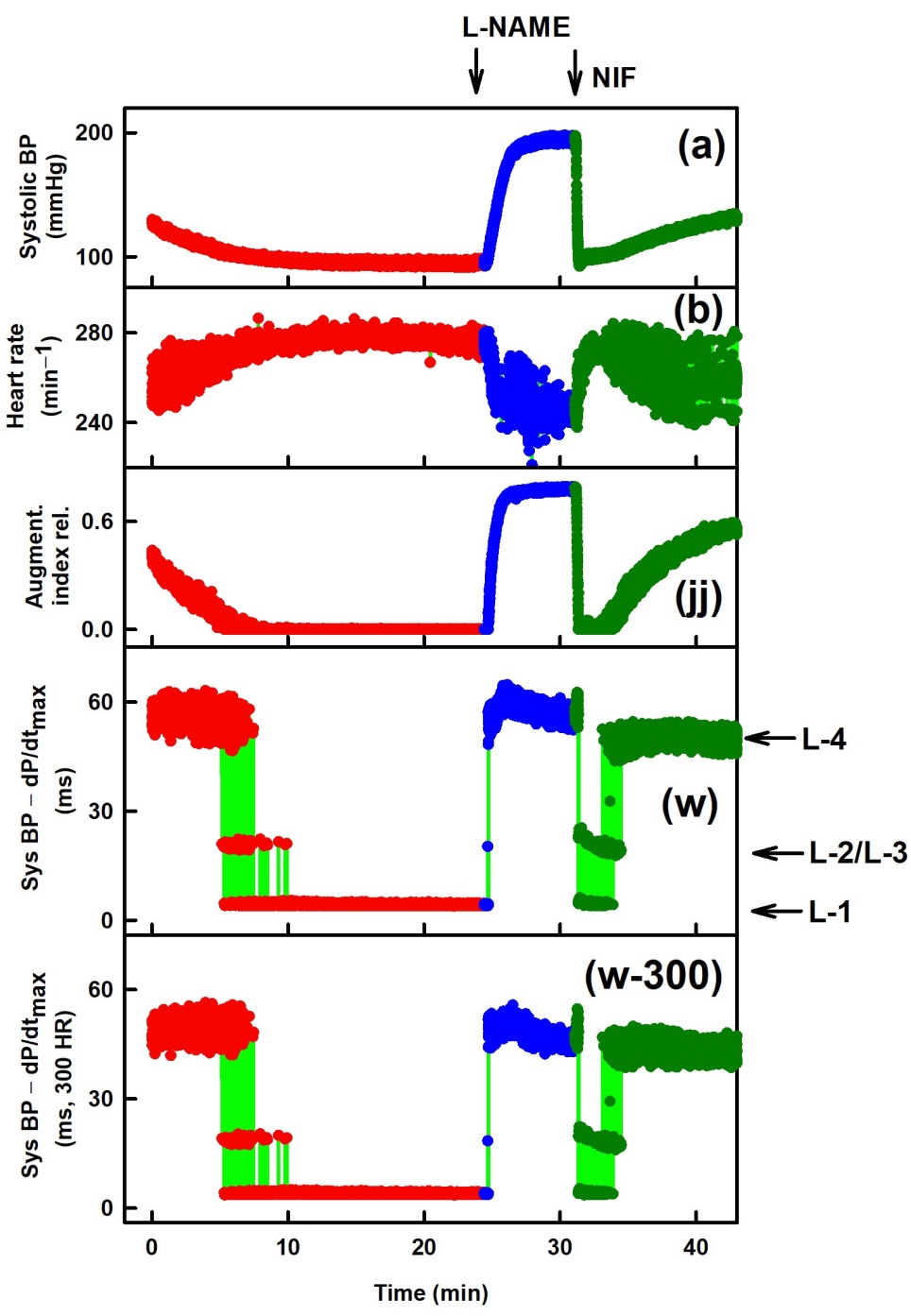


FIGURE S68 Exp-1. Time-dependent changes in APW-Ps, systolic BP (mmHg) (a), heart rate (min^–1^) (b), augmentation index (jj), systolic BP – dP/dt_max_ (ms) (w) and systolic BP – dP/dt_max_ (ms) (w-300) normalized to 300 min^–1^ HR in control **(**red**),** after the i.v. administration of of 15 mg kg^–1^ L-NAME **(**blue**)** and after subsequent administration of 400 nmol kg^–1^ of NIF (dark green). Horizontal arrows indicate predicted L-1 to L-4 levels. The green lines show the connection between adjacent heartbeats. Definitions, units and abbreviations of APW-Ps evaluated from the APW are as explained in Supplementary Information FIGURE S1. Normotensive rats were anesthetized with Zoletil/xylazine.


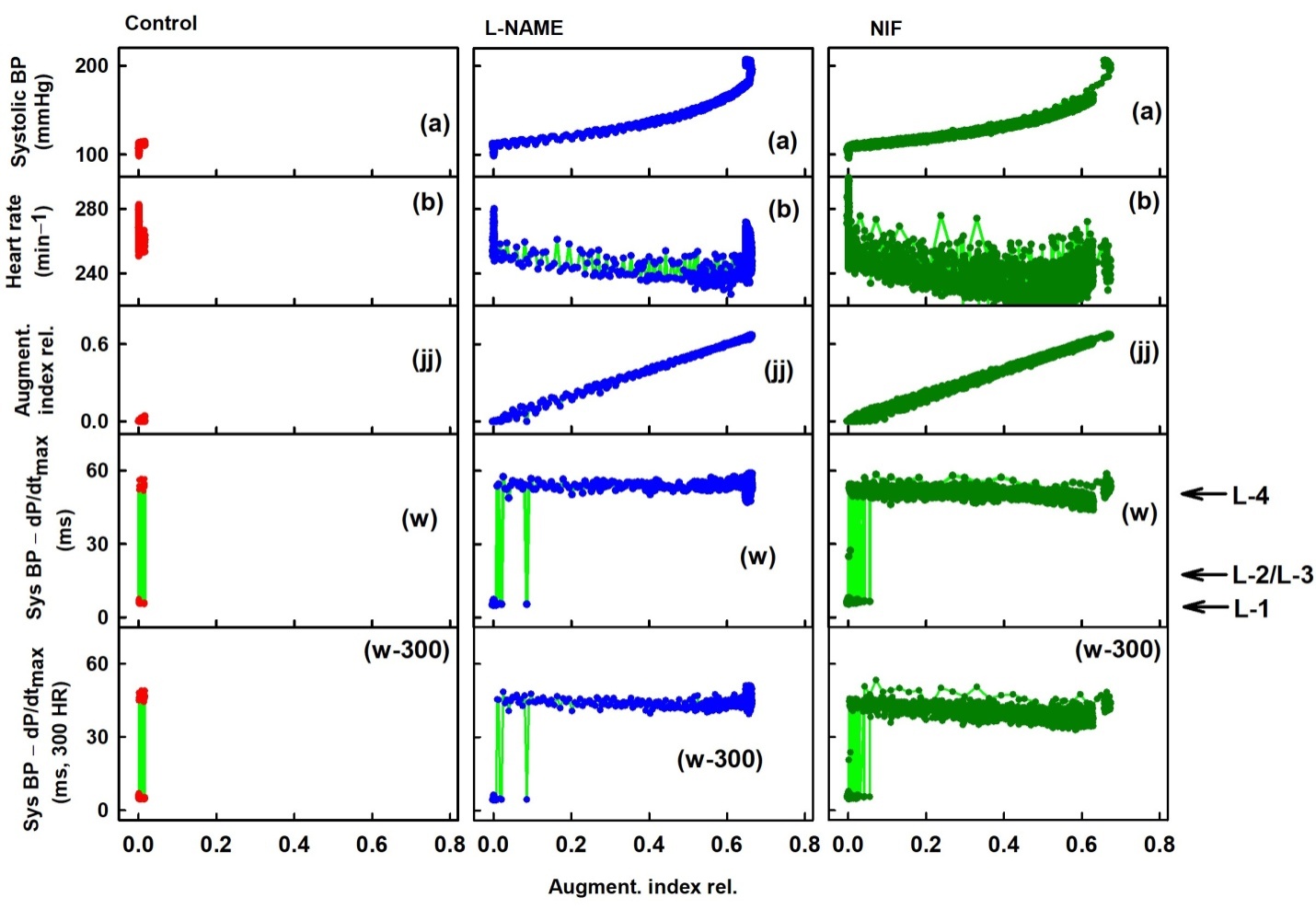


FIGURE S69 Exp-2. Cross-relationships of five APW-Ps to augmentation index in control (red heartbeats) and after the i.v. administration of of 15 mg kg^–1^ L-NAME (blue heartbeats) and after subsequent administration of 400 nmol kg^–1^ of NIF (dark green heartbeats). The green lines show the connection between adjacent heartbeats. Arrows indicate predicted L-1 to L-4 levels. Definitions, units and abbreviations of APW-Ps evaluated from the APW are as explained in Supplementary Information FIGURE S1. Normotensive rats were anesthetized with Zoletil/xylazine.


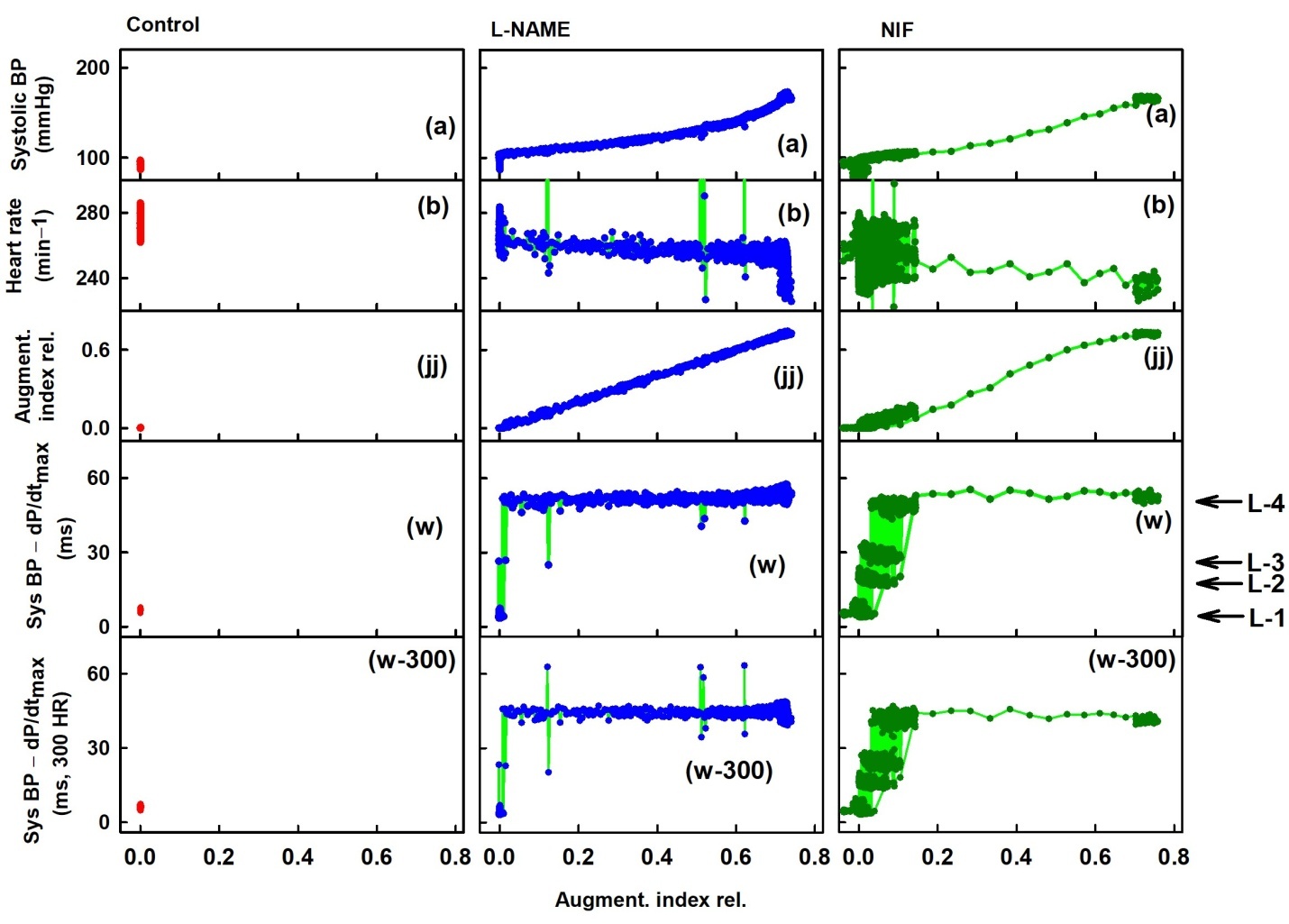


FIGURE S70 Exp-3. Cross-relationships of five APW-Ps to augmentation index in control (red heartbeats) and after the i.v. administration of of 15 mg kg^–1^ L-NAME (blue heartbeats) and after subsequent administration of 400 nmol kg^–1^ of NIF (dark green heartbeats). The green lines show the connection between adjacent heartbeats. Arrows indicate predicted L-1 to L-4 levels. Definitions, units and abbreviations of APW-Ps evaluated from the APW are as explained in Supplementary Information FIGURE S1. Normotensive rats were anesthetized with Zoletil/xylazine.


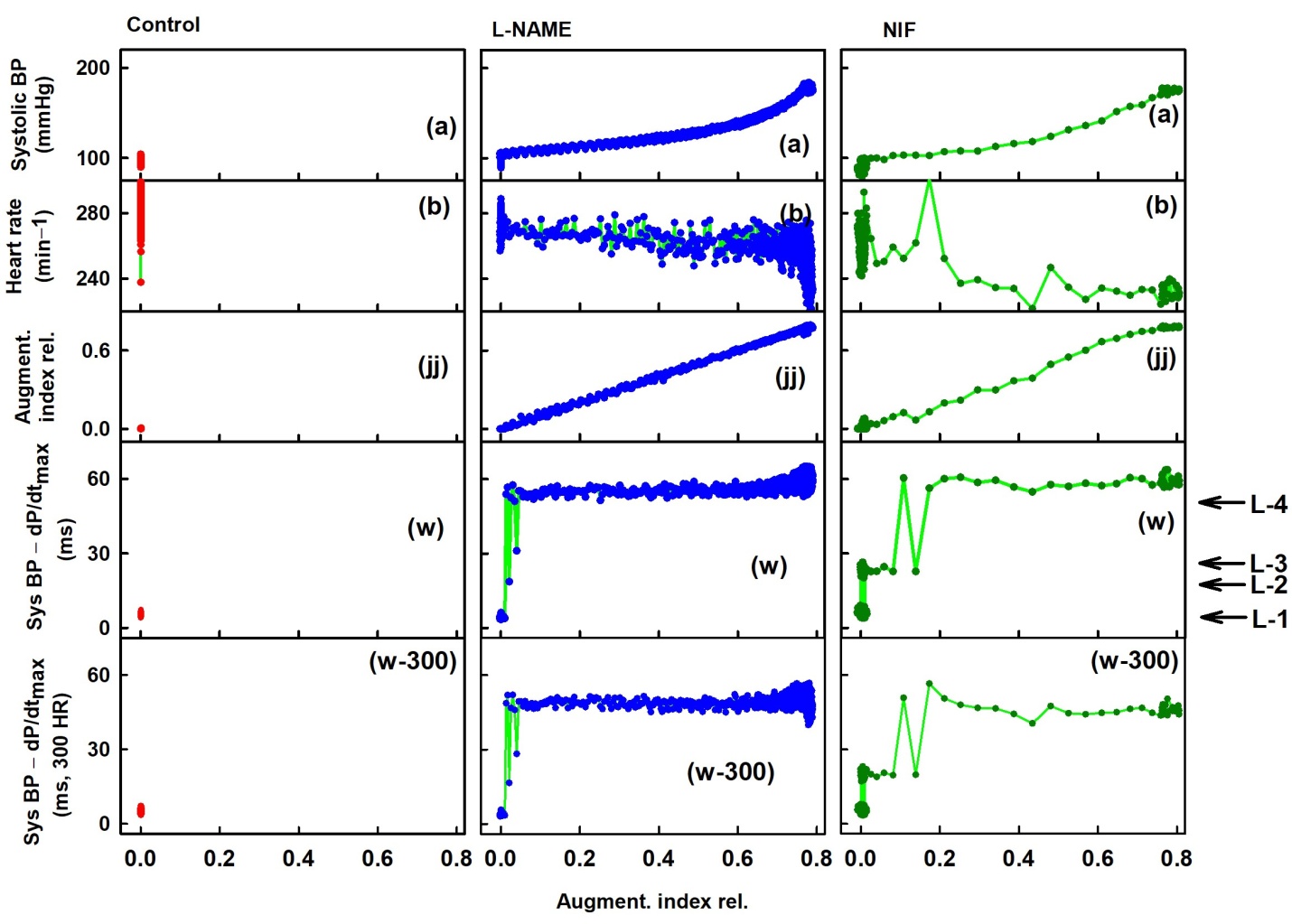


FIGURE S71 Exp-4. Cross-relationships of five APW-Ps to augmentation index in control (red heartbeats) and after the i.v. administration of of 15 mg kg^–1^ L-NAME (blue heartbeats) and after subsequent administration of 400 nmol kg^–1^ of NIF (dark green heartbeats). The green lines show the connection between adjacent heartbeats. Arrows indicate predicted L-1 to L-4 levels. Definitions, units and abbreviations of APW-Ps evaluated from the APW are as explained in Supplementary Information FIGURE S1. Normotensive rats were anesthetized with Zoletil/xylazine.


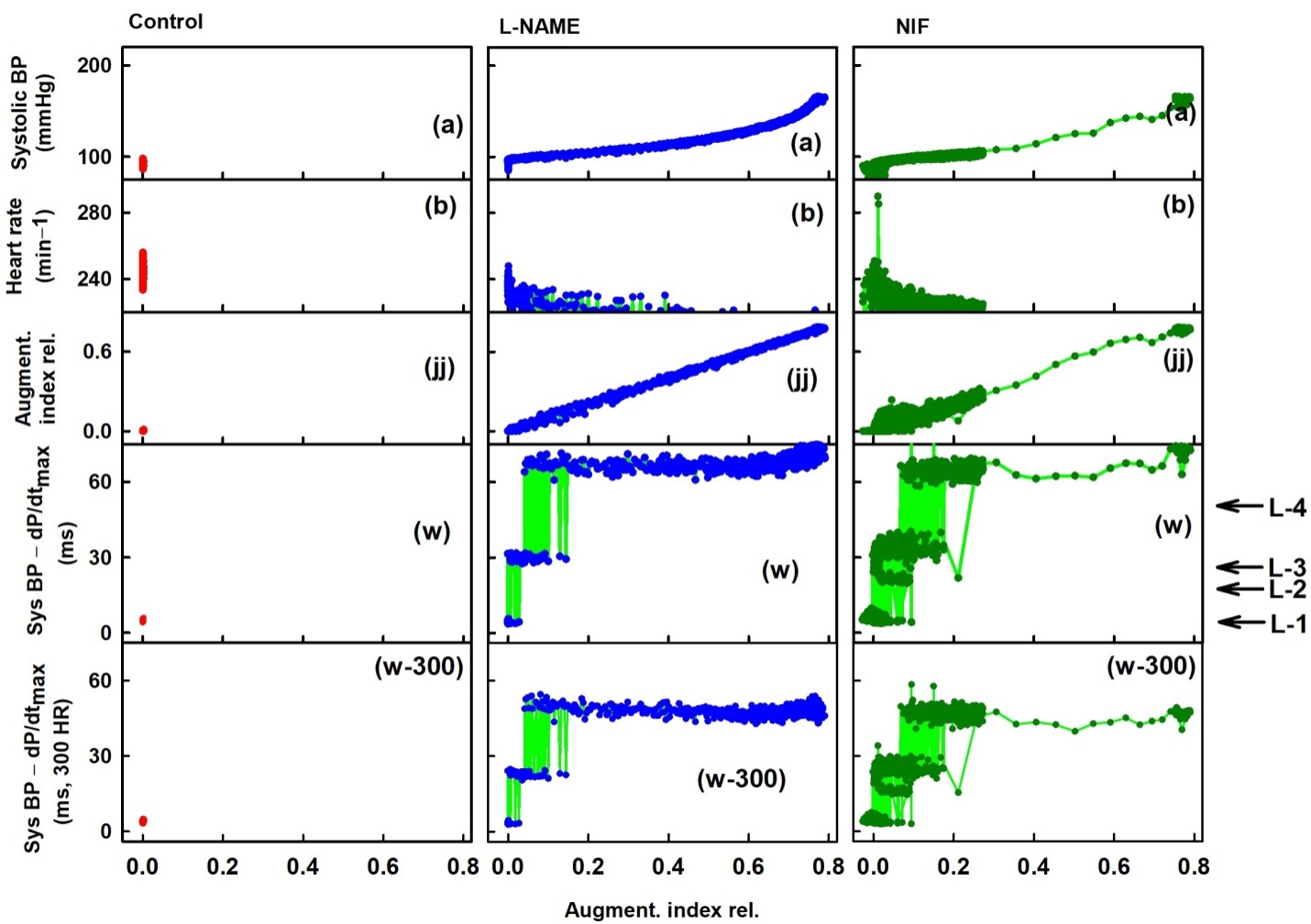


FIGURE S72 Exp-5. Cross-relationships of five APW-Ps to augmentation index in control (red heartbeats) and after the i.v. administration of of 15 mg kg^–1^ L-NAME (blue heartbeats) and after subsequent administration of 400 nmol kg^–1^ of NIF (dark green heartbeats). The green lines show the connection between adjacent heartbeats. Arrows indicate predicted L-1 to L-4 levels. Definitions, units and abbreviations of APW-Ps evaluated from the APW are as explained in Supplementary Information FIGURE S1. Normotensive rats were anesthetized with Zoletil/xylazine.


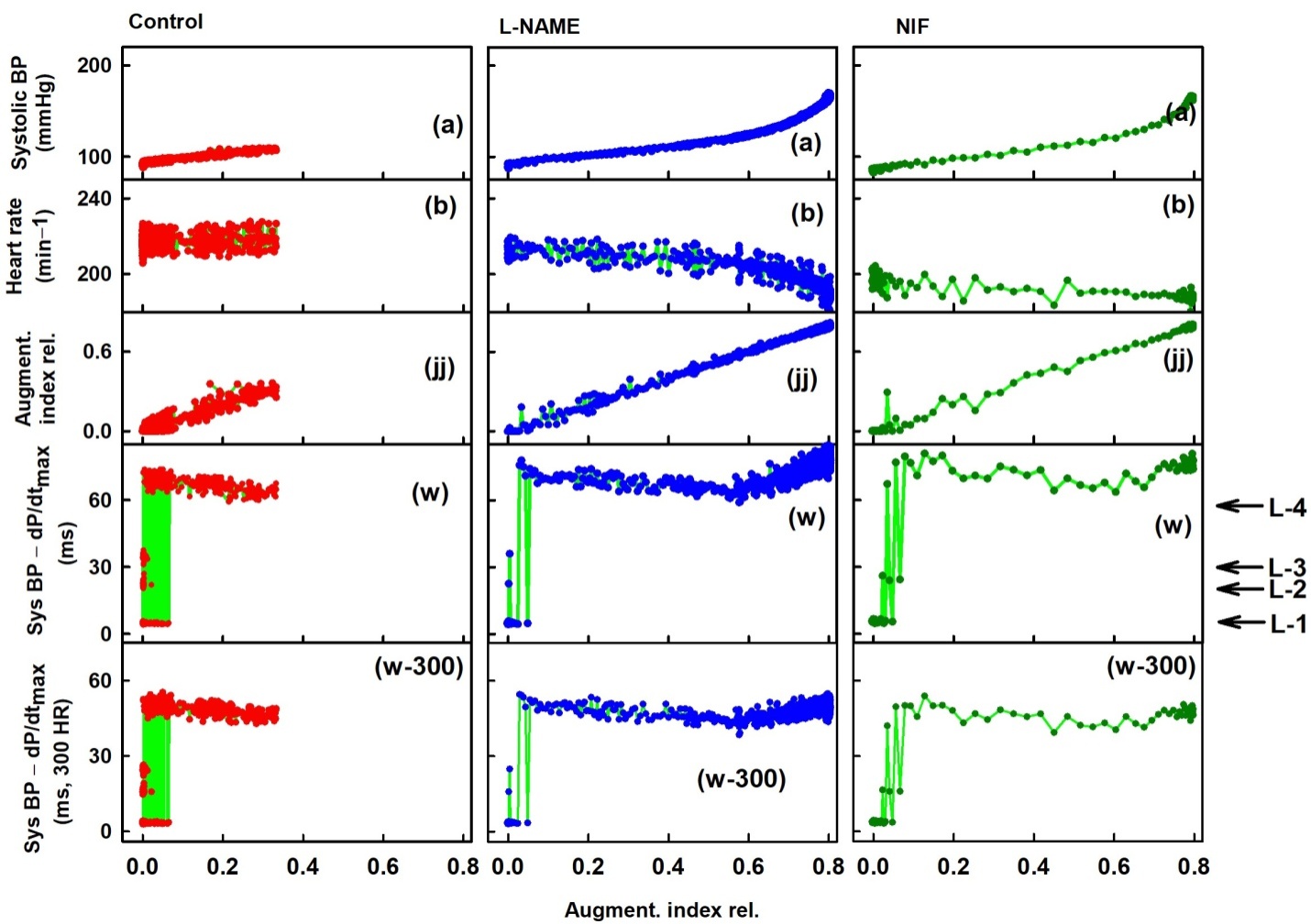


FIGURE S73 Exp-6. Cross-relationships of five APW-Ps to augmentation index in control (red heartbeats) and after the i.v. administration of of 15 mg kg^–1^ L-NAME (blue heartbeats) and after subsequent administration of 400 nmol kg^–1^ of NIF (dark green heartbeats). The green lines show the connection between adjacent heartbeats. Arrows indicate predicted L-1 to L-4 levels. Definitions, units and abbreviations of APW-Ps evaluated from the APW are as explained in Supplementary Information FIGURE S1. Normotensive rats were anesthetized with Zoletil/xylazine.


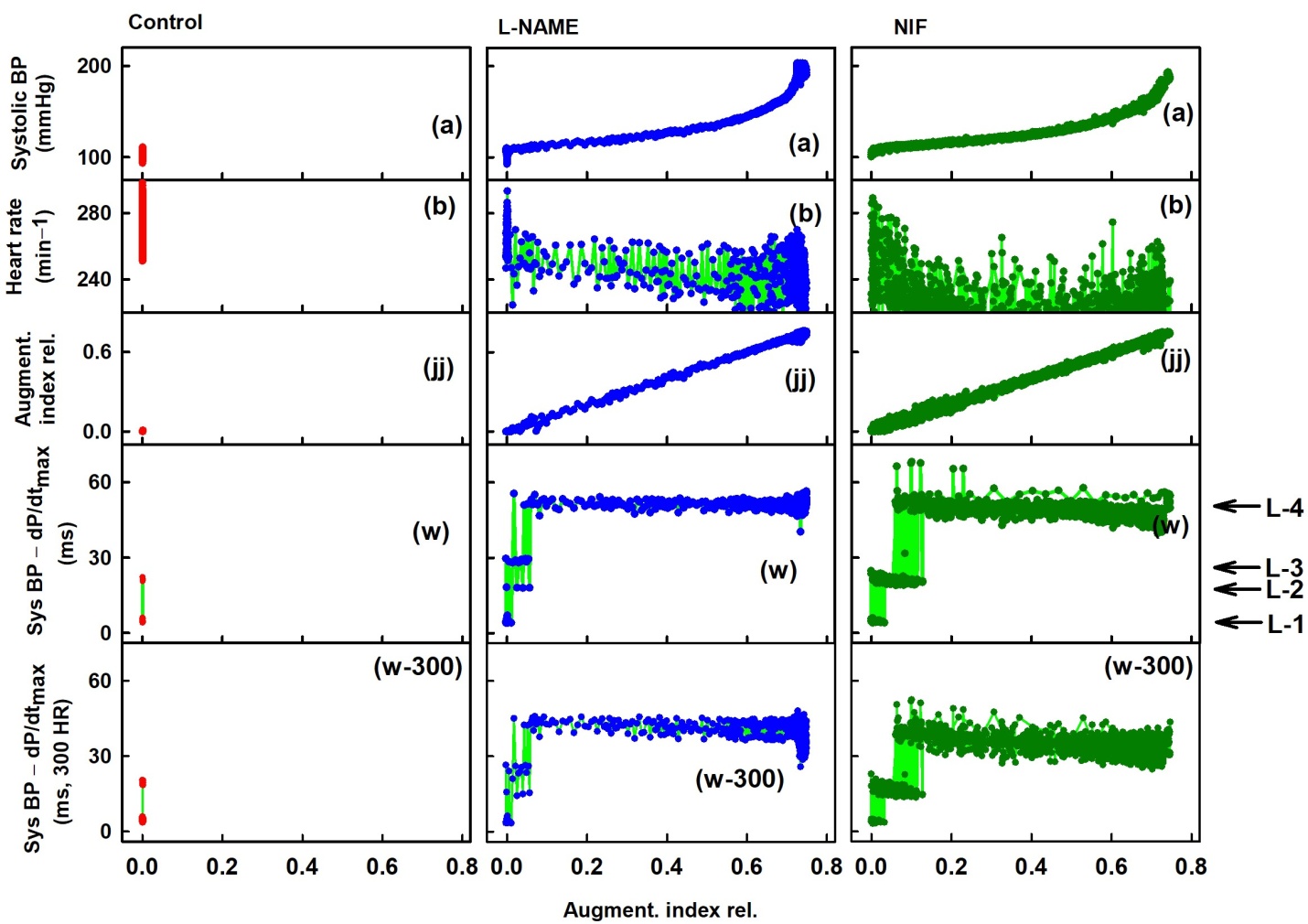


FIGURE S74 Exp-7. Cross-relationships of five APW-Ps to augmentation index in control (red heartbeats) and after the i.v. administration of of 15 mg kg^–1^ L-NAME (blue heartbeats) and after subsequent administration of 400 nmol kg^–1^ of NIF (dark green heartbeats). The green lines show the connection between adjacent heartbeats. Arrows indicate predicted L-1 to L-4 levels. Definitions, units and abbreviations of APW-Ps evaluated from the APW are as explained in Supplementary Information FIGURE S1. Normotensive rats were anesthetized with Zoletil/xylazine.


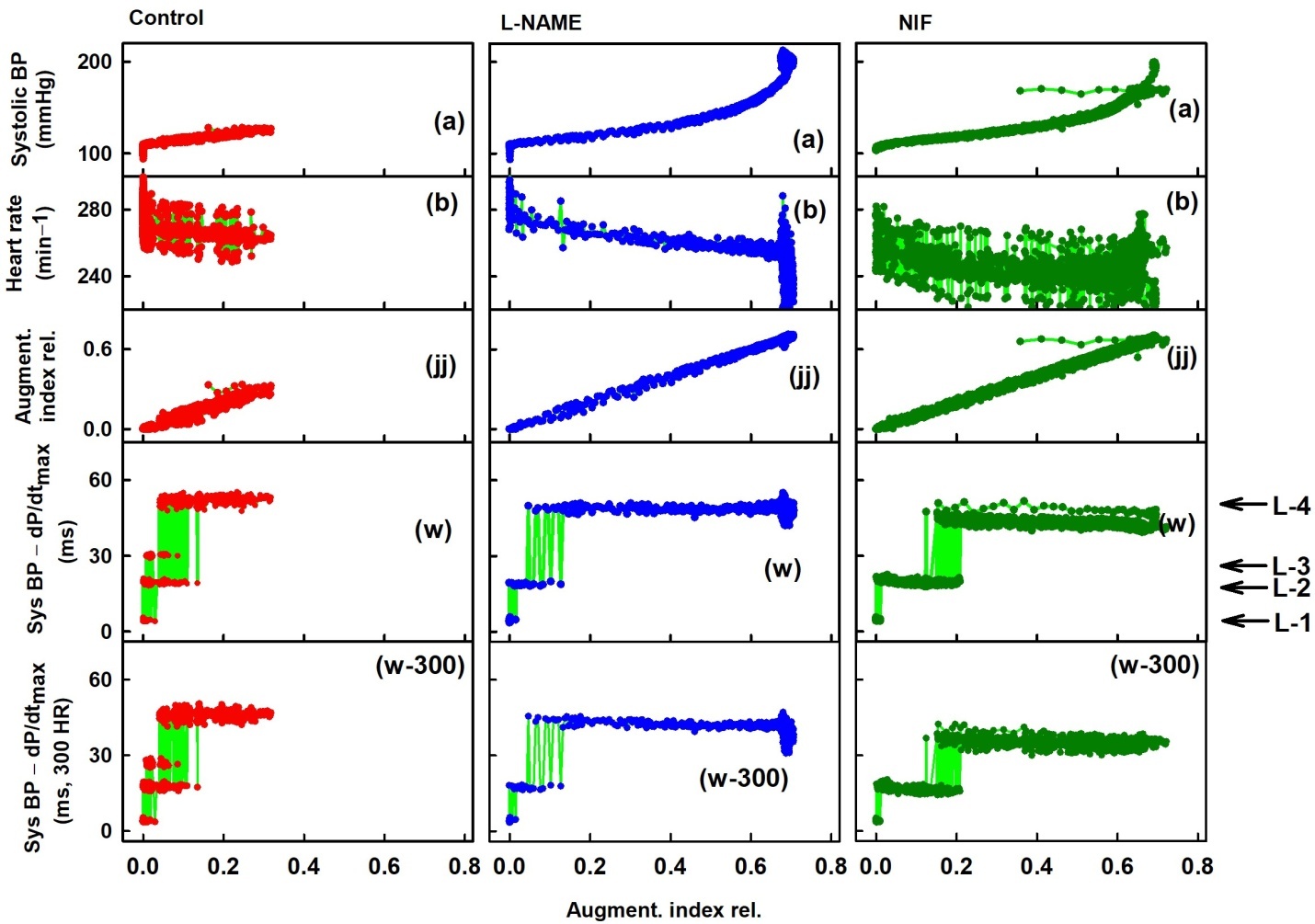


FIGURE S75 Exp-8. Cross-relationships of five APW-Ps to augmentation index in control (red heartbeats) and after the i.v. administration of of 15 mg kg^–1^ L-NAME (blue heartbeats) and after subsequent administration of 400 nmol kg^–1^ of NIF (dark green heartbeats). The green lines show the connection between adjacent heartbeats. Arrows indicate predicted L-1 to L-4 levels. Definitions, units and abbreviations of APW-Ps evaluated from the APW are as explained in Supplementary Information FIGURE S1. Normotensive rats were anesthetized with Zoletil/xylazine.


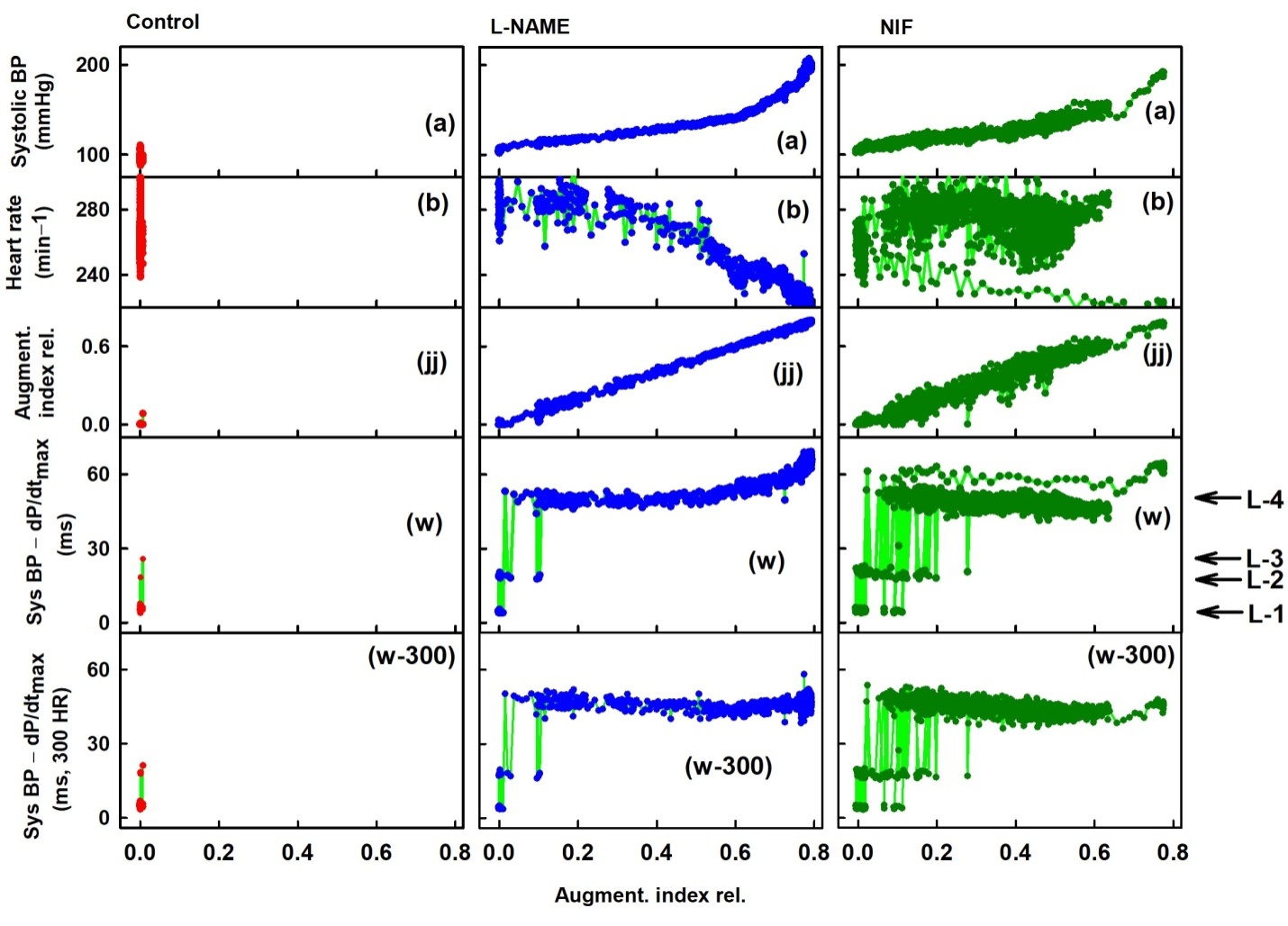


FIGURE S76 Exp-9. Cross-relationships of five APW-Ps to augmentation index in control (red heartbeats) and after the i.v. administration of of 15 mg kg^–1^ L-NAME (blue heartbeats) and after subsequent administration of 400 nmol kg^–1^ of NIF (dark green heartbeats). The green lines show the connection between adjacent heartbeats. Arrows indicate predicted L-1 to L-4 levels. Definitions, units and abbreviations of APW-Ps evaluated from the APW are as explained in Supplementary Information FIGURE S1. Normotensive rats were anesthetized with Zoletil/xylazine.


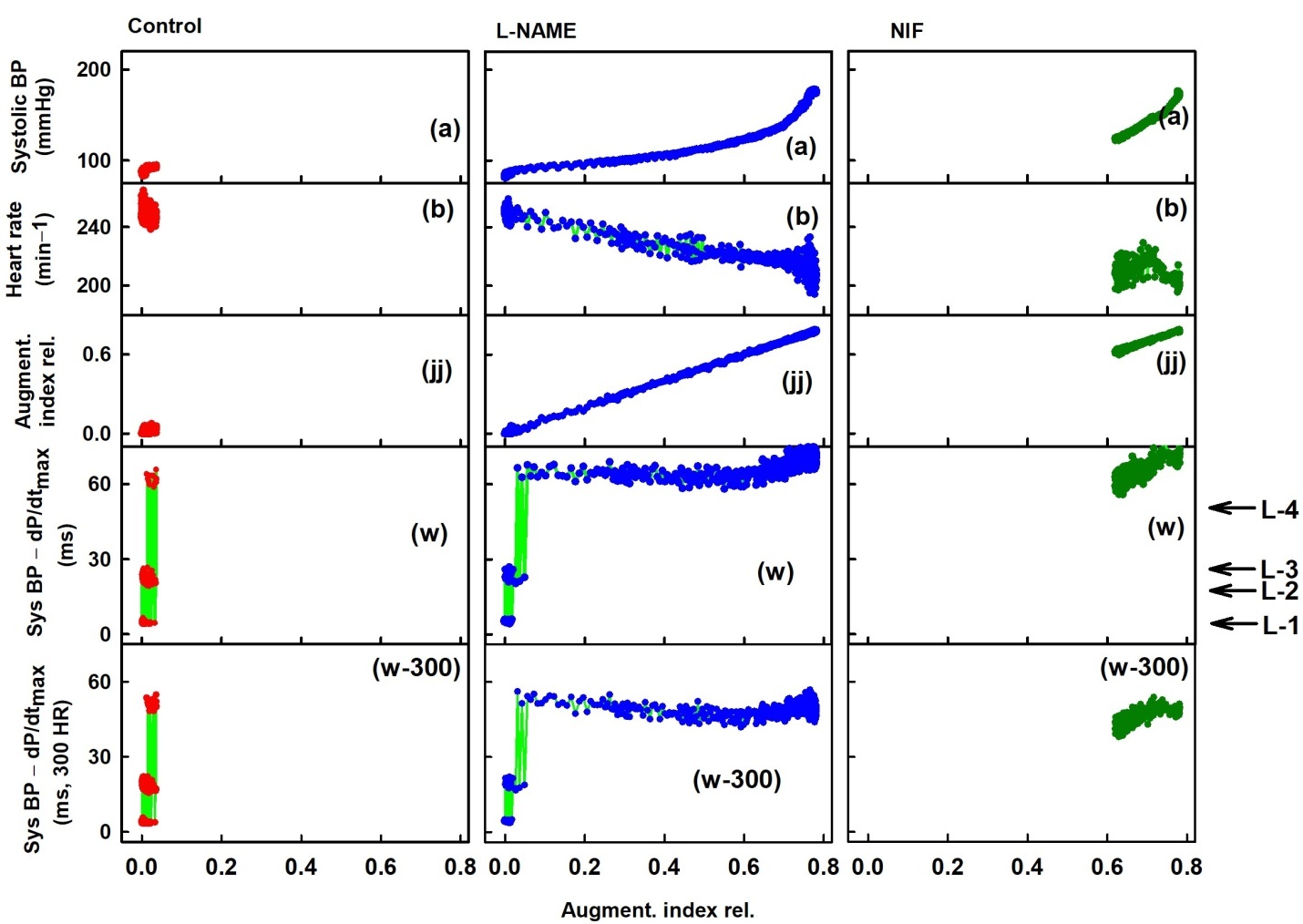


FIGURE S77 Exp-10. Cross-relationships of five APW-Ps to augmentation index in control (red heartbeats) and after the i.v. administration of of 15 mg kg^–1^ L-NAME (blue heartbeats) and after subsequent administration of 400 nmol kg^–1^ of NIF (dark green heartbeats). The green lines show the connection between adjacent heartbeats. Arrows indicate predicted L-1 to L-4 levels. Definitions, units and abbreviations of APW-Ps evaluated from the APW are as explained in Supplementary Information FIGURE S1. Normotensive rats were anesthetized with Zoletil/xylazine.

**
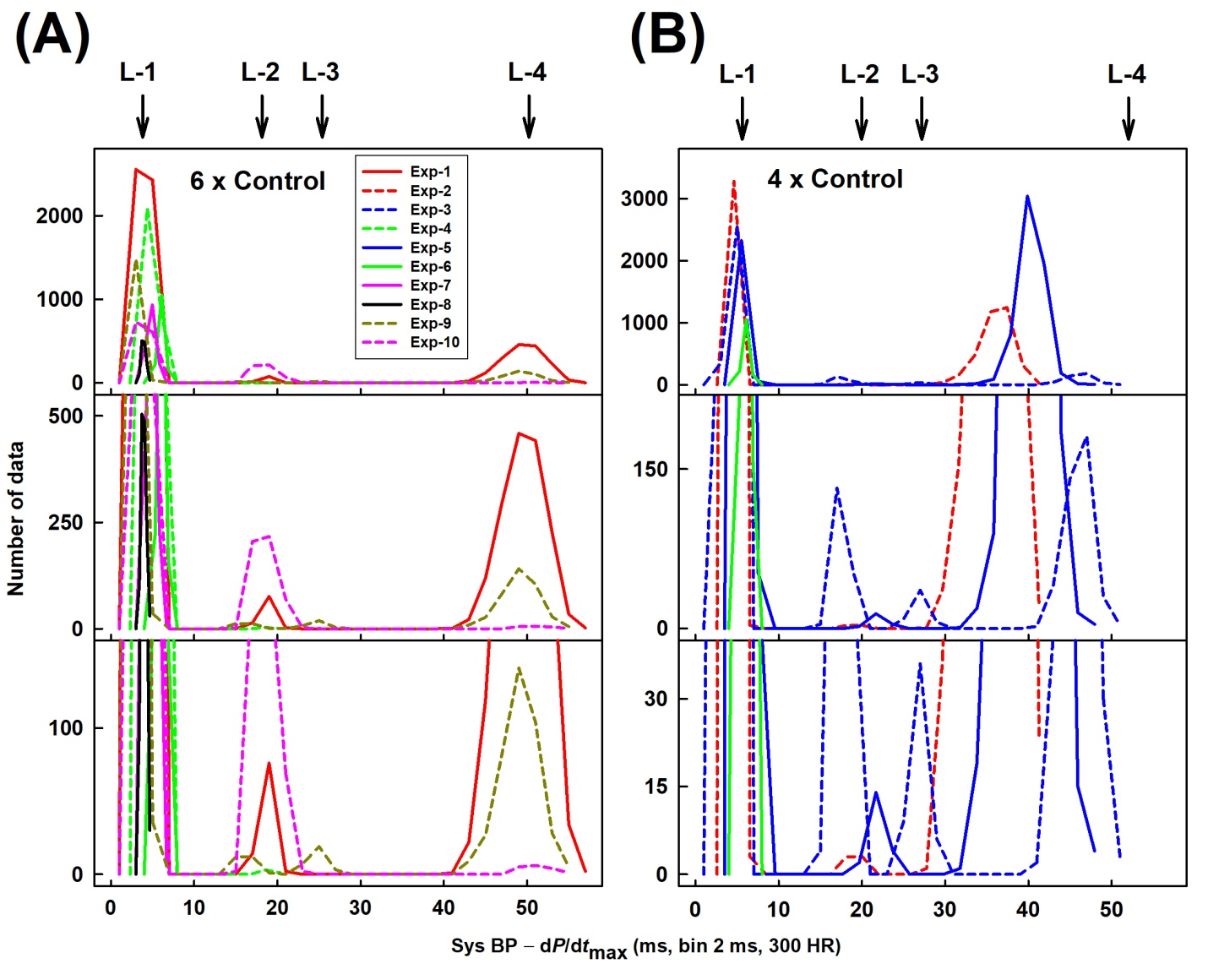
**

FIGURE S78 Histograms of time distance of (systolic BP – d*P*/d*t*_max_) for L-levels normalized to 300 min^–1^ HR in controls. (A) Selected six experiments in which histograms were approximately similar. (B) The rest four experiments in which histograms were not similar. Histograms are at three resolutions. Arrows indicate predicted levels L-1 to L-4. Colors and lines for ten experiments are the same as in FIGURES S57B,S58,S78-S80. Normotensive rats were anesthetized with Zoletil/xylazine.

**
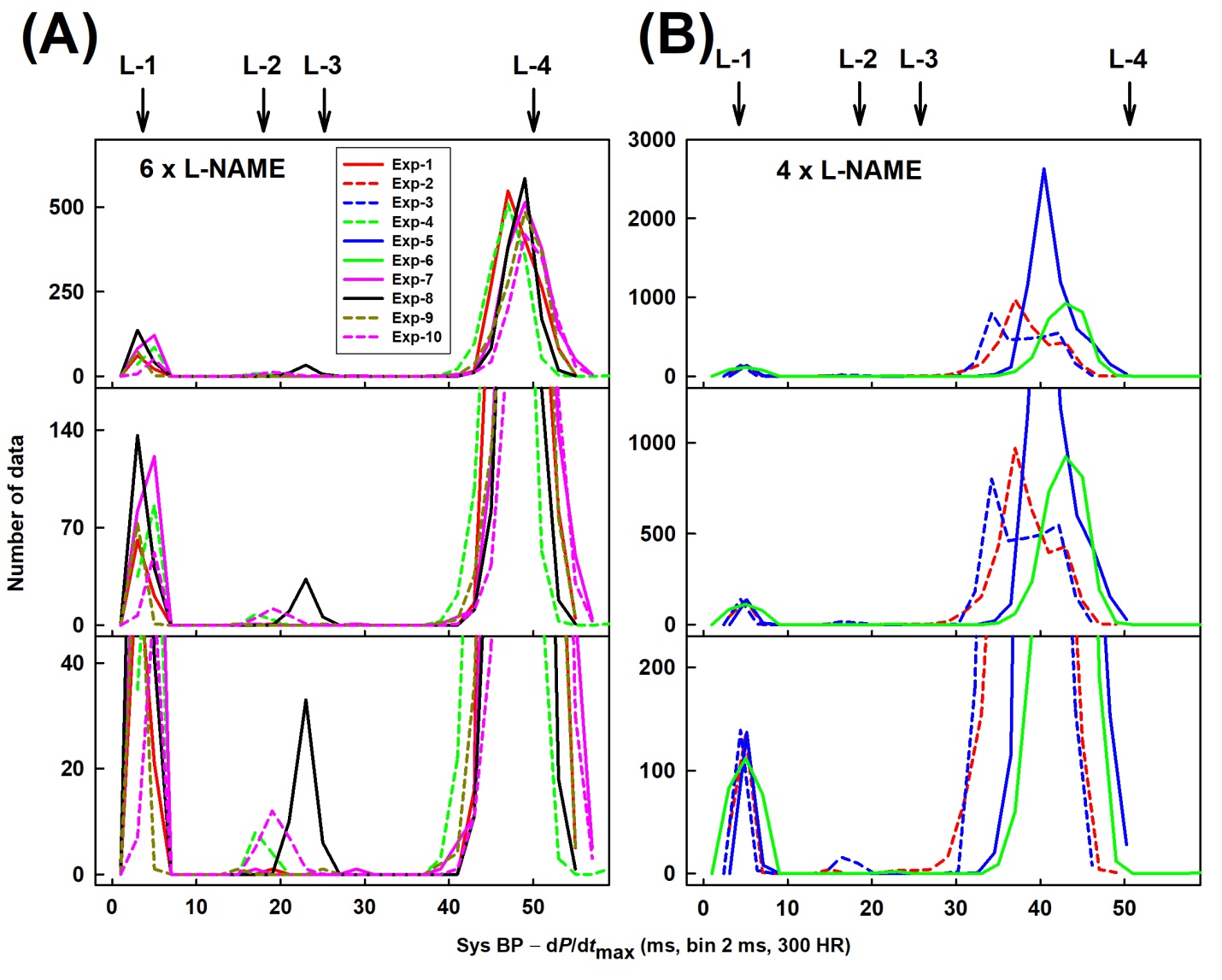
**

FIGURE S79 Histograms of time distance of (systolic BP – d*P*/d*t*_max_) for L-levels normalized to 300 min^–1^ HR after the i.v. administration of of 15 mg kg^–1^ L-NAME. (A) Selected six experiments in which histograms were approximately similar. (B) The rest four experiments in which histograms were not similar. Histograms are at three resolutions. Arrows indicate predicted levels L-1 to L-4 as they were predicted in controls. Colors and lines for ten experiments are the same as in FIGURES S57B,S58,S78-S80. Normotensive rats were anesthetized with Zoletil/xylazine.

**
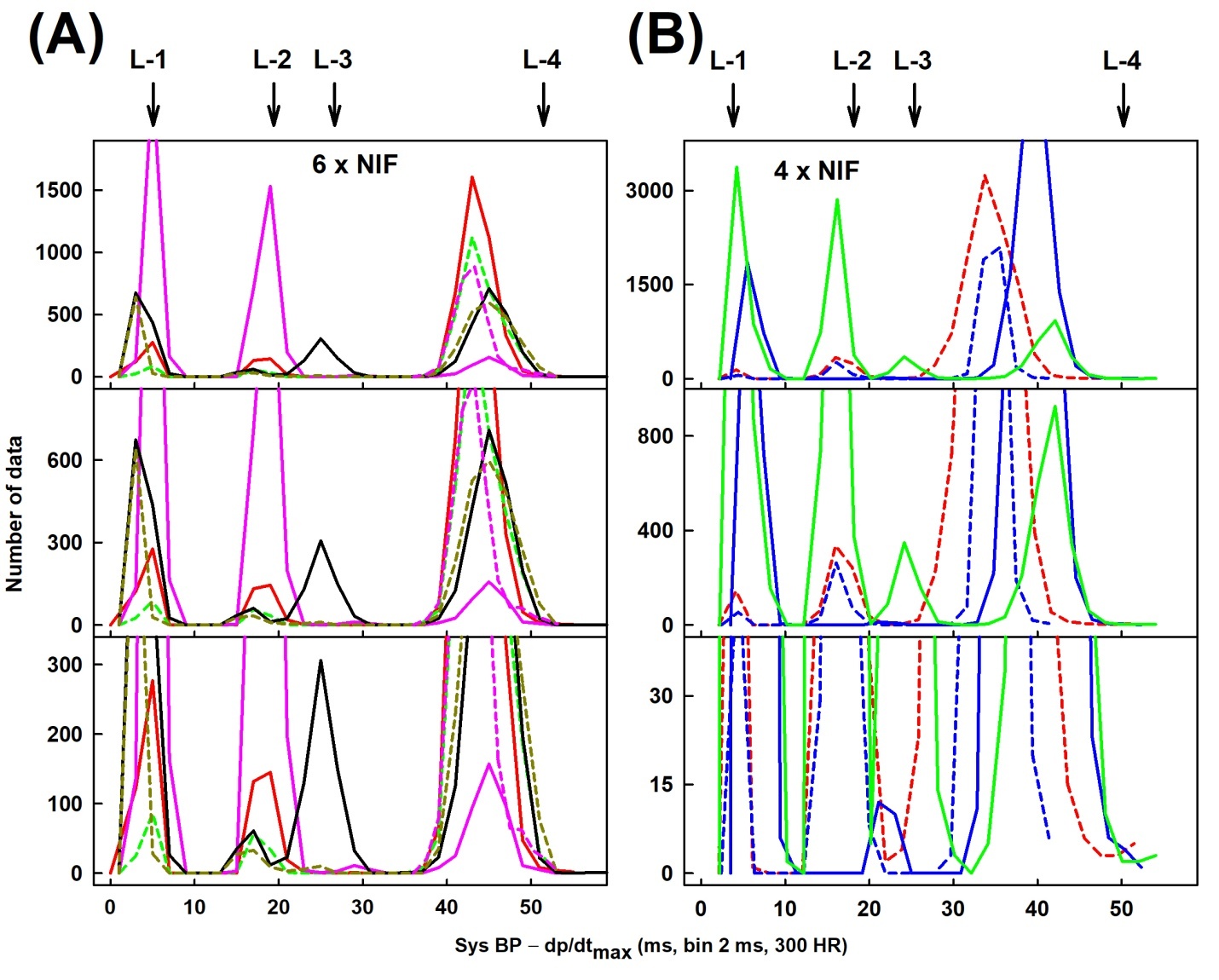
**

FIGURE S80 Histograms of time distance of (systolic BP – d*P*/d*t*_max_) for L-levels normalized to 300 min^–1^ HR in the presence of 15 mg kg^–1^ L-NAME and after subsequent administration of 200 or 400 nmol kg^–1^ of NIF. (A) Selected six experiments in which histograms were approximately similar. (B) The rest four experiments in which histograms were not similar. Histograms are at three resolutions. Arrows indicate predicted levels L-1 to L-4 as they were predicted in controls. Colors and lines for ten experiments are the same as in FIGURES S57B,S58,S78-S80. Normotensive rats were anesthetized with Zoletil/xylazine.


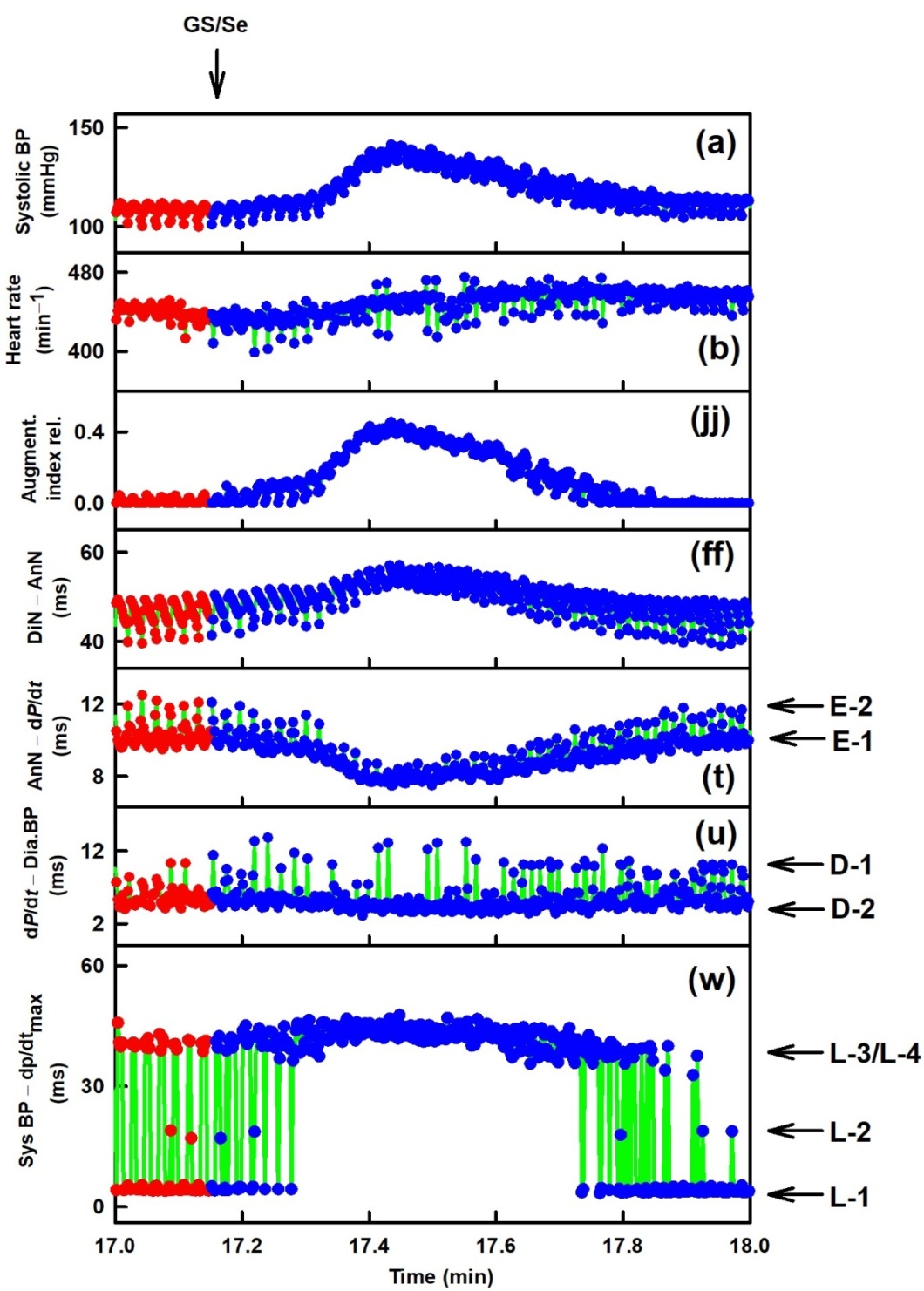


FIGURE S81 ExN-1. Time-dependent changes in APW-Ps of isoflurene anesthetized normotensive rat. Control (red heartbeats) and after i.v. administration of GS/Se (75/12.5 in µmol L^–1^, blue heartbeats). Horizontal arrows indicate predicted D-1 and D-2 levels, E-1 and E-2 levels and L-1 to L-4 levels. The green lines show the connection between adjacent heartbeats. Definitions, units and abbreviations of APW-Ps evaluated from the APW are as explained in Supplementary Information FIGURE S1.

**
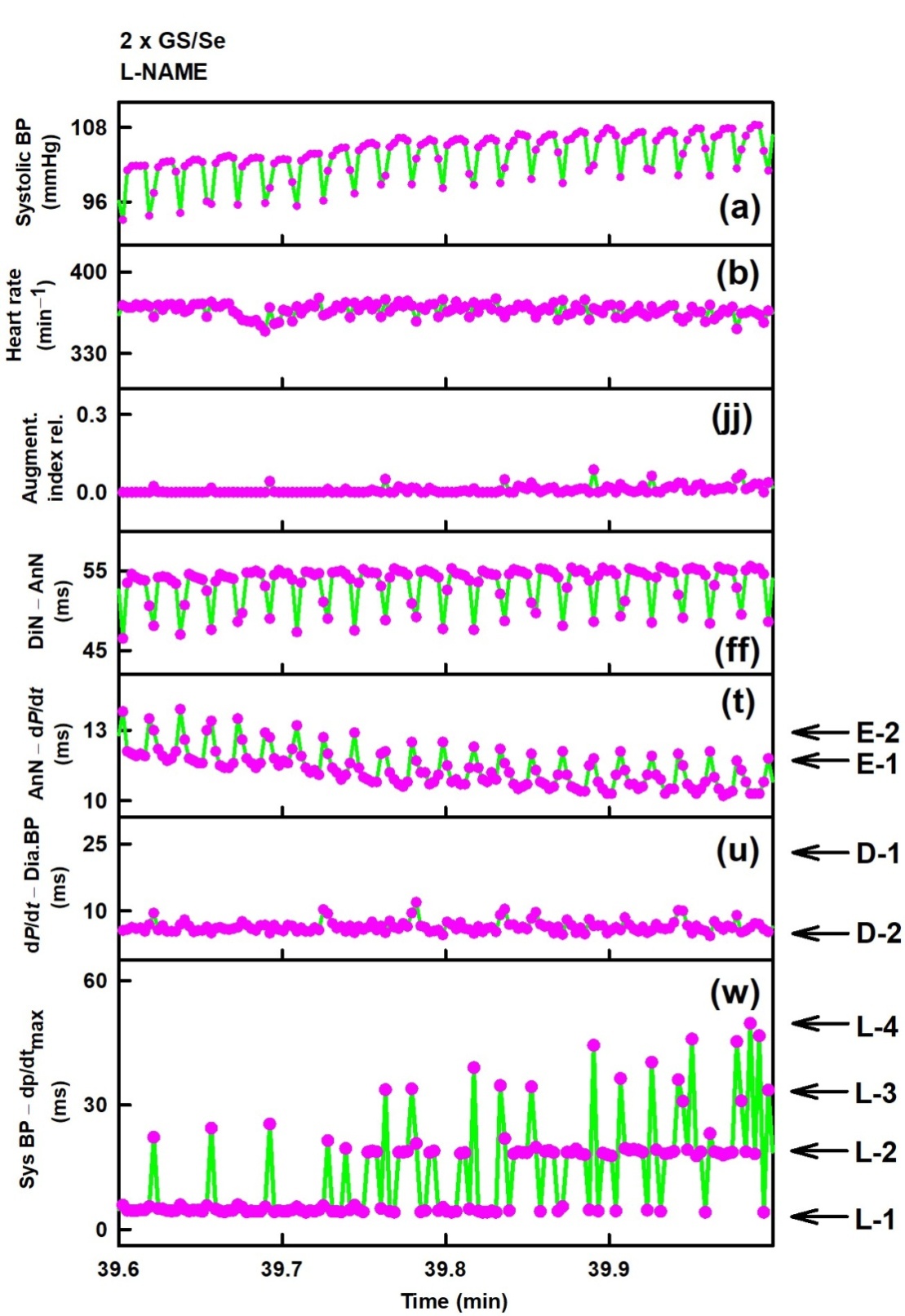
**

FIGURE S82 ExN-1. Time-dependent changes in APW-Ps of isoflurene anesthetized normotensive rat in the presence of subsequent administration of twice GS/Se (75/12.5 in µmol L^–1^) followed by 30 mg kg^–1^ L-NAME (pink heartbeats). Horizontal arrows indicate predicted D-1 and D-2 levels, E-1 and E-2 levels and L-1 to L-4 levels. The green lines show the connection between adjacent heartbeats. Definitions, units and abbreviations of APW-Ps evaluated from the APW are as explained in Supplementary Information FIGURE S1.


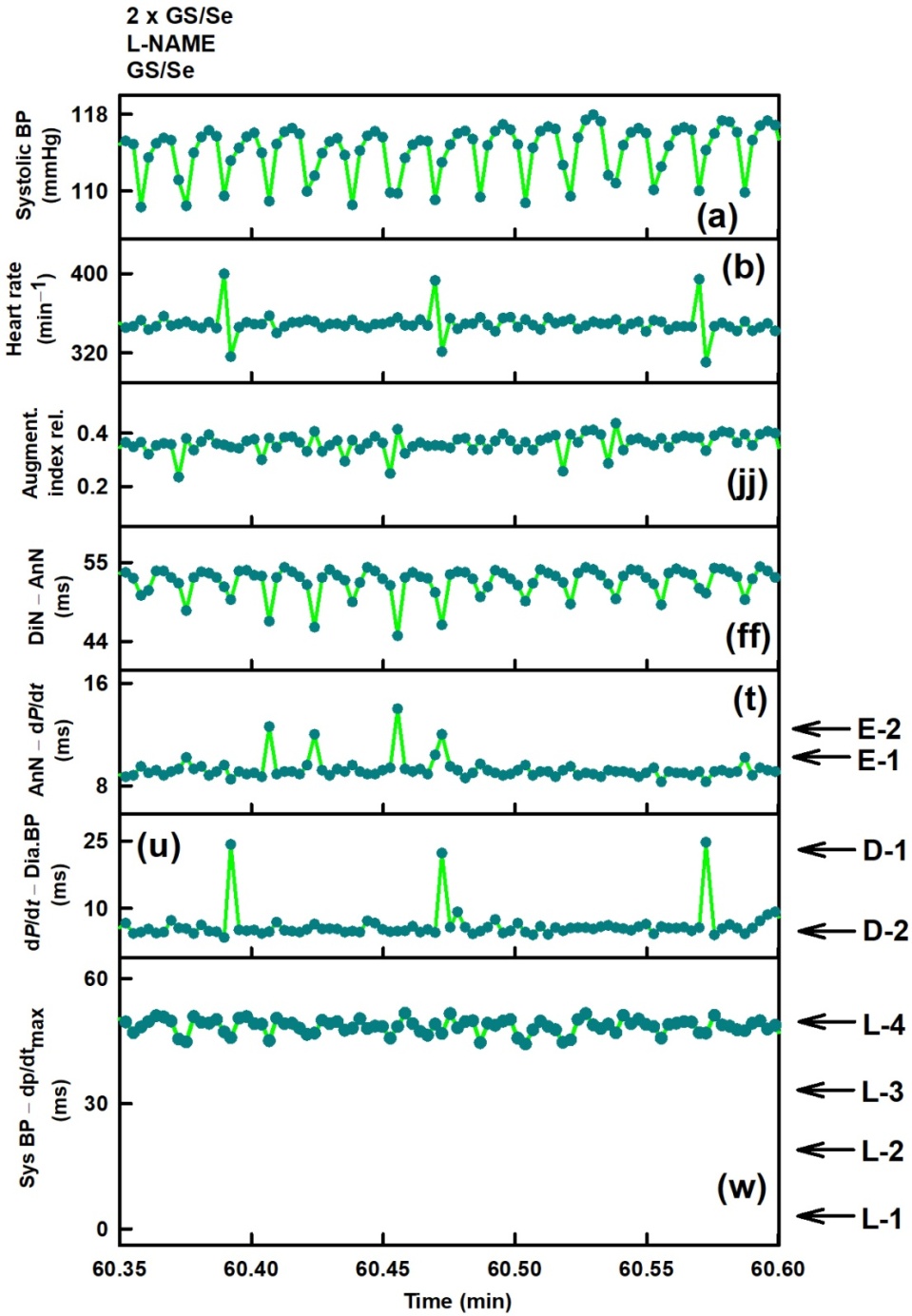


FIGURE S83 ExN-1. Time-dependent changes in APW-Ps of isoflurene anesthetized normotensive rat in the presence of subsequent administration of twice GS/Se (75/12.5 in µmol L^–1^), 30 mg kg^–1^ L-NAME followed by GS/Se (75/12.5 in µmol L^–1^, dark cyan heartbeats). Horizontal arrows indicate predicted D-1 and D-2 levels, E-1 and E-2 levels and L-1 to L-4 levels. The green lines show the connection between adjacent heartbeats. Definitions, units and abbreviations of APW-Ps evaluated from the APW are as explained in Supplementary Information FIGURE S1.


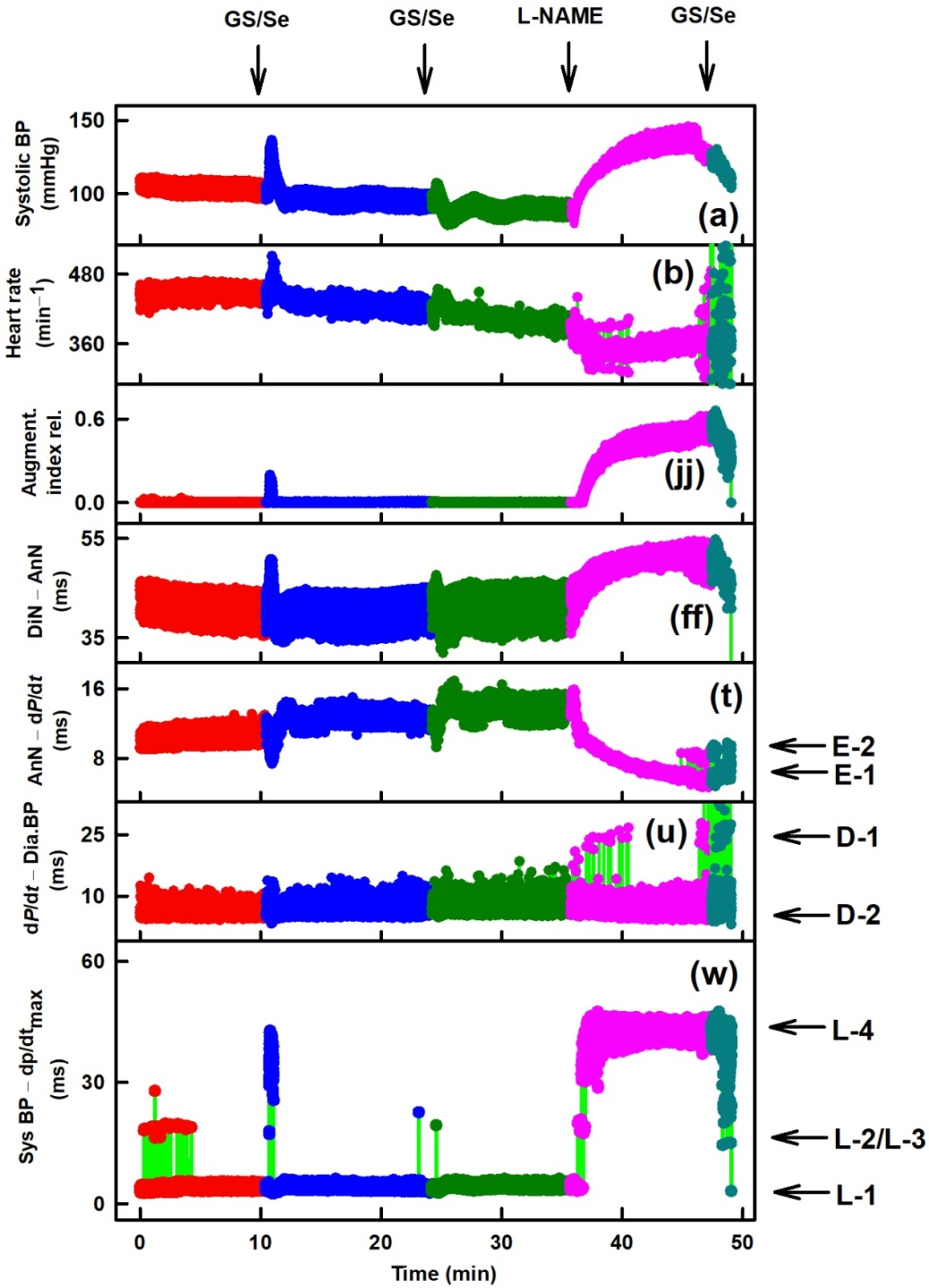


FIGURE S84 ExN-2. Time-dependent changes in APW-Ps of isoflurene anesthetized normotensive rat in control (red heartbeats) and after the subsequent administration of twice GS/Se (75/12.5 in µmol L^–1^, blue and dark green heartbeats), 30 mg kg^–1^ L-NAME (pink heartbeats) followed by GS/Se (75/12.5 in µmol L^–1^, dark cyan heartbeats). Horizontal arrows indicate predicted D-1 and D-2 levels, E-1 and E-2 levels and L-1 to L-4 levels. The green lines show the connection between adjacent heartbeats. Definitions, units and abbreviations of APW-Ps evaluated from the APW are as explained in Supplementary Information FIGURE S1.

**
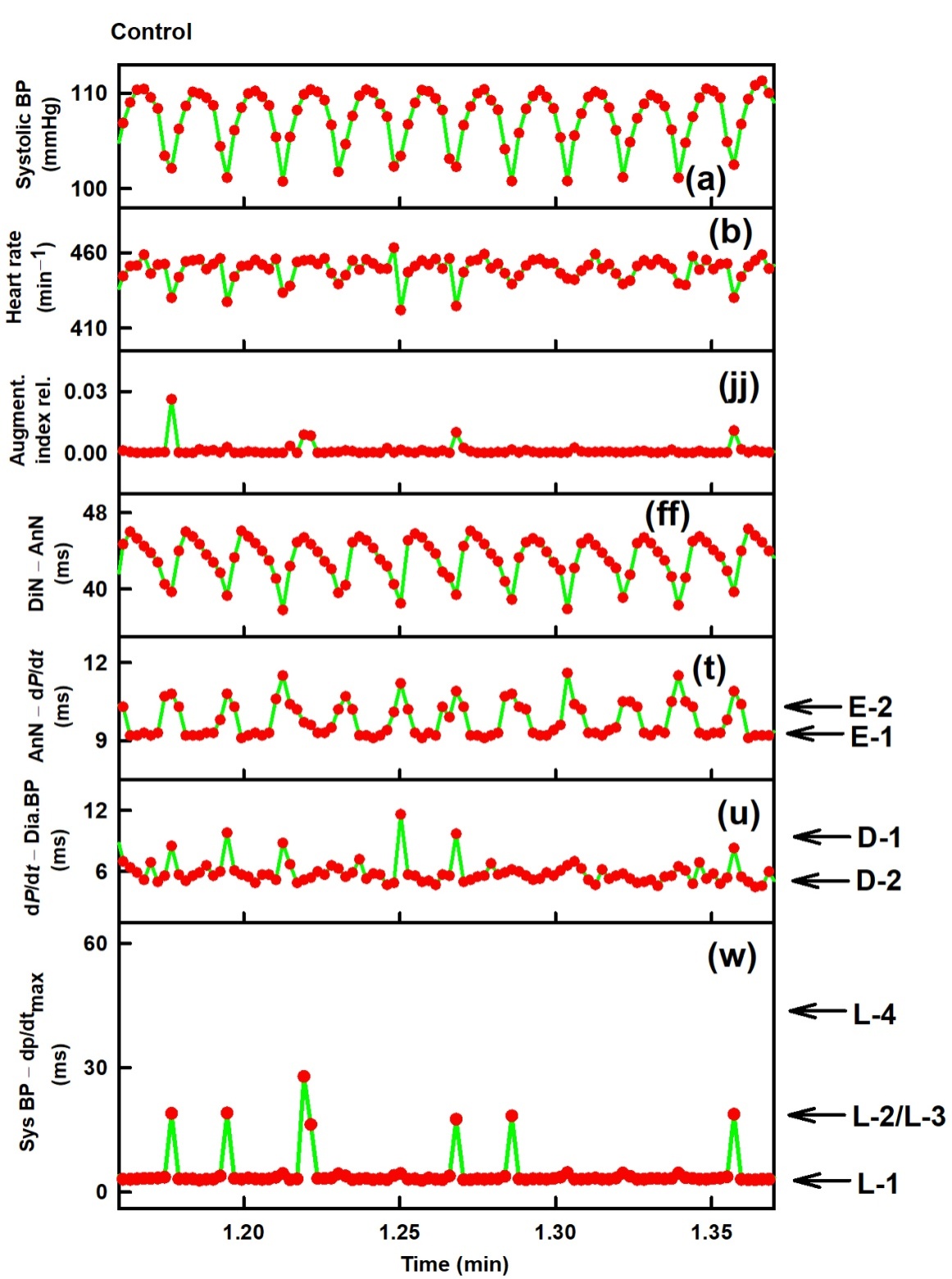
**

FIGURE S85 ExN-2. Time-dependent changes in APW-Ps of isoflurene anesthetized normotensive rat in control (red heartbeats). Horizontal arrows indicate predicted D-1 and D-2, E-1 and E-2 levels and L-1 to L-4 levels. The green lines show the connection between adjacent heartbeats. Definitions, units and abbreviations of APW-Ps evaluated from the APW are as explained in Supplementary Information FIGURE S1.


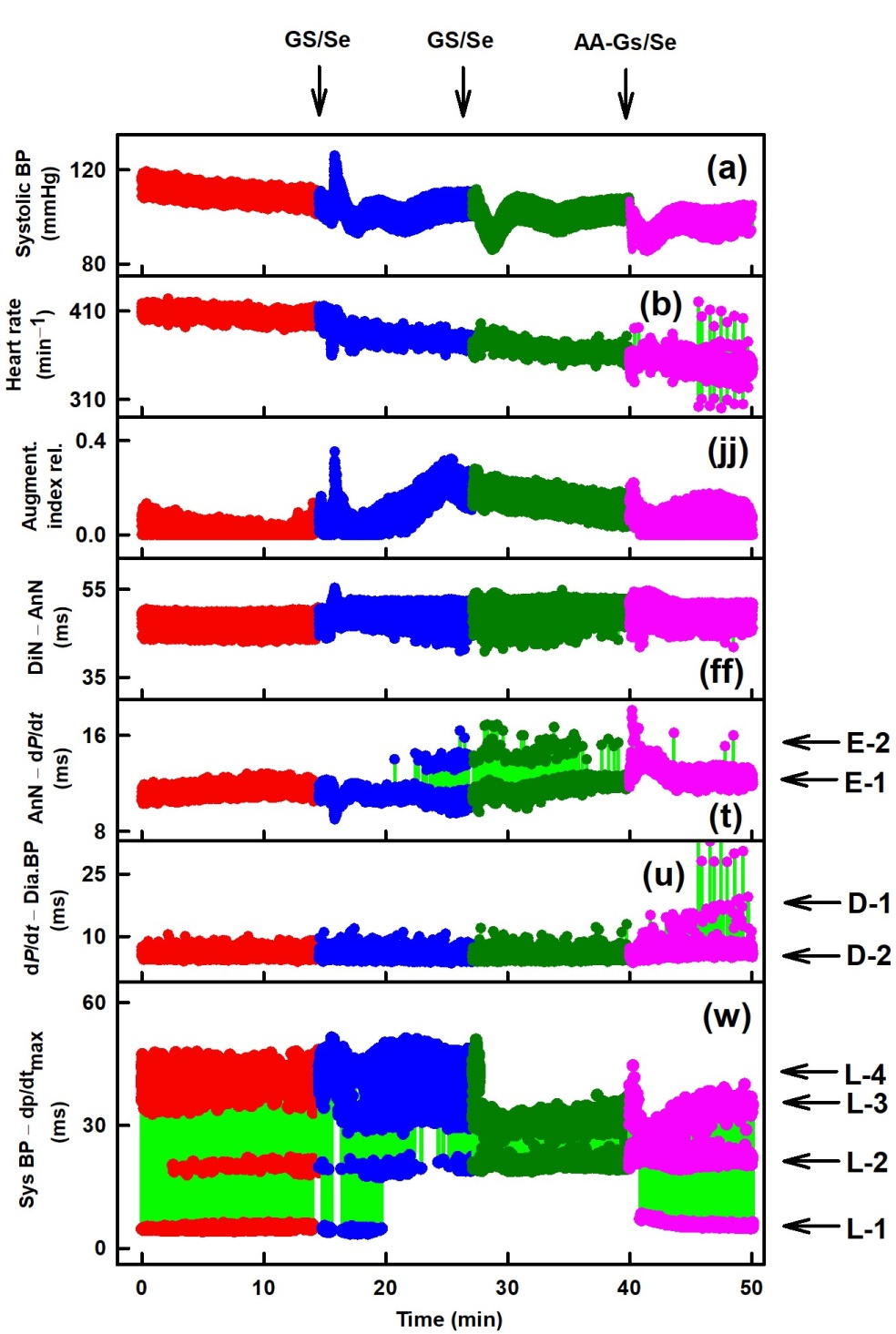


FIGURE S86 ExN-3. Time-dependent changes in APW-Ps of isoflurene anesthetized normotensive rat in control (red heartbeats) and after the subsequent administration of twice GS/Se (75/12.5 in µmol L^–1^, blue and dark green heartbeats), 30 mg kg^–1^ L-NAME (pink heartbeats) followed by GS/Se (75/12.5 in µmol L^–1^, dark cyan heartbeats). Horizontal arrows indicate predicted D-1 and D-2 levels, E-1 and E-2 levels and L-1 to L-4 levels. The green lines show the connection between adjacent heartbeats. Definitions, units and abbreviations of APW-Ps evaluated from the APW are as explained in Supplementary Information FIGURE S1.


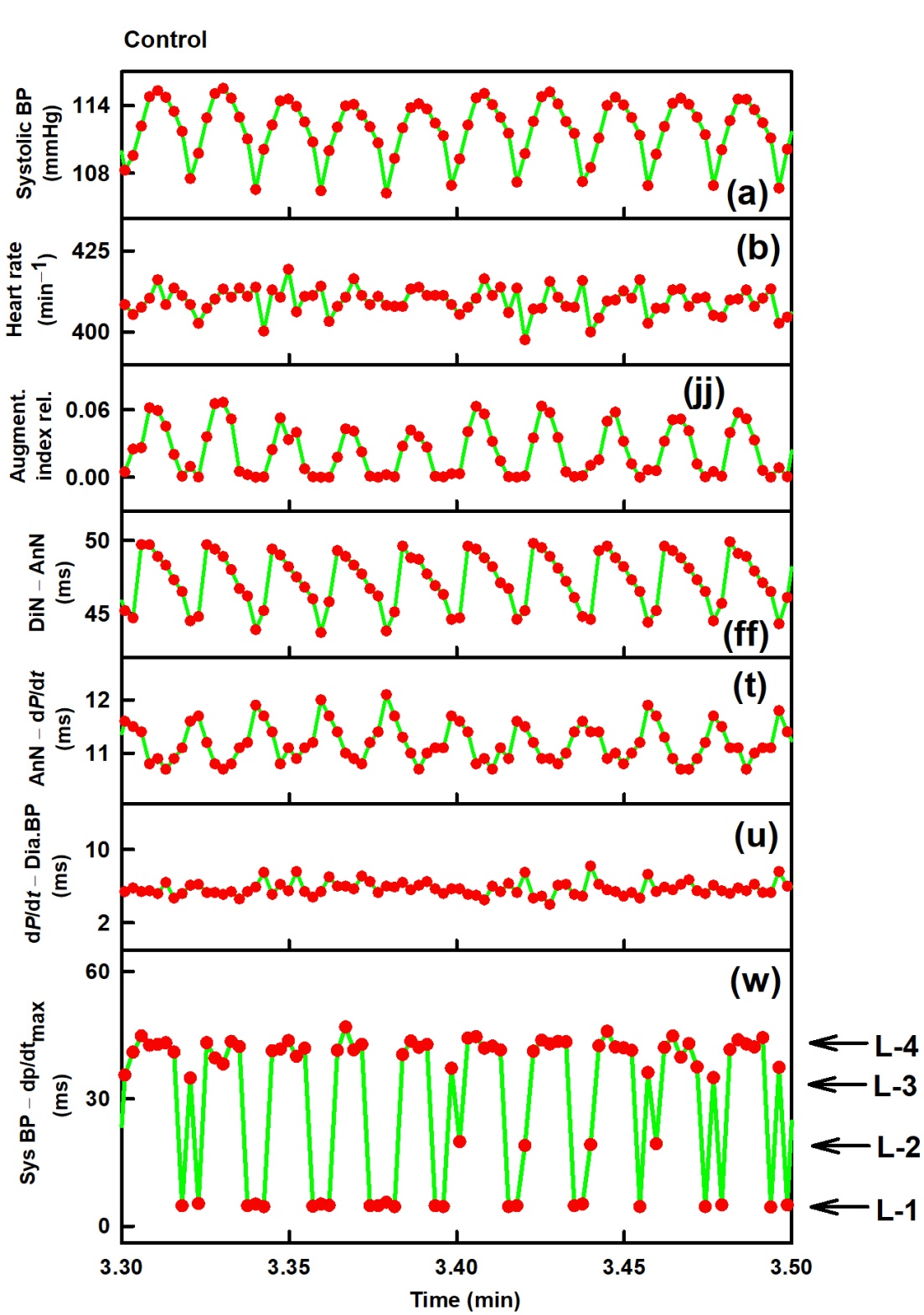


FIGURE S87 ExN-3. Time-dependent changes in APW-Ps of isoflurene anesthetized normotensive rat in control (red heartbeats). Horizontal arrows indicate predicted L-1 to L-4 levels. The green lines show the connection between adjacent heartbeats. Definitions, units and abbreviations of APW-Ps evaluated from the APW are as explained in Supplementary Information FIGURE S1.


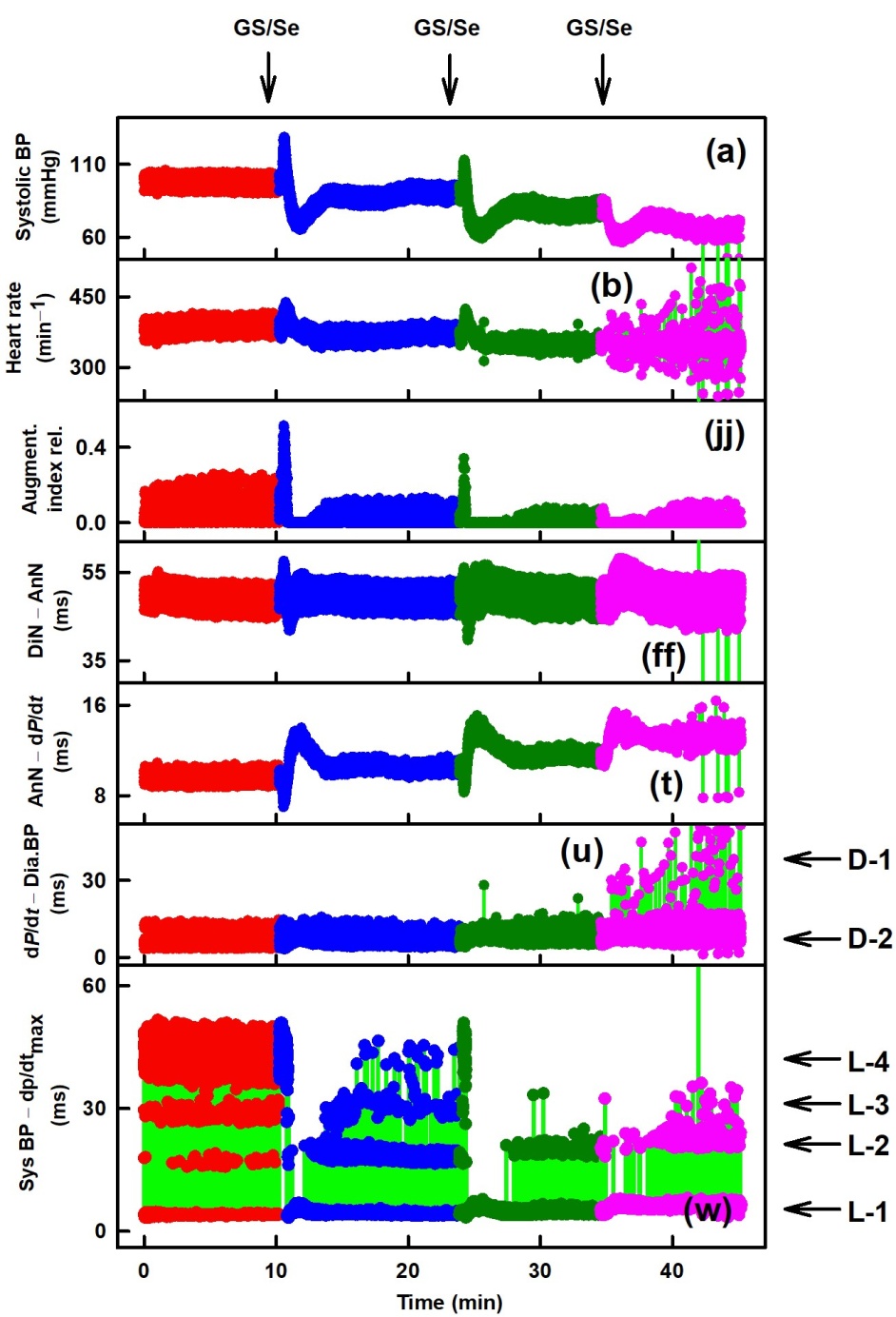


FIGURE S88 ExN-4. Time-dependent changes in APW-Ps of isoflurene anesthetized normotensive rat in control (red heartbeats) and after the subsequent administration of twice GS/Se (75/12.5 in µmol L^–1^, blue and dark green heartbeats), 30 mg kg^–1^ L-NAME (pink heartbeats) followed by GS/Se (75/12.5 in µmol L^–1^, dark cyan heartbeats). Horizontal arrows indicate predicted D-1 and D-2 levels, E-1 and E-2 levels and L-1 to L-4 levels. The green lines show the connection between adjacent heartbeats. Definitions, units and abbreviations of APW-Ps evaluated from the APW are as explained in Supplementary Information FIGURE S1.


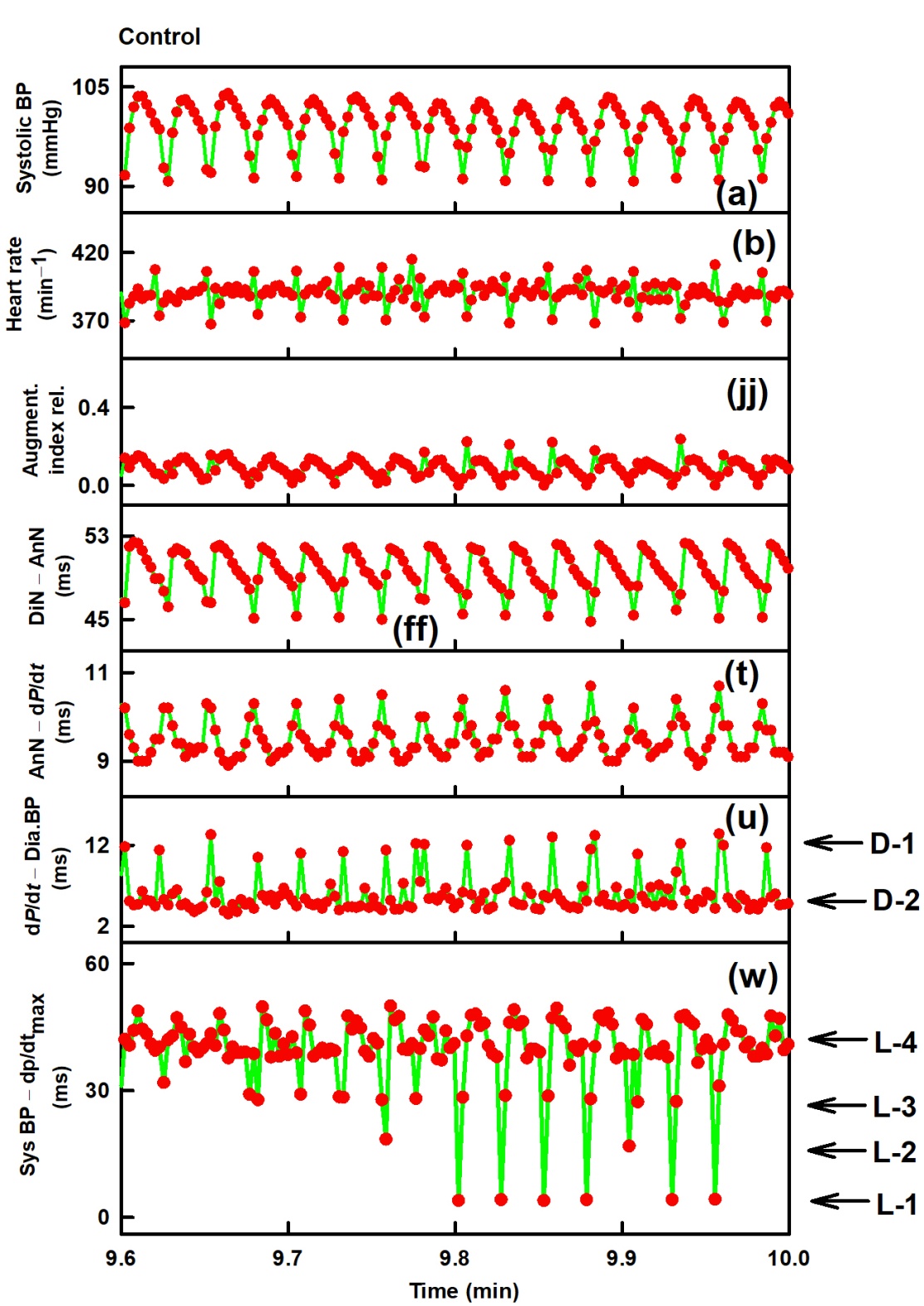


FIGURE S89 ExN-4. Time-dependent changes in APW-Ps of isoflurene anesthetized normotensive rat in control (red heartbeats). Horizontal arrows indicate predicted D-1 and D-2, and L-1 to L-4 levels. The green lines show the connection between adjacent heartbeats. Definitions, units and abbreviations of APW-Ps evaluated from the APW are as explained in Supplementary Information FIGURE S1.


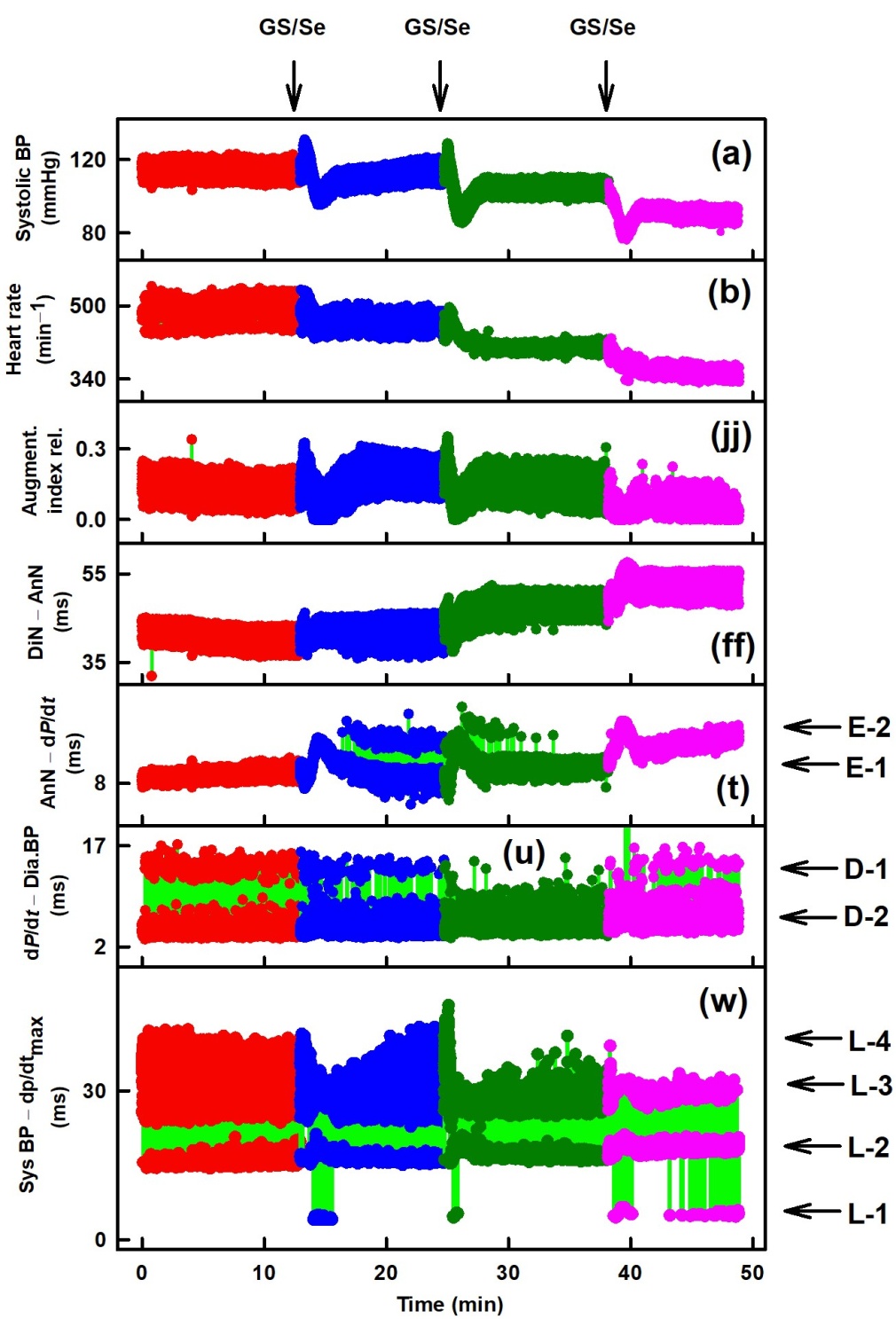


FIGURE S90 ExN-5. Time-dependent changes in APW-Ps of isoflurene anesthetized normotensive rat in control (red heartbeats) and after the subsequent administration of twice GS/Se (75/12.5 in µmol L^–1^, blue and dark green heartbeats), 30 mg kg^–1^ L-NAME (pink heartbeats) followed by GS/Se (75/12.5 in µmol L^–1^, dark cyan heartbeats). Horizontal arrows indicate predicted D-1 and D-2 levels, E-1 and E-2 levels and L-1 to L-4 levels. The green lines show the connection between adjacent heartbeats. Definitions, units and abbreviations of APW-Ps evaluated from the APW are as explained in Supplementary Information FIGURE S1.


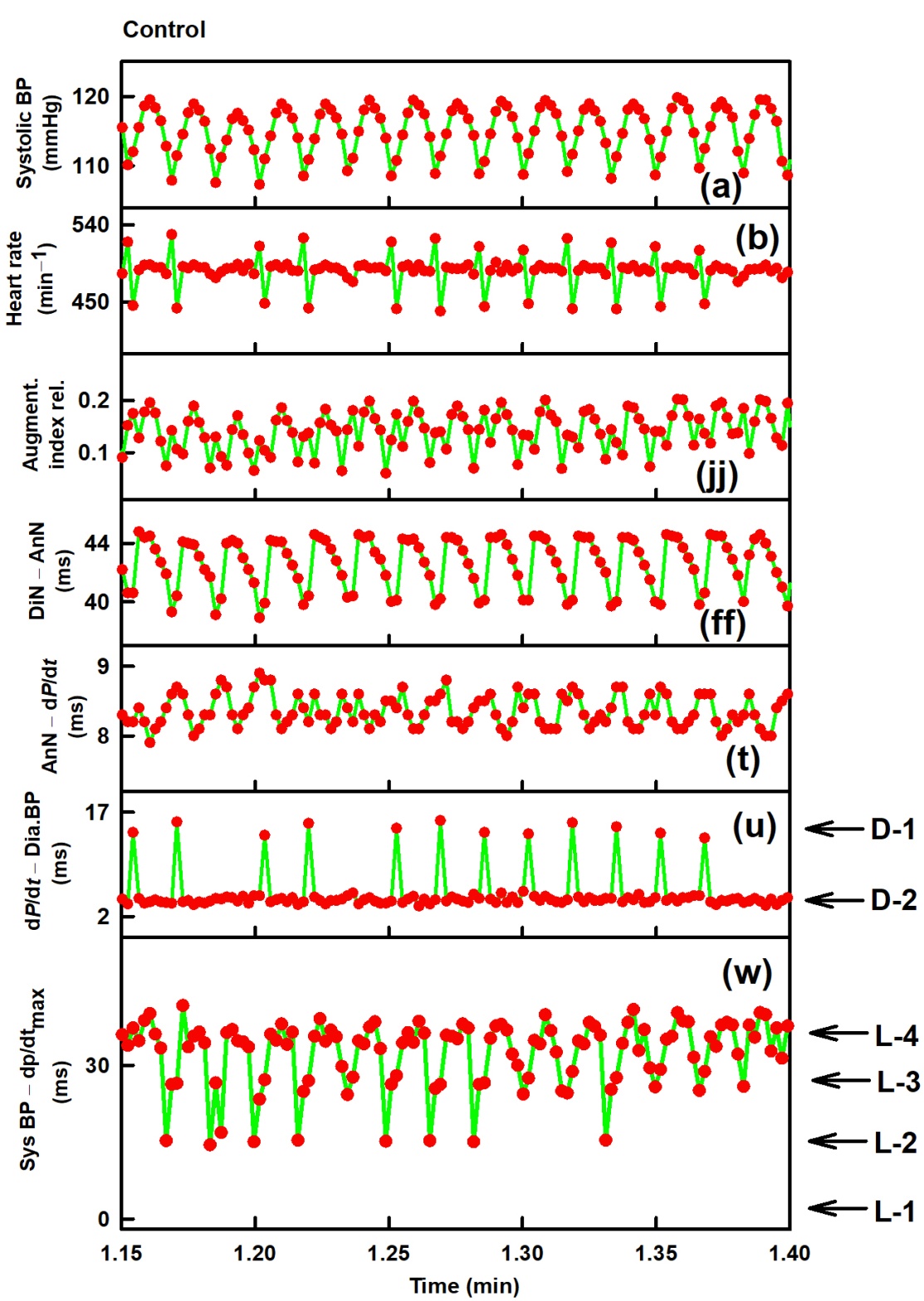


FIGURE S91 ExN-5. Time-dependent changes in APW-Ps of isoflurene anesthetized normotensive rat in control (red heartbeats). Horizontal arrows indicate predicted D-1 and D-2, and L-1 to L-4 levels. The green lines show the connection between adjacent heartbeats. Definitions, units and abbreviations of APW-Ps evaluated from the APW are as explained in Supplementary Information FIGURE S1.


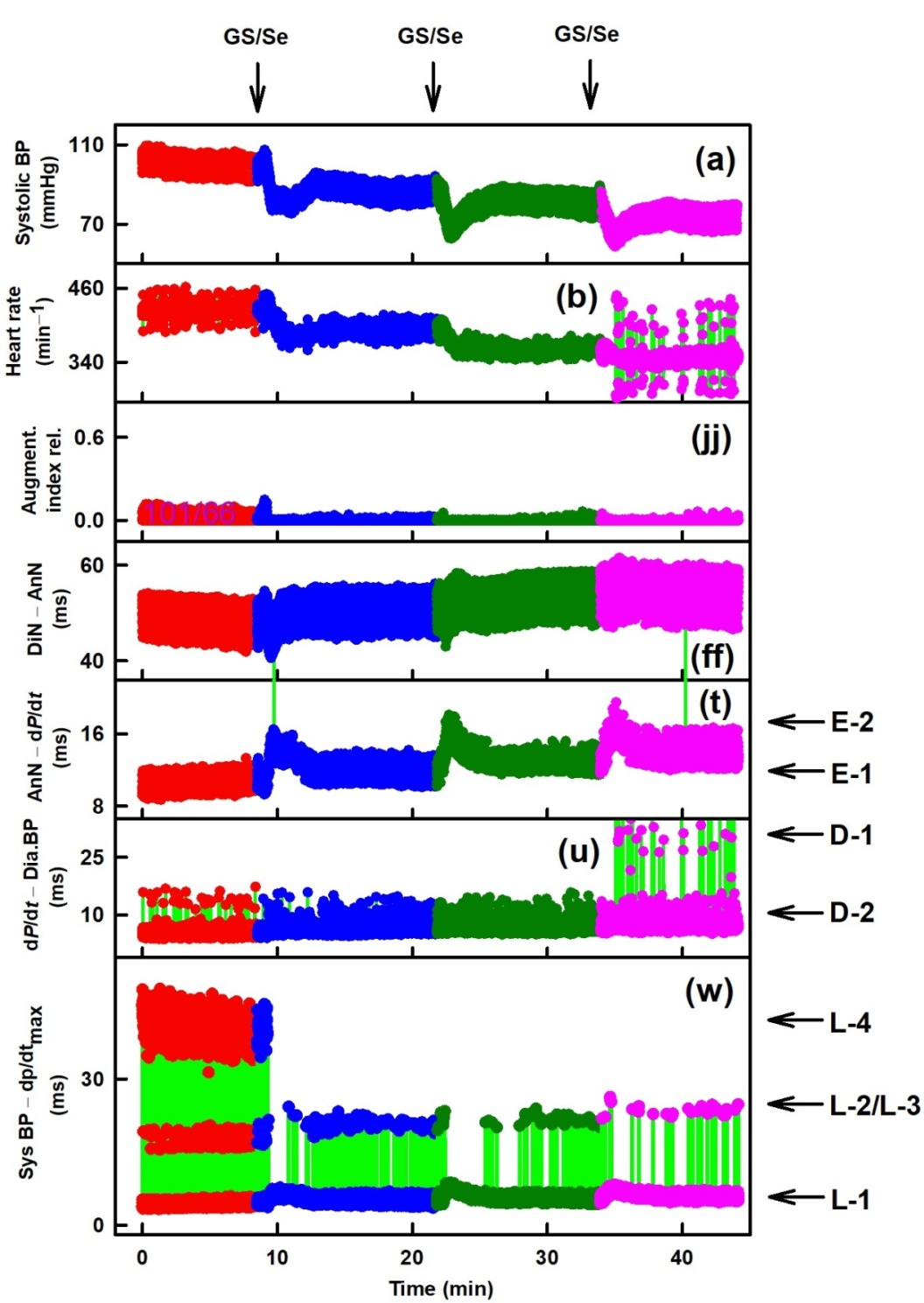


FIGURE S92 ExN-6. Time-dependent changes in APW-Ps of isoflurene anesthetized normotensive rat in control (red heartbeats) and after the subsequent administration of twice GS/Se (75/12.5 in µmol L^–1^, blue and dark green heartbeats), 30 mg kg^–1^ L-NAME (pink heartbeats) followed by GS/Se (75/12.5 in µmol L^–1^, dark cyan heartbeats). Horizontal arrows indicate predicted D-1 and D-2 levels, E-1 and E-2 levels and L-1 to L-4 levels. The green lines show the connection between adjacent heartbeats. Definitions, units and abbreviations of APW-Ps evaluated from the APW are as explained in Supplementary Information FIGURE S1.


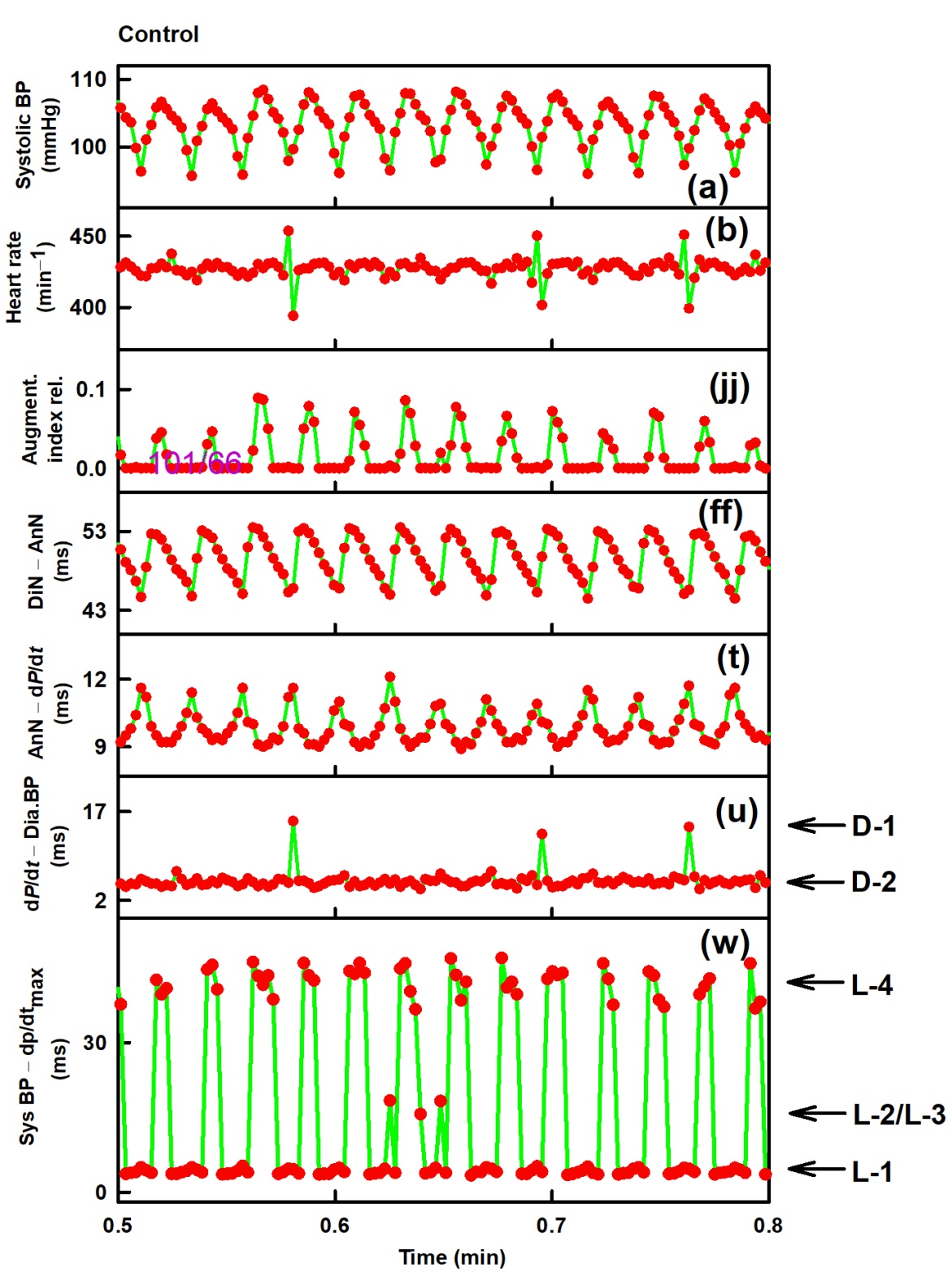


FIGURE S93 ExN-6. Time-dependent changes in APW-Ps of isoflurene anesthetized normotensive rat in control (red heartbeats). Horizontal arrows indicate predicted D-1 and D-2, and L-1 to L-4 levels. The green lines show the connection between adjacent heartbeats. Definitions, units and abbreviations of APW-Ps evaluated from the APW are as explained in Supplementary Information FIGURE S1.


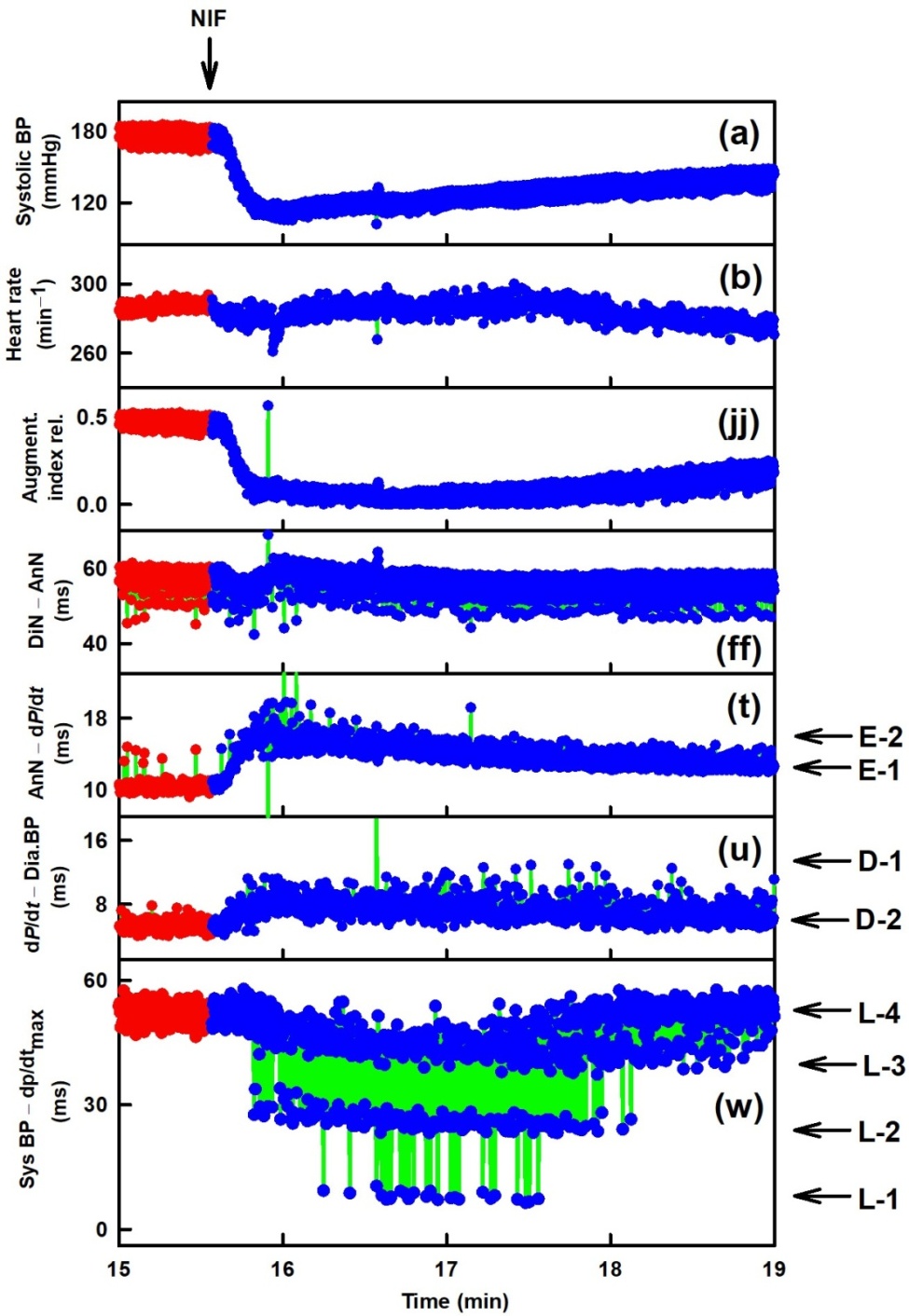


FIGURE S94 SHR-1. Time-dependent changes in APW-Ps of isoflurene anesthetized SHR rat. Control (red heartbeats) and after i.v. administration of NIF (400 nmol L^–1^, blue heartbeats). Horizontal arrows indicate predicted D-1 and D-2 levels, E-1 and E-2 levels and L-1 to L-4 levels. The green lines show the connection between adjacent heartbeats. Definitions, units and abbreviations of APW-Ps evaluated from the APW are as explained in Supplementary Information FIGURE S1.


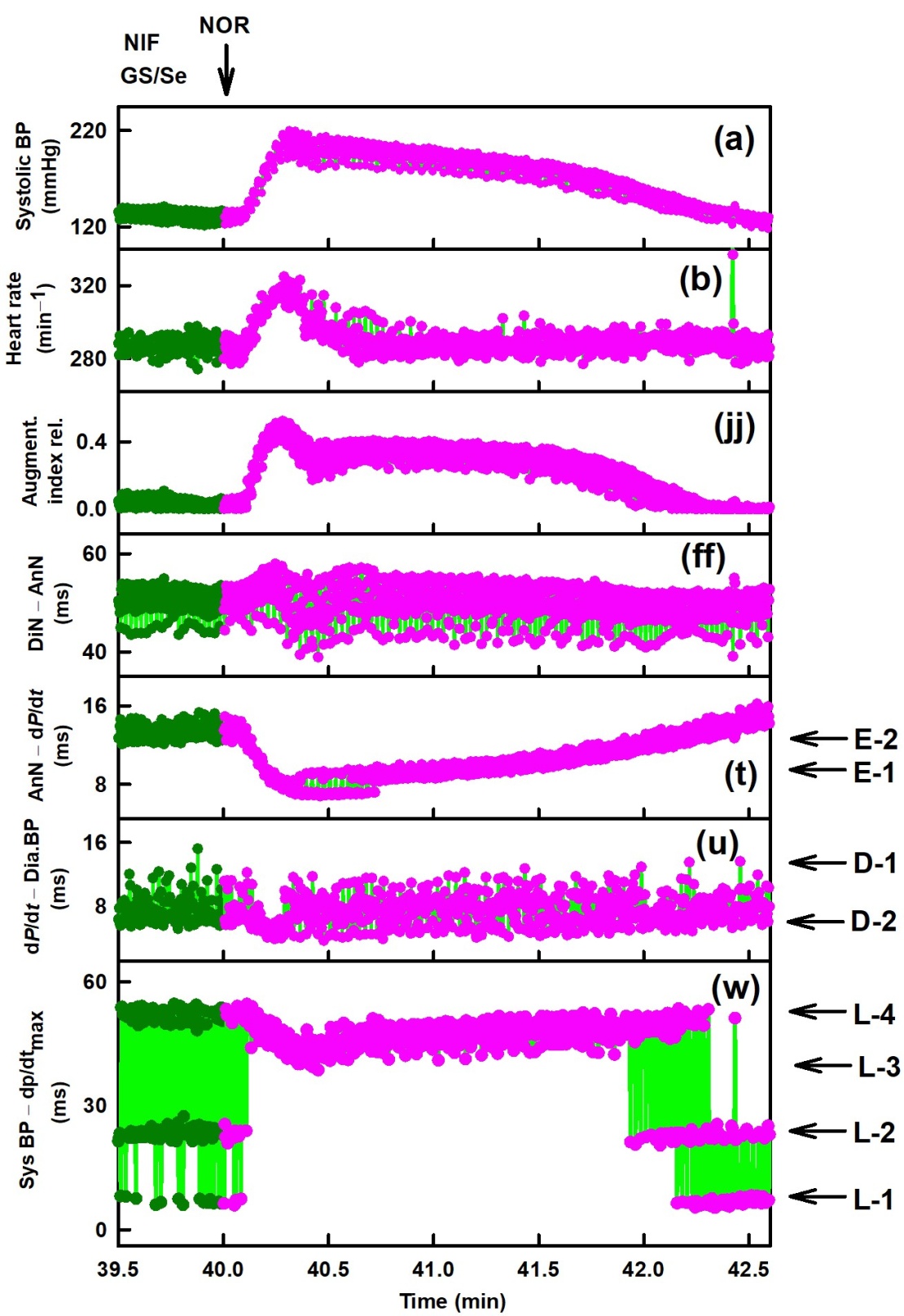


FIGURE S95 SHR-1. Time-dependent changes in APW-Ps of isoflurene anesthetized SHR rat in the presence of NIF (400 nmol L^–1^), GS/Se (75/12.5 in µmol L^–1^) after administration of NOR (0.5 µg kg^–1^, pink heartbeats). Horizontal arrows indicate predicted D-1 and D-2 levels, E-1 and E-2 levels and L-1 to L-4 levels. The green lines show the connection between adjacent heartbeats. Definitions, units and abbreviations of APW-Ps evaluated from the APW are as explained in Supplementary Information FIGURE S1.


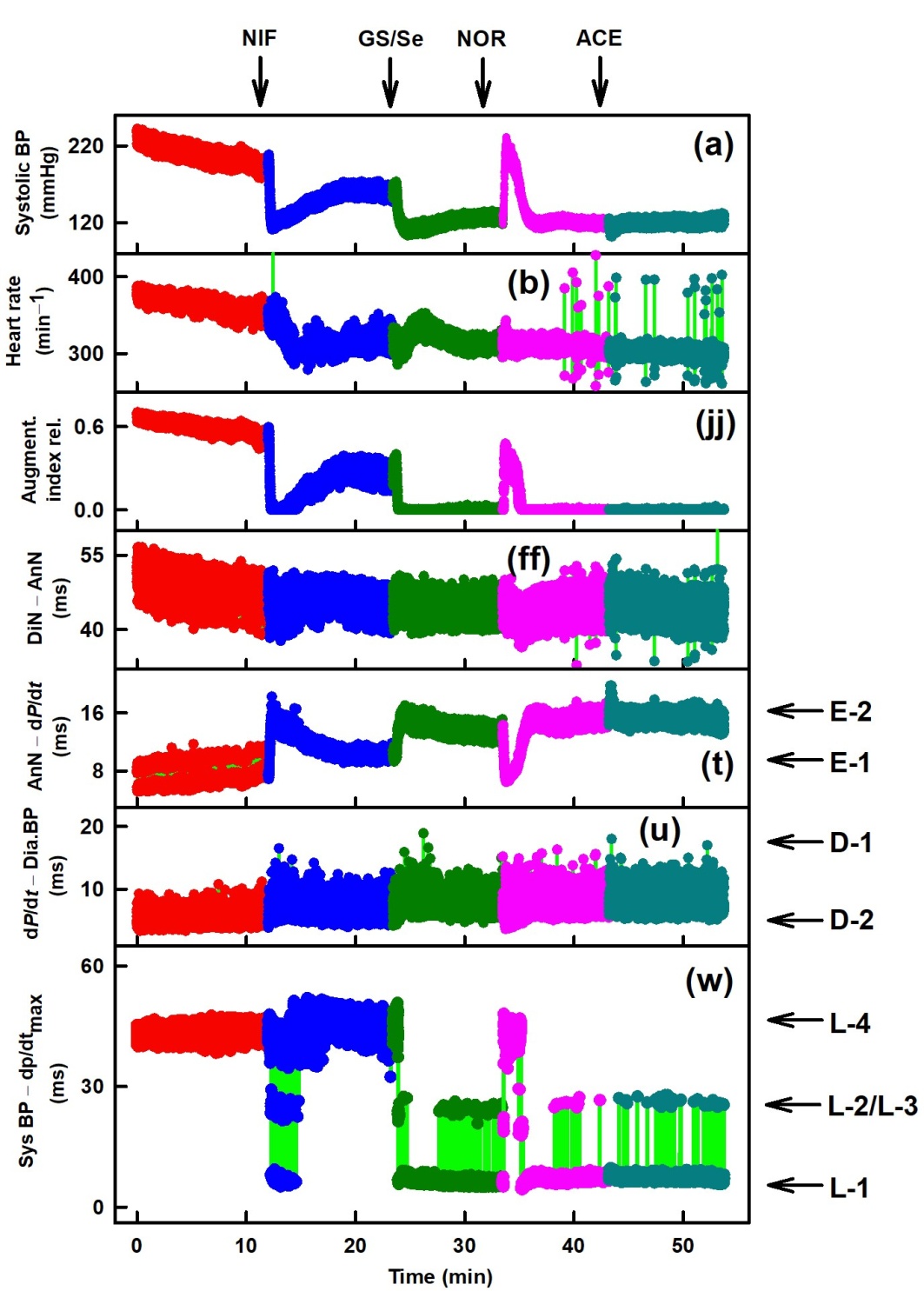


FIGURE S96 SHR-2. Time-dependent changes in APW-Ps of isoflurene anesthetized SHR rat in control (red heartbeats) and after administration of NIF (400 nmol L^–1^, blue heartbeats), GS/Se (75/12.5 in µmol L^–1^, dark green heart beats), NOR (0.5 µg kg^–1^, pink heartbeats) and ACE (1 µg kg^–1^, dark cyan heartbeats). Horizontal arrows indicate predicted D-1 and D-2, E-1 and E-2 and L-1 to L-4 levels. The green lines show the connection between adjacent heartbeats. Definitions, units and abbreviations of APW-Ps evaluated from the APW are as explained in Supplementary Information FIGURE S1.


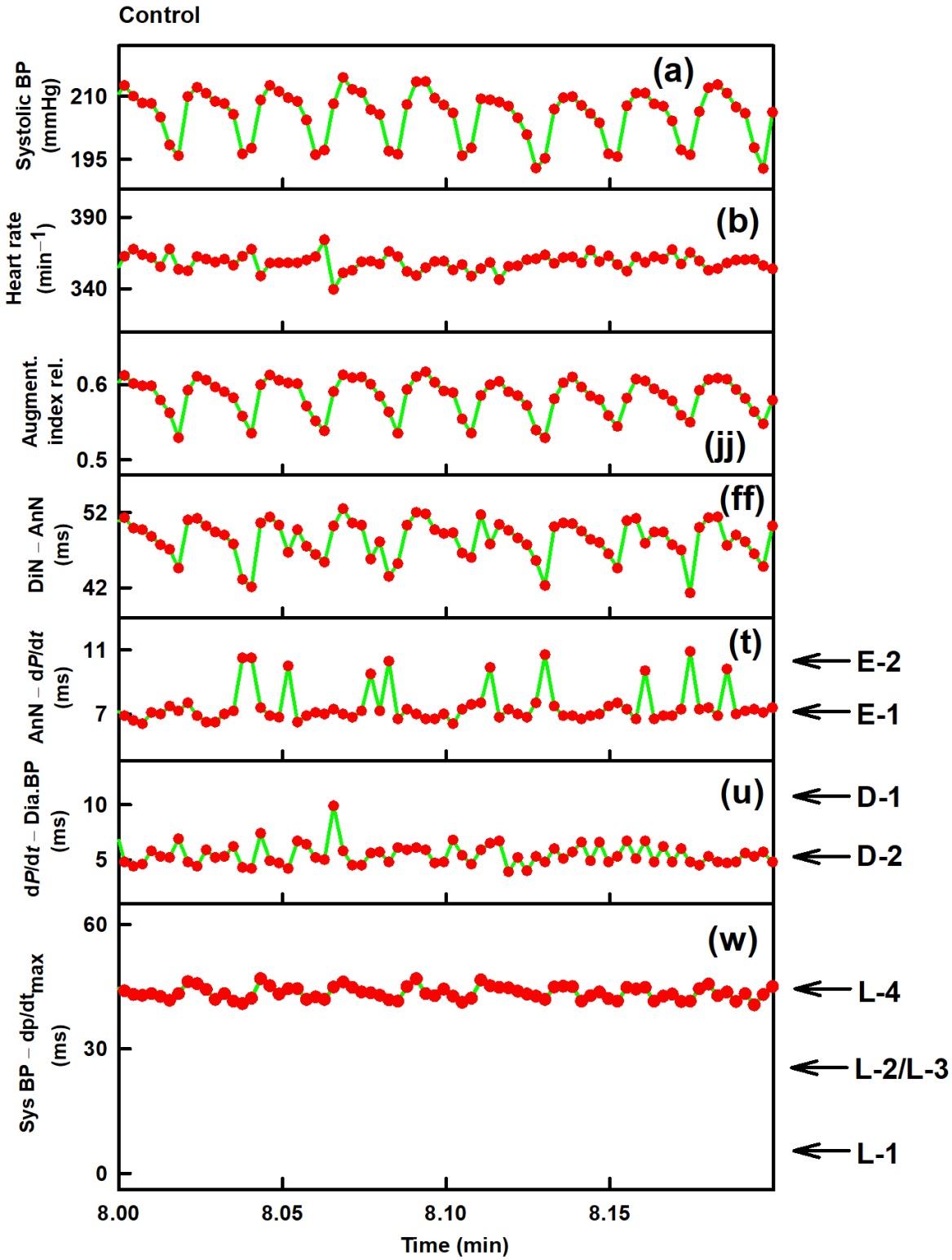


FIGURE S97 SHR-2. Time-dependent changes in APW-Ps of isoflurene anesthetized SHR rat in control (red heartbeats). Horizontal arrows indicate predicted D-1 and D-2, E-1 and E-2 and L-1 to L-4 levels. The green lines show the connection between adjacent heartbeats. Definitions, units and abbreviations of APW-Ps evaluated from the APW are as explained in Supplementary Information FIGURE S1.


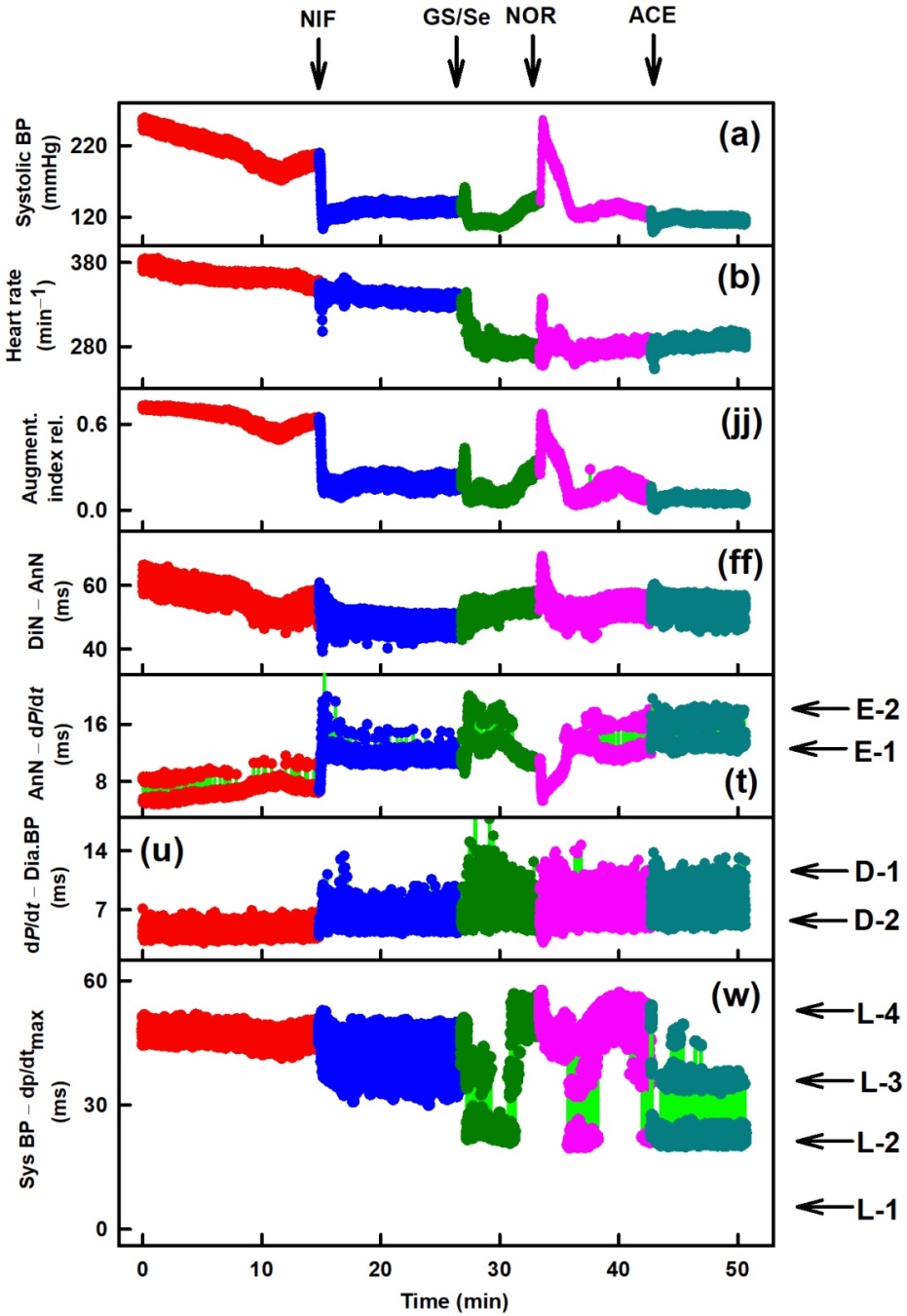


FIGURE S98 SHR-3. Time-dependent changes in APW-Ps of isoflurene anesthetized SHR rat in control (red heartbeats) and after administration of NIF (400 nmol L^–1^, blue heartbeats), GS/Se (75/12.5 in µmol L^–1^, dark green heart beats), NOR (0.5 µg kg^–1^, pink heartbeats) and ACE (1 µg kg^–1^, dark cyan heartbeats). Horizontal arrows indicate predicted D-1 and D-2, E-1 and E-2 and L-1 to L-4 levels. The green lines show the connection between adjacent heartbeats. Definitions, units and abbreviations of APW-Ps evaluated from the APW are as explained in Supplementary Information FIGURE S1.

FIGURE S99 SHR-3. Time-dependent changes in APW-Ps of isoflurene anesthetized SHR rat in control (red heartbeats). Horizontal arrows indicate predicted D-1 and D-2, E-1 and E-2 and L-1 to L-4 levels. The green lines show the connection between adjacent heartbeats. Definitions, units and abbreviations of APW-Ps evaluated from the APW are as explained in Supplementary Information FIGURE S1.

FIGURE S100 SHR-4. Time-dependent changes in APW-Ps of isoflurene anesthetized SHR rat in control (red heartbeats) and after administration of NIF (400 nmol L^–1^, blue heartbeats), GS/Se (75/12.5 in µmol L^–1^, dark green heart beats), NOR (0.5 µg kg^–1^, pink heartbeats) and ACE (1 µg kg^–1^, dark cyan heartbeats). Horizontal arrows indicate predicted D-1 and D-2, E-1 and E-2 and L-1 to L-4 levels. The green lines show the connection between adjacent heartbeats. Definitions, units and abbreviations of APW-Ps evaluated from the APW are as explained in Supplementary Information FIGURE S1.

FIGURE S101 SHR-4. Time-dependent changes in APW-Ps of isoflurene anesthetized SHR rat in control (red heartbeats). Horizontal arrows indicate predicted D-1 and D-2, E-1 and E-2 and L-1 to L-4 levels. The green lines show the connection between adjacent heartbeats. Definitions, units and abbreviations of APW-Ps evaluated from the APW are as explained in Supplementary Information FIGURE S1.

FIGURE S102 SHR-5. Time-dependent changes in APW-Ps of isoflurene anesthetized SHR rat in control (red heartbeats) and after administration of NIF (400 nmol L^–1^, blue heartbeats), GS/Se (75/12.5 in µmol L^–1^, dark green heart beats), NOR (0.5 µg kg^–1^, pink heartbeats) and ACE (1 µg kg^–1^, dark cyan heartbeats). Horizontal arrows indicate predicted D-1 and D-2, E-1 and E-2 and L-1 to L-4 levels. The green lines show the connection between adjacent heartbeats. Definitions, units and abbreviations of APW-Ps evaluated from the APW are as explained in Supplementary Information FIGURE S1.

FIGURE S103 SHR-5. Time-dependent changes in APW-Ps of isoflurene anesthetized SHR rat in control (red heartbeats). Horizontal arrows indicate predicted D-1 and D-2, E-1 and E-2 and L-1 to L-4 levels. The green lines show the connection between adjacent heartbeats. Definitions, units and abbreviations of APW-Ps evaluated from the APW are as explained in Supplementary Information FIGURE S1.

FIGURE S104 SHR-6. Time-dependent changes in APW-Ps of isoflurene anesthetized SHR rat in control (red heartbeats) and after administration of NIF (400 nmol L^–1^, blue heartbeats), GS/Se (75/12.5 in µmol L^–1^, dark green heart beats), NOR (0.5 µg kg^–1^, pink heartbeats) and ACE (1 µg kg^–1^, dark cyan heartbeats). Horizontal arrows indicate predicted D-1 and D-2, E-1 and E-2 and L-1 to L-4 levels. The green lines show the connection between adjacent heartbeats. Definitions, units and abbreviations of APW-Ps evaluated from the APW are as explained in Supplementary Information FIGURE S1.

FIGURE S105 SHR-6. Time-dependent changes in APW-Ps of isoflurene anesthetized SHR rat in control (red heartbeats). Horizontal arrows indicate predicted D-1 and D-2, E-1 and E-2 and L-1 to L-4 levels. The green lines show the connection between adjacent heartbeats. Definitions, units and abbreviations of APW-Ps evaluated from the APW are as explained in Supplementary Information FIGURE S1.

FIGURE S106 The minimal values of augmentation index at L-4 level in controls (red) and after administration of two times glutathione/selenite (GS/Se, 75/12.5 in µmol L^–1^, blue, dark green) and 30 mg kg^–1^ L-NAME (pink). Data are from ExN-1 (Figure 10) and from Supplementary Information Figures S84-S92. The numbers above the bars are the number of experiments. Means ± SD. Pink, cyan, green and blue points shows values in particular experiments. Normotensive rats were anesthetized with isoflurene.

FIGURE S107 The minimal values of augmentation index at L-4 level in controls (red) and after administration of 200 and 400 nmol kg^–1^ NIF (blue), glutathione/selenite (GS/Se, 75/12.5 in µmol L^–1^, dark green) 0.5 µg kg^–1^ NOR (pink). Data are from SHR-1 (Figure 12) and from Supplementary Information Figures S94-105. The numbers above the bars are the number of experiments. Means ± SD. Pink, cyan, green and blue points shows values in particular experiments. SHR rats were anesthetized with isoflurene.

TABLE S1 Systolic BP range in mmHg where n-gital transitions L-levels (l-e), di-gital transitions (D-1/D-2) and (E-1/E-2) were observed. Normotensive rats were anesthetized with Zoletil 100 (tiletamine+zolazepam, 80 mg kg^–1^, i.p.) and xylazine (5 mg kg^–1^, i.p.)

|  | Control | Control | Control | L-NAME | L-NAME | L-NAME | NIF | NIF | NIF |
| --- | --- | --- | --- | --- | --- | --- | --- | --- | --- |
|  | (l-e) | (D-1/D-2) | (E-1/E-2) | (l-e) | (D-1/D-2) | (E-1/E-2) | (l-e) | (D-1/D-2) | (E-1/E-2) |
|  | mmHg | mmHg | mmHg | mmHg | mmHg | mmHg | mmHg | mmHg | mmHg |
| Exp-1 | 97-105 | - | - | 98-106 | ~100-~200 | - | <94-106 | ~100-140 | ~100-140 |
| Exp-2 | 104-126 | - | - | 110-116 | - | - | 107-112 | 105-163 | - |
| Exp-3 | - | - | - | 101-110 | 97-106 | - | 95-105 | 101-122 | - |
| Exp-4 | - | - | - | 104-107 | - | 106-117 | 94-104 | 94-127 | 95-98 |
| Exp-5 | - | - | - | 95-104 | 91-165 | 145-165 | 77-105 | 96-146 | 92-103 |
| Exp-6 | 89-98 | 89-120 | - | 90-96 | 89-169 | 161-168 | 83-97 | 95-117 | 84-119 |
| Exp-7 | 105-120 | 99-111 | - | 106-112 | 97-110 | 159-199 | 103-115 | 104-186 | 117-182 |
| Exp-8 | 106-119 | 102-130 | - | 107-117 | 99-208 | 187-209 | 105-120 | 104-172 | 116-152 |
| Exp-9 | - | - | - | 103-115 | 103-138 | - | 102-121 | 102-138 | 120-152 |
| Exp-10 | 85-104 | - | - | 83-91 | 72-177 | - | - | 123-148 | 123-148 |

TABLE S2 Average values of three main peaks of parameter (l-e) histograms from ‘n‘ experiments of control without and with L-NAME and NIF. Data are from FIGURES S57,S58, normalized to HR 300 min^–1^. Normotensive rats were anesthetized with Zoletil 100 (tiletamine+zolazepam, 80 mg kg^–1^, i.p.) and xylazine (5 mg kg^–1^, i.p.)

| Peaks of  histogram | (l-e) APW  Means ± SD  (ms, 300 HR) | n |
| --- | --- | --- |
| **Control** |  |  |
| L-1 | -28.6 ± 1.9 | 10 |
| L-2/L3 | -14 ± 1.7 | 6 |
| L-4 | 15.4 ± 1.7 | 5 |
| **L-NAME** |  |  |
| L-1 | -29.4 ± 1.8 | 10 |
| L-2/L3 | -12.3 ± 2.8 | 4 |
| L-4 | 8.9 ± 2.0 | 9 |
| **NIF** |  |  |
| L-1 | -26.4 ± 2.0 | 9 |
| L-2/L3 | -12.7 ± 3.2 | 8 |
| L-4 | 11.5 ± 3.1 | 10 |

|  |  |  |  |
| --- | --- | --- | --- |
|  |  |  |  |
|  |  |  |  |
|  |  |  |  |
|  |  |  |  |
|  |  |  |  |
|  |  |  |  |
|  |  |  |  |
|  |  |  |  |
|  |  |  |  |
|  |  |  |  |
|  |  |  |  |

|  |  |  |
| --- | --- | --- |
|  |  |  |
|  |  |  |
|  |  |  |
|  |  |  |
|  |  |  |
|  |  |  |
|  |  |  |
|  |  |  |
|  |  |  |
|  |  |  |
|  |  |  |
|  |  |  |
|  |  |  |
|  |  |  |
|  |  |  |
|  |  |  |
|  |  |  |
|  |  |  |
|  |  |  |
|  |  |  |
|  |  |  |
|  |  |  |
|  |  |  |
|  |  |  |

**References**

Misak A, Kurakova L, Berenyiova A, Tomasova L, Grman M, Cacanyiova S, Ondrias K. Patterns and direct/indirect signaling pathways in cardiovascular system in the condition of transient increase of NO. BioMed Res Int. 2020;6578213. doi:10.1155/2020/6578213.

Kurakova L, Misak A, Tomasova L, Cacanyiova S, Berenyiova A, Ondriasova E, Balis P, Grman M, Ondrias K. Mathematical relationships of patterns of 35 rat haemodynamic parameters for conditions of hypertension resulting from decreased nitric oxide bioavailability. Exp Physiol. 2020;105:312–334. Doi:10.1113/EP088148.

Tomasova L, Grman M, Misak A, Kurakova L, Ondriasova E, Ondrias K. Cardiovascular “patterns” of H_2_S and SSNO-mix evaluated from 35 rat hemodynamic parameters. Biomolecules. 2021;11:293. Doi:[10.3390/biom11020293](https://doi.org/10.3390/biom11020293).

Misak A, Grman M, Tomasova L, Makara O, Rostakova Z, Waczulikova I, Ondrias K. Use of a rat model to characterize 35 arterial pulse wave parameters in a comparative study of isoflurane and Zoletil/xylazine anesthesia and the effect of Acanthopanax senticosus extract. Anim Models Exp Med. 2023:6:474-488. Doi:10.1002/ame2.12354.
